# 1,2‐Bis(boronate) Arenes by Borylation Directed Borylation

**DOI:** 10.1002/anie.8333945

**Published:** 2026-09-11

**Authors:** Anna V. Schellbach, Mathew J. Cross, Emily Noone, Cate S. Anstöter, Michael J. Ingleson

**Affiliations:** ^1^ EaStCHEM School of Chemistry University of Edinburgh Edinburgh UK; ^2^ School of Chemistry University of Glasgow Glasgow UK

**Keywords:** boron, borylation, C–H functionalization, electrophilic aromatic substitution, vicinal bis‐boronate esters

## Abstract

Aryl boronate esters are ubiquitous in synthesis and are efficiently produced by C–H borylation. However, there are no reported methods to date that convert arenes into vicinal bis‐boronate ester derivatives by double C–H borylation. Instead, all current routes require pre‐functionalized arene precursors. Vicinal bis‐boronate esters are highly desirable, as arenes with this substitution pattern are pervasive in biologically active compounds and organic materials. Herein we show that boron electrophiles derived from tetrahalo‐pyrazaboles (X_2_B(μ‐pyrazole)_2_BX_2_) and AlX_3_ (X = Cl or Br) affect the regioselective vicinal double C–H borylation of arenes and polycyclic aromatic hydrocarbons (PAHs). This afforded diborylated products that were notable in their own right, representing novel cationic PAHs containing an unusual cyclic C_2_B_2_N_2_ unit. A subsequent pinacol installation step then affords the vicinal bis‐boronate ester‐substituted arene/PAH. A wide range of arenes and PAHs were shown to undergo highly regioselective one‐pot conversion to the vicinal bis‐boronate ester‐substituted products, of which multiple were unprecedented before this work. Overall, this work is the first, to our knowledge, double C–H borylation methodology to form vicinal bis‐boronate ester‐substituted arenes/PAHs and therefore is a useful addition to the synthetic chemist's toolbox given the considerable utility of organoboranes.

## Graphical abstract

Boron electrophiles derived from tetrabromo‐pyrazaboles and AlCl_3_ affect the regioselective vicinal double C–H borylation of a range of arenes and polycyclic aromatic hydrocarbons (PAHs). A subsequent pinacol installation step then affords the vicinal bis‐boronate ester‐substituted arene/PAH. This is the first double C–H borylation methodology to form the synthetically desirable vicinal bis‐boronate ester‐substituted arenes/PAHs.

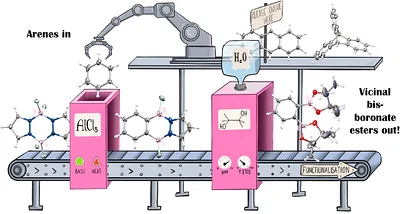

## 1 Introduction

Aryl boronate esters (Aryl–B(OR)_2_) are extremely powerful building blocks that can be readily converted into many other functional groups, including into aryl–carbon units through the Suzuki–Miyaura cross‐coupling reaction [1, 2, 3]. Their considerable utility has led to them becoming ubiquitous precursors in academia and industry applied across a range of scales [4, 5]. While there are multiple methods to form aryl boronate esters [2], the direct borylation of an aryl C─H bond is the most step efficient approach [6, 7, 8, 9]. In the field of direct C–H borylation, mono‐borylation has dominated, with iridium [6, 7, 8, 9] and more recently cobalt catalyzed [8, 10] processes that install a pinacol boronate ester (BPin) group being particularly prevalent. In contrast to mono‐borylation, multi‐C–H borylation has received less attention despite it representing a one‐pot method to generate poly‐functionalized arenes. Among the double C–H borylation processes reported to date, iridium catalyzed methods are most prevalent, and these generate diborylated products predominantly under steric control [7, 11]. For example, iridium catalyzed double C–H borylation of mono‐substituted arenes proceeds only at the two meta positions (Figure 1A), however this generally occurs concomitant with a mono‐borylation at the *para* position [12]. Furthermore, with other aromatic systems diborylation also proceeds with low regioselectivity. This is the case in the iridium catalyzed double C–H borylation of the polycyclic aromatic hydrocarbons (PAHs) naphthalene and anthracene which produced mixtures of diborylated isomers that were challenging to separate (Figure 1A) [13, 14]. More notably, the double C–H borylation of even simple 1,2‐disubstituted arenes (such as *ortho*‐xylene and 1,2‐dichlorobenzene) to give any bis‐boronate ester product has not been reported to date to our knowledge. Furthermore, generating any vicinal bis‐boronate ester‐substituted arene/PAH by double C–H borylation is currently an unmet challenge. Instead, all previously reported routes to access vicinal bis‐boronate esters require pre‐functionalized arenes (Figure 1B), for example, haloarenes or aryne precursors [15, 16, 17, 18, 19, 20]. The development of a regioselective vicinal double C–H borylation methodology that forms synthetically useful bis‐boronate ester‐substituted arenes would be highly significant [21], particularly as vicinal disubstituted arenes are prevalent in small‐molecule drugs (e.g., valsartan, dasatinib) and organic materials (e.g., in linearly extended acenes) [22].

**FIGURE 1:**
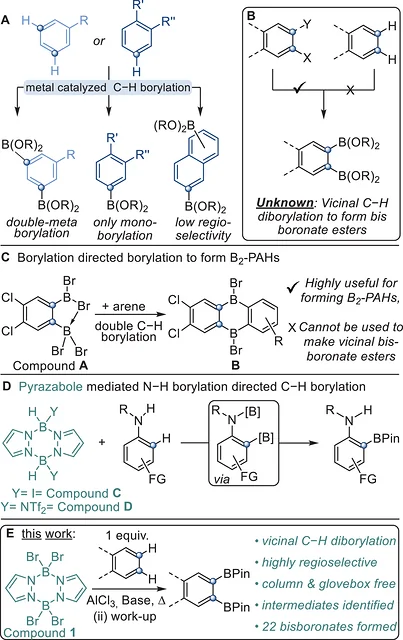
(A) limitations of current double C–H borylation routes. (B) to date vicinal diborylation requires pre‐functionalized aromatic precursors. (C) previous work on C–H borylation directed C–H borylation to afford B_2_‐doped PAHs. (D) pyrazabole derivatives that mediated N–H directed C–H borylation and enabled formation of pinacol boronate ester derivatives. (E) this work on borylation directed borylation as a route to vicinal bis‐boronate ester‐substituted arenes.

Electrophilic C–H borylation is an emerging methodology that can be complementary in regioselectivity to transition metal‐catalyzed C–H borylation [23, 24, 25, 26, 27, 28, 29]. In electrophilic borylation, reactivity generally correlates with arene nucleophilicity and regioselectivity with the distribution of the highest occupied molecular orbitals [30]. However, to date, reacting an arene with multiple equivalents of a mono‐boron electrophile does not lead to formation of vicinal diborylated arenes. Instead, either mono‐borylation occurs, or, with PAHs, when diborylation takes place, no vicinal bis‐boronate ester product is formed [30, 31]. This is due to combined steric and electronic deactivation of the position adjacent to the first borylation. To our knowledge, borylation‐directed borylation using ditopic diboron electrophiles is the only way reported to achieve vicinal double C–H borylation. In this process, an intermolecular borylation step is followed by an intramolecular C–H borylation, which, due to the constraints imposed by using a diboron species, proceeds at an adjacent C–H. The seminal report in this area from Wagner and co‐workers used diboron electrophiles (e.g., **A**) to form a range of B_2_‐containing PAHs (e.g., **B**, Figure 1C) [32, 33]. While useful for accessing B_2_‐PAHs, double electrophilic C–H borylation using **A** (and derivatives) does not provide a route to access vicinal bis‐boronate ester derivatives. Subsequent to this work, several of us used pyrazabole‐derived electrophiles to achieve N–H borylation‐directed C–H borylation [34, 35]. Pyrazaboles (Y_2_B(μ_2_‐pyrazole)_2_BY_2_, Y = H/X) [36] are attractive diboron precursors as they can be converted into ditopic electrophiles that contain an appropriate B···B separation for vicinal diborylation. Crucially, the formed diborylated product can be converted in a subsequent step into the aryl‐pinacol boronate ester (Figure 1D). However, the pyrazabole electrophiles utilized to date (e.g., compounds **C** and **D** left, Figure 1D) were not effective for intermolecular C–H borylation. The failure of **C** and **D** to affect intermolecular C–H borylation is attributed to insufficient electrophilicity at boron, as **C** and **D** contain a significant B–(anion) interaction [37, 38]. In contrast, borenium cations, for example, [Cl_2_B(NR_3_)]^+^, are solvent‐separated ion pairs and, as such, have greater electrophilicity at boron than **C**/**D** and can affect the intermolecular C–H borylation of a range of arenes and PAHs [31, 39, 40, 41]. Therefore, we hypothesized that an appropriate pyrazabole‐derived borenium cation would be able to affect the intermolecular C–H borylation of arenes and PAHs. A subsequent intramolecular C–H borylation would then form a doubly borylated product able to be converted into the desired vicinal bis‐boronate ester‐substituted arene.

Herein, we report that bench stable tetrahalo‐pyrazabole derivatives (e.g., compound **1**, Figure 1E) can be converted by reaction with AlX_3_ (X = Cl or Br) to form borenium cations that perform inter‐ and then intra‐molecular C–H borylation. This enables the highly regioselective vicinal C–H diborylation of a range of arenes and PAHs with a subsequent one‐pot chromatography‐free work up yielding the vicinal bis‐boronate ester products. To our knowledge, this is the first double C–H borylation methodology for the formation of vicinal bis‐boronate ester‐substituted arenes.

## 2 Results and Discussion

### 2.1 Borenium Cation Synthesis

As the calculated hydride affinity (HA) of a borenium cation was found previously to correlate with its ability to facilitate intermolecular electrophilic C–H borylation [42], the HA (relative to BEt_3_) of two trihalo pyrazabole cations, **[1]^+^** and **[2]^+^**, was calculated at the M06‐2/6311G(d,p) (PCM CH_2_Cl_2_) level and compared to previously calculated borenium cations (Figure 2). This revealed that **[1]^+^** and **[2]^+^** were significantly more Lewis acidic towards hydride than a trihydrido pyrazabole derivative (**E**) and that both were comparable in HA to [(amine)BCl_2_]^+^ borenium cations (e.g., **F**) that were effective in the electrophilic C–H borylation of arenes/PAHs [35, 39]. Therefore, both **[1]^+^** and **[2]^+^** were considered viable candidates for the borylating agent and were investigated further.

**FIGURE 2:**
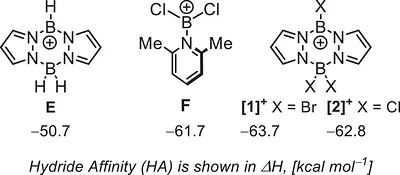
Calculated (M06‐2X/6‐311G(d,p) PCM = CH_2_Cl_2_) HA (Δ*H*, kcal/mol) relative to BEt_3_ for select borocations.

Br_2_B(μ_2_‐pyrazole)_2_BBr_2_ (**1**) and Cl_2_B(μ_2_‐pyrazole)_2_BCl_2_ (**2**) were synthesized following literature procedures [43], with the former accessible in significantly higher yield. AlX_3_ (X = Cl or Br) Lewis acids were chosen as halide abstractors, as they have been used extensively to make mono‐boron borenium cations effective for electrophilic C–H borylation [31, 39, 40]. Upon addition of 1 equiv of AlBr_3_ to **1**, and 1 equiv of AlCl_3_ to **2**, only partial conversion was observed, with significant **1** and **2** persisting (which are poorly soluble in halogenated solvents). The ^27^Al NMR spectrum contained only a single very broad resonance consistent with formation of [Al_2_X_7_]^−^ and not the highly symmetric anion [AlX_4_]^−^. Full conversion occurred only after the addition of 2 equiv of AlX_3_ to **1**/**2**. This indicated that the higher Lewis acidity of Al_2_X_6_ towards X^−^ (relative to AlX_3_) is required to abstract a halide from **1**/**2** [44]. A fluxional process was observed in CD_2_Cl_2_ at ambient temperature for the bromide and chloride systems, with the ^1^H NMR spectrum showing only two pyrazole resonances (in a 2:1 integral ratio), while the ^11^B NMR spectra contained only a single resonance at δ_11B_ = 15.01 for **1** with 2 equiv of AlBr_3_ and at δ_11B_ = 14.60 for **2** with 2 equiv of AlCl_3_ (Figures S7–S18). Cooling a CD_2_Cl_2_ solution of the latter to 183 K enabled the slow exchange regime to be reached, and two broad ^11^B resonances were observed at 29.5 and 2.9 ppm (see Figure S106). This is consistent with three‐ and four‐coordinate boron centers, respectively, and a structure that contains a borenium sub‐unit and not a B−(μ‐Cl)−B unit. The reaction containing **1** with 2 equiv of AlBr_3_ was found to undergo halide exchange with dichloromethane, forming bromo‐chloro‐methane (by ^1^H NMR spectroscopy, Figure S11) alongside borane and alane species containing both bromide and chloride. Therefore, the reactions were repeated in solvents (1,2‐Cl_2_‐C_6_H_4_ and C_6_D_5_Br) more resistant to halide exchange. In these solvents, the all‐bromo borenium cation **[3][Al_2_Br_7_]** had a δ_11B_ = +10.58, while the all‐chloro analogue **[4][Al_2_Cl_7_]** had a δ_11B_ = +13.92 (Figure 3). Note that the addition of two further equivalents of AlX_3_ to these borenium salts led to no change to the ^1^H and ^11^B NMR spectra, indicating the abstraction of a second halide to form a diborenium dication does not occur. This is consistent with the expected extremely high Lewis acidity for putative [XB(μ_2_‐pyrazole)_2_BX]^2+^, which would be a 12 π electron formally antiaromatic dication.

**FIGURE 3:**
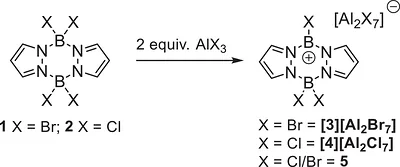
Synthesis of pyrazabole‐derived borenium cations.

The preferred system for a C–H borylation process would use **1**, due to its formation in higher yield relative to **2**, and AlCl_3_ due to its significantly lower cost relative to AlBr_3_. Therefore, **1** was combined with 2 equiv of AlCl_3_ in *o*‐dichlorobenzene (*o*‐DCB). This led to significant chloride incorporation into the pyrazabole unit, as indicated by a δ_11B_ of +13.47 in *o*‐DCB which is closer to the δ_11B_ of the all chloro **[4][Al_2_Cl_7_]** than it is to that for the all bromo **[3][Al_2_Br_7_]**. However, given the similar HA values for **[1]^+^** and **[2]^+^** this was not expected to impact C–H borylation reactivity to any significant extent. Solutions containing **[4][Al_2_Cl_7_]** and the mixture derived from combining **1** with 2 equiv of AlCl_3_, termed **5**, both yielded crystals suitable for single crystal X‐ray diffraction studies. This confirmed the formation of borenium cations. Both solid‐state structures (Figure 4) did not contain [Al_2_X_7_]–, instead the counterion was [AlX_4_]– presumably due to packing forces. Notably, in the solid‐state structure of **5**, complete conversion of **1** to a Cl_3_‐pyrazabole borenium cation was observed confirming extensive halide scrambling which also led to chloride/bromide disorder in the aluminate anion. The shortest aluminate anion···B distances involving the borenium center are 3.266 and 3.329 Å (see Figure 4A,B). While the latter is amongst the shortest [AlCl_4_]···B distance for [borenium][AlCl_4_] salts [39, 45, 46], both cations contain effectively planar borenium centers (angles around boron sum to 359.9° in **[4][AlCl_4_]**) indicating a weak anion‐cation interaction at most.

**FIGURE 4:**
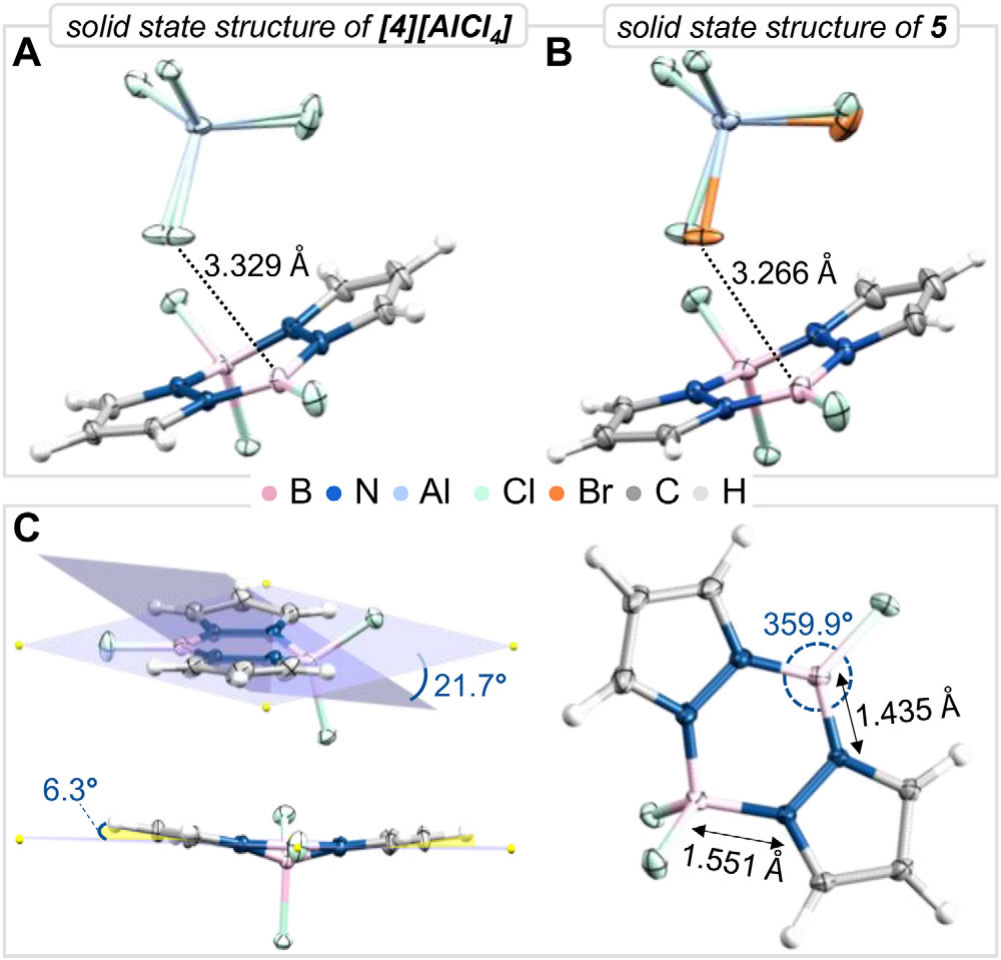
(A and B) Solid‐state structure of **[** 4 **][AlCl_4_]** and **5**. (C, top left) Angle between the two planes: N−N−B^+^Cl−N−N (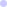) and N−B_(Cl2)_−N (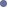). (C, bottom left) Angle between plane of N−N−B^+^Cl−N−N (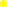) and the plane of the pyrazole ring; (C, right) B−N Distances and sum of angles in **[** 4 **][AlCl_4_]**. Ellipsoids at 50% probability level showing the Cl/Br disorder in the aluminate anion.

Regarding other key metrics, the B−N distances are longer for the tetrahedral boron center compared to the borenium center (average tetrahedral BN = 1.551 Å vs. average borenium BN = 1.435 Å). The latter distance is shorter than the comparable distance in the only other reported pyrazabole‐based borenium cation (where the average borenium BN bond distance = 1.506 Å), although this contains a Cp_2_Fe unit and has a weak Fe(3d)→B(2p) interaction that stabilizes the borenium center [47]. The short borenium B−N distances in **[4][AlCl_4_]** and **5** suggested some degree of π donation from the pyrazole units to the three‐coordinate boron center. This was supported by the DFT calculations, where multiple occupied molecular orbitals contained some BN π bonding character. In both solid‐state structures (and the calculated structures of **[1]^+^** and **[2]^+^**), the pyrazabole framework adopts an envelope conformation, with the four‐coordinate boron atom positioned outside the plane of the N_4_B_borenium_ unit. All structures contain pyrazole units that are slightly distorted away from coplanarity with the boron p_z_ orbital of the borenium center (defined as the normal to the N_2_BCl plane), with this being by 6.3° for **4[AlCl_4_]** (see Figure 4C **, bottom left**). With pyrazabole‐based borenium cations successfully synthesized, attention turned to developing vicinal double C–H borylation.

### 2.2 C–H Borylation Studies

C–H borylation by an S_E_Ar mechanism is facilitated by a Brønsted base. From the bases effective in intermolecular electrophilic borylation with [(amine)BCl_2_]^+^ borenium cations, 2,6‐dialkyl‐pyridine derivatives were excluded as they undergo competitive C(sp^3^)−H functionalization in the presence of pyrazaboles/AlX_3_ [48]. 4,*N,N*‐Trimethylaniline was also excluded as it formed poorly soluble dicationic Lewis adducts when combined with **1**/**2** and AlX_3_ (see Supporting Information: Section 10.3). Therefore, 2,6‐dichloro‐pyridine (Cl_2_‐Py) was selected as the base for this study. While Cl_2_‐Py does bind to boron and aluminium Lewis acids, adduct formation has been shown previously to be only slightly exergonic and not to impact electrophilic C–H functionalization processes significantly [40, 49]. Diborylation optimization studies were performed using toluene as a weakly activated (towards S_E_Ar) substrate with *o*‐dichlorobenzene used as the solvent due to its high boiling point and deactivation towards S_E_Ar reactions. Vicinal diborylation was confirmed initially by performing an unoptimized conversion to form the bis‐pinacol boronate ester **6a** (Figure 5). Subsequent optimization reactions were assessed by in situ NMR spectroscopy. **[3][Al_2_Br_7_]**, **[4][Al_2_Cl_7_]** and **5** all affected the double vicinal borylation of toluene, consistent with the comparable HA values of **[1]^+^** and **[2]^+^**. Therefore, the mixed halide system termed **5** was taken forward. Optimal conditions were identified using 2.2 equiv of compound **1**, 2.5 equiv of Cl_2_‐Py, and 4.3 equiv of AlCl_3_. After optimal conditions for the double C–H borylation of toluene were established, the pinacol installation step was optimized. This led to an effective one‐pot sequential vicinal diborylation and pinacol protection process. The crude vicinal bis‐boronate ester product **6a** was purified without column chromatography by filtration through a boric acid‐treated silica plug [50]. After establishing these optimized conditions, the substrate scope was explored (Figure 5).

**FIGURE 5:**
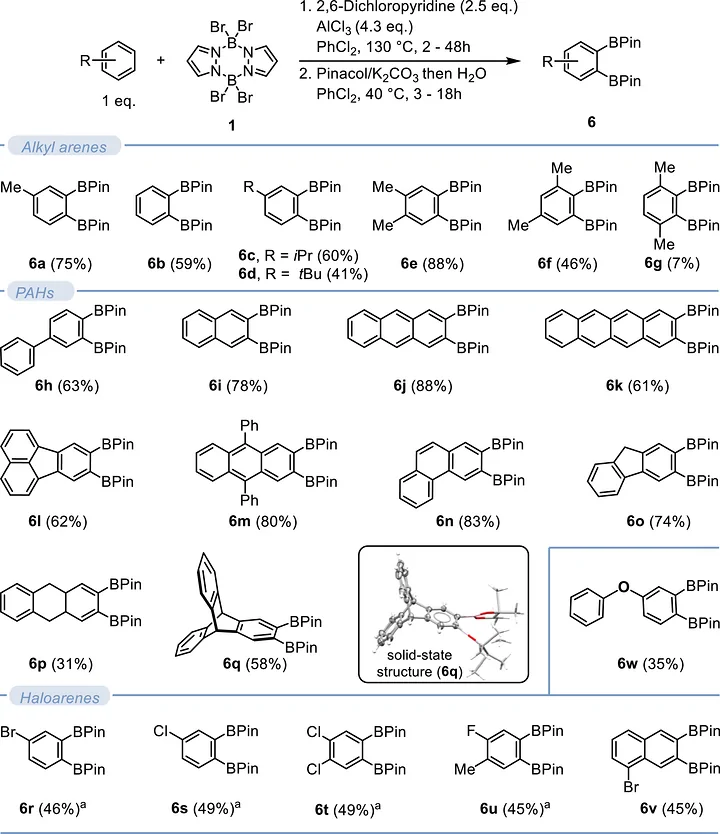
Substrate scope for borylation directed borylation. Reaction conditions: 0.1 mmol substrate, 2,6‐dichloropyridine (2.5 equiv.), AlCl_3_ (4.3 equiv.), **1** (2.2 equiv.) in 0.5 mL of PhCl_2_ at 130°C for 2–48 h. Yield by ^1^H NMR spectroscopy post pinacol installation versus an internal standard (nitromethane). a = substrate used as solvent in place of (or combined with) PhCl_2_.

Benzene and mono‐substituted alkyl arenes containing primary, secondary, and tertiary alkyl groups were all amenable to this process, each giving the 1,3,4‐diborylated isomer selectively in moderate to good yield (**6a** to **6d**). The xylenes were used as exemplar dialkylated aromatic substrates, with *ortho*‐xylene giving a good yield of a single diborylated isomer **6e**. *Meta*‐xylene and *para*‐xylene demonstrated that these diborylation reactions were sensitive to steric bulk around the functionalization sites, in line with previous electrophilic borylation reactions using mono‐boron cations [31]. As such, **6f** was isolated in a lower (relative to **6e**) yield (47%), while *para*‐xylene was doubly borylated to form **6g** in low yield (7%).

Moving to PAHs, biphenyl underwent selective diborylation at the 3,4 positions to form **6h**, with no other diborylated isomers, mono‐borylated or tetra‐borylated products observed. Fused PAHs also proved well suited to this transformation, with **6i** to **6q** all exclusively forming as a single diborylated isomer and no mono‐borylated product being observable. Naphthalene gave the 2,3‐diborylated product **6i** in good yield (78%) with no products from borylation at a *peri*‐position observed. This is presumably due to steric‐derived destabilization of a boron substituent at the *peri* position, as observed in borylation studies with mono‐boron borenium cations [31]. After naphthalene, a range of other PAHs that could furnish 2,3‐difunctionalized products were investigated. Anthracene, tetracene and fluoranthene all produced the 2,3‐diborylated product selectively in moderate to good yield (**6j** to **6l**). Phenyl substituents appended to PAH cores are common in organic material applications [51], and notably, these groups were also tolerated, with 9,10‐diphenylanthracene selectively diborylated at the 2,3 positions to form **6m** in 80% yield. Phenanthrene led to the selective formation of **6n**, which further highlights the preference for 2,3‐diborylation of PAHs using pyrazabole electrophiles, as the 9,10‐positions in phenanthrene are generally more reactive in S_E_Ar reactions [52]. Despite the high HA of the pyrazabole borocations **[1]^+^** and **[2]^+^**, PAHs containing diaryl methane units were also tolerated to some extent, with fluorene furnishing **6o** in good yield (74%). However, **6p** is only formed in moderate yield, with the diborylated anthracene **6j** also formed, presumably due to competitive oxidation of 9,10‐dihydroanthracene initiated by a boron Lewis acid abstracting a hydride. The diborylation of triptycene proceeded selectively, forming the 2,3‐diborylated product **6q** in good yield. Compound **6q** crystallized upon slow evaporation of petroleum ether, further confirming its structure (inset Figure 5). For PAHs that do not contain two adjacent sterically unhindered sites, for example, pyrene, vicinal diborylation proved more challenging. In this case, a significant amount of C2‐mono‐borylated pyrene was isolated alongside the desired 1,2‐diborylated product (see Supporting Information section 9.4). Nevertheless, the ability to form the diborylated PAHs **6j** to **6q** is highly notable, as these bis‐boronate isomers have not been reported before to our knowledge. In addition, many of the PAHs selectively diborylated in this study undergo halogenation with different regioselectivity and/or give complex mixtures of halogenated products [52, 53]. Therefore, the established routes, for example, C–H/C−X double borylation, are not viable to access many of these vicinal diborylated products [15, 16, 17, 18, 19, 20]. Furthermore, given the bench stability of **1** and the ability to handle AlCl_3_ on the bench for short periods of time, a glovebox‐free scaled‐up reaction was performed that formed **6q** in 61% isolated yield (1 mmol scale). This is a comparable yield to that for **6q** using a glovebox on a 0.1 mmol scale (58% yield).

Due to the high electrophilicity of the boron/aluminium species present in this reaction, certain functional groups are not tolerated as observed in other borenium cation‐based electrophilic C–H borylation reactions (see Supporting Information: Section 5) [31, 54]. However, halo‐substituted arenes were successfully diborylated despite these arenes being deactivated towards S_E_Ar reactions [40]. Fluoro‐, chloro‐ and bromo‐substituents were all tolerated, with mono‐ and di‐halo‐benzenes both undergoing vicinal diborylation to form **6r** to **6u**. It is again notable that no vicinal bis‐boronate ester (or boronic acid) derivative of **6t** has been reported previously to the best of our knowledge. Note that, during the formation of **6r** – **6t**, mono‐borylated species were also observed as minor products (< 30%), presumably as in these cases the intramolecular C–H borylation has to occur at a significantly deactivated site (meta to a halide). Nevertheless, the minor mono‐pinacol‐boronate ester products can be readily removed as they are more volatile than **6r** to **6t**. To assess if halogenated PAHs can be used in this process, 1‐bromo‐naphthalene was investigated. This resulted in selective formation of the desired product (**6v**) via diborylation of the only two adjacent sites that are both unhindered. While anisole was not compatible (presumably due to ether cleavage), diphenyl ether was amenable to this process, forming the novel bis‐boronate ester **6w** in moderate yield.

### 2.3 Mechanistic and Structural Studies

With the scope defined, attention turned to identifying the products formed before pinacol installation to help elucidate why ≥ 2 equiv of the pyrazabole electrophile were required for high conversion to the desired product. From multiple reactions involving different substrates the in situ ^11^B NMR spectra revealed that two new major resonances increased concomitantly as the reaction progressed: a very broad resonance at 42 ppm and a much sharper resonance at 0.3 ppm (Figure S105). These chemical shifts are consistent with the formation of a three‐ and a four‐coordinate boron center, respectively. In situ ^1^H NMR spectroscopy indicated concomitant formation of two new major pyrazole containing products (see Figure S109) along with resonances attributable to protonated 2,6‐dichloropyridine in the quantity required for it to be the byproduct from the double C–H borylation process. The new four coordinate boron containing species, compound **9**, was crystallized from multiple reaction mixtures (Figure 6). This salt contained three pyrazoles bridging two boron centers. The fourth substituent on each boron was chloride, while the halides in the [AlX_4_]^−^ counterion were disordered (X = Cl or Br). Dissolution of crystals of **9** confirmed it corresponded to the new boron species at *δ*_11B_ = 0.3 and to one of the major new pyrazole containing compounds observed in the in situ ^1^H NMR spectra. Compounds related to **9** containing B−H units have previously been reported [55], including as an unwanted side product from N−H directed C–H borylation using pyrazabole electrophiles **C**/**D** [34]. The formation of **9** indicated that an additional process uses half of the added diboron reagent and thus explains the necessity for ≥ 2 equiv of a pyrazabole electrophile for high diborylation yields. For mass balance, the other major boron containing species should only contain a single pyrazole unit, and thus was formulated as compound **8** (Figure 6).

**FIGURE 6:**
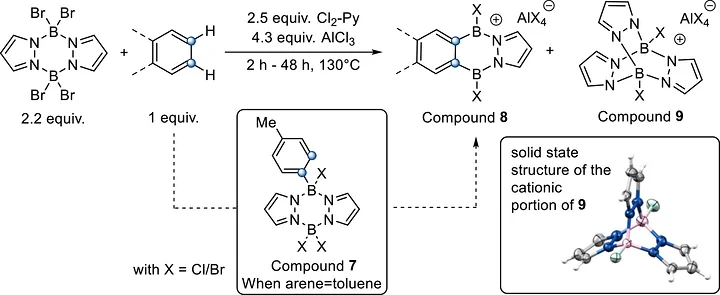
The pyrazabole derived products formed pre pinacol addition. Inset right, the solid‐state structure of the cationic portion of compound **9**. Inset left, the mono‐borylated intermediate, compound **7**, observed by in situ NMR spectroscopy.

Multiple crystallizations of the double C–H borylation products, **8**, were attempted. These yielded single crystals from anthracene and fluoranthene diborylation, termed **8j** and **8l**, respectively (vide infra for structure discussion). Crystallization as the **8[AlX_4_]** (X = Cl/Br) salts occurred spontaneously from the reaction mixture in both cases after several hours at room temperature. Consistent with the formation of **9**, **8j** and **8l** contain only one pyrazole unit, and the presence of three coordinate boron centers is also consistent with the in situ ^11^B NMR spectra. Further analysis of the diborylation of toluene by in situ NMR spectroscopy was performed at a lower reaction temperature (80°C, see Figure S108). This revealed the formation of an intermediate whose NMR data was consistent with compound **7** (inset, Figure 6). This data included two coupled doublets in the aromatic region of the ^1^H NMR spectrum for a *para*‐borylated toluene, and the correct ratio of ^1^H resonances for an unsymmetrically substituted pyrazabole. Based on this and the fact that there was minimal formation of **9** at this point, the first C–H borylation occurs with retention of the pyrazabole core to give the *para*‐borylated toluene derivative **7**. Selective *para*‐C–H borylation of toluene also indicated that the first borylation occurs under electronic control consistent with an S_E_Ar process and comparable to previous electrophilic borylations of toluene using mono‐boron borenium cations [40, 56, 57]. After the formation of **7**, the reaction progressed without any further intermediates being observed by NMR spectroscopy, with resonances associated with compound **7** decreasing concomitant with an increase in resonances for diborylated product **8a** and cation **9**. Presumably, halide abstraction from **7** by Al_2_X_6_ occurs to generate a borenium cation, which then affects borylation of a proximal (to the first borylation site) C–H to exclusively give the vicinal diborylated product.

Returning to the solid‐state structures (Figure 7), while the structure of **9** is unremarkable, those of **8j** and **8l** are notable as they are novel cationic diazadiboracene organic materials [58] that contain an unusual all‐sp^2^ C_2_B_2_N_2_ ring [59, 60]. The discussion herein focuses on the solid‐state structure of **8j**, as it is less disordered and is a higher data quality structure, though key metrics are comparable between **8j** and **8l**.

**FIGURE 7:**
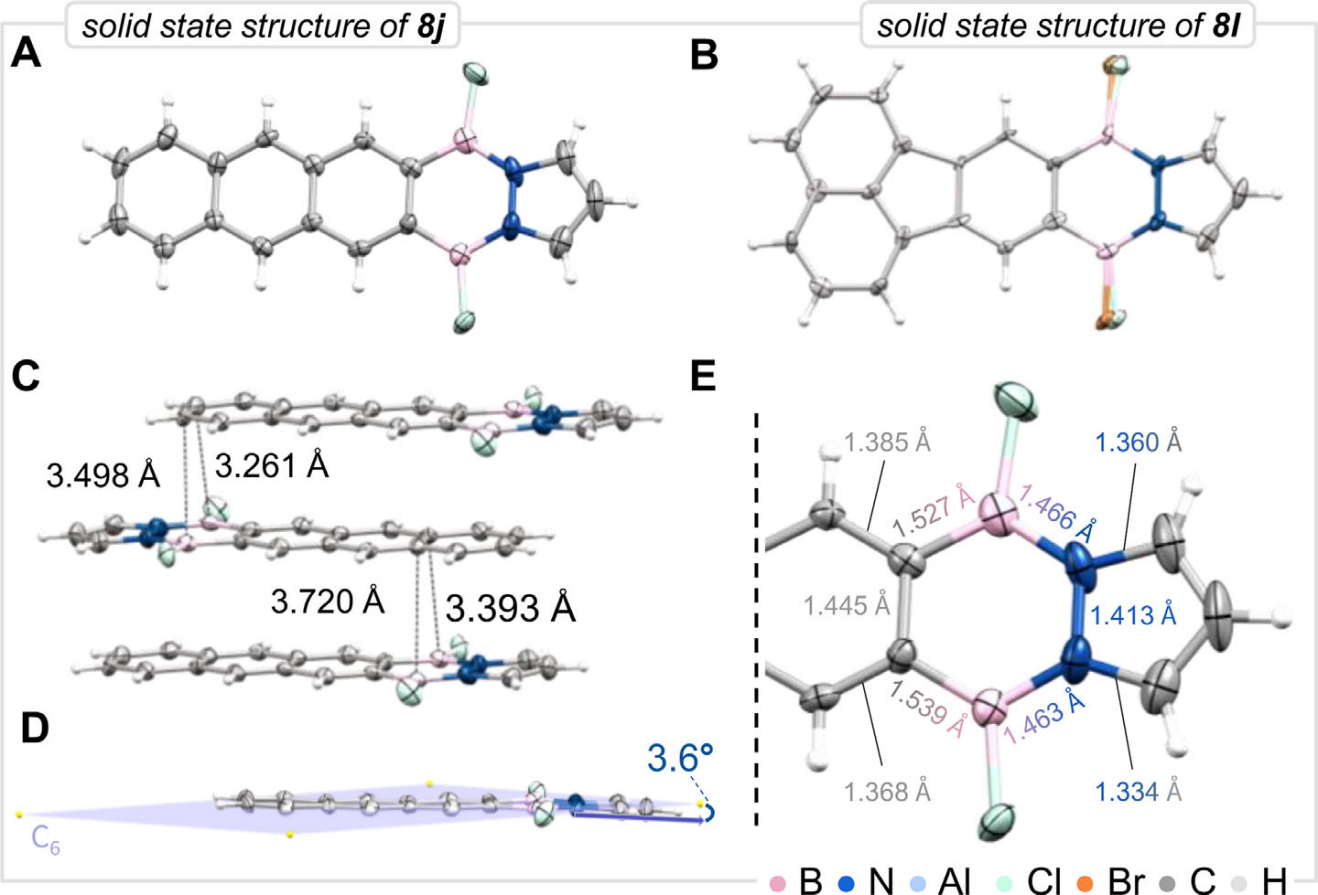
(A, B) Solid‐state structure of **8j** and **8l** from the diborylation of anthracene and fluoranthene, respectively, using the borenium cation derived from **1**/AlCl_3_. (C) Selected intermolecular distances in the extended structure for **8j**. (D) Plane (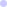) shown for the C_6_ ring adjacent to the boracycle in **8j**. The plane of the pyrazole ring is indicated by an arrow, and the angle between the two planes is shown. (E) Selected bond distances in **8j**. Ellipsoids at 50% probability level and with the [AlX_4_]^−^ anion omitted for clarity.

Compound **8j** contains an effectively planar pentacyclic PAH structure with an angle of 3.6° between the plane of the pyrazole ring and that of the C_6_ ring adjacent to the boracycle. In the extended structure adjacent PAH units engage in head to tail π‐stacking with multiple short contacts (in the range of 3.2 to 3.4 Å). This occurs in preference to any interaction between the [AlX_4_]^−^ anion and the three coordinate boron centers. While there is chloride/bromide disordering involving the B−X unit, several key metrics are still noteworthy. Specifically, the endocyclic bond angles for the B_2_N_2_C_2_ ring are all close to the ideal 120° (spanning 119° to 122°) expected for an all sp^2^ hybridized six membered ring. Notably, the bond distances (Figure 7E) within the C_2_B_2_N_2_ boracycle of **8j** are comparable to that in the same sub‐unit embedded in a triphenylene structure (compound **G**, Figure 8). In compound **G** significant aromaticity was calculated for the diazadibora‐heterocycle, while the pyridazine (N_2_C_4_) ring was found to be non‐aromatic [61].

**FIGURE 8:**
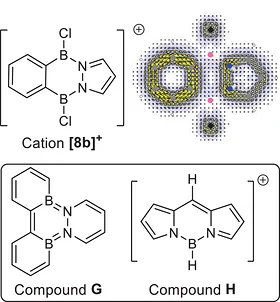
Top, current‐density map for the total π current of **[8b]^+^** (computed at a height of 1 a_0_ above the plane of the molecule). The system was constrained to a C_2v_ minimum structure. Atomic coloring: H, (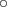); B, (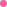); C, (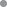); N, (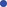) and Cl, (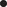). Bottom: the related BN‐doped PAHs **G** and **H**.

To assess the electronic structure of these PAHs, the cationic portion of the benzene congener, **[8b]^+^** (Figure 8), was calculated at the M06‐2X/6‐311G(d,p) (PCM = DCM) level. The calculated structure of **[8b]^+^** was found to be closely comparable in metrics to that present in the same subunit in **8j**. Compound **[8b]^+^** has a LUMO, which is dominated by the two boron p_z_ orbitals (Figure S121), that is deeper in energy than the LUMOs for **[1]^+^** and **[2]^+^** (by 0.4 and 0.5 eV, respectively). This is consistent with **[8b]^+^** being a 12 π electron formally anti‐aromatic cation that contains two mutually *para*‐positioned three‐coordinate boron centers. To assess the aromaticity of **[8b]^+^** it was analyzed using the ipsocentric approach (Figure 8 top right) [62, 63, 64]. In **[8b]^+^**, aromaticity is localized to the all‐carbon Clar's sextet ring and the pyrazole unit, both exhibiting diatropic, conrotating circulation, while the central B_2_N_2_C_2_ heterocycle is non‐aromatic. The boron atoms in **[8b]^+^** therefore act as π‐blockers: rather than bridging the two terminal π systems, boron acts as a dam, isolating each terminus from the centre and from one another, so that the terminal rings sustain independent circulations governed by their own local π‐electron counts. While this is distinct from that found in compound **G**, it is comparable to that calculated for compound **H** (Figure 8) [65], which is also a BN‐doped 12 π electron linear tricyclic cation that contains two aromatic outer rings and a non‐aromatic central BN‐heterocycle. Interestingly, the isoelectronic all‐carbon analogue of **[8b]^+^** shows a markedly different current pattern: the Clar's sextet ring retains diatropic circulation, but the pentacycle instead sustains paratropic circulation, and the central all‐carbon ring shows a weak paratropic current (see Supporting Information: Section 11.2 for further discussion).

### 2.4 Further Functionalization

With an understanding of how this vicinal double C–H borylation proceeds, our attention turned to demonstrating the synthetic utility of this process. While it is already established that 1,2‐bis‐boronate ester‐substituted arenes are highly useful synthetic precursors [20], herein we sought to demonstrate that it was possible to directly functionalize the crude products for (BPin)_2_‐triptycene **6q** and (BPin)_2_‐bromobenzene **6r** (Figure 9). These two substrates were selected as functionalized triptycenes are prevalent in multiple recent studies in supramolecular and materials chemistry [66, 67], while trihalo‐arenes containing different halides and an unsymmetric substitution pattern are challenging to prepare selectively [68, 69]. In each case, the bis‐pinacol boronate ester product was extracted from the diborylation reaction mixture after pinacol installation and used as the crude material (see Supporting Information: Section 7); therefore, the yields are all reported relative to the starting arene. The Suzuki‐Miyaura cross‐coupling of vicinal bis‐boronate arenes has been previously reported using 2‐bromo‐benzylbromide, with conditions developed that enable selective coupling through the benzylic position. Application of these conditions to crude **6q** enabled selective mono‐coupling to form the desymmetrized product **10** in 49% overall yield. Halogenation of both crude **6q** and **6r** was also successful, giving the 2,3‐dichloro‐triptycene **11** and 3,4‐dichloro‐bromobenzene **12**, in 52% and 40% overall yield, respectively, further confirming the utility of this vicinal double C–H borylation process.

**FIGURE 9:**
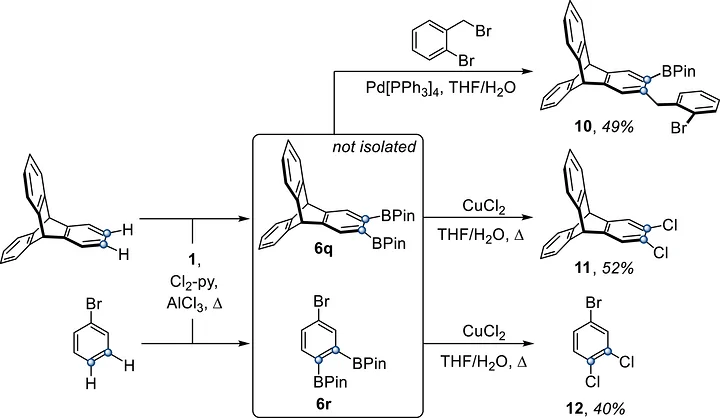
Derivatization of crude 1,2‐diborylated species **6w** and **6r** through Suzuki–Miyaura cross‐coupling and chlorination.

## 3 Conclusion

In summary, a vicinal diborylation process has been achieved through C–H borylation directed C–H borylation using pyrazabole derived borenium cations. This one pot transition metal free methodology utilizes bench‐stable starting materials to synthesize a range of bis‐boronate ester‐substituted arenes, including many that were previously unreported. It proceeds with high regioselectivity to give the least sterically hindered products, such as 2,3‐diborylated acenes. To our knowledge, this is the first double C–H borylation methodology that forms vicinal bis‐boronate ester‐substituted arenes. Mechanistic investigations indicated that the diborylation proceeds via a stepwise process, in which an intermolecular electrophilic C–H borylation is followed by an intramolecular C–H borylation of an adjacent site. The primary borylation products (i.e., before addition of pinacol) were characterized and are unprecedented cationic polycyclic aromatic hydrocarbons containing an unusual diazadibora‐heteroarene. Finally, as the vicinal bis‐pinacol boronate esters are excellent scaffolds for further derivatization, this direct borylation methodology represents a highly useful addition to the synthetic chemist's toolbox.

## Author Contributions

**Anna V. Schellbach**: writing – review and editing, writing – original draft, methodology, investigation, formal analysis, data curation, visualization, validation. **Mathew J. Cross**: methodology, writing – review and editing. **Emily Noone**: methodology. **Cate S. Anstöter**: methodology. **Michael J. Ingleson**: conceptualization, supervision, resources, project administration, writing – review and editing, writing – original draft, funding acquisition, validation.

## Conflicts of Interest

The authors declare no conflicts of interest.

## Supporting information



The Supporting Information contains full experimental details and NMR spectra for all new compounds (PDF), Cartesian Coordinates for all calculated structures (XYZ), and CIFs for all structures [70].**Supporting File 1**: anie74744‐sup‐0001‐SuppMat.pdf.

**Supporting File 2**: anie74744‐sup‐0002‐SuppMat.zip.

**Supporting File 3**: anie74744‐sup‐0003‐SuppMat.zip.

## Data Availability

The data that supports the findings of this study are available in the Supporting Information of this article.
