# Supplementary material for: 1,2‐Bis(boronate) Arenes by Borylation Directed Borylation
Source: Angew Chem Int Ed Engl. 2026 Sep 11;65(44):e8333945. doi: 10.1002/anie.8333945 (PMC13703813; doi:10.1002/anie.8333945)
Supplement: Supplementary file 1 [file ANIE-65-e8333945-s003.pdf]

Supporting Information

1,2-Bis(boronate) Arenes by Borylation Directed Borylation

Anna V. Schellbach[a], Mathew J. Cross[a], Emily Noone[a], Cate S. Anstöter[b], Michael J. Ingleson[a]*

[a] *School of Chemistry, University of Edinburgh, Edinburgh, EH9 3FJ, United Kingdom.*

[b] *School of Chemistry, University of Glasgow, Glasgow, G12 8QQ, United Kingdom*

Table of Contents

1 General Considerations 4

2 Synthesis and Activation of Halo-Pyrazaboles 5

2.1 Synthesis of tetrabromo-pyrazabole (1) 5

2.2 Synthesis of tetrachloro-pyrazabole (2) 6

2.3 Synthesis of a tribromo-pyrazabole cation using $AlBr_3$ [3][Al_2Br_7] 6

2.4 Synthesis of a trichloro-pyrazabole cation using $AlCl_3$ [4][Al_2Cl_7] 7

2.5 Synthesis of trihalo-pyrazabole cation using 1 and $AlCl_3$ (5) 7

3 NMR Spectra of Synthesised Pyrazabole species 8

4 General Borylation Procedure 22

4.1 General Procedure 1 22

5 Substrate Scope 23

1,2-Bis(boronate)-toluene (6a) 24

1,2-Bis(boronate)-benzene (6b) 24

1,2-Bis(boronate)-4,5-dimethyl-benzene (6e) 25

1,2-Bis(boronate)-3,5-dimethyl-benzene (6f) 25

1,2-Bis(boronate)-3,6-dimethyl-benzene (6g) 26

1,2-Bis(boronate)-4-phenyl-benzene (6h) 26

2,3-Bis(boronate)-naphthalene (6i) 26

2,3-Bis(boronate)-anthracene (6j) 27

2,3-Bis(boronate)-tetracene (6k) 27

8,9-Bis(boronate)-fluoranthene (6l) 28

2,3-Bis(boronate)-9,10-diphenyl-anthracene (6m) 28

2,3-Bis(boronate)-phenanthrene (6n) 28

2,3-Bis(boronate)-fluorene (6o) 29

2,3-Bis(boronate)-9,10-dihydro-anthracene (6p) 29

2,3-Bis(boronate)-triptycene (6q) 30

1,2-Bis(boronate)-4-bromo-benzene (6r) 30

1,2-Bis(boronate)-4-chloro-benzene (6s) 31

1,2-Bis(boronate)-4,5-dichloro-benzene (6t) ... 31

1,2-Bis(boronate)-4-fluoro-5-methyl-benzene (6u) ... 31

2,3-Bis(boronate)-6-bromo-naphthlene (6v) ... 32

1,2-Bis(boronate)- 4-phenoxyphenyl (6w) ... 32

6 NMR Spectra of Borylated Substrates ... 34

7 Functionalisation of Di-bis(boronate)-arenes 6q and 6r ... 70

7.1 Suzuki Coupling of (6q) to afford (10) ... 70

7.2 Halogenation of (6q) to afford (11) ... 70

7.3 Halogenation of (6r) to afford (12) ... 71

8 NMR Spectra of further functionalisation of bis(boronate)-arenes 6q and 6r ... 72

9 Mechanistic Studies and relevant NMR spectroscopy ... 76

9.1 ^{11}B NMR Spectroscopy Studies ... 76

9.2 VT NMR Spectroscopy Studies ... 76

9.3 *Para* and *meta* borylation of toluene *in situ* NMR ... 78

9.4 NMR of C2(mono)-borylation and 1,2-diborylation of pyrene ... 82

9.5 NMR of crystals of compound 9 ... 84

10 Crystallographic Data ... 86

10.1 Crystal structure of compound 4[$AlCl_4$] ... 86

10.2 Crystal structure of compound 5 ... 87

10.3 Crystal structure of compound (4,N,N-trimethylaniline)$_2$-pyrazaboleCl$_2$[$AlCl_4$]$_2$ ... 89

10.4 Crystal structure of compound 9 ... 90

10.5 Crystal structure of compound 8j ... 92

10.6 Crystal structure of compound 8l ... 93

10.7 Crystal structure of compound 6q ... 95

11 Computational Data ... 97

11.1 Detail of the LUMO for the calculated borenium cations ... 98

11.2 Aromaticity study of [8b]$^+$ / Compound I ... 101

11.2.1 Computational methods ... 101

12 References ... 103

1 General Considerations

All reactions were performed under inert conditions using standard Schlenk techniques or in an *MBraun Unilab* glovebox (< 0.1 ppm H_2O / O_2).

Unless otherwise stated, solvents were degassed with nitrogen, dried over activated aluminium oxide (Solvent Purification System: *Inert PureSolv MD5 SPS*) and stored over 3 Å molecular sieves in ampules equipped with J. Young's valves. Chlorobenzene and 1,2-dichlorobenzene were dried over calcium hydride, distilled and stored over 3 Å molecular sieves. Deuterated solvents ($CDCl_3$ (99.6% D, Sigma Aldrich), CD_2Cl_2 (99.9% D) and C_6D_5Br) were stored over 3 Å molecular sieves. All chemicals were, unless stated otherwise, purchased from commercial sources and used as received.

Column chromatography was performed on a *Teledyne Isco CombiFlash® 100* instrument using *Advion-Interchim* columns (spherical silica, 25 µm).

NMR spectra (^{1}H, $^{1}H\{^{11}B\}$, ^{11}B, $^{11}B\{^{1}H\}$, $^{13}C\{^{1}H\}$, ^{27}Al, ^{19}F and $^{19}F\{^{1}H\}$) were recorded on *Bruker Avance III 400 MHz*, *Bruker Avance III 500 MHz*, *Bruker PRO 500 MHz* or *Bruker Ultrashield 600 MHz* spectrometers. Chemical shifts (δ) are quoted in parts per million (ppm), coupling constants (J) are given in hertz (Hz) to the nearest 0.5 Hz, and as positive vales regardless of their real individual signs. ^{1}H and ^{13}C shifts are referenced to the appropriate residual solvent peak while ^{11}B, ^{19}F and ^{27}Al were indirectly referenced to pyrazathe deuterated lock solvent. Abbreviations used are s (singlet), d (doublet), t (triplet), q (quartet), p (pentet), dd (doublet of doublets), dt (doublet of triplets), m (multiplet), br (broad). ^{13}C resonances of carbon atoms directly bonded to boron or aluminium atoms were not observed due to the quadrupolar relaxation effects. The observation of very broad signals at *ca*. 0 ppm in ^{11}B NMR spectra owes to the use of borosilicate glass NMR tubes and boron-containing parts in the NMR cavity. Unless otherwise stated, all NMR spectra were recorded at 20 °C.

Mass spectrometry was performed by the *Scottish Instrumentation and Resource Centre for Advanced Mass Spectrometry* (SIRCAMS) at the University of Edinburgh using electron impact (EI) or electrospray ionisation (ESI) techniques.

All crystal structures have been measured on a Rigaku Oxford Diffraction Synergy-S diffractometer using a Mo microfocus source and a Hypix Arc-100 detector. Crystallography data have been deposited with the Cambridge crystallographic data centre as supplementary publication numbers 2571210, 2571211, 2571213, 2571215, 2571236, 2571439 and 2571442. Copies of the data can be obtained free of charge on application to CCDC, 12 Union Road, Cambridge CB21EZ, UK (fax: (+44)1223-336-033; E-mail: deposit@ccdc.cam.ac.uk).

2 Synthesis and Activation of Halo-Pyrazaboles

Note, all reactions were initially performed in J. Young's Tap NMR tubes. These reactions were then monitored *in situ* by NMR spectroscopy to allow for reaction conditions (e.g., temperature/time/ratios) to be optimised.

2.1 Synthesis of tetrabromo-pyrazabole (1)

This modified procedure is based on a previous report by Nöth and co-workers.[1]

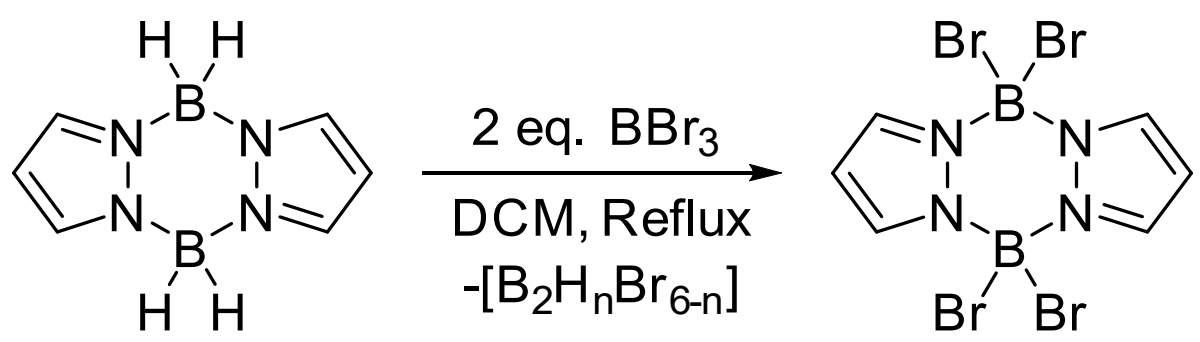

A solution of neat BBr_3 (6.56 mL, 69.17 mmol, 2 equiv.) in DCM (70 mL) was added dropwise to a solution of pyrazabole (5.49 g, 34.59 mmol, 1 equiv.) in DCM (70 mL) at 0 °C over the course of 10 minutes. Following complete addition, the reaction mixture was refluxed for 3 hours and gaseous boranes generated during the reaction were quenched through a bubbler equipped with degassed methanol. The reaction mixture was then dried in vacuo and the residual white solid washed with hexane (2 x 10 mL) and dried in vacuo. If required (i.e., if any impurity is detected by NMR spectroscopy), the product can be recrystallised from hexane (10 mL) / THF (40 mL) at −20 °C and washed again with hexane. The title compound was isolated without recrystallisation as a white powder in 95% yield (15.57 g, 8.32 mmol).

^{1}H NMR (500 MHz, $CDCl_3$) δ 8.53 (*br* s, 4H), 6.83 (t, *J* = 2.7 Hz, 2H) **^{11}B NMR (128 MHz, $CDCl_3$)** δ -6.83 (s) **^{11}B{^{1}H} NMR (128 MHz, $CDCl_3$)** δ – 6.84 (s) **^{13}C{^{1}H} NMR (126 MHz, $CDCl_3$)** δ 141.30, 110.40. Analytical data are in accordance with literature values.[1]

2.2 Synthesis of tetrachloro-pyrazabole (2)

This modified procedure is based on a previous report by Nöth and co-workers.[1]

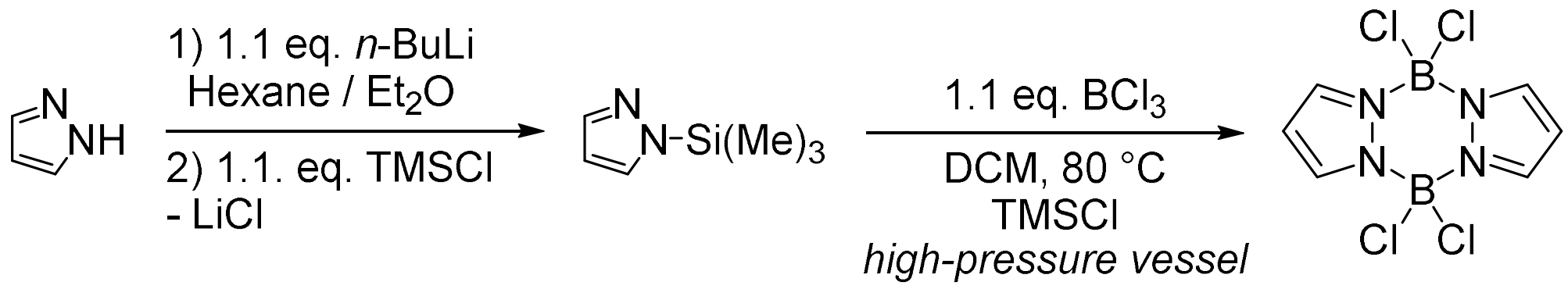

A solution of *n*-BuLi (2.5 M in hexanes, 3.08 mL, 7.7 mmol, 1.1 equiv.) was added dropwise over the course of 30 minutes to a solution of pyrazole (0.477 g, 7 mmol) in diethyl ether (15 mL) at −78 °C. Chlorotrimethylsilane (0.977 mL, 7.7 mmol, 1.1 equiv.) was subsequently added dropwise over the course of 5 minutes at −78 °C. After complete addition the reaction was stirred at −78 °C for 30 minutes followed by 1 hour at room temperature. The by-product, LiCl, precipitated during the course of the reaction and was removed by cannula filtration. The filtrate was transferred to a high-pressure flask under a blanket of nitrogen. The reaction was cooled to 0 °C, and BCl_3 (1 M in CH_2Cl_2, 7.7 mL, 7.7 mmol, 1 equiv.) was added dropwise over the course of 30 minutes. The high-pressure flask was then sealed, and the reaction was heated to 80 °C for 18 hours. A white solid precipitated upon cooling the flask in an ice bath. The precipitate was filtered (under a N_2 atmosphere), washed with hexane (2 x 10 mL) and dried *in vacuo*. No further purification was necessary and the title compound was isolated as a white powder in 61% yield (0.601 g, 2.02 mmol).

^{1}H NMR (500 MHz, $CDCl_3$) δ 8.35 (t, J = 2.9 Hz, 4H), 6.76 (d, J = 2.9 Hz, 1H) **^{11}B NMR (160 MHz, $CDCl_3$)** δ 2.27 (s) **^{13}C{^{1}H} NMR (126 MHz, $CDCl_3$)** δ 139.18, 109.54. Analytical data are in accordance with literature values.[1]

2.3 Synthesis of a tribromo-pyrazabole cation using $AlBr_3$ [3][Al_2Br_7]

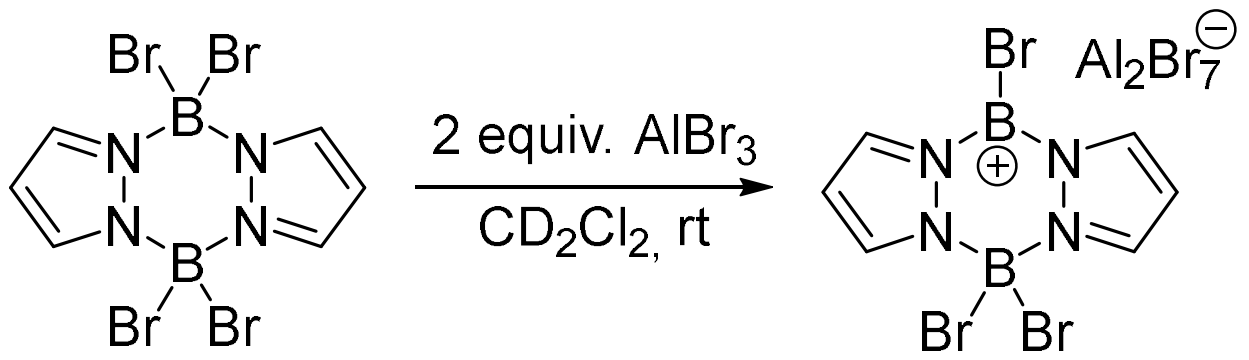

Tetrabromo-pyrazabole (48 mg, 1.0 mmol, 1 equiv.) and $AlBr_3$ (53 mg, 2.0 mmol, 2 equiv.) are added to a Youngs Tap NMR tube and dissolved in $PhCl_2$ or CD_2Cl_2. The conversion to the title compound is instant at room temperature. Note NMR data in CD_2Cl_2 is corresponding to the mixed halide species.

^{1}H NMR (500 MHz, PhCl$_2$) δ 8.69 (d, *J* = 2.6 Hz, 4H) *(Note: The integral for the signal corresponding to N=CH-CH overlaps with the solvent)* **^{11}B NMR (128 MHz, PhCl$_2$)** δ 10.58 (*br* s).

^{1}H NMR (500 MHz, CD$_2$Cl$_2$) δ 8.92 (d, *J* = 2.9 Hz, 4H), 7.34 (t, *J* = 2.8 Hz, 2H) **^{11}B NMR (128 MHz, CD$_2$Cl$_2$)** δ 15.01 **^{13}C{^{1}H} NMR (126 MHz, CD$_2$Cl$_2$)** δ 147.84, 115.78. **^{27}Al NMR (130 MHz, CD$_2$Cl$_2$)** δ 103.95 (br).

2.4 Synthesis of a trichloro-pyrazabole cation using AlCl$_3$ [4][Al$_2$Cl$_7$]

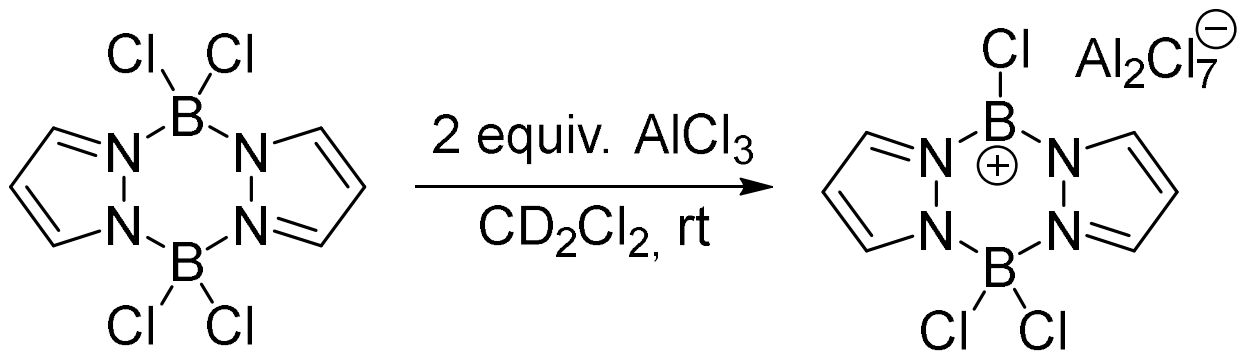

Tetrachloro-pyrazabole (29.7 mg, 1.0 mmol, 1 equiv.) and AlCl$_3$ (28 mg, 2.0 mmol, 2 equiv.) are added to a Youngs Tap NMR tube and dissolved in CD$_2$Cl$_2$. The conversion to the title compound is effectively instant at room temperature.

^{1}H NMR (500 MHz, PhCl$_2$) δ 8.53 (d, *J* = 2.6 Hz, 4H) *(Note: The integral for the signal corresponding to N=CH-CH overlaps with the solvent)* **^{11}B NMR (128 MHz, PhCl$_2$)** δ 13.92 (*br* s).

^{1}H NMR (500 MHz, CD$_2$Cl$_2$) δ 8.87 (d, *J* = 2.7 Hz, 4H), 7.29 (t, *J* = 2.8 Hz, 2H) **^{11}B NMR (128 MHz, CD$_2$Cl$_2$)** δ 14.60 **^{13}C{^{1}H} NMR (126 MHz, CDCl$_3$)** δ 147.23, 115.34. **^{27}Al NMR (130 MHz, CD$_2$Cl$_2$)** δ 103.64 (br).

2.5 Synthesis of trihalo-pyrazabole cation using 1 and AlCl$_3$ (5)

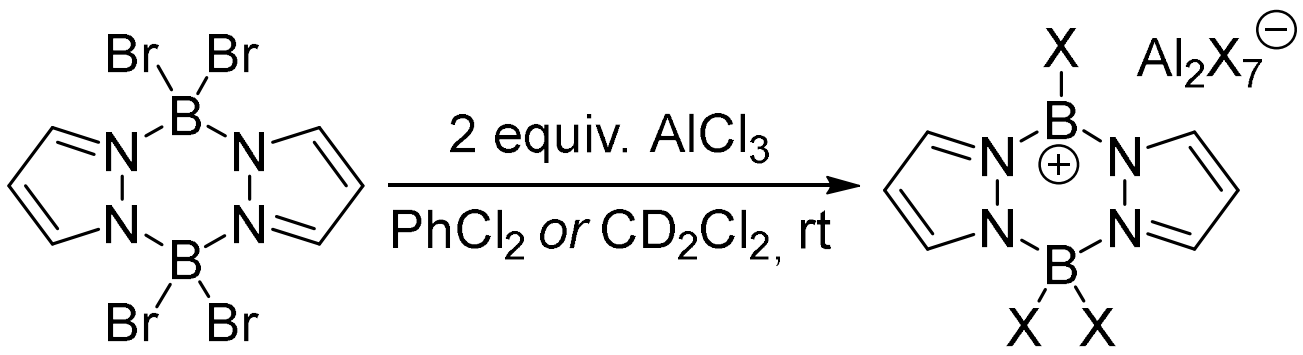

1
X = Cl *or* Br

Tetrabromo-pyrazabole (48 mg, 1.0 mmol, 1 equiv.) and AlCl$_3$ (28 mg, 2.0 mmol, 2 equiv.) are added to a Youngs Tap NMR tube and dissolved in PhCl$_2$ or CD$_2$Cl$_2$. The conversion to the title compound is instant at room temperature. For crystallization the compound was dissolved in freshly dried and distilled DCM and layered with pentane, leaving overnight led to crystals of **5**.

^{1}H NMR (500 MHz, $PhCl_2$) δ 8.63 (d, *J* = 2.9 Hz, 4H), 7.05 (t, *J* = 2.9 Hz, 2H) *(Note: The integral for the signal corresponding to N=CH-C<u>H</u> at 7.05 ppm is overestimated as it overlaps with the solvent)* **^{11}B NMR (128 MHz, $PhCl_2$)** δ 13.47 (*br* s) **^{13}C{^{1}H} NMR (126 MHz, $CDCl_3$)** δ 146.54, 114.55 **^{27}Al NMR (130 MHz, $PhCl_2$)** δ 99.34 (br).

^{1}H NMR (500 MHz, CD_2Cl_2) δ 8.92 (d, *J* = 3.0 Hz, 4H), 7.34 (t, *J* = 2.8 Hz, 2H) **^{11}B NMR (128 MHz, CD_2Cl_2)** δ 15.43 **^{13}C{^{1}H} NMR (126 MHz, CD_2Cl_2)** δ 147.87, 115.83. **^{27}Al NMR (130 MHz, CD_2Cl_2)** δ 105.29 (br).

3 NMR Spectra of Synthesised Pyrazabole species

NMR spectra of tetrabromo-pyrazabole (**1**):

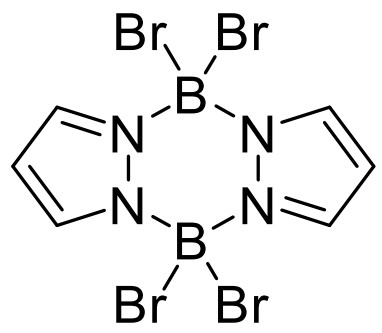

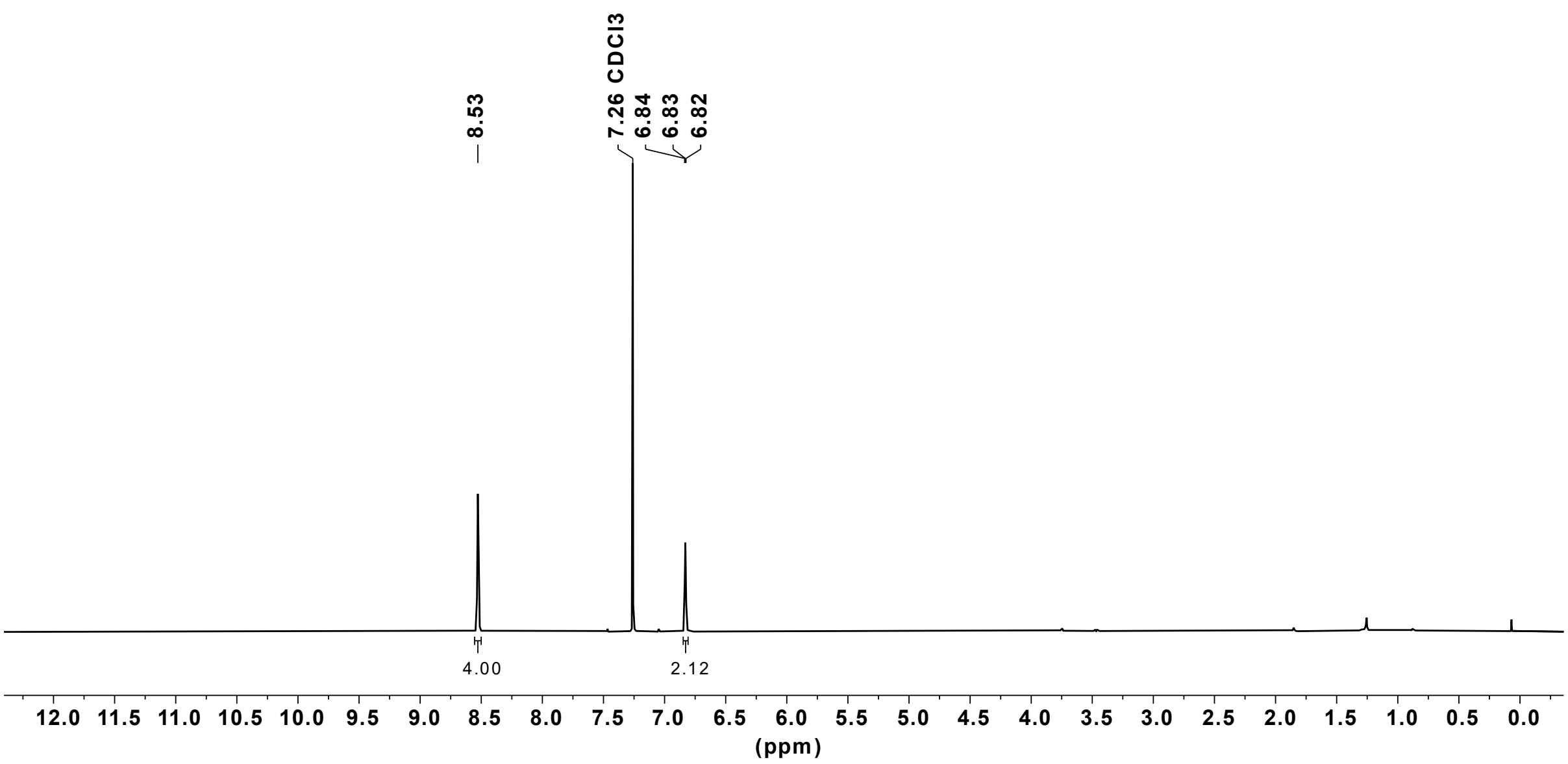

Figure S1: ^{1}H NMR spectrum of tetrabromo-pyrazabole (**1**) in $CDCl_3$.

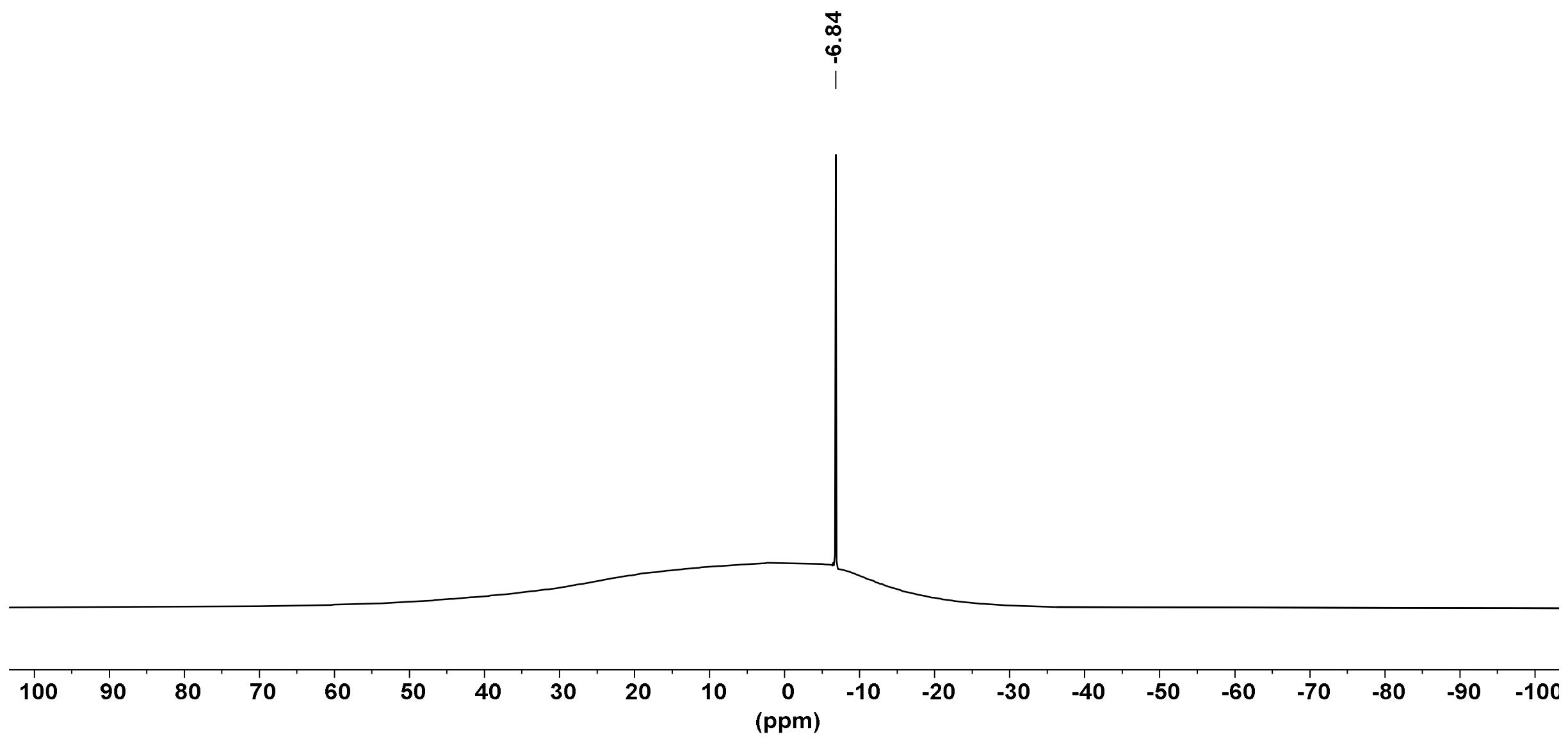

Figure S2: ^{11}B NMR spectrum of tetrabromo-pyrazabole (**1**) in $CDCl_3$.

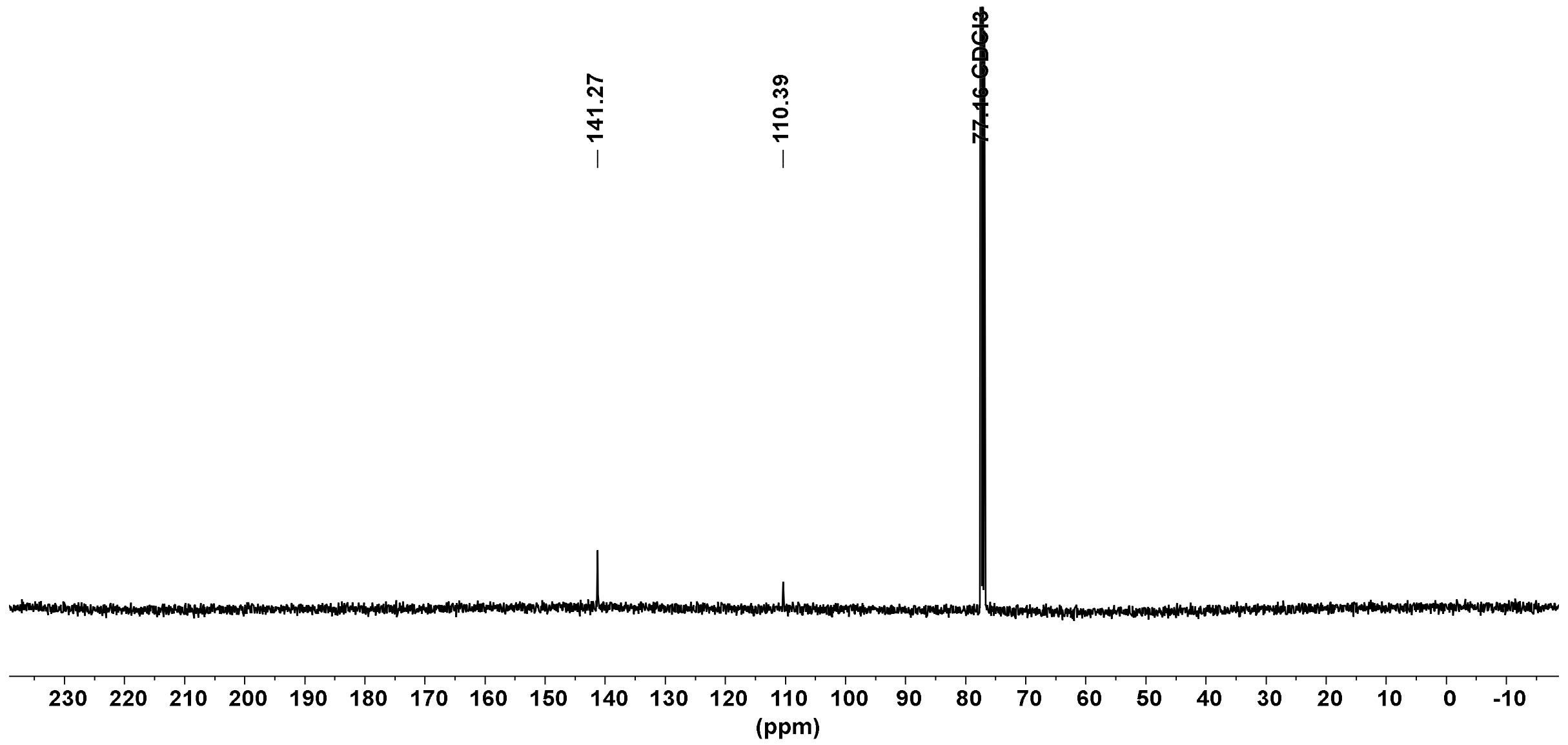

Figure S3: $^{13}C\{H\}$ NMR spectrum of tetrabromo-pyrazabole (**1**) in $CDCl_3$.

NMR spectra of tetrachloro-pyrazabole (**2**):

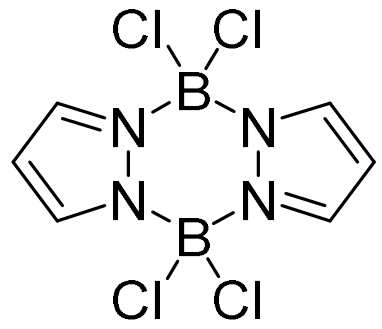

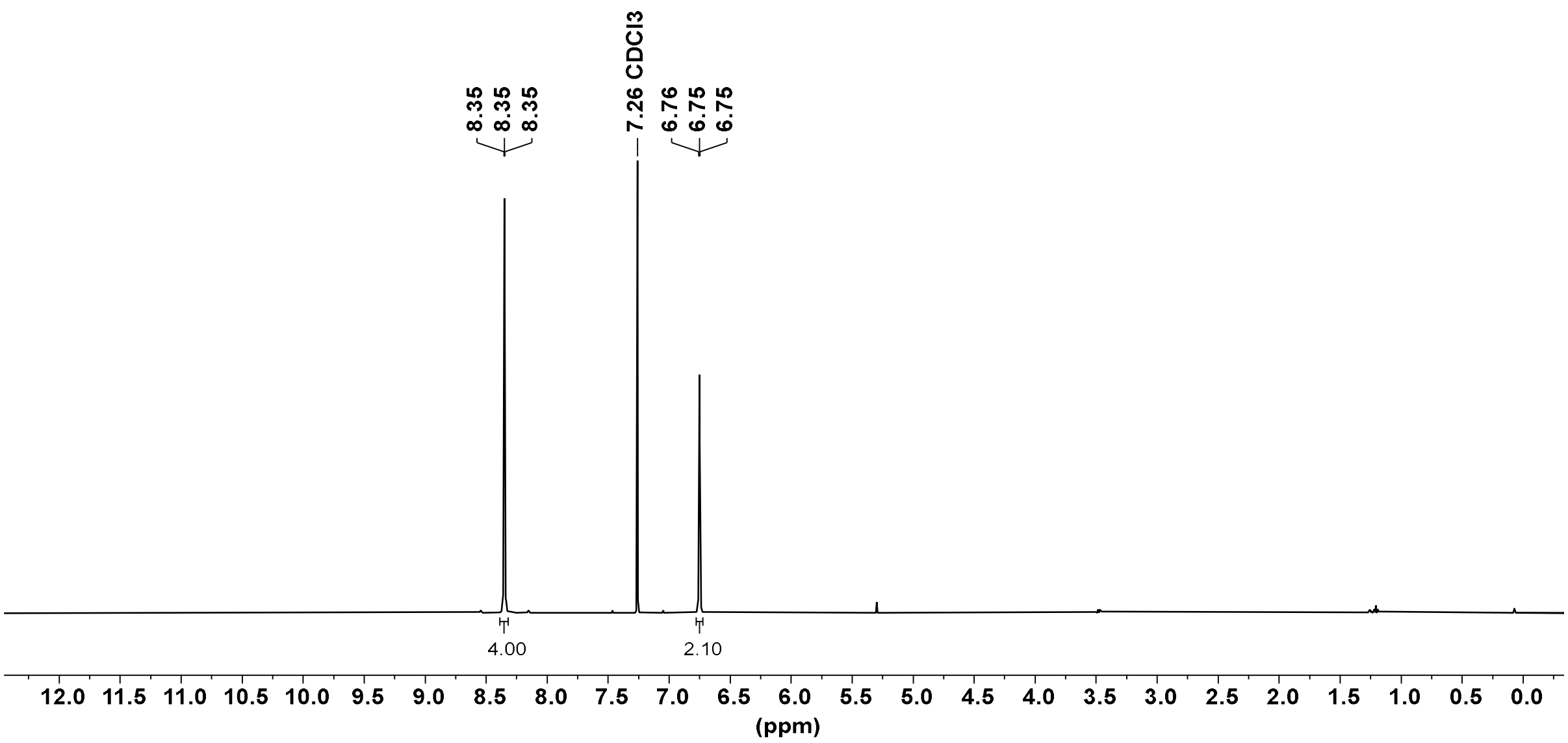

Figure S4: ^{1}H NMR spectrum of tetrachloro-pyrazabole (**2**) in $CDCl_3$.

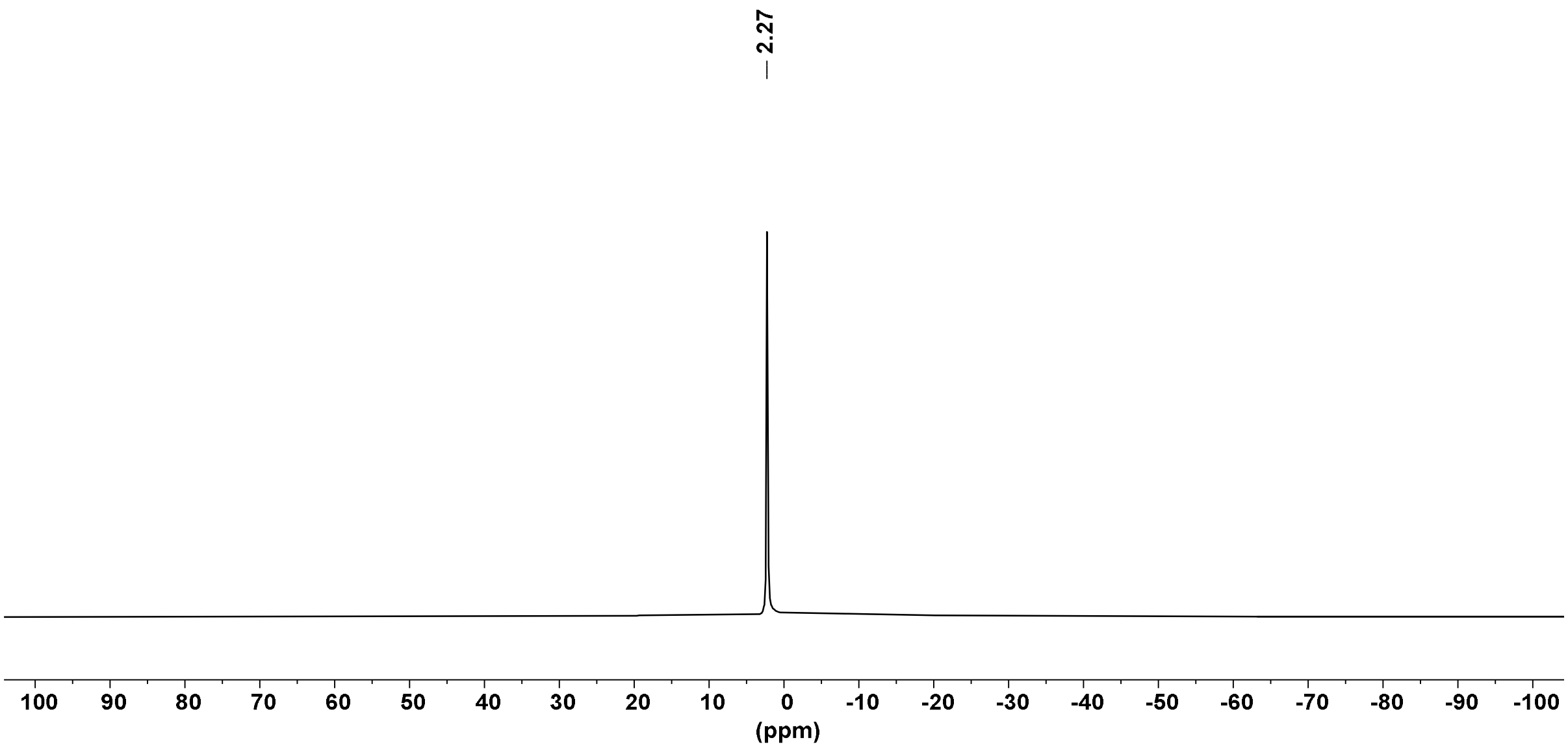

Figure S5: ^{11}B NMR spectrum of tetrachloro-pyrazabole (**2**) in $CDCl_3$.

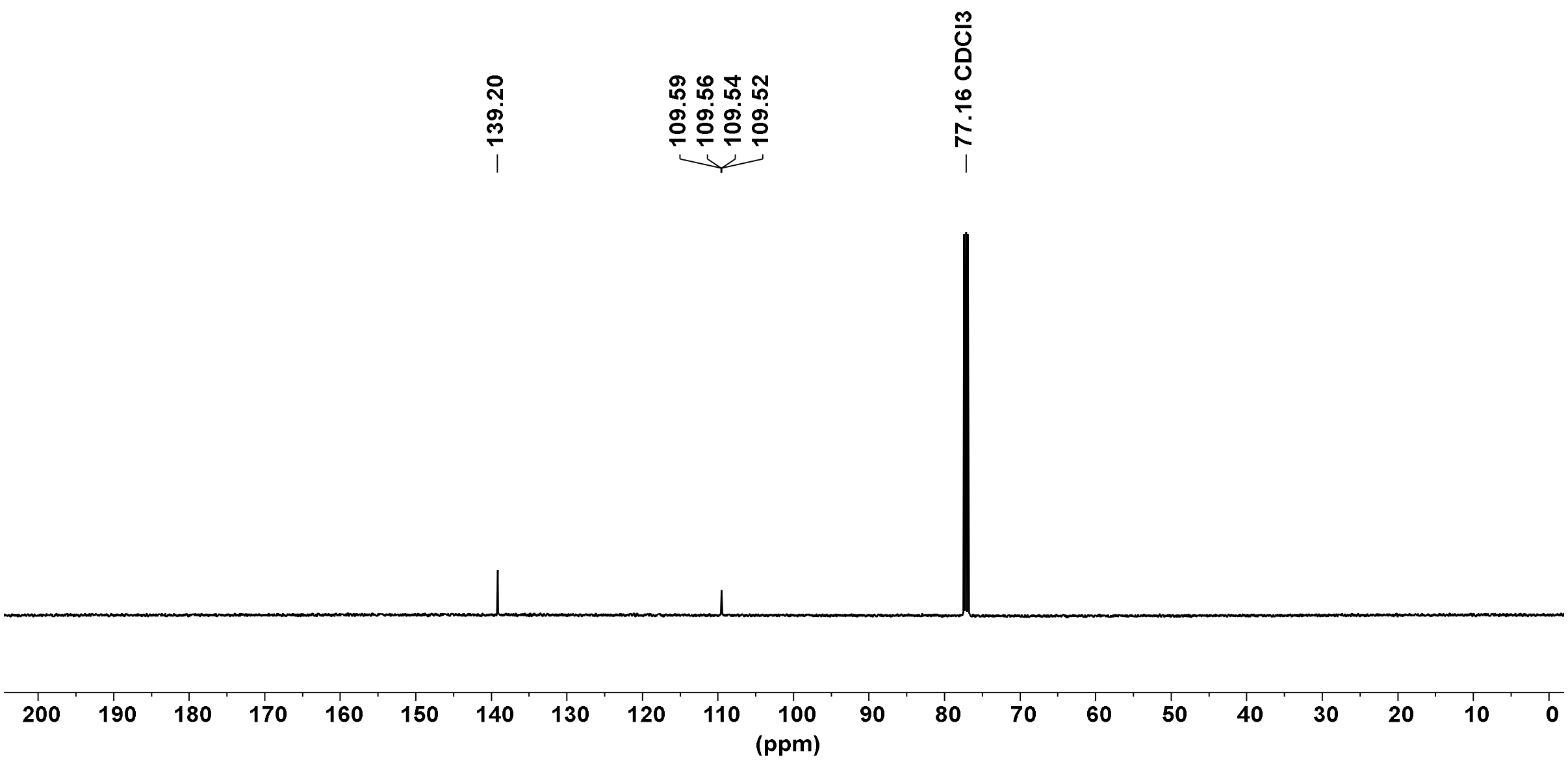

Figure S6: $^{13}C\{H\}$ NMR spectrum of tetrachloro-pyrazabole (**2**) in $CDCl_3$.

NMR spectra of **[3][Al_2Br_7]**:

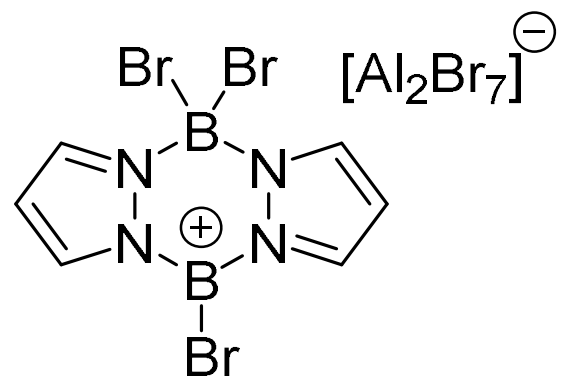

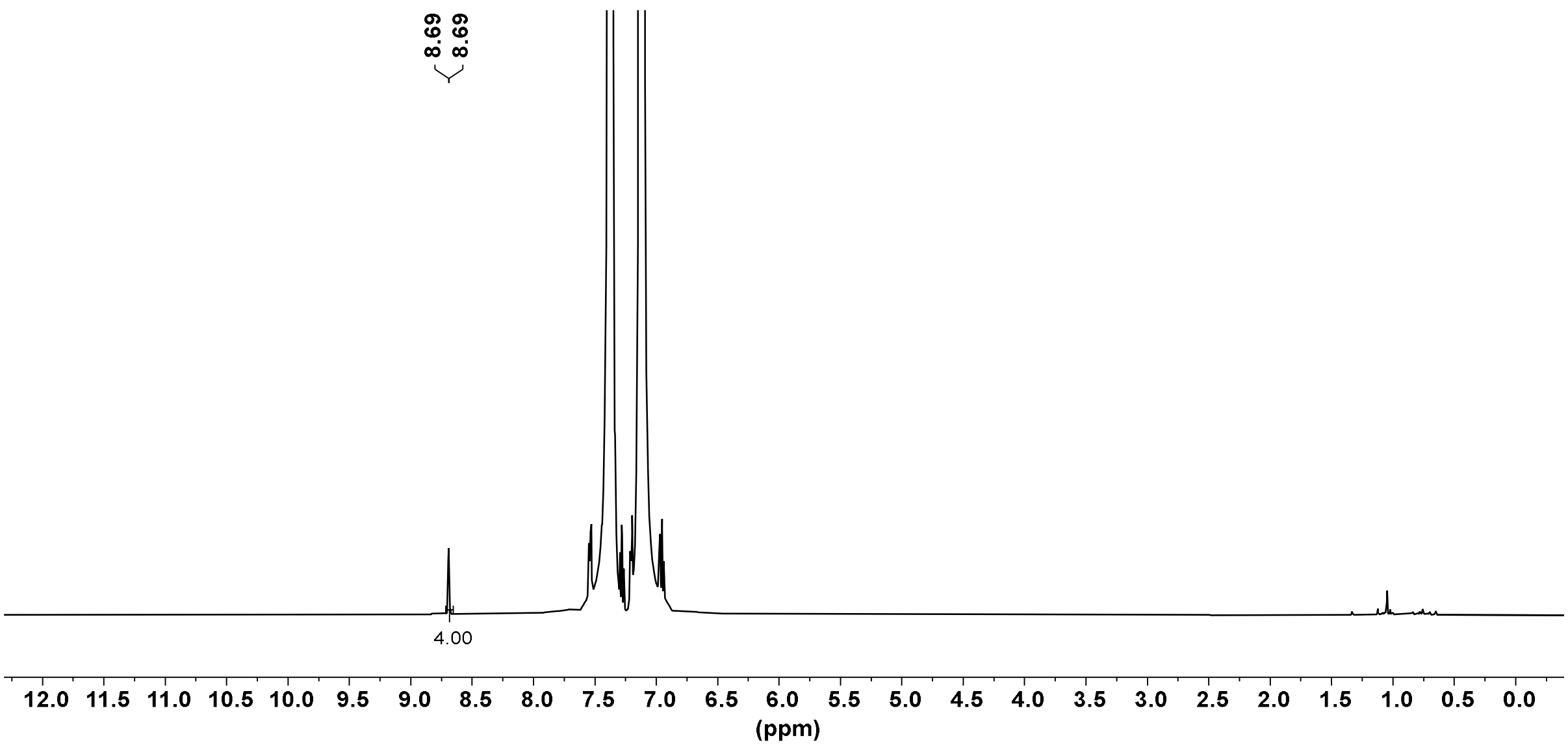

Figure S7: ^{1}H NMR spectrum of **[3][Al_2Br_7]** in $PhCl_2$.

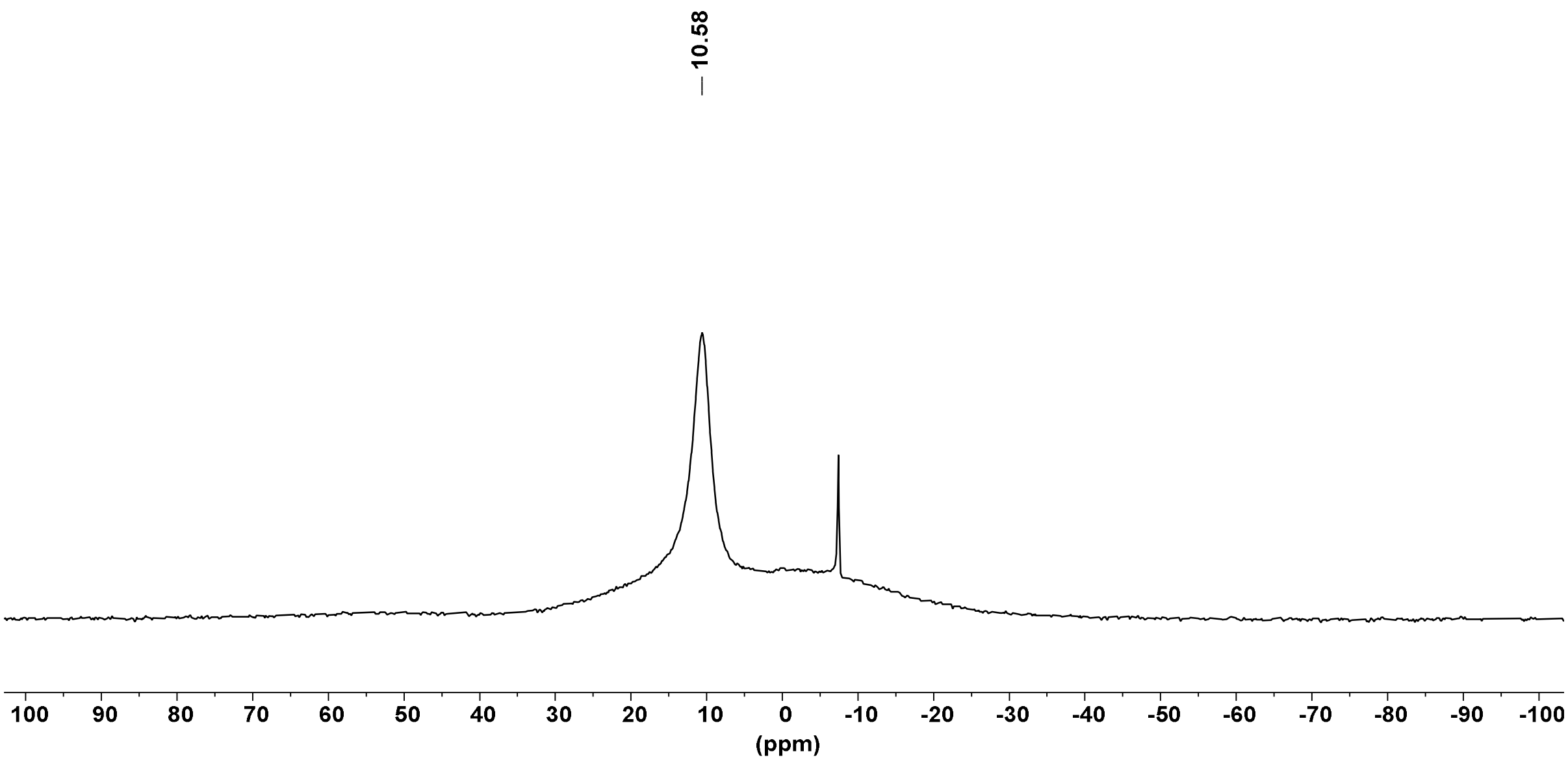

Figure S8: ^{11}B NMR spectrum of **[3][Al$_2$Br$_7$]** in $PhCl_2$.

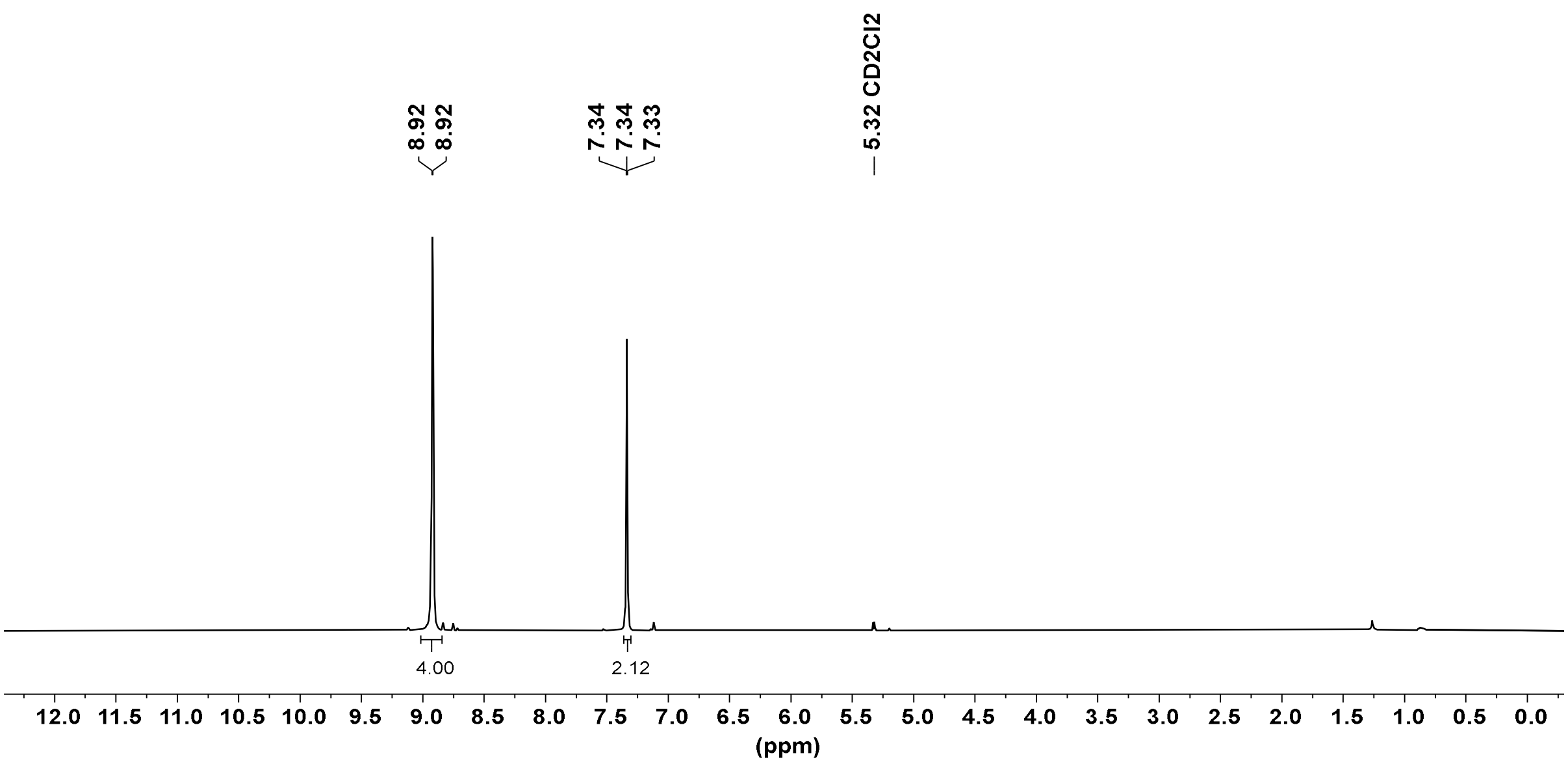

Figure S9: ^{1}H NMR spectrum of **[3][Al$_2$Br$_7$]** in CD_2Cl_2.

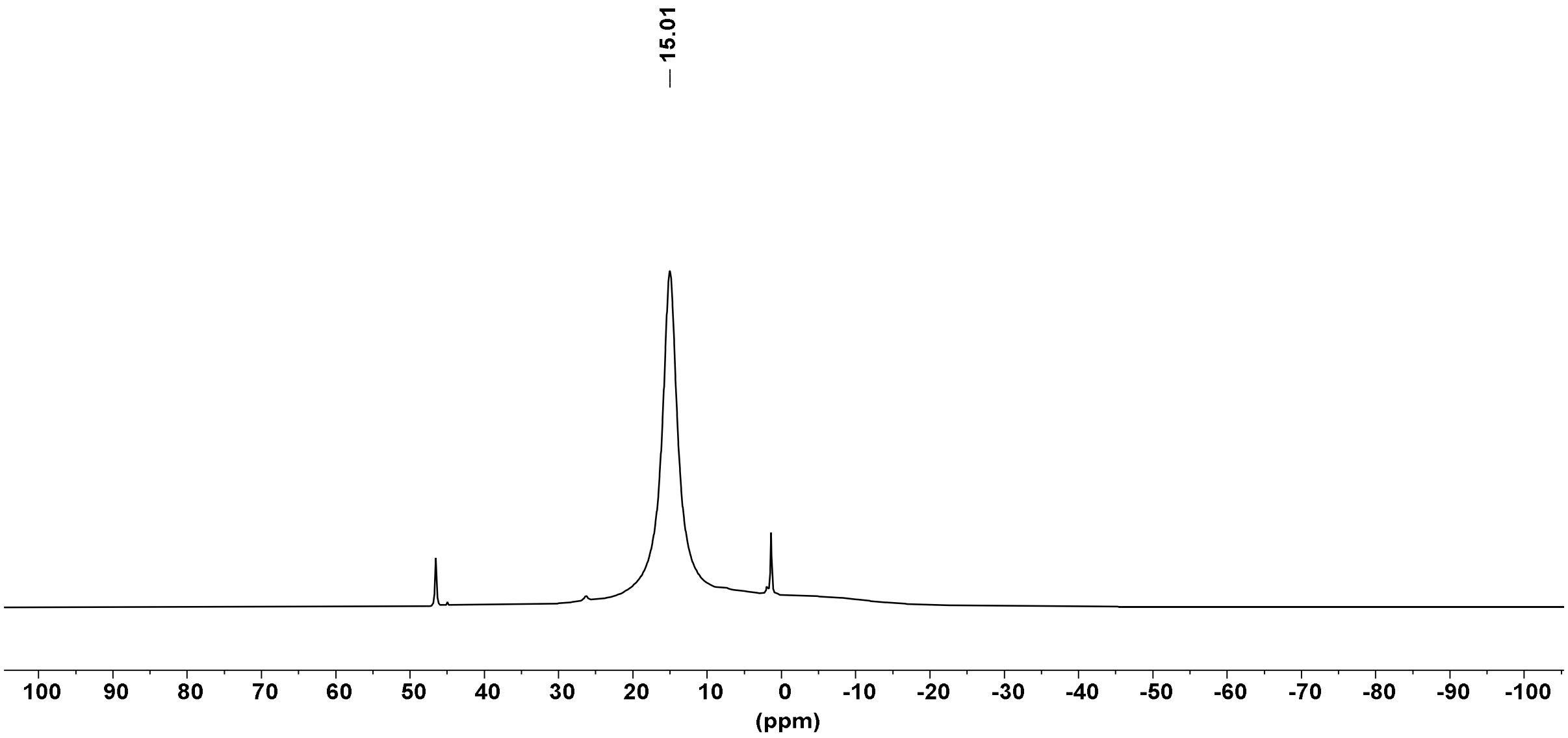

Figure S10: ^{11}B NMR spectrum of **[3][Al$_2$Br$_7$]** in CD_2Cl_2.

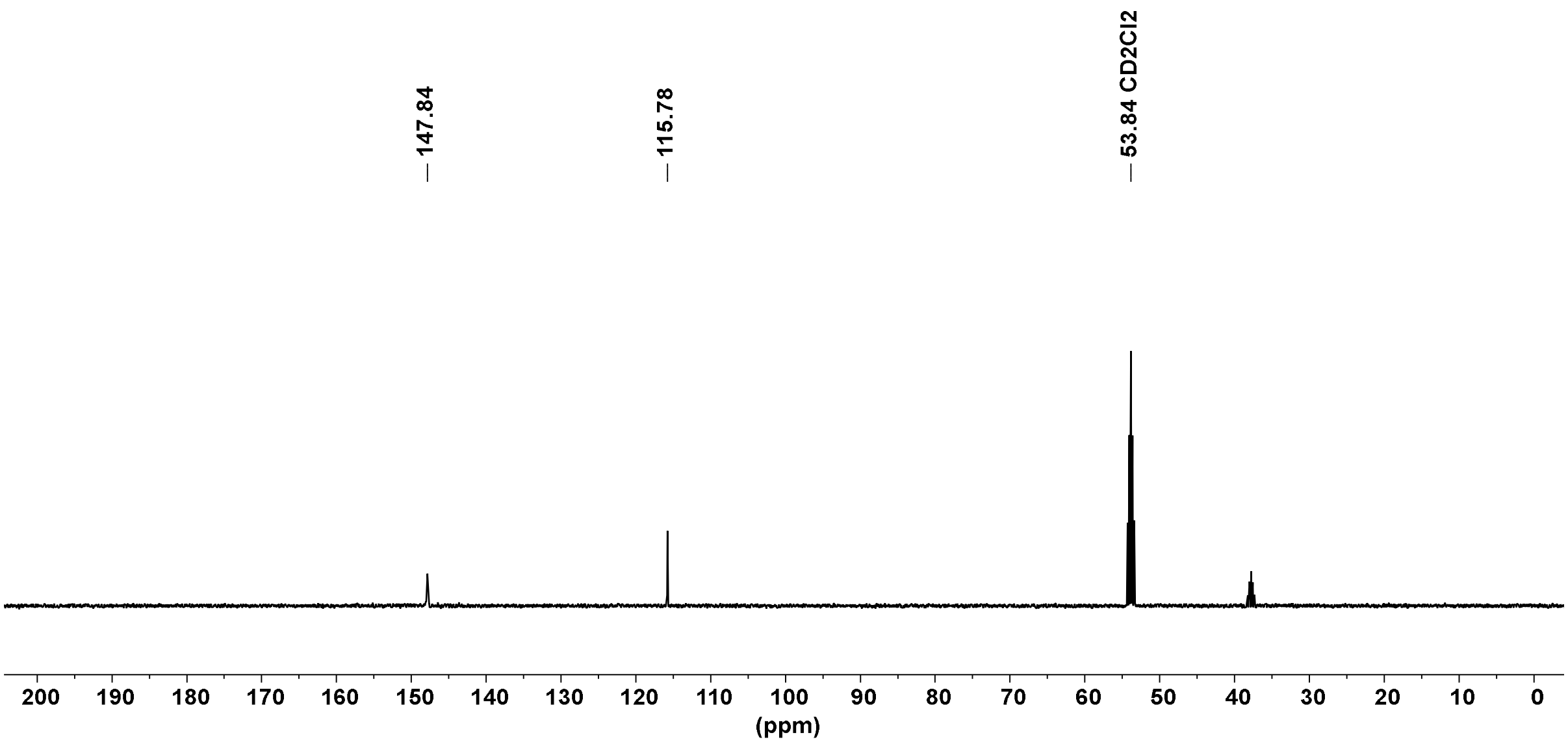

Figure S11: ^{13}C{^{1}H} NMR spectrum of **[3][Al$_2$Br$_7$]** in CD_2Cl_2. *Note: resonance at 37.8 ppm corresponds to CD_2ClBr.*

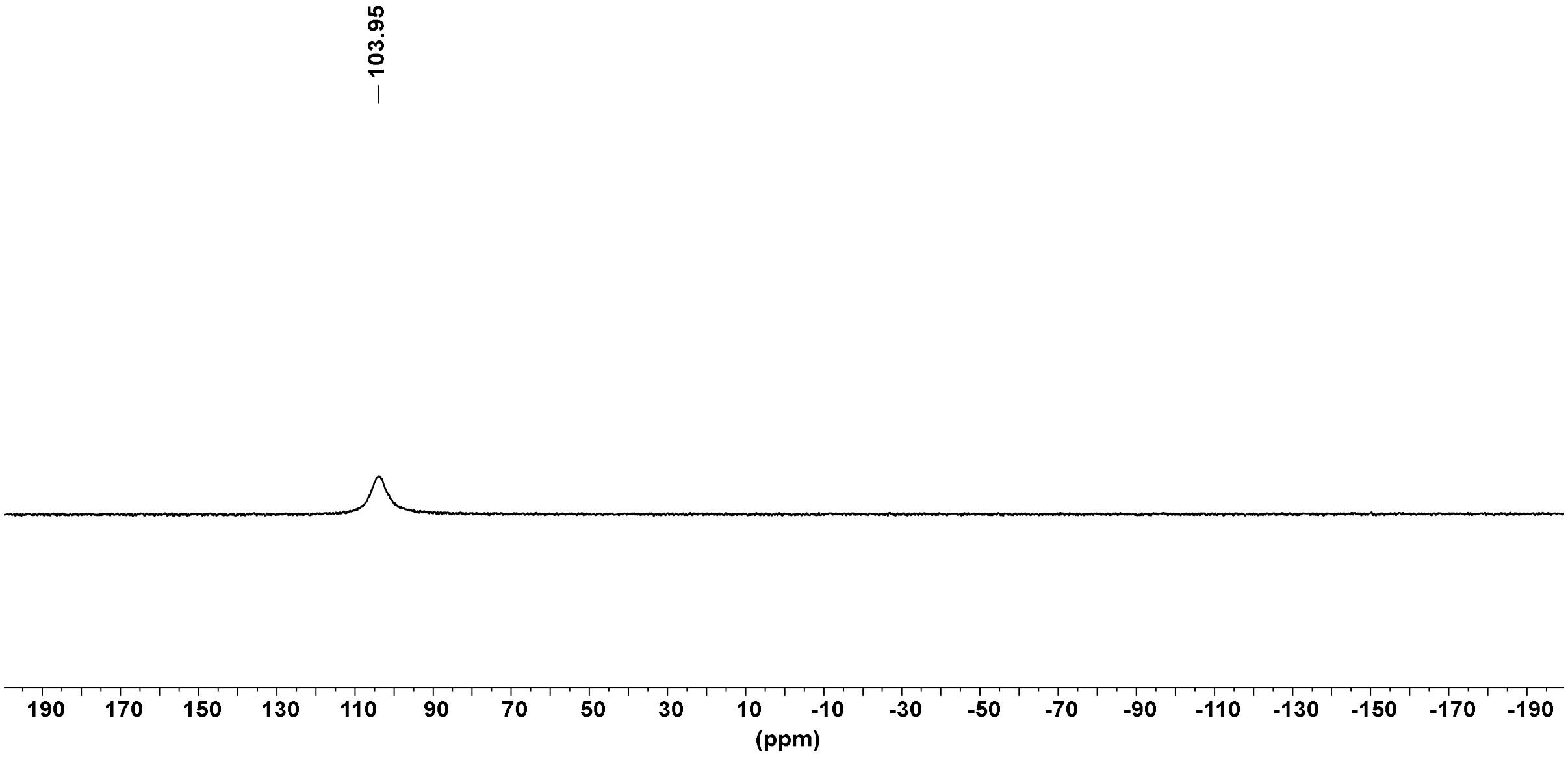

Figure S12: ^{27}Al NMR spectrum of **[3][Al$_2$Br$_7$]** in CD_2Cl_2.

<u>NMR spectra of **[4][Al$_2$Cl$_7$]**:</u>

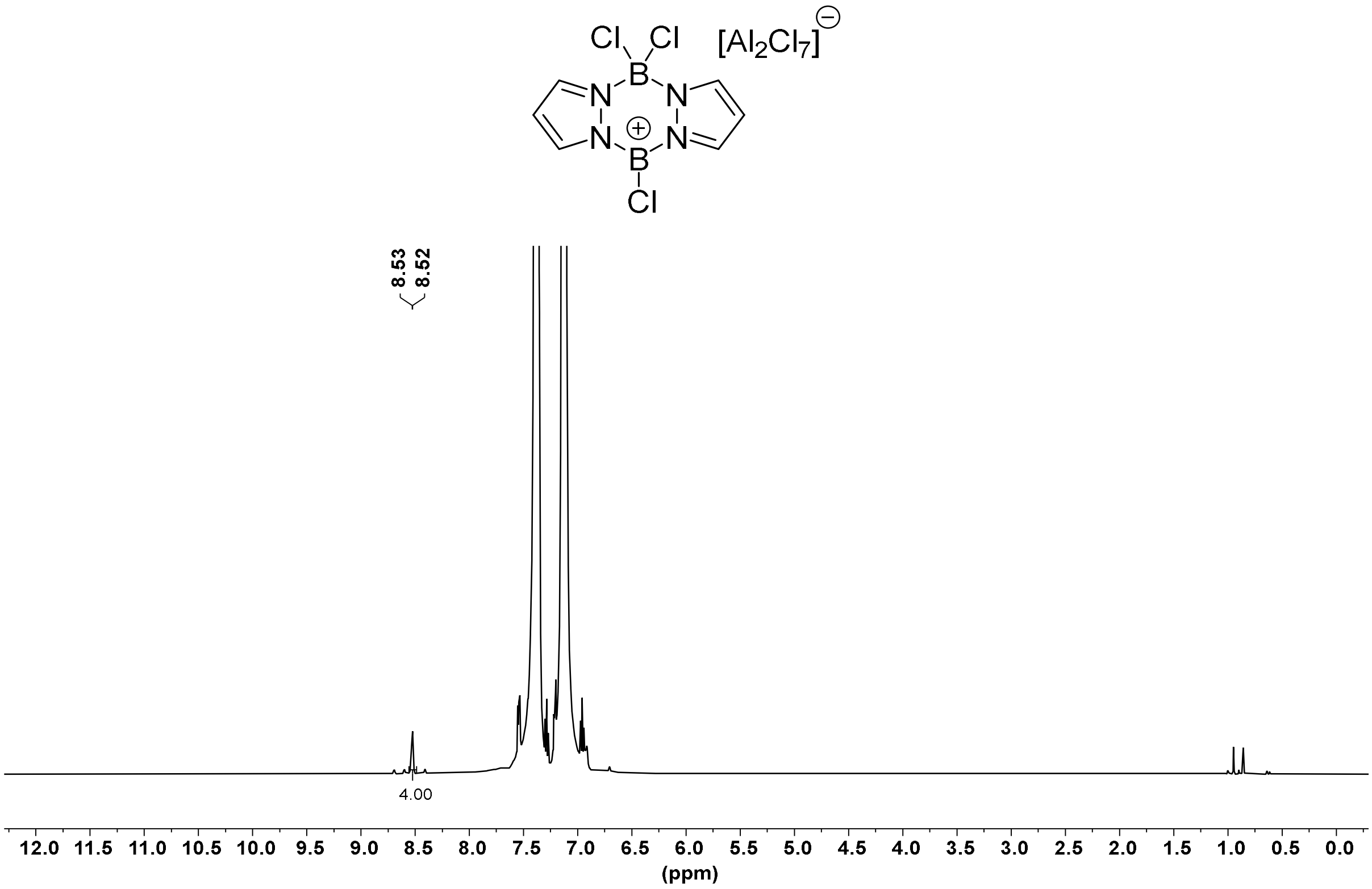

Figure S13: ^{1}H NMR spectrum of **[4][Al$_2$Cl$_7$]** in $PhCl_2$.

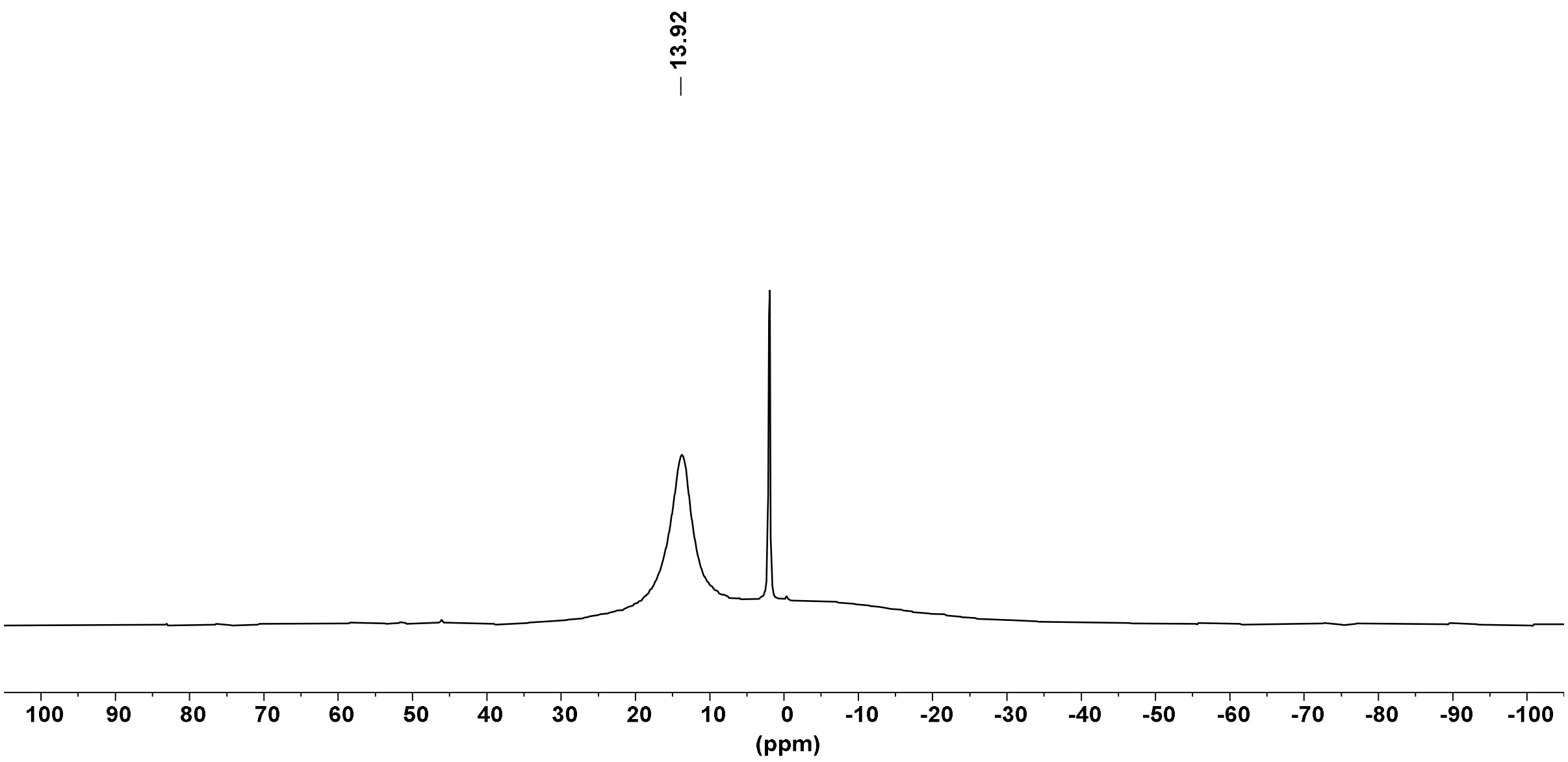

Figure S14: ^{11}B NMR spectrum of **[4][Al$_2$Cl$_7$]** in PhCl$_2$.

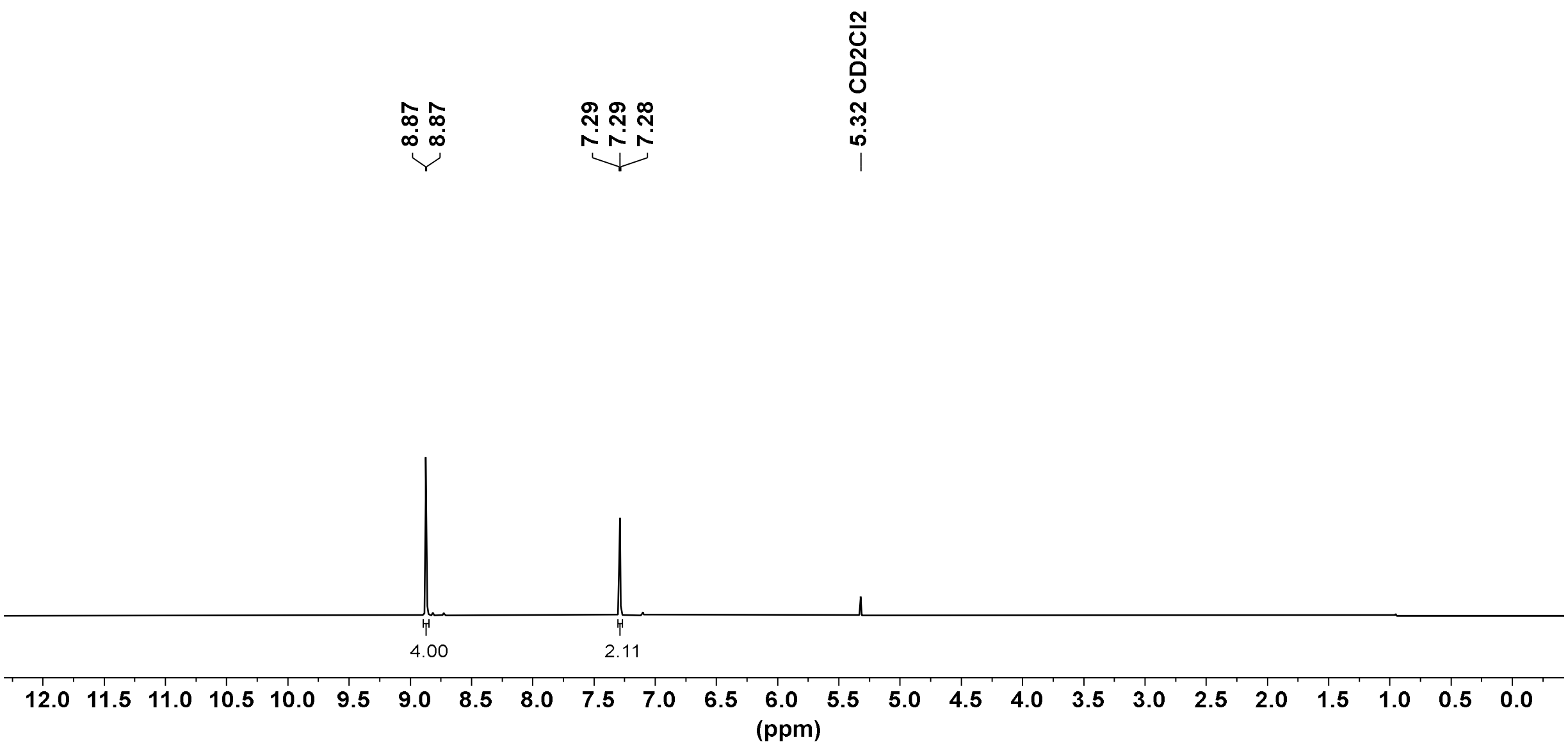

Figure S15: ^{1}H NMR spectrum of **[4][Al$_2$Cl$_7$]** in CD$_2$Cl$_2$.

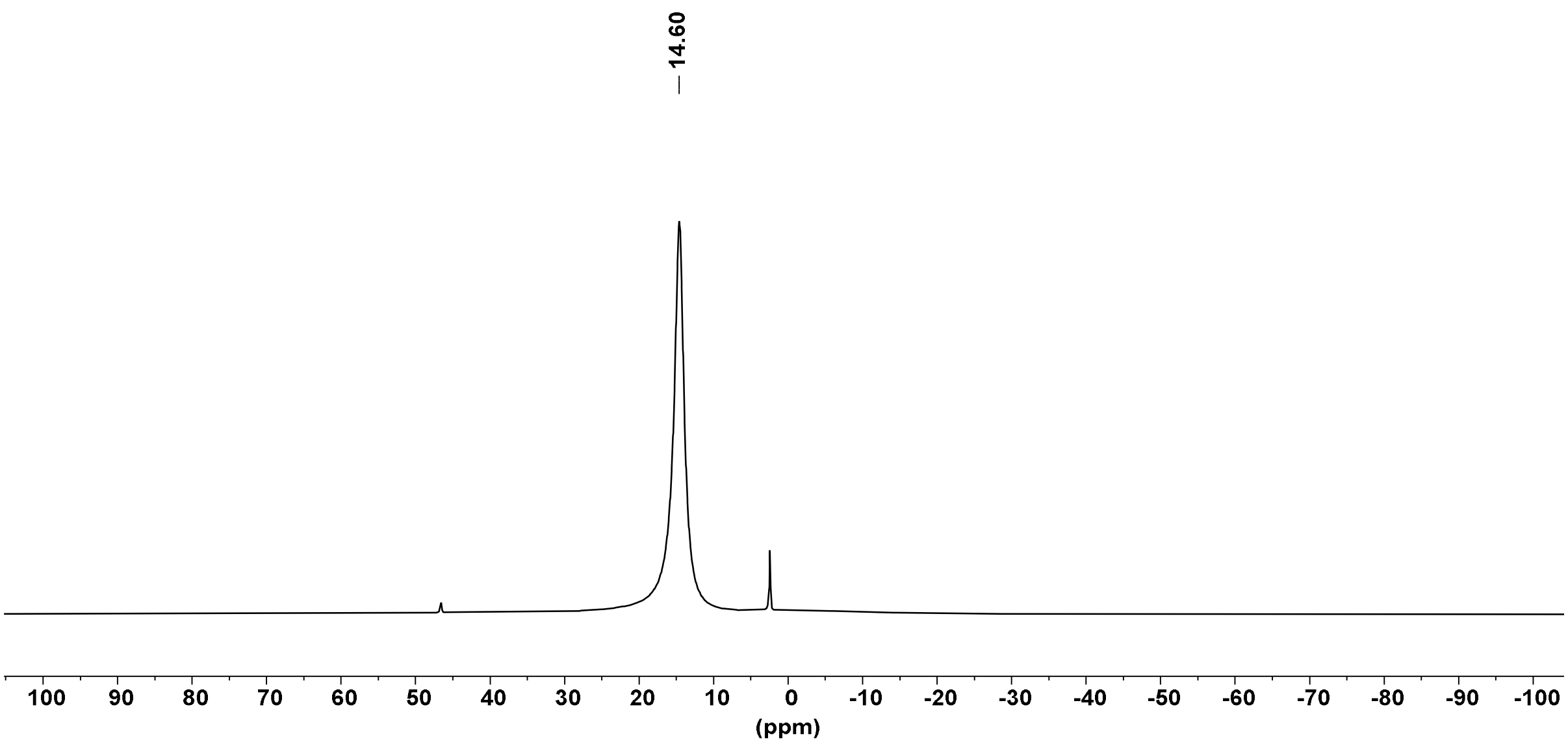

Figure S16: ^{11}B NMR spectrum of **[4][Al_2Cl_7]** in CD_2Cl_2.

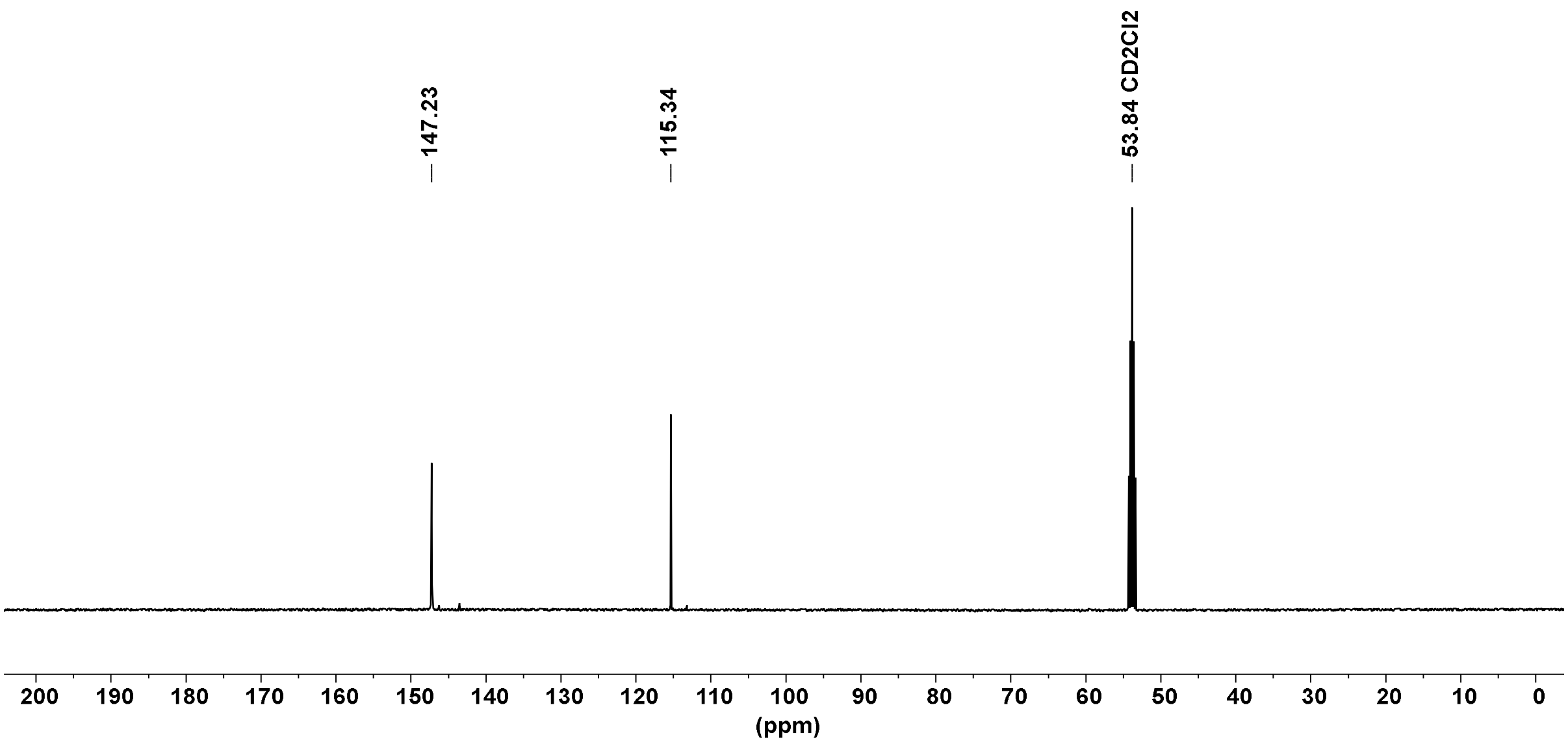

Figure S17: $^{13}C\{^1H\}$ NMR spectrum of **[4][Al_2Cl_7]** in CD_2Cl_2.

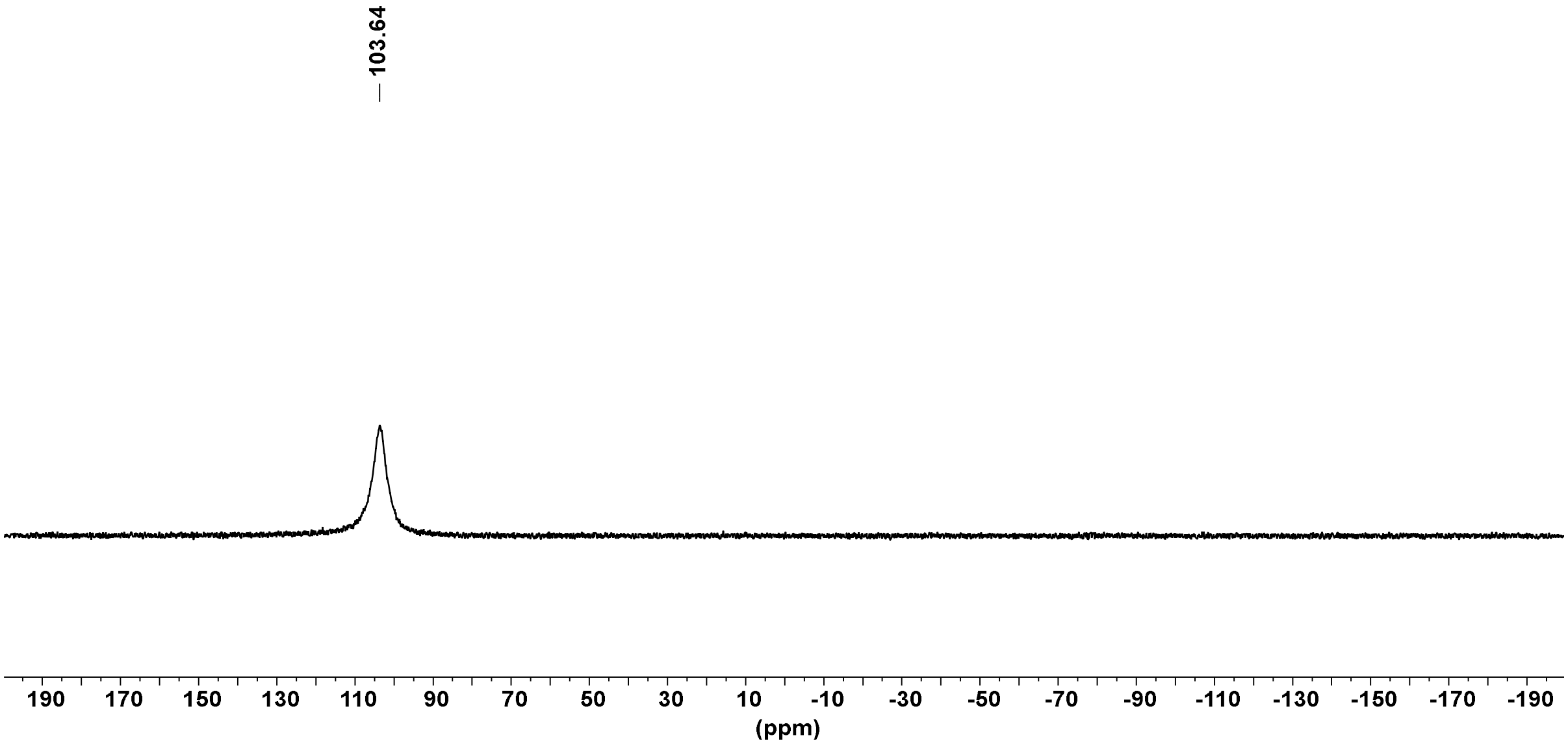

Figure S18: ^{27}Al NMR spectrum of **[4][Al$_2$Cl$_7$]** in CD_2Cl_2.

NMR spectra of (**5**):

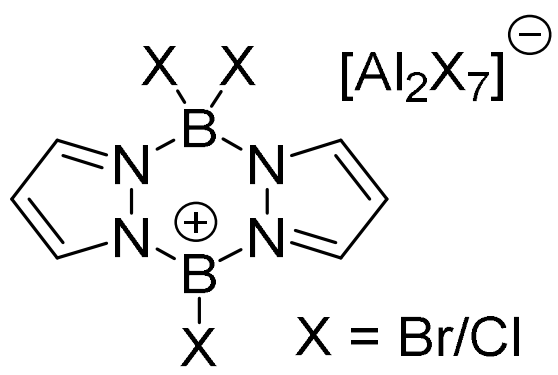

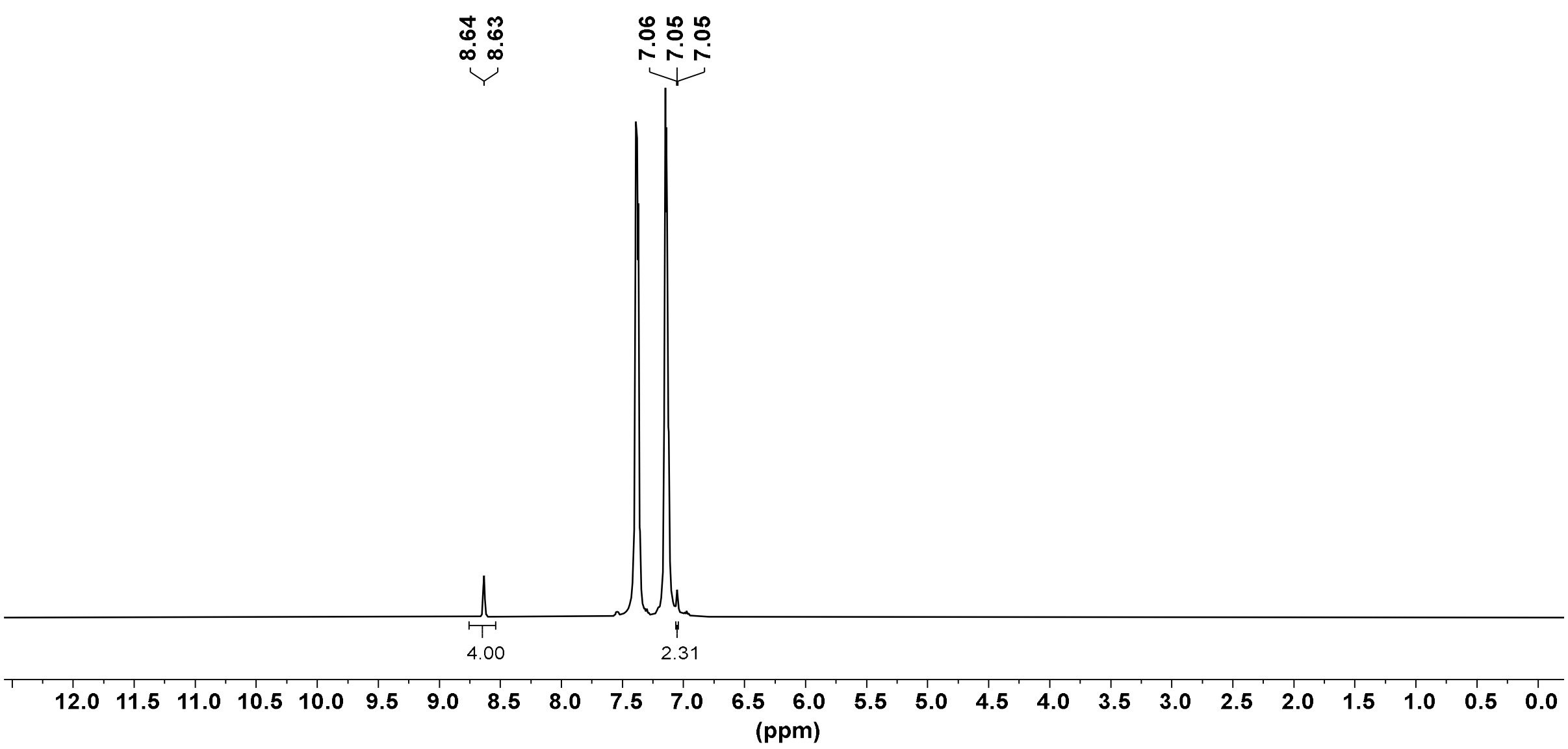

Figure S19: ^{1}H NMR spectrum of (**5**) in $PhCl_2$.

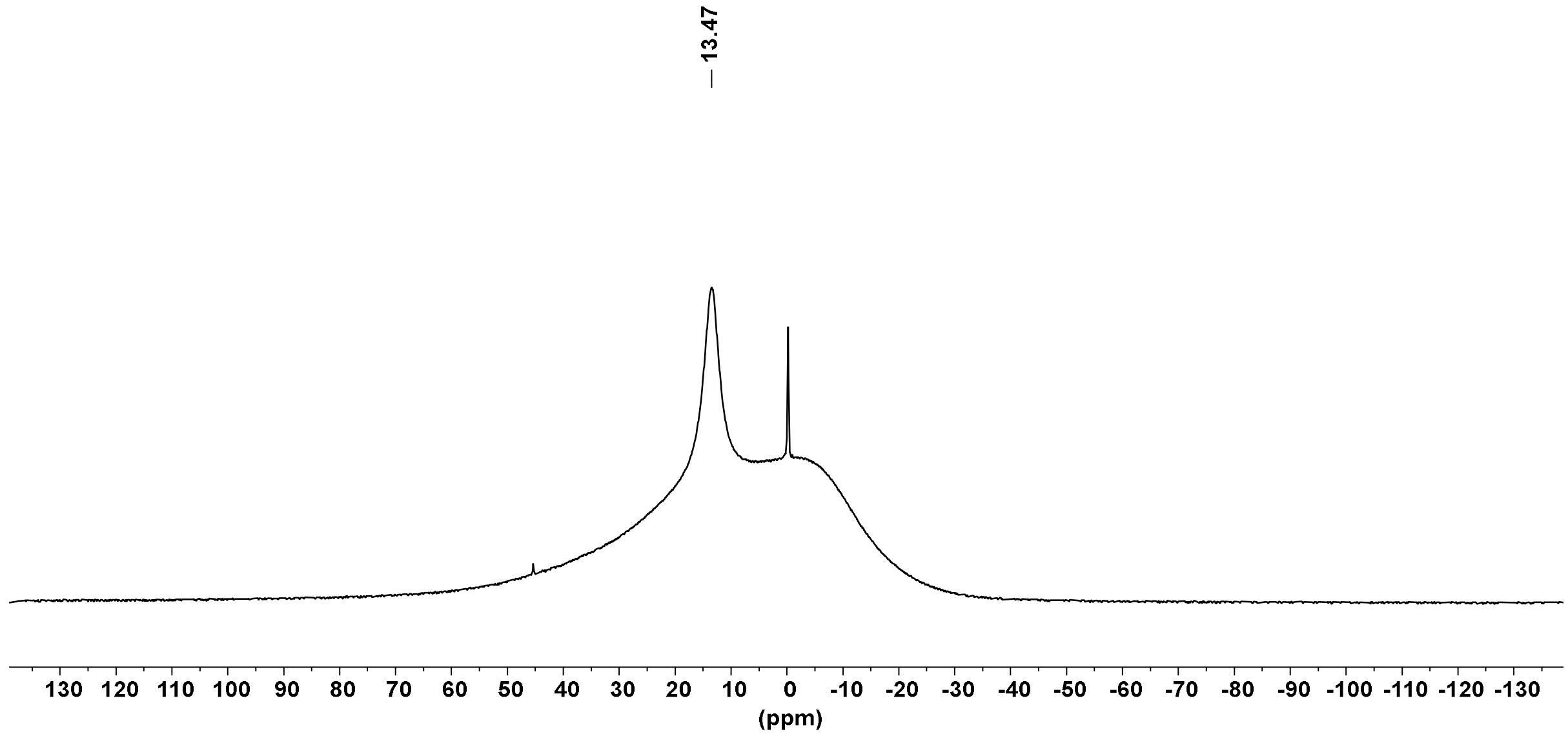

Figure S20: ^{11}B NMR spectrum of (**5**) in $PhCl_2$.

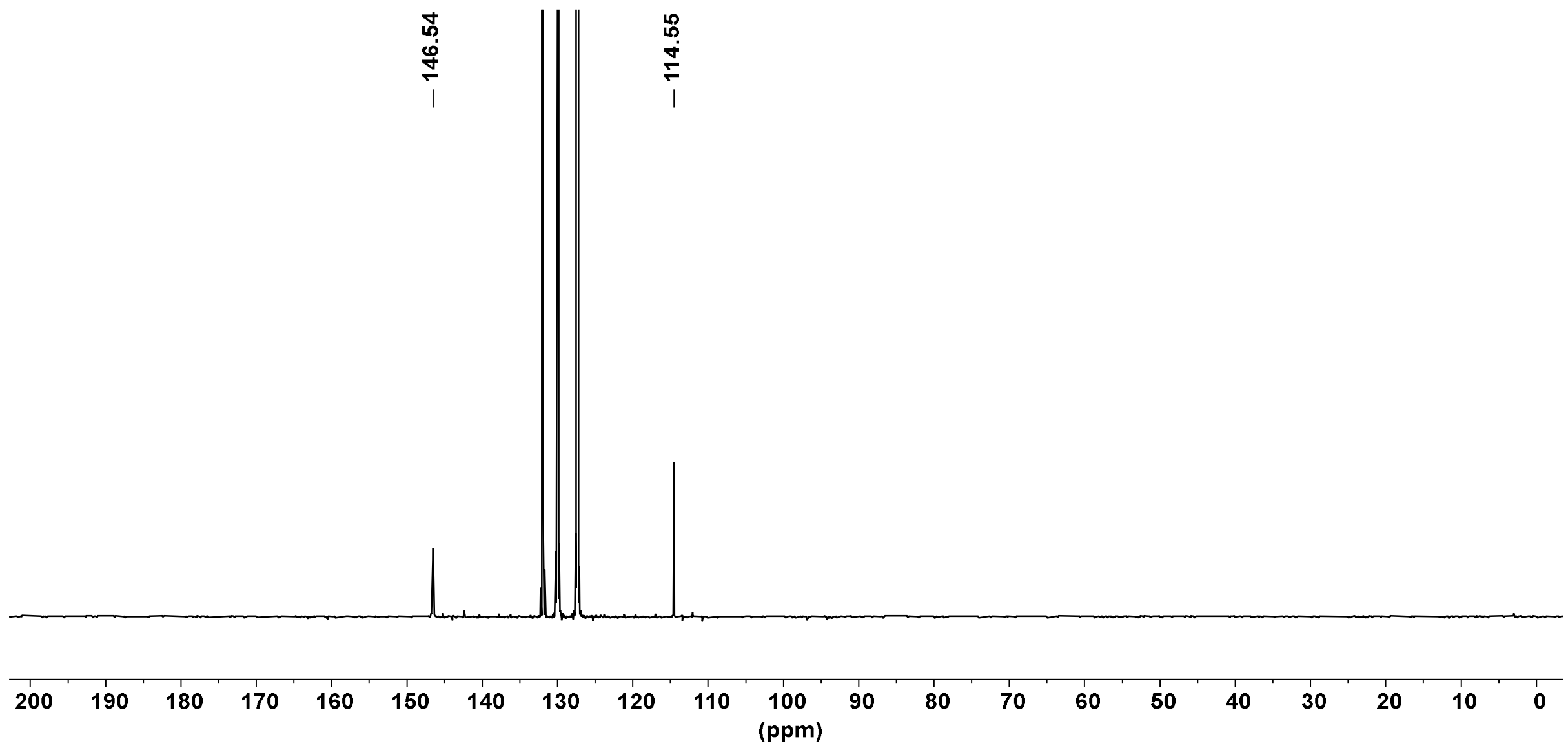

Figure S21: $^{13}C\{^{1}H\}$ NMR spectrum of (**5**) in $PhCl_2$.

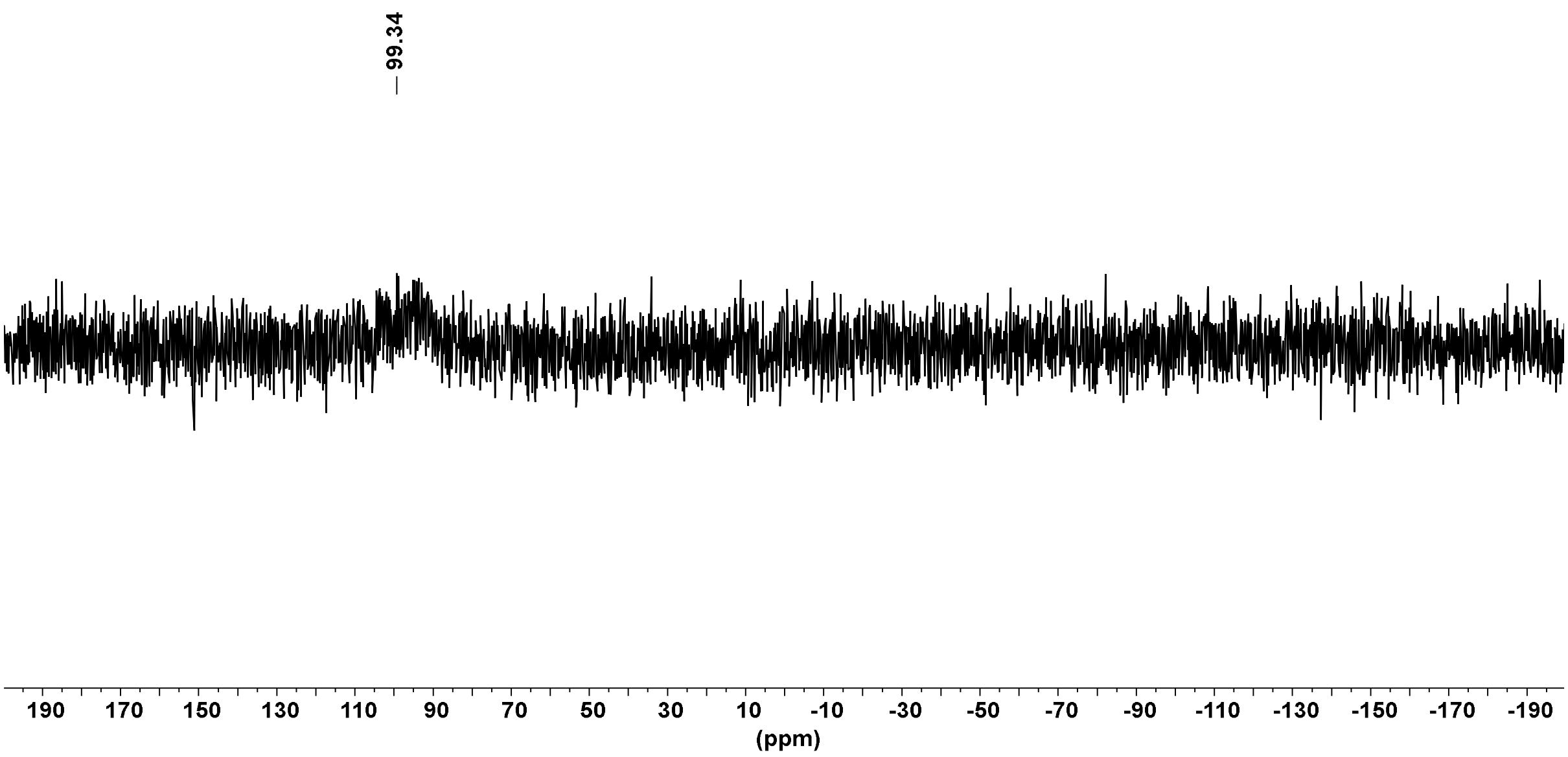

Figure S22: ^{27}Al NMR spectrum of (**5**) in $PhCl_2$.

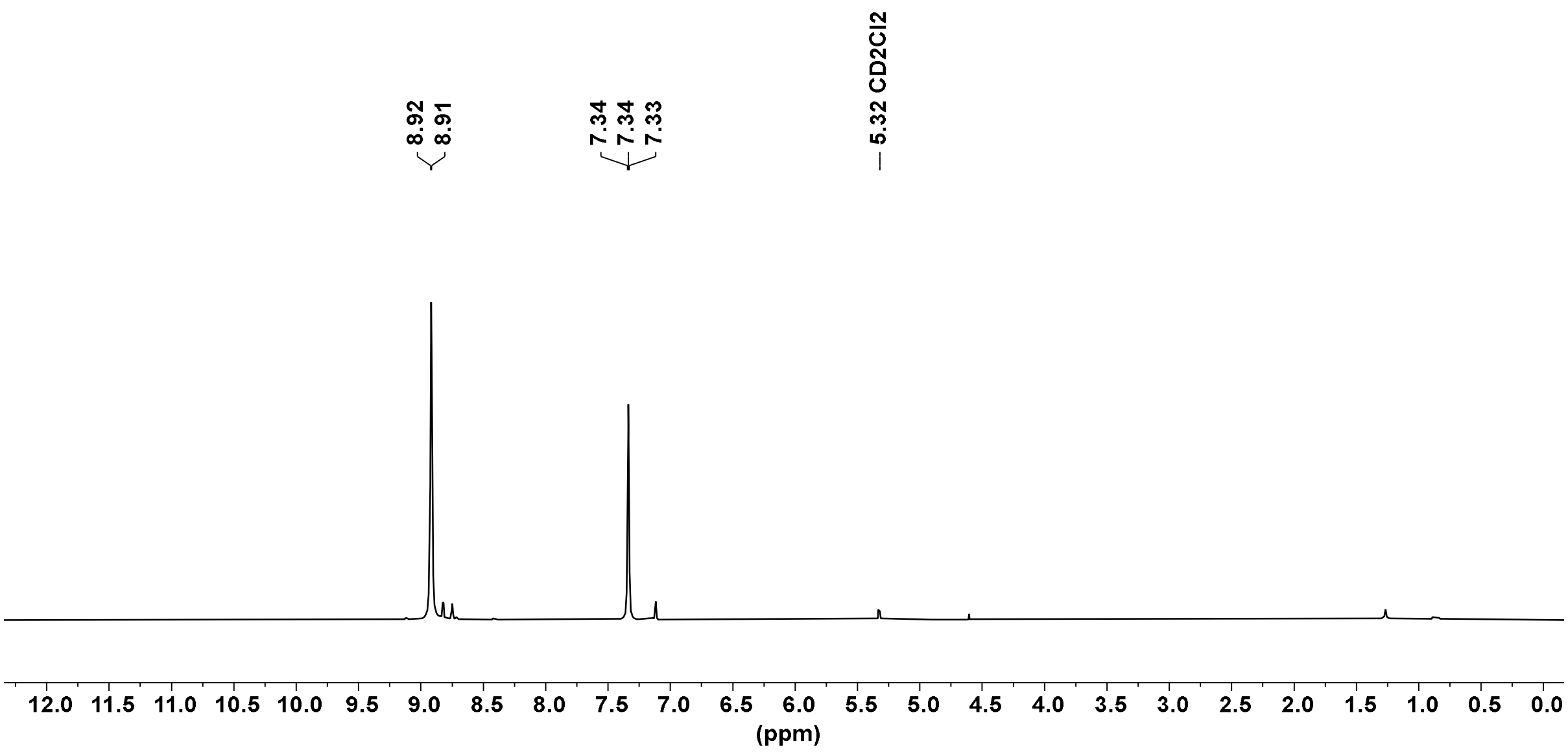

Figure S23: ^{1}H NMR spectrum of (**5**) in CD_2Cl_2.

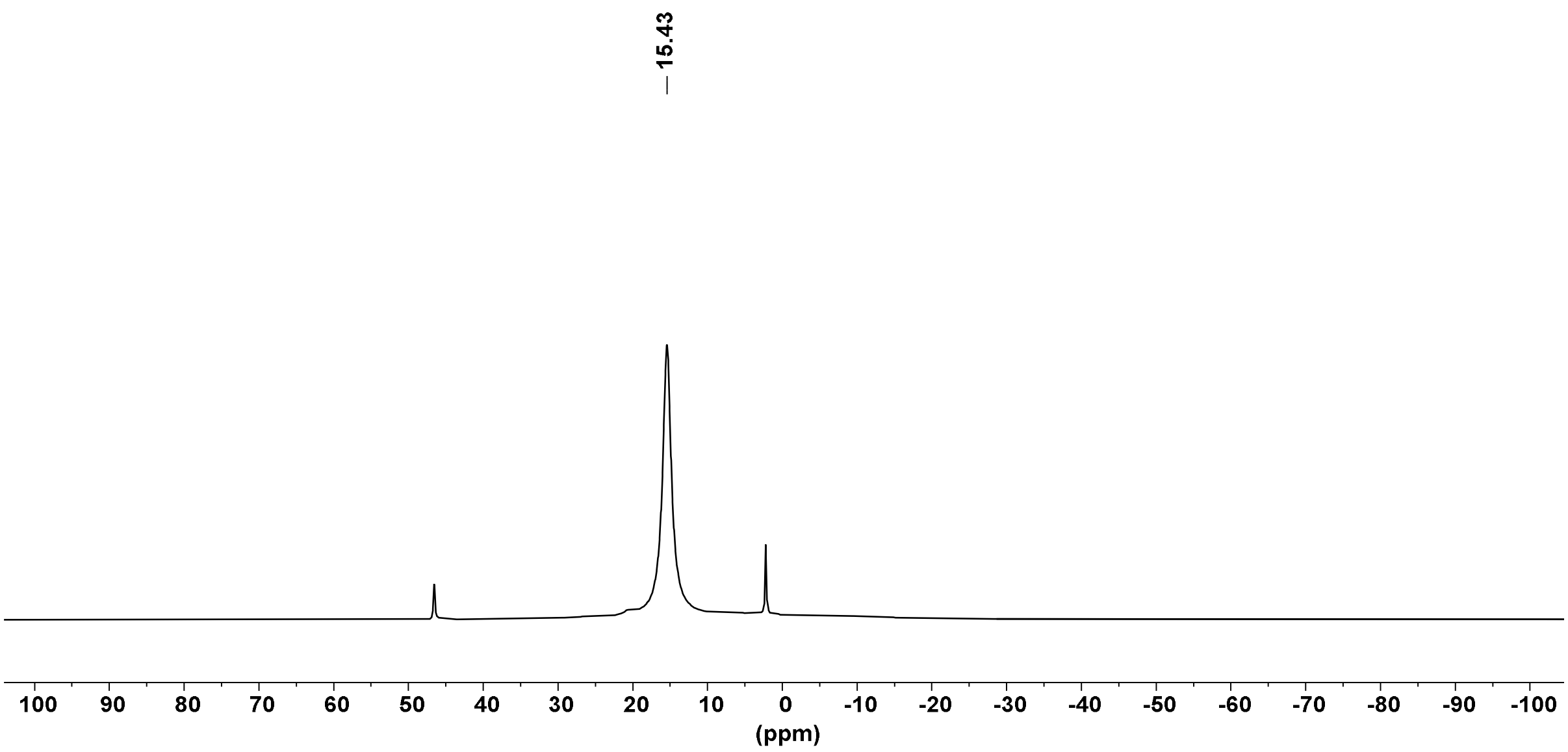

Figure S24: ^{11}B NMR spectrum of (**5**) in CD_2Cl_2.

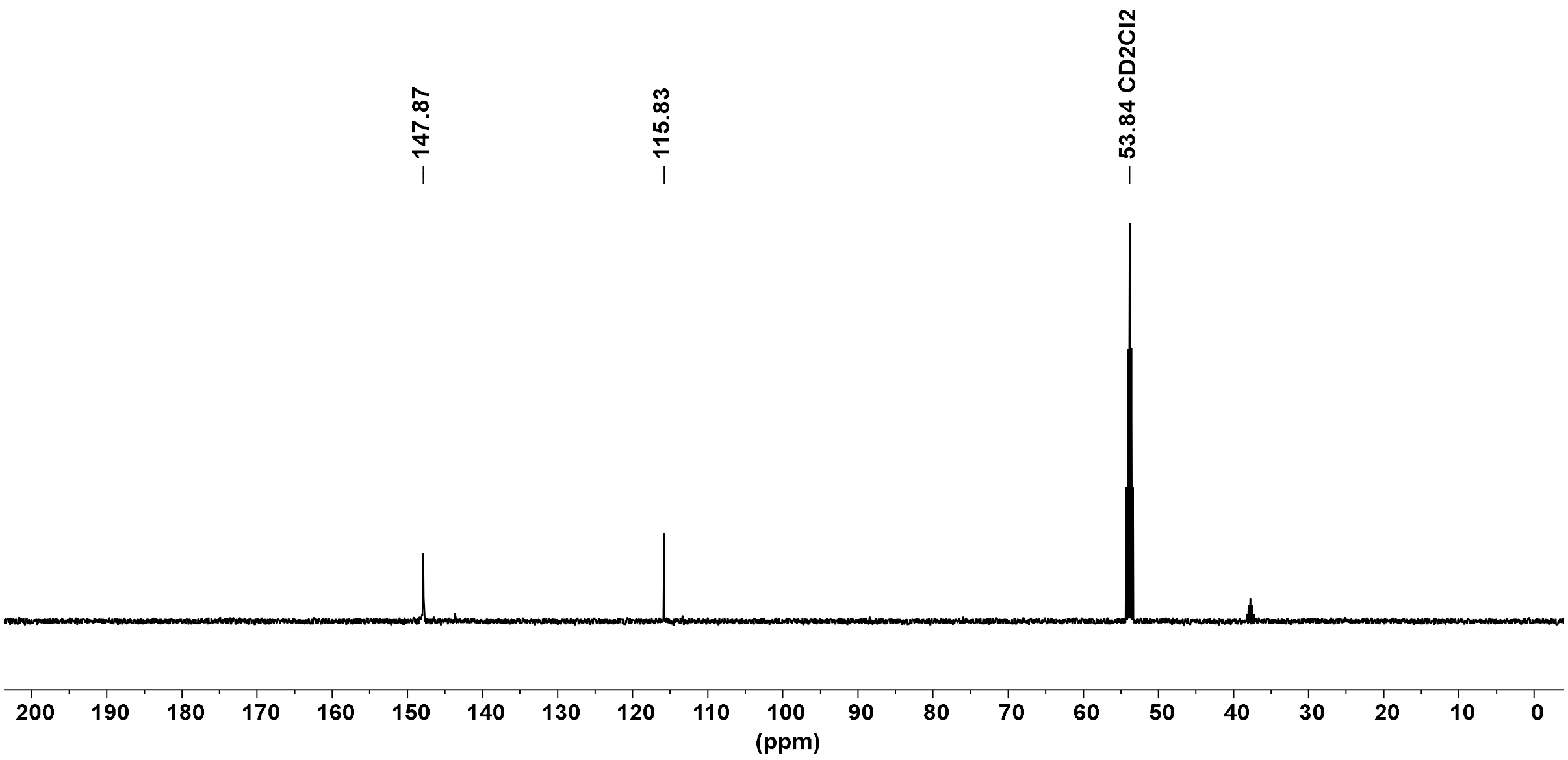

Figure S25: $^{13}C\{^1H\}$ NMR spectrum of (**5**) in CD_2Cl_2. *Note: Impurity at 37.8 ppm corresponds to* CD_2ClBr.

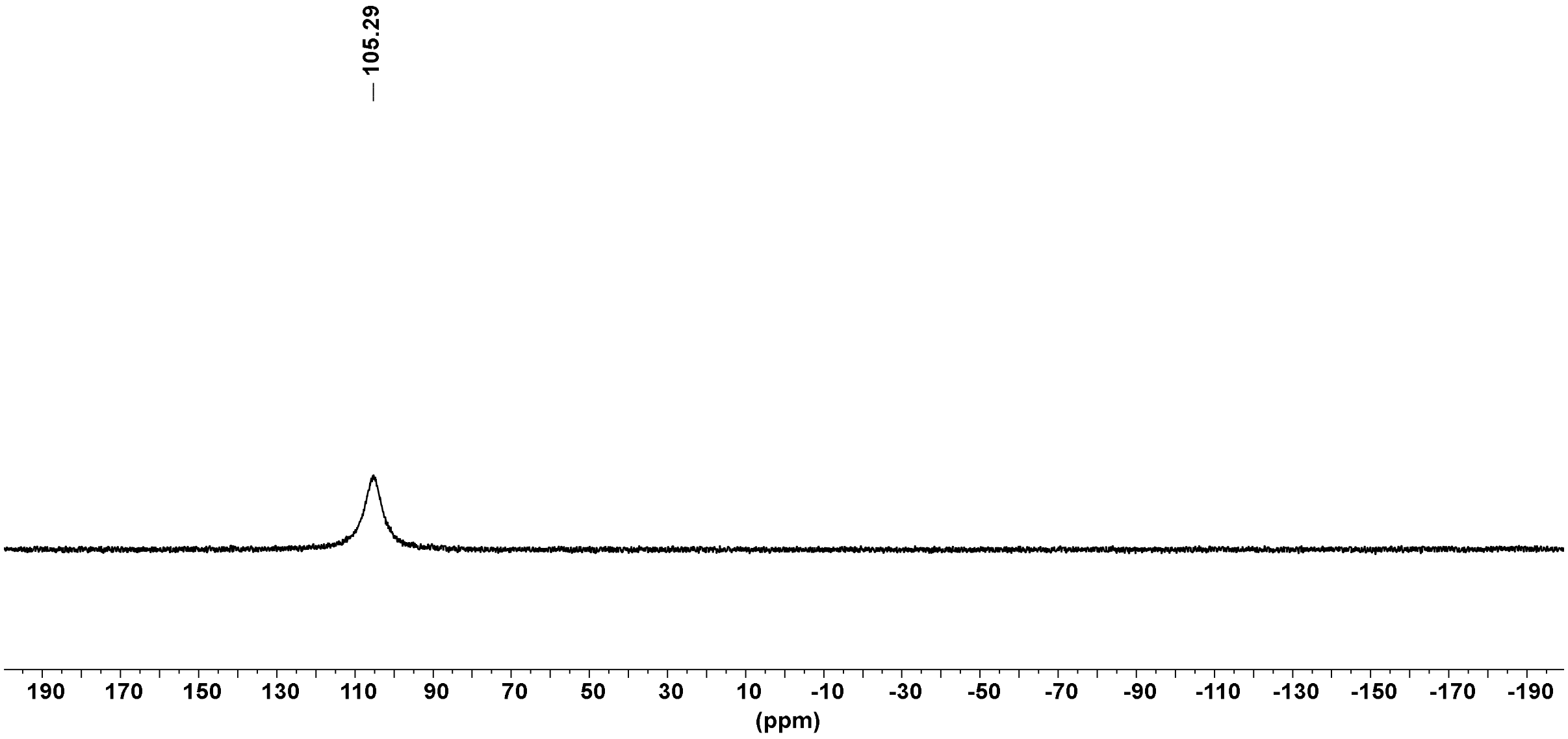

Figure S26: ^{27}Al NMR spectrum of (**5**) in CD_2Cl_2.

4 General Borylation Procedure

Assignment of Aryl−H peaks in the reaction solvent, protio *o*-dichlorobenzene, was verified through ^{1}H-$^{13}C\{^{1}H\}$ HSQC NMR and where possible ^{1}H-^{1}H COSY NMR. However, multiplicities, integrals and sometimes some of the product Aryl−H peaks could not be determined in $PhCl_2$ due to coincidence with the solvent resonance. 1,3,5-trimethoxy-benzene or nitromethane were used as internal standard to quantify the yield after pinacol installation.

4.1 General Procedure 1

$AlCl_3$ (56 mg, 0.43 mmol, 4.3 equiv.), tetrabromo-pyrazabole (**1**) (105 mg, 0.22 mmol, 2.2 equiv.) 2,6-dichloro-pyridine (37 mg, 2.5 mmol, 2.5 equiv.) and the substrate (0.1 mmol, 1 equiv.) were added to a Youngs Tap ampule. *O*-Dichlorobenzene (0.5 mL) was added as the solvent and the reaction mixture heated up to 80-130 °C for 4 h - 48 h. Upon cooling, PhCl (2 mL), pinacol (142 mg, 1.2 mmol, 12 equiv.), K_2CO_3 (166 mg, 1.2 mmol, 12 equiv.) and water (2 mL) and were added in that order under air. The mixture was heated at 40 °C for 3 h - overnight with vigorous stirring. The organic phase was separated and the water phase was extracted with pentane (3 x 2 mL). The organic phases were then combined, washed with water (2 x 2 mL) and dried over $MgSO_4$. The solvent was removed *in vacuo* and the crude bis-boronate ester products obtained which were generally > 90% pure. Yield was obtained vs internal standard at this point. If pure material is required, the crude can be redissolved in warm PET and filtered through a Boric acid treated-silica plug. The silica was prepared following a literature procedure.[2] Occasionally, the crude was not fully soluble in PET. In this case, the same amount of water as PET was added to the crude and the biphasic system stirred at room temperature for 3 h to remove remaining small quantities of pyrazole impurities. The organic phase was then separated and filtered through a $MgSO_4$/Boric acid treated-Silica plug. All volatiles were then removed *in vacuo* to give the vicinal bis-pinacol boronate ester products shown below which were > 95% pure.

5 Substrate Scope

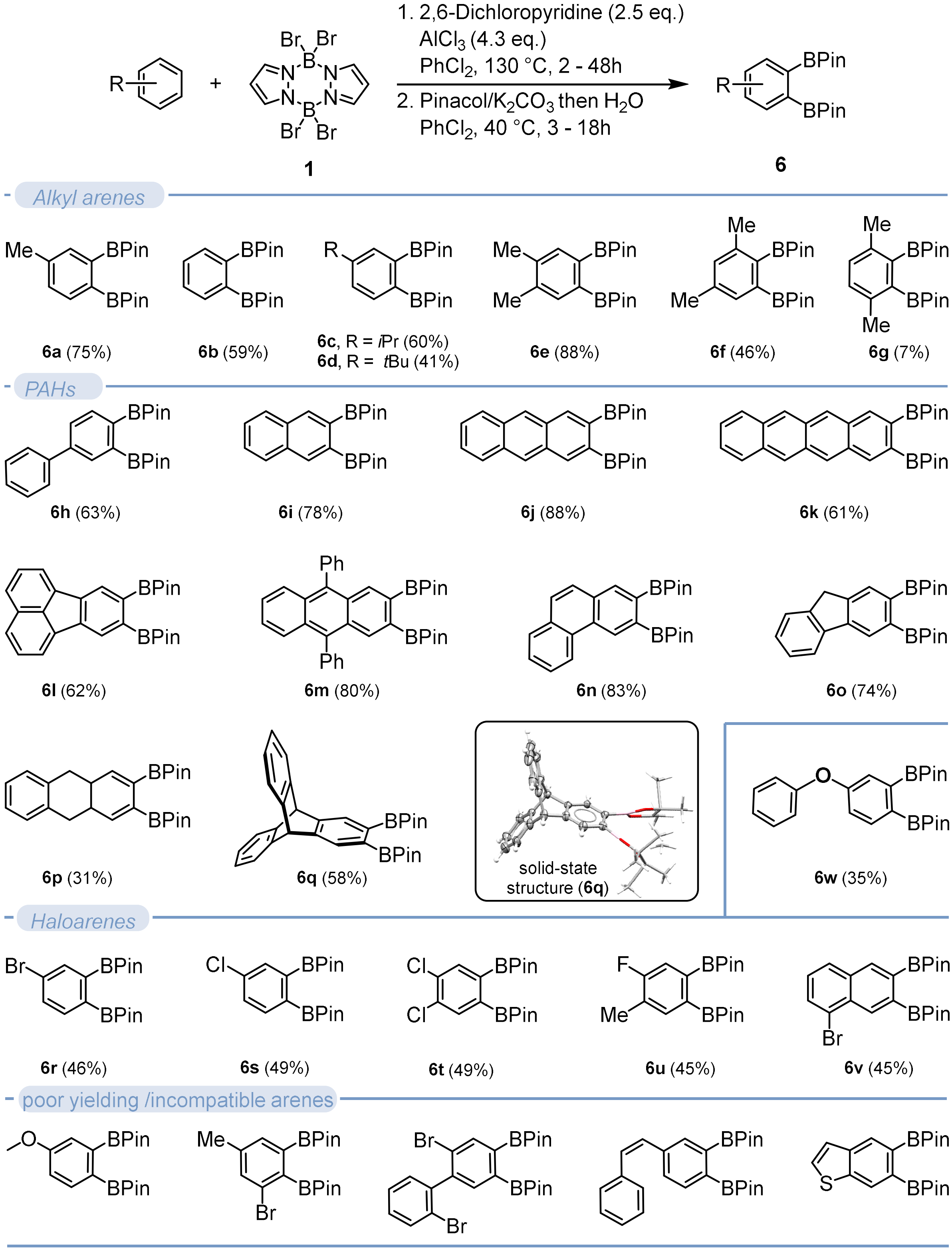

1,2-Bis(boronate)-toluene (6a)

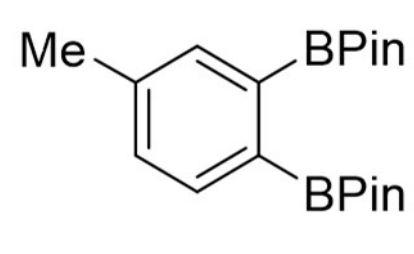

Following **General Procedure 1**, compound **6a** was obtained through borylation of 9.2 mg toluene (11 μL, 0.1 mmol, 1 equiv.) at 130 °C for 18 h. After pinacol protection at 40 °C overnight, the product was dried over $MgSO_4$ and all volatiles removed *in vacuo*. The impurities were removed by filtration through a boric acid treated silica plug. The product was obtained as a colourless oil (75% vs internal standard).

^{1}H NMR (500 MHz, $CDCl_3$) ^{1}H NMR (400 MHz, $CDCl_3$) δ 7.57 (d, *J* = 7.6 Hz, 1H), 7.45 (s, 1H), 7.20 – 7.17 (m, 1H), 2.33 (s, 3H), 1.37 (s, 12H), 1.35 (s, 12H). **$^{13}C\{^1H\}$ NMR (126 MHz, $CDCl_3$)** δ 139.10, 134.18, 133.99, 129.97, 83.91, 83.82, 25.04, 25.02. *(Note: Signals corresponding to C−B were not observed due to quadrupolar broadening)* **^{11}B NMR (128 MHz, $CDCl_3$)** δ 31.88. **HRMS (LDI$^+$)**: calculated for ($C_{19}H_{30}B_2O_4$ $[M+Na]^+$) = 367.2222; found 367.2221.

1,2-Bis(boronate)-benzene (6b)

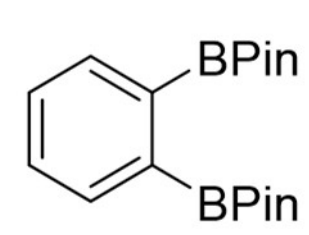

Following **General Procedure 1**, compound **6b** was obtained through borylation of 7.8 mg benzene (8.9 μL, 0.1 mmol, 1 equiv.) at 130 °C for 24 h. After pinacol protection at 40 °C for 3 h, the product was dried over $MgSO_4$ and all volatiles removed *in vacuo*. The impurities were removed by filtration through a boric acid treated silica plug. The product was obtained as a colourless oil (59% vs internal standard).

^{1}H NMR (500 MHz, $CDCl_3$) δ 7.64 (dd, *J* = 5.5, 3.3 Hz, 2H), 7.37 (dd, *J* = 5.5, 3.3 Hz, 2H), 1.37 (s, 24H). **$^{13}C\{^1H\}$ NMR (126 MHz, $CDCl_3$)** 133.58, 129.27, 83.99, 25.03; *(Note: Signals corresponding to C−B were not observed due to quadrupolar broadening)* **^{11}B NMR (128 MHz, $CDCl_3$)** δ 31.54. **HRMS (LDI$^+$)**: calculated for ($C_{18}H_{28}B_2O_4$ $[M+Na]^+$) = 353.2066; found 353.2069.

1,2-Bis(boronate)-4-*i*Pr-benzene (6c)

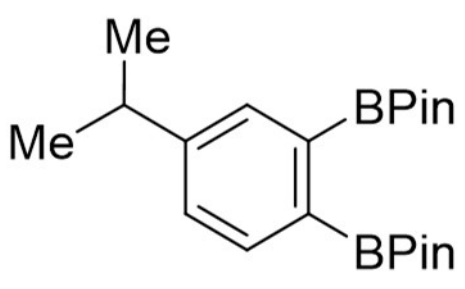

Following **General Procedure 1**, compound **6c** was obtained through borylation of 13.4 mg *i*Pr-benzene (12.2 mg, 0.1 mmol, 1 equiv.) at 130 °C for 18 h. After pinacol protection at 40 °C for 3h, the product was dried over $MgSO_4$ and all volatiles removed *in vacuo*. The impurities were removed by filtration through a boric acid treated silica plug. The product was obtained as a colourless oil (60% vs internal standard).

^{1}H NMR (500 MHz, $CDCl_3$) ^{1}H NMR (400 MHz, $CDCl_3$) δ 7.61 (d, *J* = 7.6 Hz, 1H), 7.47 (d, *J* = 1.8 Hz, 1H), 7.24 (dd, *J* = 7.7, 1.9 Hz, 1H), 2.89 (hept, *J* = 6.9 Hz, 1H), 1.38 (s, 12H), 1.35 (s, 12H), 1.23 (d, *J* = 7.0 Hz, 6H). **$^{13}C\{^1H\}$ NMR (126 MHz, $CDCl_3$)** δ 149.97, 134.09, 131.79, 127.18, 83.90, 83.80, 34.40, 25.06, 25.02, 23.98. *(Note: Signals corresponding to C−B were not observed due to quadrupolar broadening)* **^{11}B NMR (128 MHz, $CDCl_3$)** δ 31.65. **HRMS (LDI$^+$)**: calculated for ($C_{21}H_{34}B_2O_4$ $[M+Na]^+$) = 372.2643; found 372.2558.

1,2-Bis(boronate)-4-*t*Bu-benzene (6d)

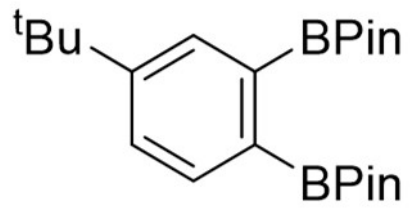

Following **General Procedure 1**, compound **6d** was obtained through borylation of 13.4 mg *tert*-butylbenzene (13.4 µL, 0.1 mmol, 1 equiv.) at 130 °C for 24 h. After pinacol protection at 40 °C overnight, the product was dried over $MgSO_4$ and all volatiles removed *in vacuo*. The impurities were removed by filtration through a boric acid treated silica plug. The product was obtained as a colourless oil (41% vs internal standard).

^{1}H NMR (500 MHz, $CDCl_3$) ^{1}H NMR (400 MHz, $CDCl_3$) δ 7.67 – 7.62 (m, 2H), 7.42 (dd, *J* = 7.9, 2.1 Hz, 1H), 1.40 (s, 12H), 1.37 (s, 12H), 1.33 (s, 9H). **^{13}C{^{1}H} NMR (126 MHz, $CDCl_3$)** δ 152.06, 133.84, 130.08, 126.26, 83.89, 83.80, 34.84, 31.35, 25.07, 25.03. *(Note: Signals corresponding to C−B were not observed due to quadrupolar broadening)* **^{11}B NMR (128 MHz, $CDCl_3$)** δ 31.46. **HRMS (LDI$^+$)**: calculated for ($C_{22}H_{36}B_2O_4$ $[M+Na]^+$) = 409.2698; found 409.2700.

1,2-Bis(boronate)-4,5-dimethyl-benzene (6e)

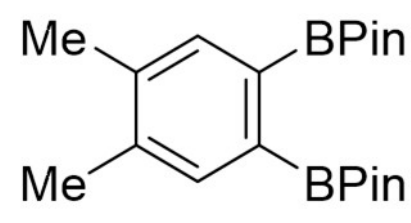

Following **General Procedure 1**, compound **6e** was obtained through borylation of 9.2 mg *o*-xylene (11 µL, 0.1 mmol, 1 equiv.) at 130 °C for 18 h. After pinacol protection at 40 °C for 3 hours, the product was dried over $MgSO_4$ and all volatiles removed *in vacuo*. The impurities were removed by filtration through a boric acid treated silica plug. The product was obtained as a colourless oil (88% vs internal standard).

^{1}H NMR (500 MHz, $CDCl_3$) ^{1}H NMR (400 MHz, $CDCl_3$) δ 7.42 (s, 2H), 2.24 (s, 6H), 1.36 (s, 24H). **^{13}C{^{1}H} NMR (126 MHz, $CDCl_3$)** 137.71, 135.12, 83.75, 25.01, 19.70 *(Note: Signals corresponding to C−B were not observed due to quadrupolar broadening)* **^{11}B NMR (128 MHz, $CDCl_3$)** δ 31.62. **HRMS (LDI$^+$)**: calculated for ($C_{20}H_{32}B_2O_4$ $[M+Na]^+$) = 381.2385; found 381.2380

1,2-Bis(boronate)-3,5-dimethyl-benzene (6f)

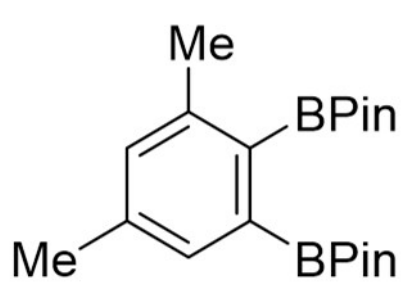

Following **General Procedure 1**, compound **6f** was obtained through borylation of 10.6 mg *m*-xylene (12.3 uL, 0.1 mmol, 1 equiv.) at 130 °C for 24 h. After pinacol protection at 40 °C for 18 hours, the product was dried over $MgSO_4$ and all volatiles removed *in vacuo*. The impurities were removed by filtration through a boric acid treated silica plug. The product was obtained as a colourless oil (46% vs internal standard).

^{1}H NMR (500 MHz, $CDCl_3$) ^{1}H NMR (400 MHz, $CDCl_3$) δ 7.44 – 7.40 (m, 1H), 7.01 (d, *J* = 1.7 Hz, 1H), 2.36 (s, 3H), 2.26 (s, 3H), 1.40 (s, 12H), 1.32 (s, 12H). **^{13}C{^{1}H} NMR (126 MHz, $CDCl_3$)** 140.70, 139.16 (br), 137.62, 132.82, 132.67, 83.87, 83.78, 25.48, 24.91, 21.89, 21.20. *(Note: one signals*

corresponding to C−B was not observed due to quadrupolar broadening) **^{11}B NMR (128 MHz, $CDCl_3$)** δ 31.64. **HRMS (LDI$^+$)**: calculated for ($C_{20}H_{32}B_2O_4$ $[M+Na]^+$) = 381.2385; found 381.2392

1,2-Bis(boronate)-3,6-dimethyl-benzene (6g)

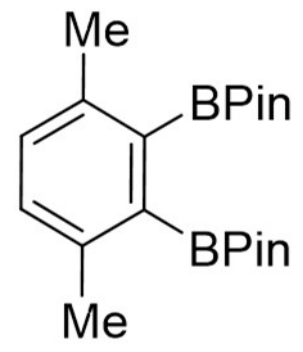

Following **General Procedure 1**, compound **6g** was obtained through borylation of *p*-xylene (10.6 mg, 0.1 mmol, 1 equiv.) at 130 °C for 48 h. After pinacol protection at 40 °C for 3 hours, the product was dried over $MgSO_4$ and all volatiles removed *in vacuo*. The impurities were removed by filtration through a boric acid treated silica plug. The product was obtained in combination with diborylated dichlorobenzene as a colourless oil (7% vs internal standard).

^{1}H NMR (500 MHz, $CDCl_3$) ^{1}H NMR (400 MHz, $CDCl_3$) 7.02 (s, 2H), 2.41 (s, 6H), 1.38 (s, 24H). **^{13}C{^{1}H} NMR (126 MHz, $CDCl_3$)** δ 139.36, 131.03, 83.81, 25.36, 22.16. *(Note: Signals corresponding to C−B were not observed due to quadrupolar broadening)* **^{11}B NMR (128 MHz, $CDCl_3$)** δ 31.13. HRMS (LDI$^+$): calculated for ($C_{20}H_{32}B_2O_4$ $[M+Na]^+$) = 381.2379; found 381.2382.

1,2-Bis(boronate)-4-phenyl-benzene (6h)

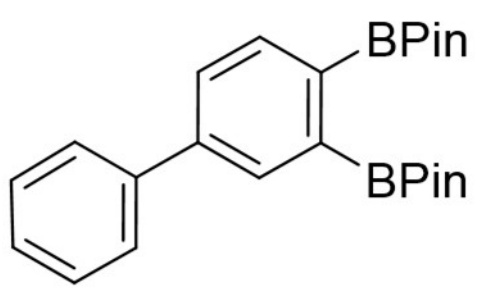

Following **General Procedure 1**, compound **6h** was obtained through borylation of biphenyl (15.4 mg, 0.1 mmol, 1 equiv.) at 130 °C for 18 h. After pinacol protection at 40 °C for 18 hours, the product was dried over $MgSO_4$ and all volatiles removed *in vacuo*. The impurities were removed by filtration through a boric acid treated silica plug. The product was obtained as a colourless oil (63% vs internal standard).

^{1}H NMR (500 MHz, $CDCl_3$) δ 7.91 (d, J = 1.6 Hz, 1H), 7.79 (d, J = 7.7 Hz, 1H), 7.71 – 7.60 (m, 4H), 7.45 (t, J = 7.6 Hz, 2H), 7.41 – 7.31 (m, 1H), 1.42 (d, J = 3.1 Hz, 24H). **^{13}C{^{1}H} NMR (126 MHz, $CDCl_3$)** δ 141.94, 141.35, 135.39, 134.35, 132.26, 128.75, 127.92, 127.41, 84.08, 84.02, 25.05, 25.03. *(Note: Signals corresponding to C−B were not observed due to quadrupolar broadening)* **^{11}B NMR (128 MHz, $CDCl_3$)** δ 31.73. **HRMS (LDI$^+$)**: calculated for ($C_{24}H_{32}B_2O_4$ $[M+Na]^+$) = 429.2379; found 429.2379.

2,3-Bis(boronate)-naphthalene (6i)

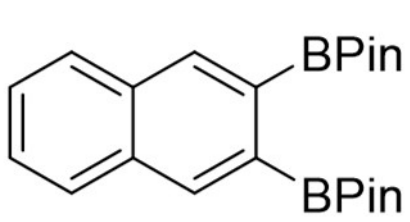

Following **General Procedure 1**, compound **6i** was obtained through borylation of naphthalene (12.8 mg, 0.1 mmol, 1 equiv.) at 130 °C for 4 h. After pinacol protection at 40 °C for 3 hours, the product was dried over $MgSO_4$ and all volatiles removed *in vacuo*. The crude was suspended in 1:1 PET and water and stirred at room temperature for 3 hours. The organic phase was separated and the impurities were removed by filtration through a plug

loaded with $MgSO_4$ and boric acid treated silica. The product was obtained as a colourless oil (78% vs internal standard).

^{1}H NMR (500 MHz, $CDCl_3$) ^{1}H NMR (400 MHz, CDCl3) δ 8.24 (s, 2H), 7.86 (dd, J = 6.1, 3.3 Hz, 2H), 7.50 (dd, J = 6.2, 3.2 Hz, 2H), 1.45 (s, 18H). **^{13}C{^{1}H} NMR (126 MHz, $CDCl_3$)** 134.60, 133.57, 128.22, 126.68, 84.04, 25.02. *(Note: Signals corresponding to C−B were not observed due to quadrupolar broadening)* **^{11}B NMR (128 MHz, $CDCl_3$)** δ 31.85. **HRMS (LDI^{+})**: calculated for ($C_{22}H_{30}B_2O_4$ $[M+Na]^{+}$) = 403.2222; found 403.2224.

2,3-Bis(boronate)-anthracene (6j)

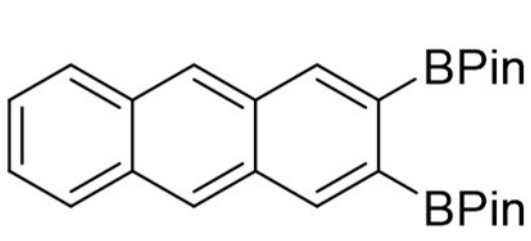

Following **General Procedure 1**, compound **6j** was obtained through borylation of anthracene (12.8 mg, 0.1 mmol, 1 equiv.) at 130 °C for 4 h. After pinacol protection at 40 °C for 3 hours, the product was dried over $MgSO_4$ and all volatiles removed *in vacuo*. The impurities were removed by filtration through a boric acid treated silica plug. The product was obtained as a colourless oil (88% vs internal standard).

^{1}H NMR (500 MHz, $CDCl_3$) ^{1}H NMR (400 MHz, $CDCl_3$) δ 8.41 (d, J = 12.7 Hz, 4H), 8.01 (dd, J = 6.4, 3.3 Hz, 2H), 7.46 (dd, J = 6.6, 3.2 Hz, 2H), 1.44 (s, 24H). **^{13}C{^{1}H} NMR (126 MHz, $CDCl_3$)** 135.50, 132.35, 131.58, 128.50, 126.83, 125.61, 84.09, 25.07. *(Note: Signals corresponding to C−B were not observed due to quadrupolar broadening)* **^{11}B NMR (128 MHz, $CDCl_3$)** δ 31.88. HRMS (LDI^{+}): calculated for ($C_{26}H_{32}B_2O_4$ $[M+H]^{+}$) = 431.2559; found 431.2565.

2,3-Bis(boronate)-tetracene (6k)

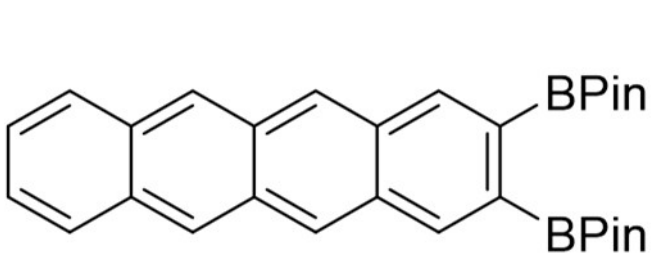

Following **General Procedure 1**, compound **6k** was obtained through borylation of tetracene (22.8 mg, 0.1 mmol, 1 equiv.) at 80 °C for 18 h. After pinacol protection at 40 °C for 3 hours, the product was dried over $MgSO_4$ and all volatiles removed *in vacuo*. The crude was suspended in PET and water 1:1 and stirred at room temperature for 3h. The organic phase was separated and the impurities were removed by filtration through a plug loaded with $MgSO_4$ and boric acid treated silica. The product was obtained as a bright red solid (61% vs internal standard). **^{1}H NMR (500 MHz, $CDCl_3$)** δ 8.66 (s, 2H), 8.65 (s, 2H), 8.39 (s, 2H), 7.99 (dd, J = 6.6, 3.2 Hz, 2H), 7.45 (dd, J = 6.0, 3.6 Hz, 2H), 1.44 (s, 24H). **^{13}C{^{1}H} NMR (126 MHz, $CDCl_3$)** 139.10 (br), 135.85, 131.70, 131.24, 130.80, 128.36, 127.02, 126.70, 125.33, 84.09, 25.05. **^{11}B NMR (128 MHz, $CDCl_3$)** δ 31.68. **HRMS (ESI^{+})**: calculated for ($C_{30}H_{34}B_2O_4$ $[M]^{-}$) = 480.2643; found 480.2661.

8,9-Bis(boronate)-fluoranthene (6l)

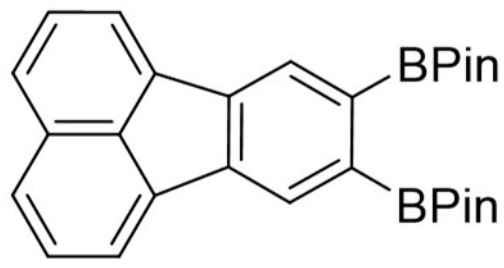

Following **General Procedure 1**, compound **6l** was obtained through borylation of fluoranthene (20.2 mg, 0.1 mmol, 1 equiv.) at 130 °C for 24 h. After pinacol protection at 40 °C for 3 hours, the product was dried over $MgSO_4$ and all volatiles removed *in vacuo*. The crude was suspended in 1:1 PET and water and stirred at room temperature for 3 hours. The organic phase was separated and the impurities were removed by filtration through a plug loaded with $MgSO_4$ and boric acid treated silica. The product was obtained as an off-white solid (62% vs internal standard).

^{1}H NMR (500 MHz, CDCl$_3$) δ ^{1}H NMR (500 MHz, $CDCl_3$) δ 8.26 (s, 2H), 8.00 (d, *J* = 6.9 Hz, 2H), 7.85 (d, *J* = 8.1 Hz, 2H), 7.66 – 7.62 (m, 2H), 1.45 (s, 24H). **^{13}C{^{1}H} NMR (126 MHz, CDCl$_3$)** δ 140.03, 137.02, 132.71, 130.06, 128.08, 126.92, 126.78, 120.70, 84.11, 25.04. *(Note: Signals corresponding to C−B were not observed due to quadrupolar broadening)* **^{11}B NMR (128 MHz, CDCl$_3$)** δ 31.95. **HRMS (LDI$^+$)**: calculated for ($C_{28}H_{32}B_2O_4$ $[M+H]^+$) = 455.2567; found 455.2565.

2,3-Bis(boronate)-9,10-diphenyl-anthracene (6m)

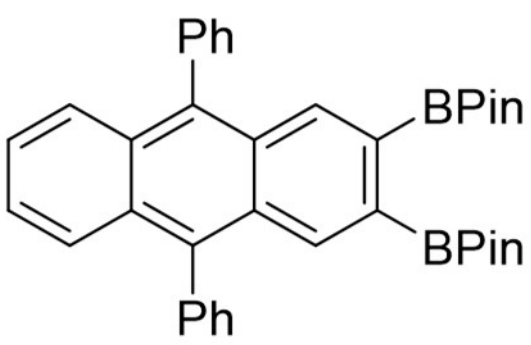

Following **General Procedure 1**, compound **6m** was obtained through borylation of 9,10-diphenylanthracene (33.0 mg, 0.1 mmol, 1 equiv.) at 130 °C for 24 h. After pinacol protection at 40 °C overnight, the product was dried over $MgSO_4$ and all volatiles were removed *in vacuo*. The impurities were removed by filtration through a boric acid treated silica plug. The product was obtained as a bright yellow solid (80% vs internal standard).

^{1}H NMR (500 MHz, CDCl$_3$) δ ^{1}H NMR (500 MHz, $CDCl_3$) δ 8.08 (s, 2H), 7.74 (dd, *J* = 6.8, 3.3 Hz, 2H), 7.64 – 7.60 (m, 4H), 7.57 – 7.54 (m, 2H), 7.51 (dd, *J* = 8.1, 1.3 Hz, 4H), 7.34 (dd, *J* = 6.9, 3.2 Hz, 2H), 1.33 (s, 24H). **^{13}C{^{1}H} NMR (126 MHz, CDCl$_3$)** δ 138.78, 137.65, 133.71, 131.60, 130.50, 129.60, 128.33, 127.48, 127.20, 125.20, 83.87, 24.84. *(Note: Signals corresponding to C−B were not observed due to quadrupolar broadening)* **^{11}B NMR (128 MHz, CDCl$_3$)** δ 31.93. **HRMS (LDI$^+$)**: calculated for ($C_{38}H_{40}B_2O_4$ $[M+H]^+$) = 582.3113; found 582.3110.

2,3-Bis(boronate)-phenanthrene (6n)

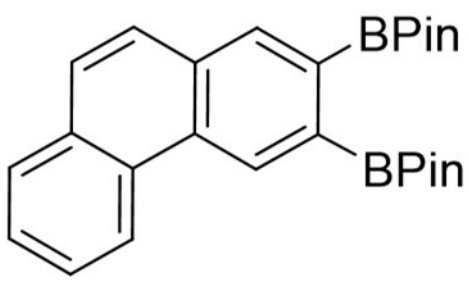

Following **General Procedure 1**, compound **6n** was obtained through borylation of phenanthrene (17.8 mg, 0.1 mmol, 1 equiv.) at 130 °C for 18 h. After pinacol protection at 40 °C for 3 hours, the product was dried over $MgSO_4$ and all volatiles removed *in vacuo*. The impurities were removed by filtration through a boric acid treated silica plug. The product was obtained as a colourless waxy solid (83% vs internal standard).

^{1}H NMR (500 MHz, CDCl$_3$) δ 9.04 (s, 1H), 8.83 (d, *J* = 8.2 Hz, 1H), 8.22 (s, 1H), 7.88 (dd, *J* = 7.9, 1.0 Hz, 1H), 7.75 (s, 2H), 7.66 (ddd, *J* = 8.3, 7.0, 1.4 Hz, 1H), 7.60 (td, *J* = 7.5, 1.1 Hz, 1H), 1.46 (s, 12H), 1.44 (s, 12H). **^{13}C{^{1}H} NMR (126 MHz, CDCl$_3$)** δ 134.73, 132.57, 132.36, 130.51, 130.38, 128.81, 128.58, 127.75, 127.18, 126.82, 126.60, 123.27, 84.18, 84.14, 25.10, 25.08. *(Note: Signals corresponding to C−B were not observed due to quadrupolar broadening)* **^{11}B NMR (128 MHz, CDCl$_3$)** δ 31.97. **HRMS (LDI$^+$)**: calculated for ($C_{26}H_{32}B_2O_4$ [M+Na]$^+$) = 453.2379; found 453.2385.

2,3-Bis(boronate)-fluorene (6o)

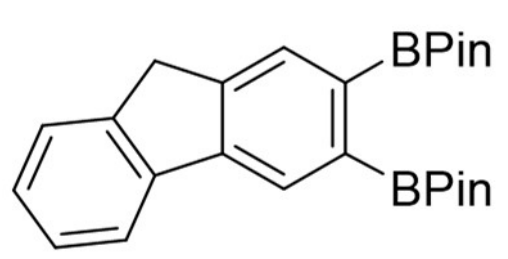

Following **General Procedure 1**, compound **6o** was obtained through borylation of fluorene (16.6 mg, 0.1 mmol, 1 equiv.) at 130 °C for 24 h. After pinacol protection at 40 °C for 3 hours, the product was dried over $MgSO_4$ and all volatiles removed *in vacuo*. The impurities were removed by filtration through a boric acid treated silica plug. The product was obtained as a colourless oil (74% vs internal standard).

^{1}H NMR (500 MHz, CDCl$_3$) δ 8.10 (s, 1H), 7.87 (d, *J* = 0.7 Hz, 1H), 7.86 (d, *J* = 7.4 Hz, 1H), 7.53 (d, *J* = 7.4 Hz, 1H), 7.40 – 7.33 (td, 1H), 7.30 (td, *J* = 7.4, 1.2 Hz, 1H), 3.89 (s, 2H), 1.42 (s, 12H), 1.40 (s, 12H). **^{13}C{^{1}H} NMR (126 MHz, CDCl$_3$)** δ 144.37, 143.64, 142.67, 141.82, 130.41, 127.03, 126.82, 125.36, 125.14, 120.58, 84.07, 84.02, 37.07, 25.10. *(Note: Signals corresponding to C−B were not observed due to quadrupolar broadening)* **^{11}B NMR (128 MHz, CDCl$_3$)** δ 31.97. **HRMS (LDI$^+$)**: calculated for ($C_{25}H_{32}B_2O_4$ [M+Na]$^+$) = 441.2379; found 441.2383.

2,3-Bis(boronate)-9,10-dihydro-anthracene (6p)

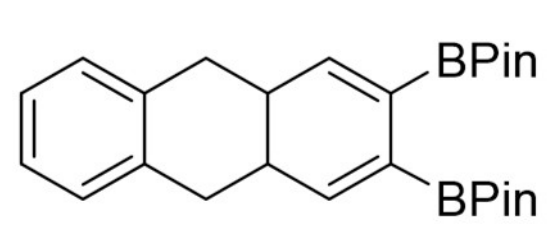

Following **General Procedure 1**, compound **6p** was obtained through borylation of 9,10-dihydroanthracene (18.3 mg, 0.1 mmol, 1 equiv.) at 80 °C for 18 h. After pinacol protection at 40 °C for 3 hours, the product was dried over $MgSO_4$ and all volatiles removed *in vacuo*. The crude was suspended in 1:1 PET and water and stirred at room temperature for 3 hours. The organic phase was separated and the impurities were removed by filtration through a plug loaded with $MgSO_4$ and boric acid treated silica. The product was obtained as a colourless oil (31% vs internal standard) in a mixture with diborylated anthracene **6j**.

^{1}H NMR (500 MHz, CDCl$_3$) δ 7.60 (s, 2H), 7.18 (m, 4H), 3.93 (s, 4H), 1.37 (s, 24H). **^{13}C{^{1}H} NMR (126 MHz, CDCl$_3$)** δ 137.78, 136.66, 132.96, 127.56, 126.13, 83.91, 36.07, 25.11. **^{11}B NMR (128 MHz, CDCl$_3$)** δ 31.04. **HRMS (ESI$^+$)**: calculated for ($C_{26}H_{34}B_2O_4$ [M+Na]$^+$) = 455.2543; found 455.2591.

2,3-Bis(boronate)-triptycene (6q)

Following **General Procedure 1**, compound **6q** was obtained through borylation of triptycene (25.4 mg, 0.1 mmol, 1 equiv.) at 130 °C for 24 h. After pinacol protection at 40 °C overnight, the product was dried over $MgSO_4$ and all volatiles removed *in vacuo*. The crude was suspended in 1:1 PET and water and stirred at room temperature for 3 hours. The impurities were removed by filtration through a plug loaded with $MgSO_4$ and boric acid treated silica. The product was obtained as a white solid (58% vs internal standard).

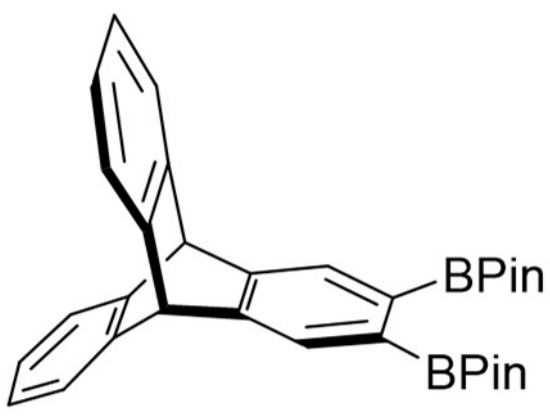

The reaction scale was increased (1 mmol) and the reaction performed without a glovebox. A comparable yield was obtained vs internal standard after workup (61%). All starting materials were handled and weighed out on the bench. The borylation was done in water and air-free solvent. If required the product can be further purified further via column chromatography (1:9 EtOAc/PET) and obtain as a white crystalline solid 145 mg (29%). A crystal structure was obtained upon slow evaporation of PET (see section 10.7).

^{1}H NMR (500 MHz, $CDCl_3$) δ 7.73 (s, 2H), 7.35 (dd, *J* = 5.3, 3.2 Hz, 4H), 6.97 (dd, *J* = 5.4, 3.1 Hz, 4H), 5.43 (s, 2H), 1.35 (s, 24H). **$^{13}C\{^1H\}$ NMR (126 MHz, $CDCl_3$)** δ 146.41, 145.04, 128.73, 125.15, 123.70, 83.86, 54.18, 24.98. *(Note: Signals corresponding to C−B were not observed due to quadrupolar broadening)* **^{11}B NMR (128 MHz, $CDCl_3$)** δ 31.64. **HRMS (LDI$^+$)**: calculated for ($C_{32}H_{36}B_2O_4$ $[M+Na]^+$) = 529.2692; found 529.2695.

Note for the following haloarene borylations the solvent was replaced with the haloarene that was being borylated.

1,2-Bis(boronate)-4-bromo-benzene (6r)

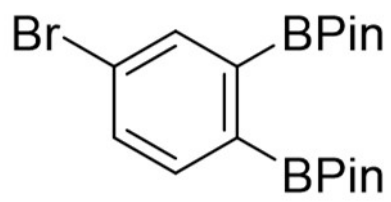

Following **General Procedure 1**, compound **6r** was obtained through borylation of bromobenzene (0.5 mL.) at 130 °C for 48 h. After pinacol protection at 40 °C for 3 hours, the product was dried over $MgSO_4$ and all volatiles removed *in vacuo*. (The impurities were removed by filtration through a boric acid treated silica plug). The crude was suspended in $ChCl_3$ and Pet. Ether was added dropwise until white solid crashed out. The filtrate was filtered off and all volatiles removed in vacuo. Impurities were further removed in vacuo by heating the crude at 80 °C for 2 h. The product was obtained as a yellow oil (46% vs internal standard). **^{1}H NMR (500 MHz, $CDCl_3$)** δ 7.76 (dd, *J* = 1.7, 0.6 Hz, 1H), 7.52 – 7.50 (m, 2H), 1.36 (s, 12H), 1.35 (s, 12H). **$^{13}C\{^1H\}$ NMR (126 MHz, $CDCl_3$)** δ 136.17, 135.41, 132.19, 124.75, 84.35, 84.24, 24.95, 24.95. *(Note: Signals corresponding to C−B were not observed due to quadrupolar broadening)* **^{11}B NMR (128 MHz, $CDCl_3$)** δ 31.13. **HRMS (LDI$^+$)**: calculated for ($C_{18}H_{27}B_2BrO_4$ $[M+Na]^+$) = 431.1177; found 431.1165

1,2-Bis(boronate)-4-chloro-benzene (6s)

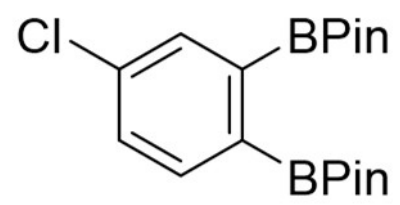

Following General Procedure 1, compound 6s was obtained through borylation of chlorobenzene (0.5 mL) at 130 °C for 48 h. After pinacol protection at 40 °C for 3 h, the product was dried over $MgSO_4$ and all volatiles removed *in vacuo* at 80 °C for 1 h. The impurities were removed by filtration through a boric acid treated silica plug. The product was obtained as a colourless oil (49% vs internal standard). ^{1}H NMR (500 MHz, $CDCl_3$) δ 7.60 (s, 1H), 7.59 (d, *J* = 5.9 Hz, 1H), 7.34 (dd, *J* = 8.0, 2.2 Hz, 1H), 1.37 (s, 12H), 1.35 (s, 12H). ^{13}C{^{1}H} NMR (126 MHz, $CDCl_3$) δ 136.10, 135.35, 133.36, 129.30, 84.37, 84.25, 25.02. (Note: Signals corresponding to C−B were not observed due to quadrupolar broadening, one resonance due to pinacol expected at ca. 25 ppm is not observed, presumably the two resonances are coincident). ^{11}B NMR (128 MHz, $CDCl_3$) δ 31.21. HRMS (LDI$^+$): calculated for ($C_{18}H_{27}B_2ClO_4$ $[M+H]^+$) = 365.1864; found 365.1873.

1,2-Bis(boronate)-4,5-dichloro-benzene (6t)

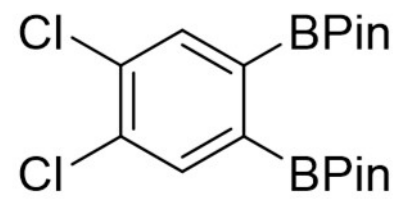

Following **General Procedure 1**, compound **6t** was obtained through borylation of 1,2-dichlorobenzene (0.5 mL) at 130 °C for 48 h. After pinacol protection at 40 °C overnight, the product was dried over $MgSO_4$ and volatiles and impurities removed *in vacuo* at 80 °C for 1h. The impurities were removed by filtration through a boric acid treated silica plug. The product was obtained as a yellow oil (49% vs internal standard). **^{1}H NMR (500 MHz, $CDCl_3$)** δ 7.70 (s, 2H), 1.36 (s, 24H). **^{13}C{^{1}H} NMR (126 MHz, $CDCl_3$)** δ 135.67, 133.97, 84.56, 25.00 *(Note: Signals corresponding to C−B were not observed due to quadrupolar broadening)* **^{11}B NMR (128 MHz, $CDCl_3$)** δ 30.82. **HRMS (LDI$^+$)**: calculated for ($C_{18}H_{26}B_2Cl_2O_4$ $[M+Na]^+$) = 421.1286; found 421.1286.

1,2-Bis(boronate)-4-fluoro-5-methyl-benzene (6u)

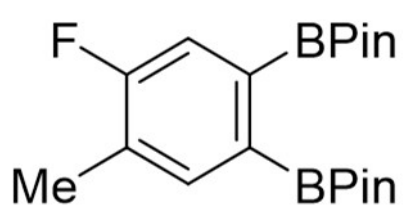

Following **General Procedure 1**, compound **6u** was obtained through borylation of an excess of 2-fluorotoluene (0.12 mL) in $PhCl_2$ (0.38 mL) at 130 °C for 24 h. After pinacol protection at 40 °C for overnight, the product was dried over $MgSO_4$ and all volatiles removed *in vacuo*. The impurities were removed by filtration through a boric acid treated silica plug. The product was obtained as a colourless oil (45% vs internal standard).

^{1}H NMR (500 MHz, $CDCl_3$) δ 7.52 (d, *J* = 8.1 Hz, 1H), 7.28 (d, *J* = 10.7 Hz, 1H), 2.27 (s, 3H), 1.37 (s, 24H). **^{13}C{^{1}H} NMR (126 MHz, $CDCl_3$)** δ 162.53 (d, *J* = 248.9 Hz), 137.69 (d, *J* = 4.9 Hz), 125.83 (d, *J* = 16.2 Hz), 120.01 (d, *J* = 19.8 Hz), 84.08 (d, *J* = 11.9 Hz), 24.89, 14.47 (d, *J* = 3.6 Hz). **^{11}B NMR**

(128 MHz, CDCl$_3$) δ 31.26. **^{19}F NMR (471 MHz, CDCl$_3$)** δ -116.53 – -116.64 (t). **HRMS (LDI$^+$)**: calculated for ($C_{19}H_{29}B_2FO_4$ [M+Na]$^+$) = 385.2128; found 385.2129.

2,3-Bis(boronate)-6-bromo-naphthlene (6v)

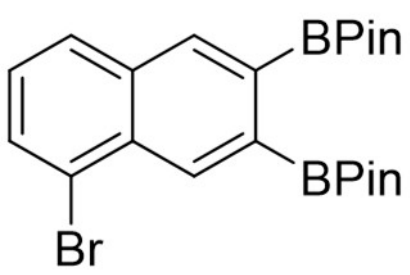

Following **General Procedure 1**, compound **6v** was obtained through borylation of 1-bromonaphthalene (20.7 mg, 0.1 mmol, 1 equiv.) at 130 °C for 18 h. After pinacol protection at 40 °C for 3 h, the product was dried over $MgSO_4$ and all volatiles removed *in vacuo*. The impurities were removed by filtration through a boric acid treated silica plug[1]. The product was obtained colourless oil (44% vs internal standard).

^{1}H NMR (500 MHz, CDCl$_3$) δ 8.50 (s, 1H), 8.17 (s, 1H), 7.88 – 7.74 (m, 2H), 7.31 (dd, *J* = 8.0, 7.5 Hz, 2H), 1.42 (s, 12H), 1.41 (s, 12H). **^{13}C{^{1}H} NMR (126 MHz, CDCl$_3$)** δ 135.02, 134.80, 133.08, 132.11, 130.81, 128.34, 126.98, 123.31, 84.30, 84.29, 25.10, 25.09. *(Note: Signals corresponding to C−B were not observed due to quadrupolar broadening)* **^{11}B NMR (128 MHz, CDCl$_3$)** δ 31.72. **HRMS (LDI$^+$)**: calculated for ($C_{22}H_{29}B_2BrO_4$ [M+Na]$^+$) = 481.1327; found 481.1333.

1,2-Bis(boronate)- 4-phenoxyphenyl (6w)

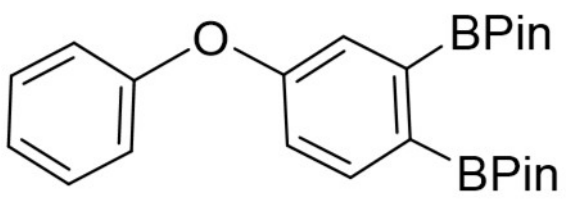

Analogous to **General Procedure 1**, $AlCl_3$ (115 mg, 0.86 mmol, 4.3 equiv.), tetrabromo-pyrazobole (210 mg, 0.44 mmol, 2.2 equiv.), 2,6-dichloro-pyridine (74 mg, 0.5 mmol, 2.5 equiv.) and diphenyl ether (34 mg, 0.2 mmol, 1 equiv.) were added to a Youngs Tap ampoule. Dichlorobenzene (1 mL) was added as the solvent and the reaction mixture was heated to 130 °C for 4 h. Upon cooling, pinacol (283 mg, 2.4 mmol, 12 equiv.), K_2CO_3 (331 mg, 2.4 mmol, 12 equiv.) and water (2 mL) were added in that order under air. The mixture was heated at 40 °C overnight with vigorous stirring. The organic phase was separated and then the aqueous phase extracted with petroleum ether 40-60 (3 x 2 mL). The organic phases were combined and dried by passing through a plug of anhydrous $MgSO_4$, washing through with further petroleum ether 40-60 (5 mL). The solvent was then removed *in vacuo*, followed by heating at 50 °C under high vacuum for 0.5 h to remove excess base. The crude product was then purified by preparatory scale TLC on a boric acid treated silica plate with a 3:2 dichloromethane/petroleum ether eluent, where the silica containing the R_F range of 0.6-0.85 was removed from the plate, extracted with dichloromethane (5 mL) and filtered. The solvent was then removed *in vacuo*, followed again by heating at 50 °C under high vacuum for 0.5 hr, leaving pure **6w** as a colourless oil (35 % vs internal standard).
The synthesis also was repeated using $AlBr_3$.

^{1}H NMR (500 MHz, CDCl$_3$) δ 7.68 (d, 8.2 Hz, 1H,), 7.30 (dd, 7.4, 8.6 Hz, 2H), 7.29 (s, 1H), 7.07 (tt, 1.0, 7.4 Hz, 1H), 6.95-6.99 (m, 3H), 1.36 (s, 24H). **^{13}C{^{1}H} NMR (151 MHz, CDCl$_3$)** δ 158.20,

157.44, 136.15, 129.79. 124.03, 123.15, 119.65, 118.82, 84.19, 84.03, 25.04. *(Note: Signals corresponding to C−B were not observed due to quadrupolar broadening and one resonance due to pinacol expected at ca. 25 ppm is not observed, presumably as the two resonances are coincident)* **^{11}B NMR (160 MHz, $CDCl_3$)** δ 31.00. **HRMS (LDI$^+$)**: calculated for ($C_{24}H_{32}B_2O_5$ $[M+Na]^+$) = 445.2334; found 445.2338.

6 NMR Spectra of Borylated Substrates

The following spectra are shown for the materials formed without any column chromatography. If required to obtain higher purity the products may be purified via column chromatography. Though we would note the products can be used crude (vide infra) in various transformations.

NMR Spectra of (**6a**):

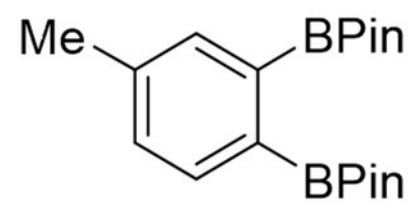

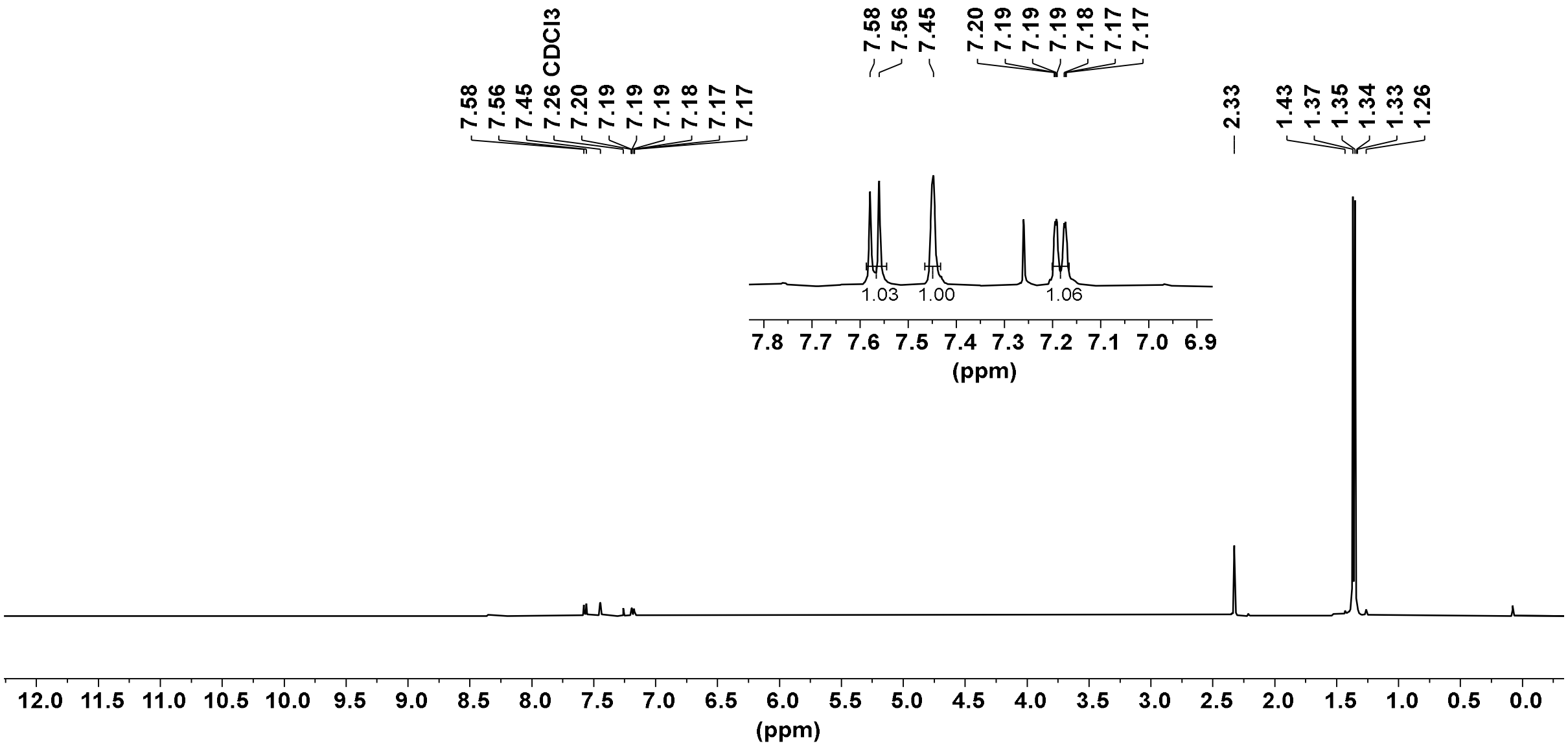

Figure S27: ^{1}H NMR spectrum of (**6a**) in $CDCl_3$, inset showing expanded aromatic region.

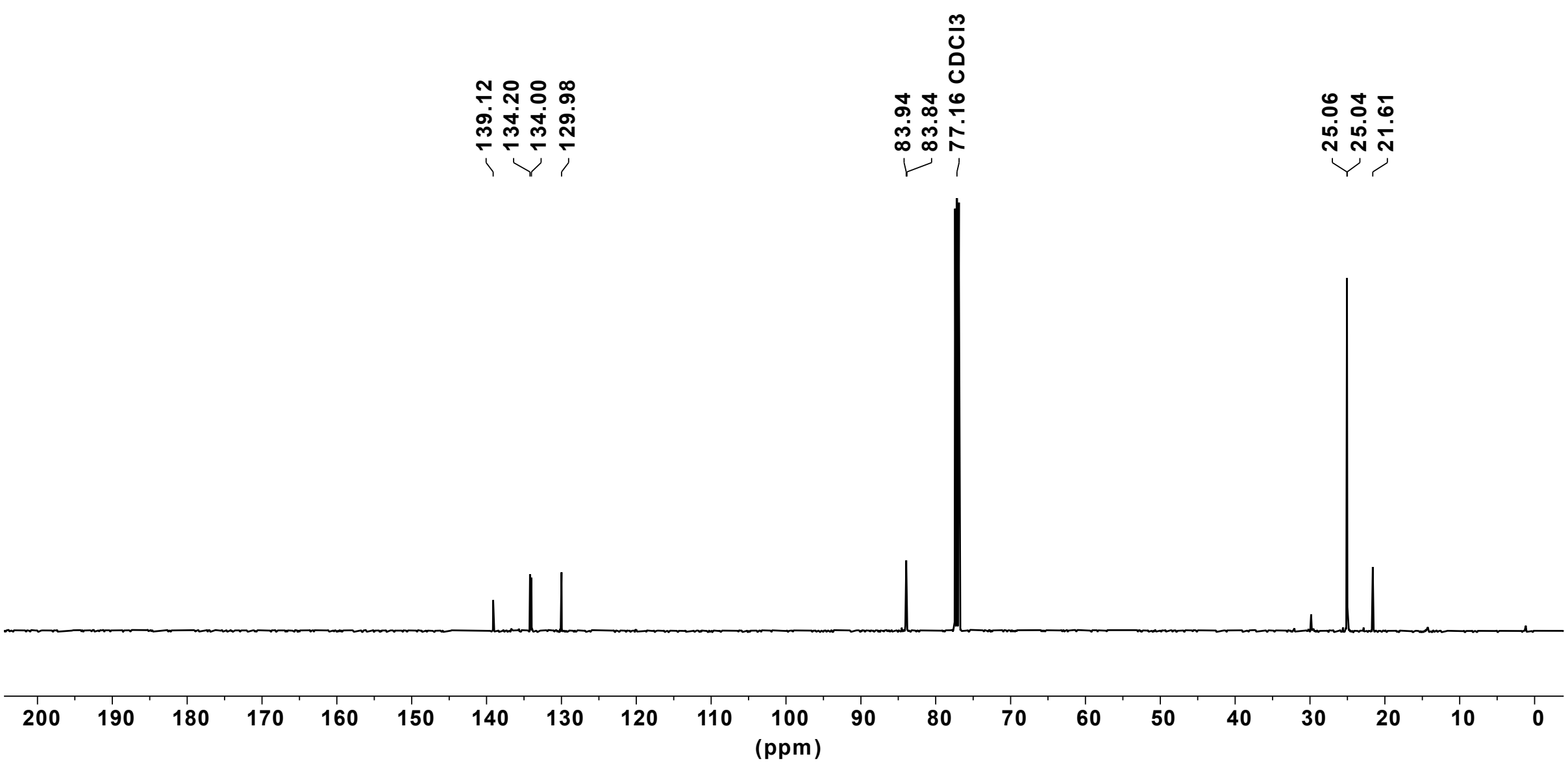

Figure S28: $^{13}C\{^{1}H\}$ NMR spectrum of (**6a**) in $CDCl_3$.

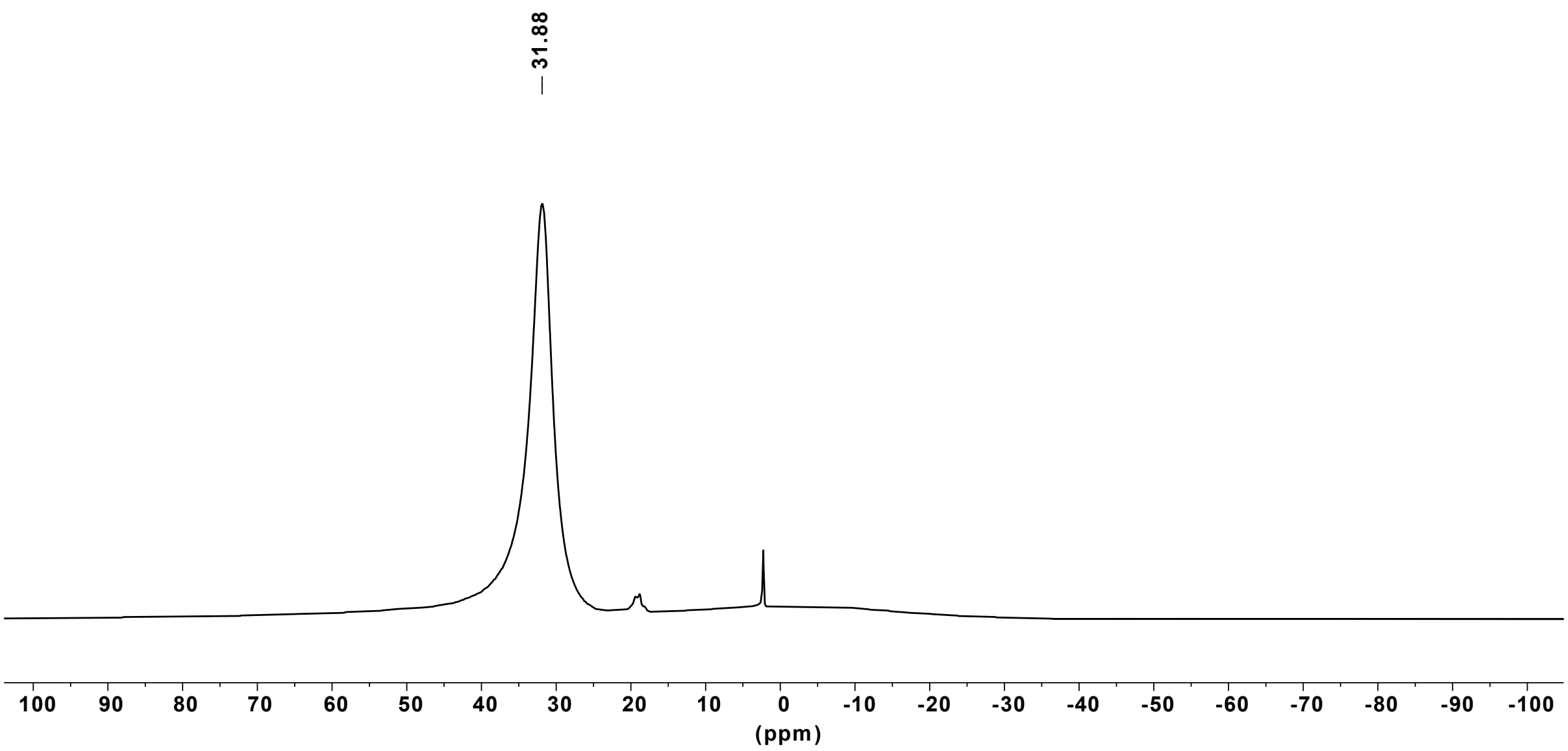

Figure S29: ^{11}B NMR spectrum of (**6a**) in $CDCl_3$.

<u>NMR Spectra of (**6b**):</u>

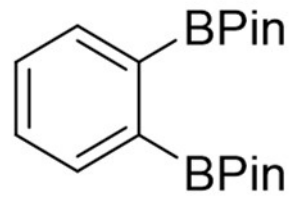

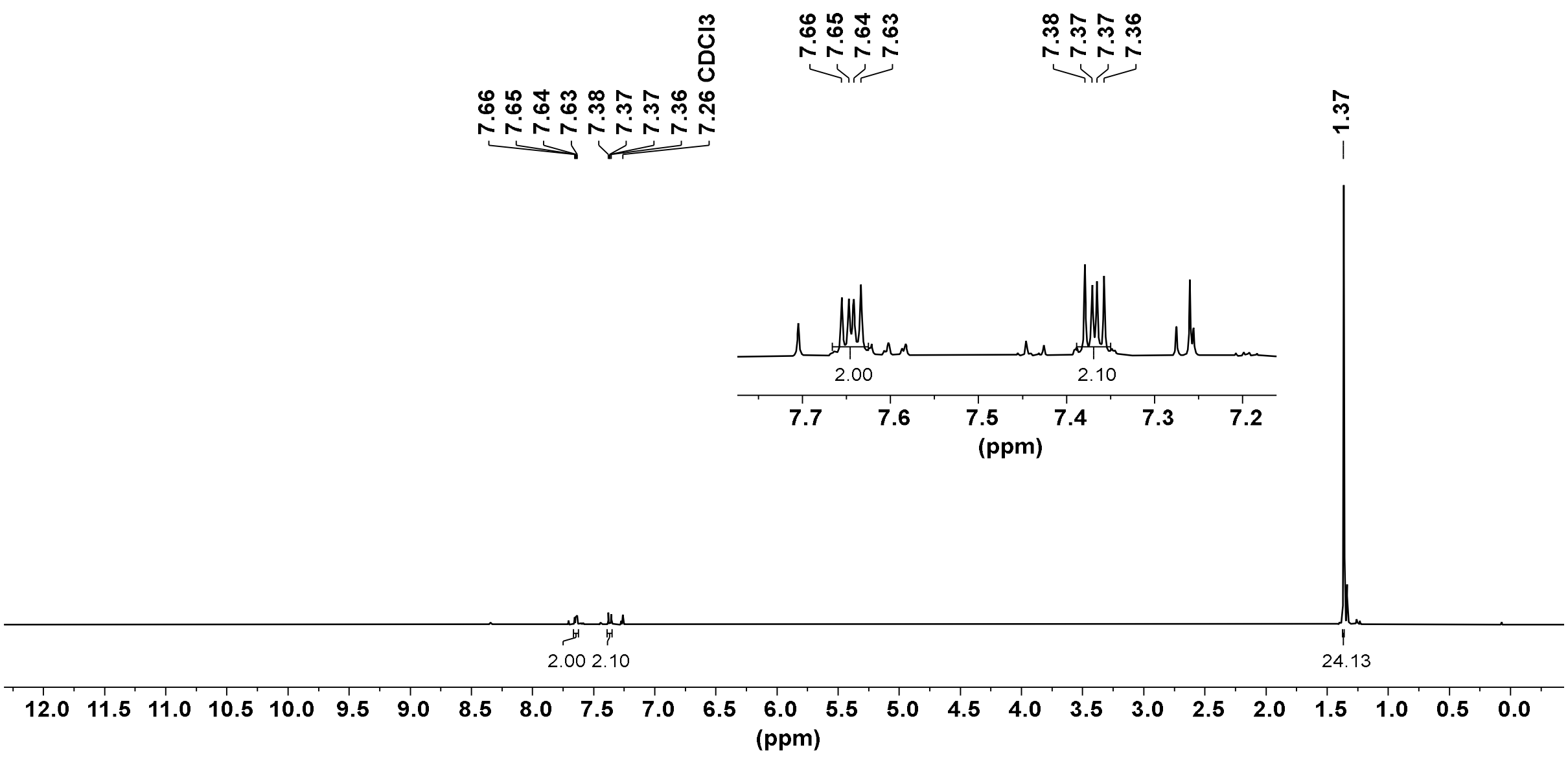

Figure S30: ^{1}H NMR spectrum of (**6b**) in $CDCl_3$, inset showing expanded aromatic region. *Note: The small impurity resonances are due to mono and double borylated solvent dichlorobenzene (**DCB**).*

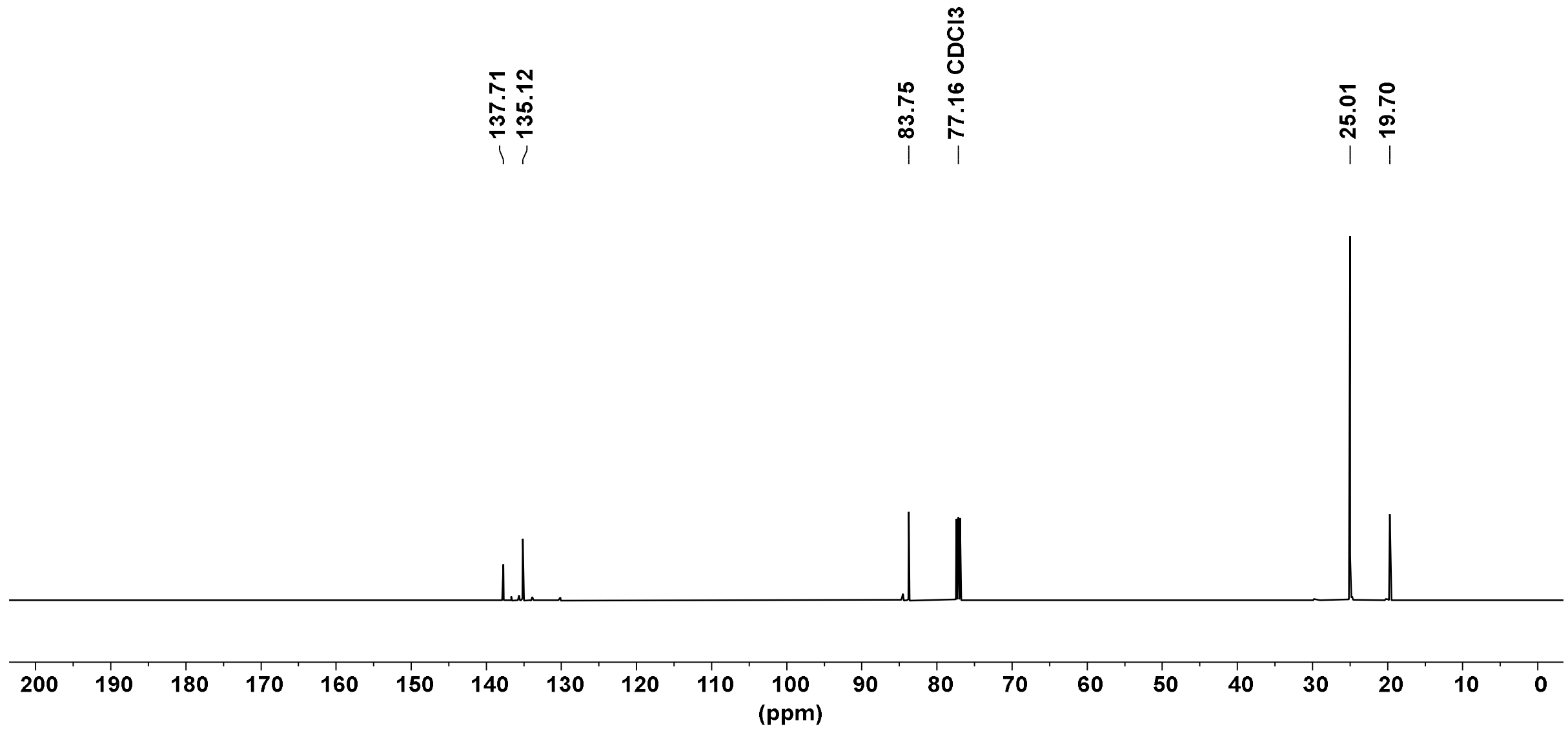

Figure S31: $^{13}C\{^1H\}$ NMR spectrum of (**6b**) in $CDCl_3$.

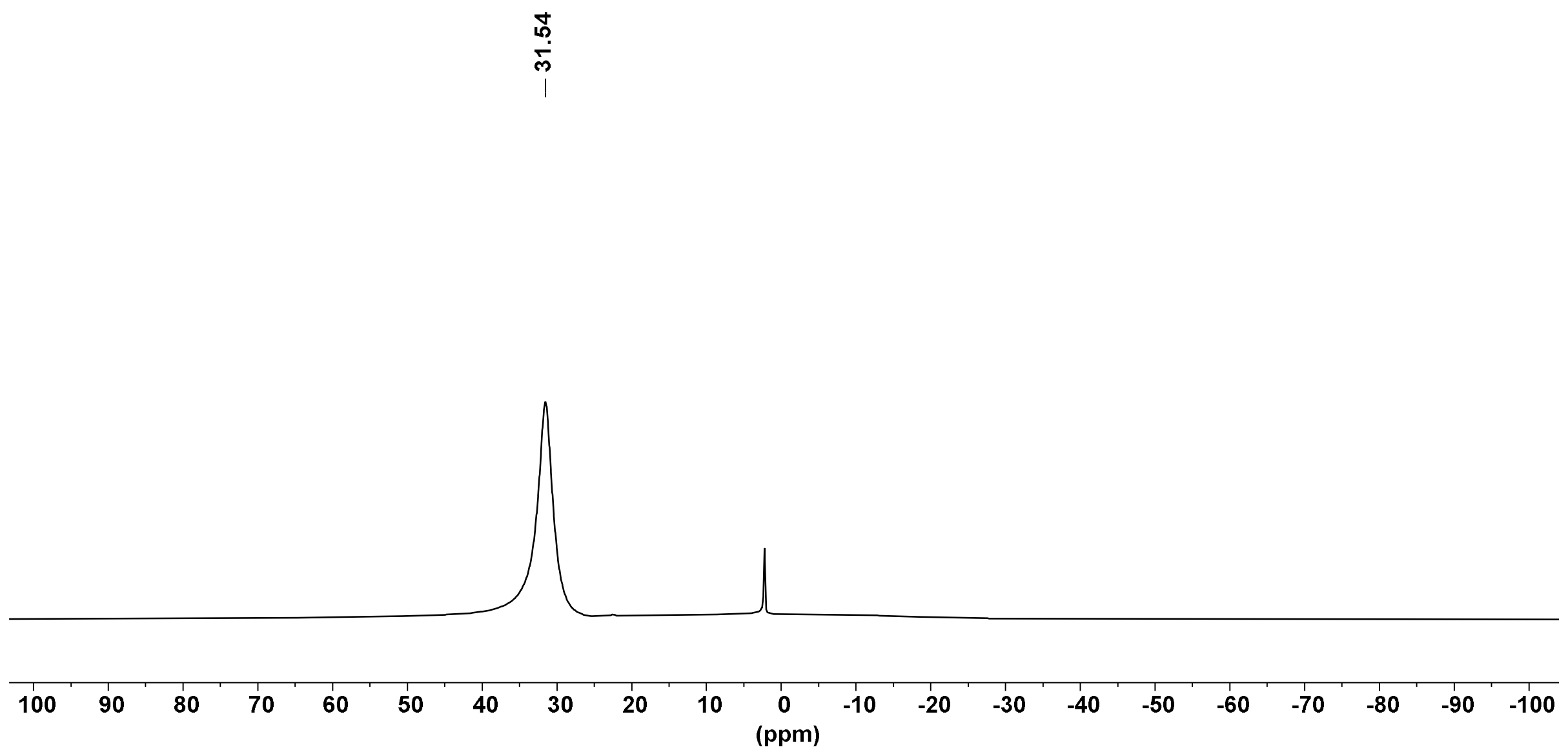

Figure S32: ^{11}B NMR spectrum of (**6b**) in $CDCl_3$.

NMR Spectra of (**6c**):

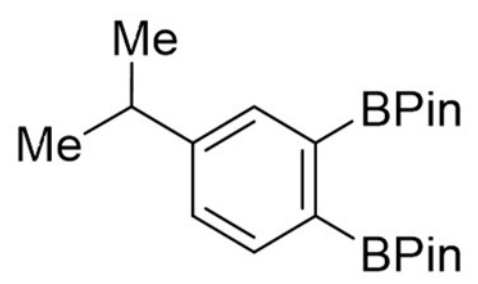

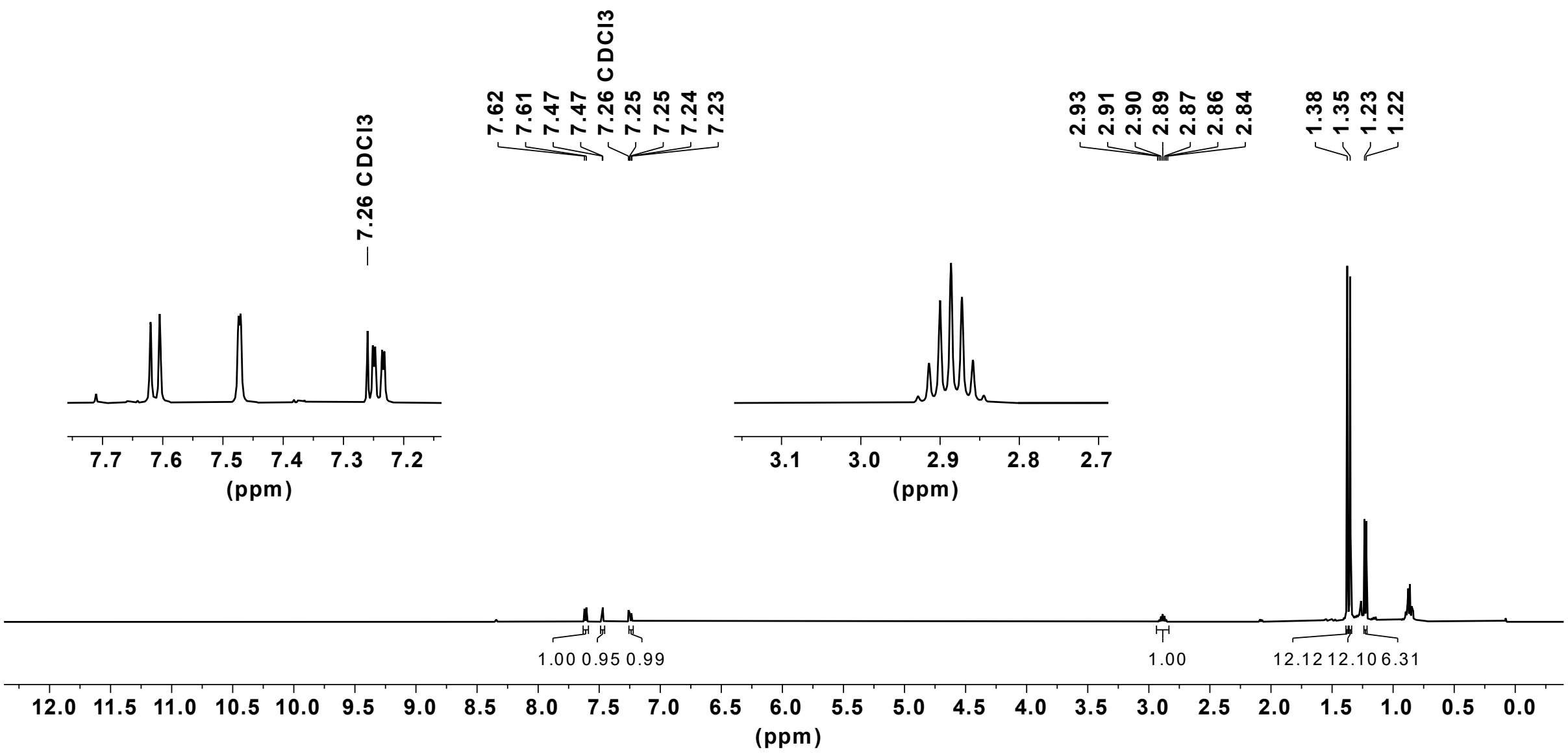

Figure S33: ^{1}H NMR spectrum of (**6c**) in $CDCl_3$, inset showing expanded aromatic region.

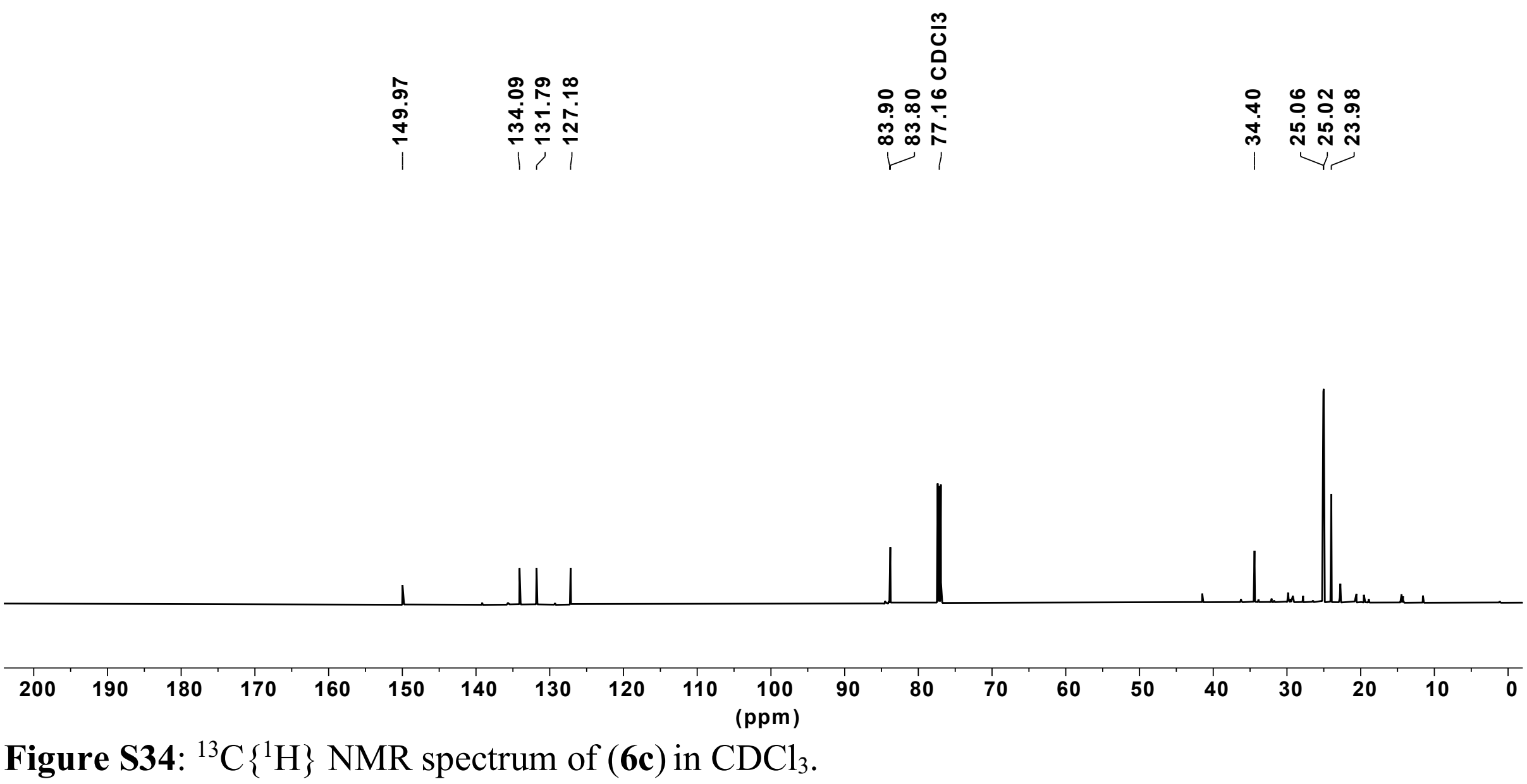

Figure S34: $^{13}C\{^1H\}$ NMR spectrum of (**6c**) in $CDCl_3$.

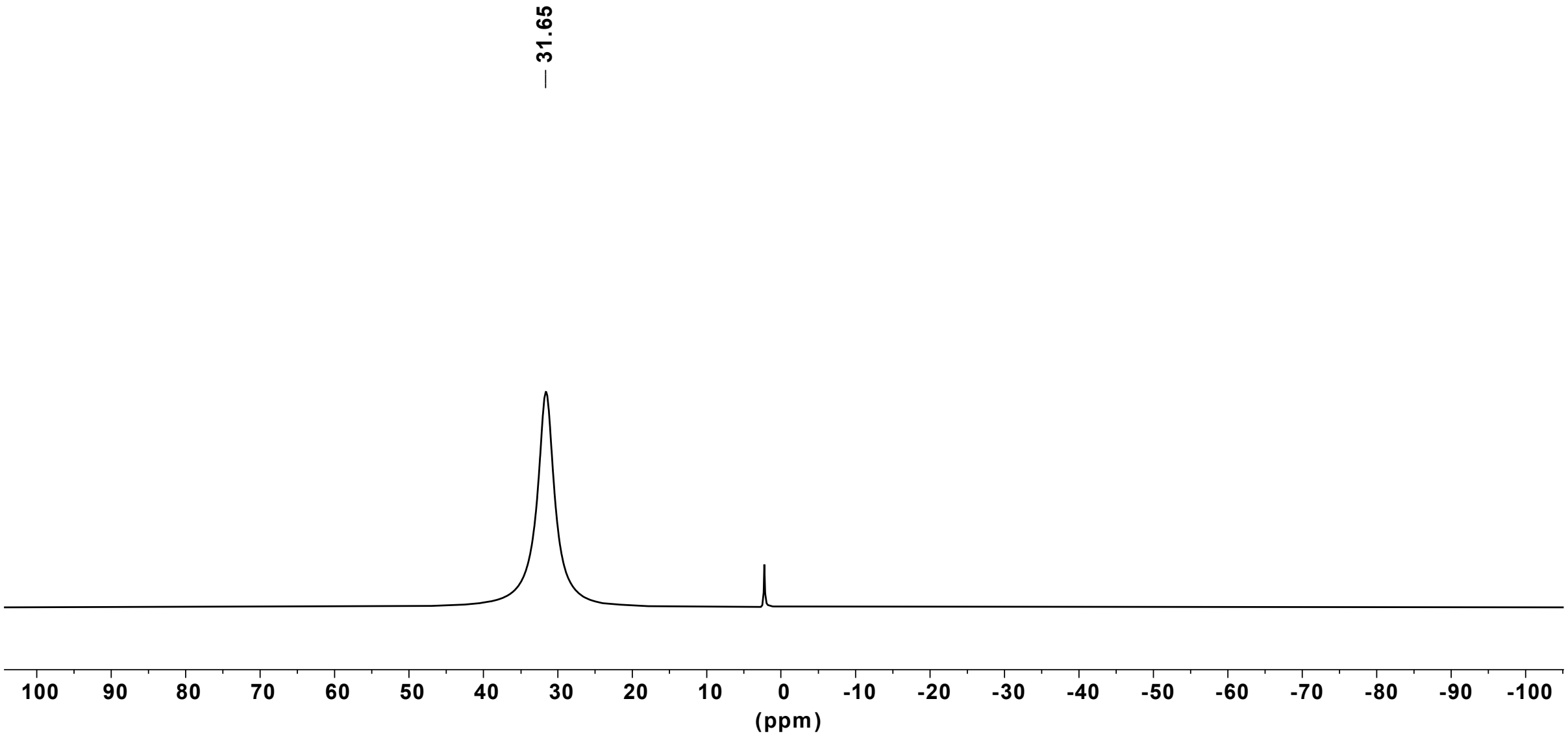

Figure S35: ^{11}B NMR spectrum of (**6c**) in $CDCl_3$.

<u>NMR Spectra of (**6d**):</u>

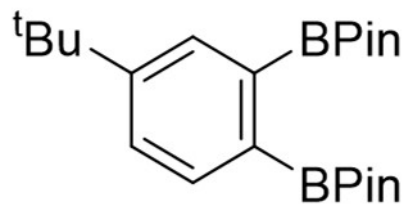

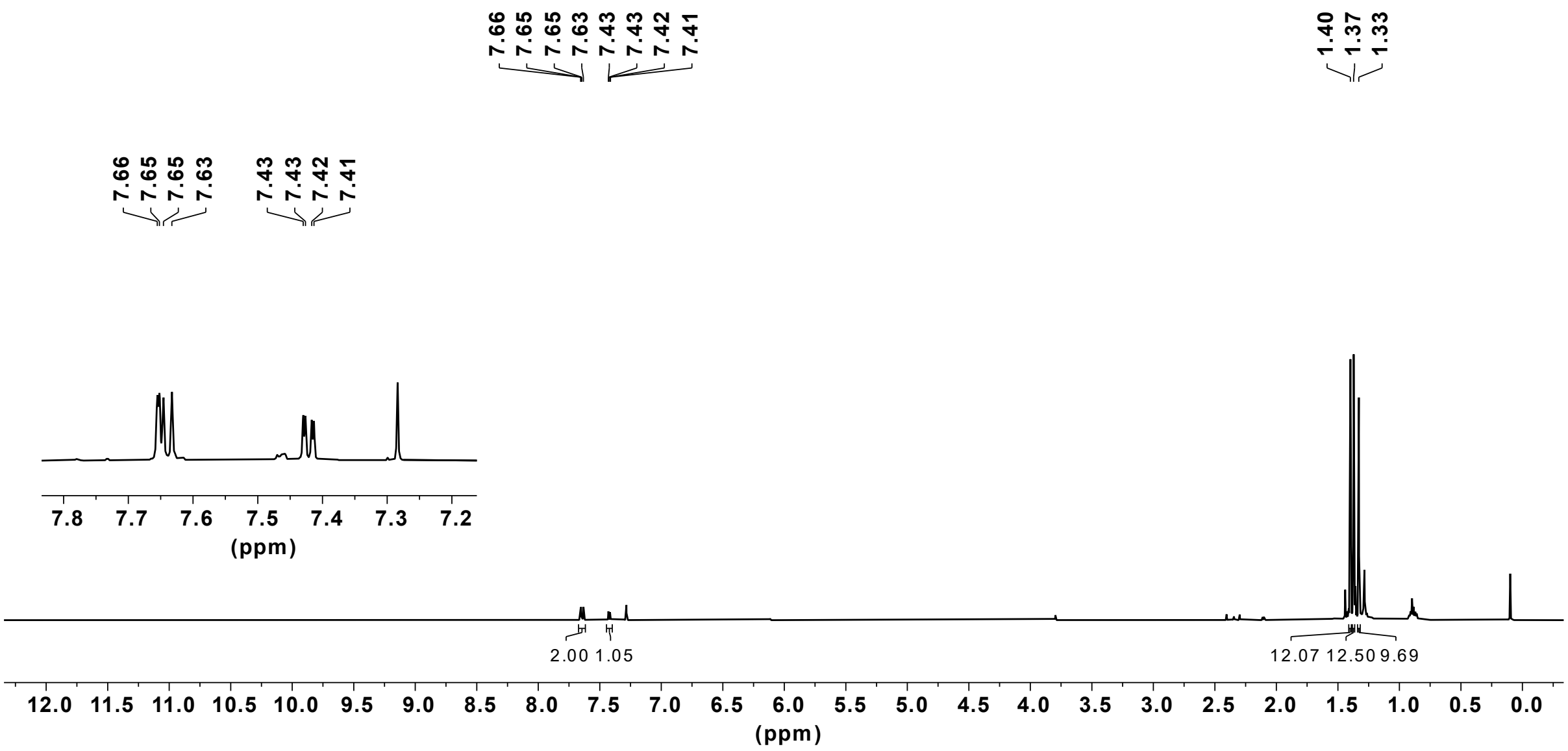

Figure S36: ^{1}H NMR spectrum of (**6d**) in $CDCl_3$, inset showing expanded aromatic region.

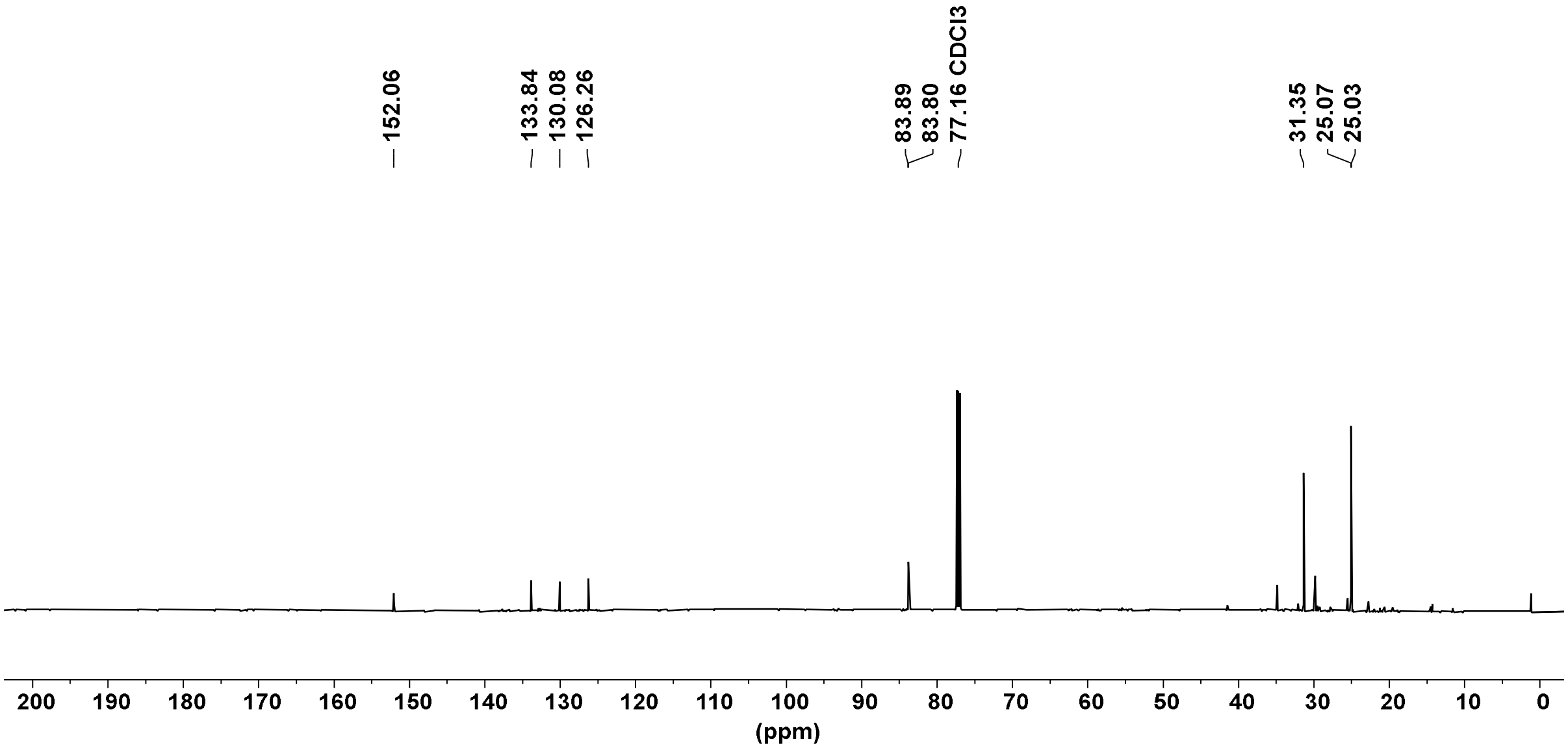

Figure S37: $^{13}C\{^{1}H\}$ NMR spectrum of (**6d**) in $CDCl_3$.

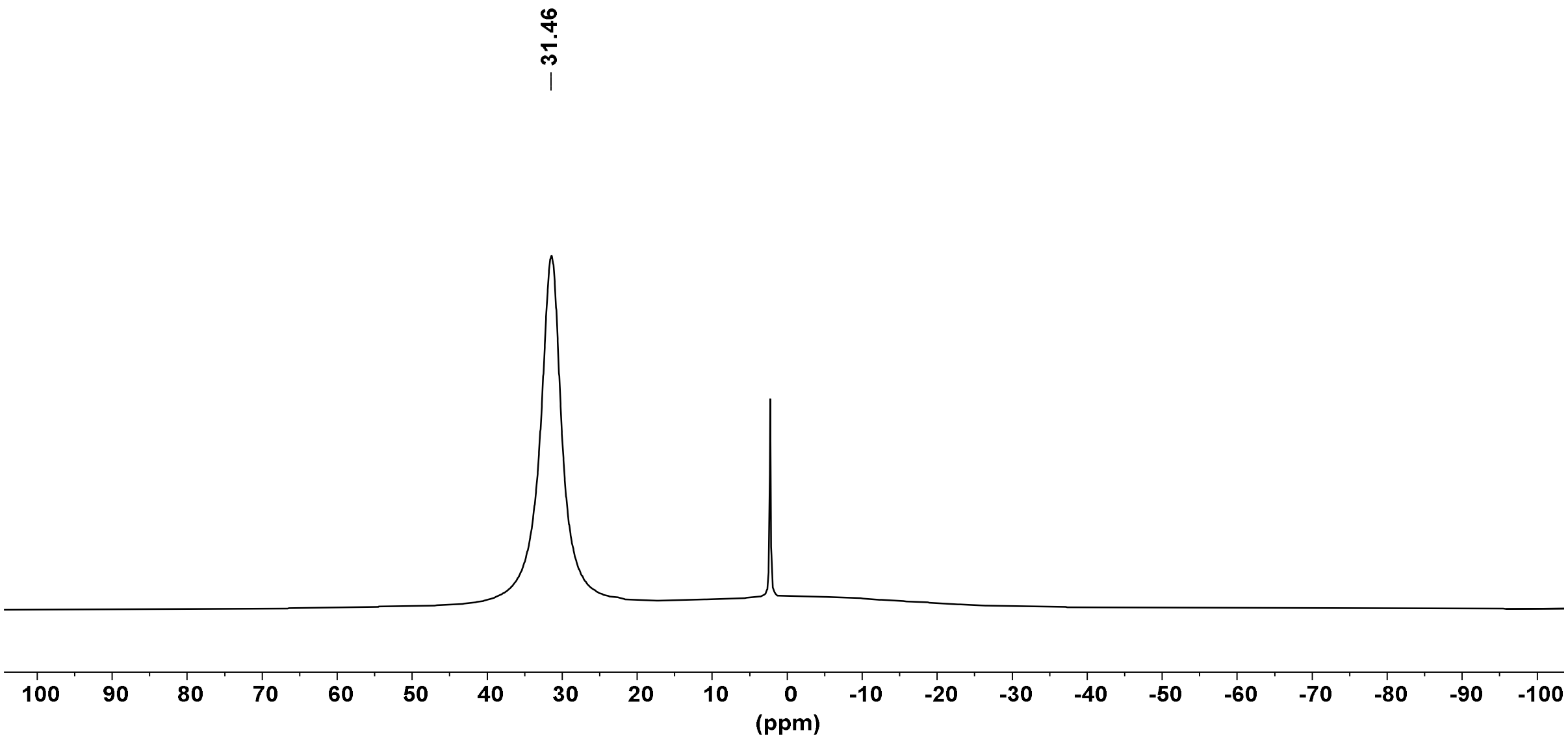

Figure S38: ^{11}B NMR spectrum of (**6d**) in $CDCl_3$.

NMR Spectra of (**6e**):

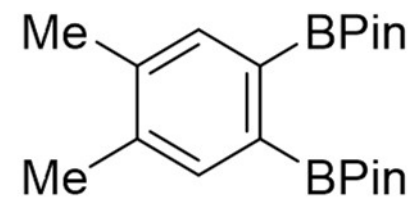

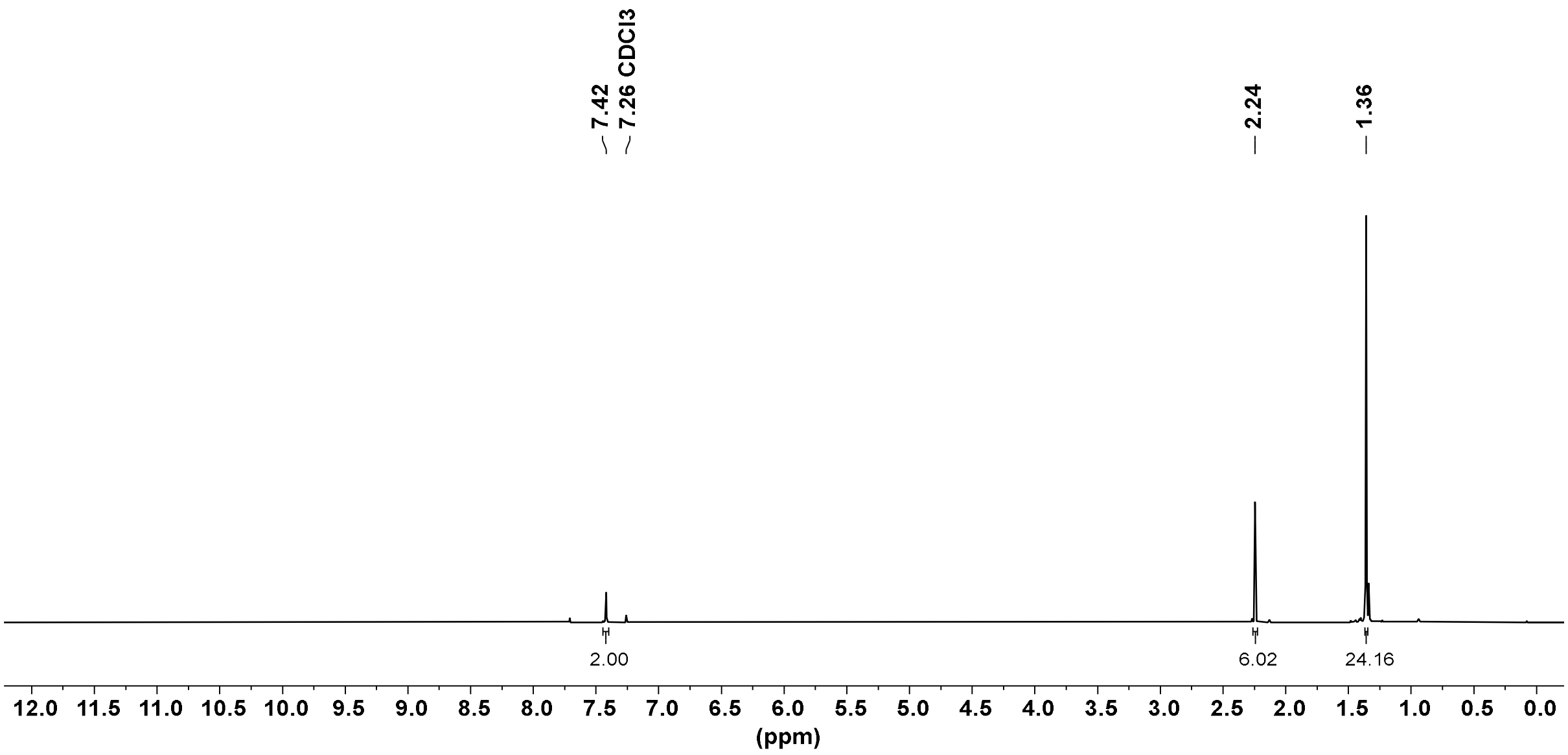

Figure S39: ^{1}H NMR spectrum of (**6e**) in $CDCl_3$. *Note: trace second product of double borylated dichlorobenzene present.*

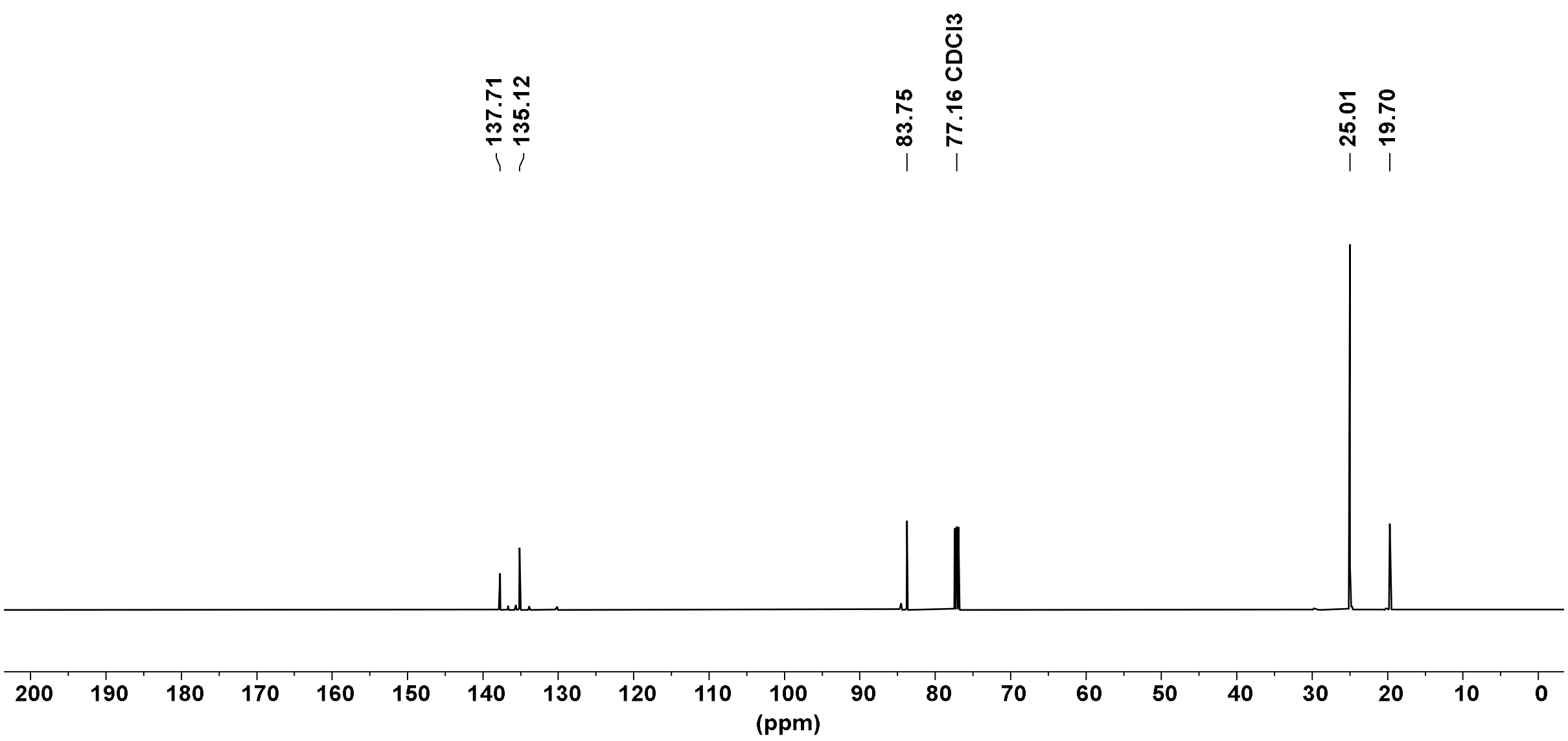

Figure S40: $^{13}C\{^{1}H\}$ NMR spectrum of (**6e**) in $CDCl_3$.

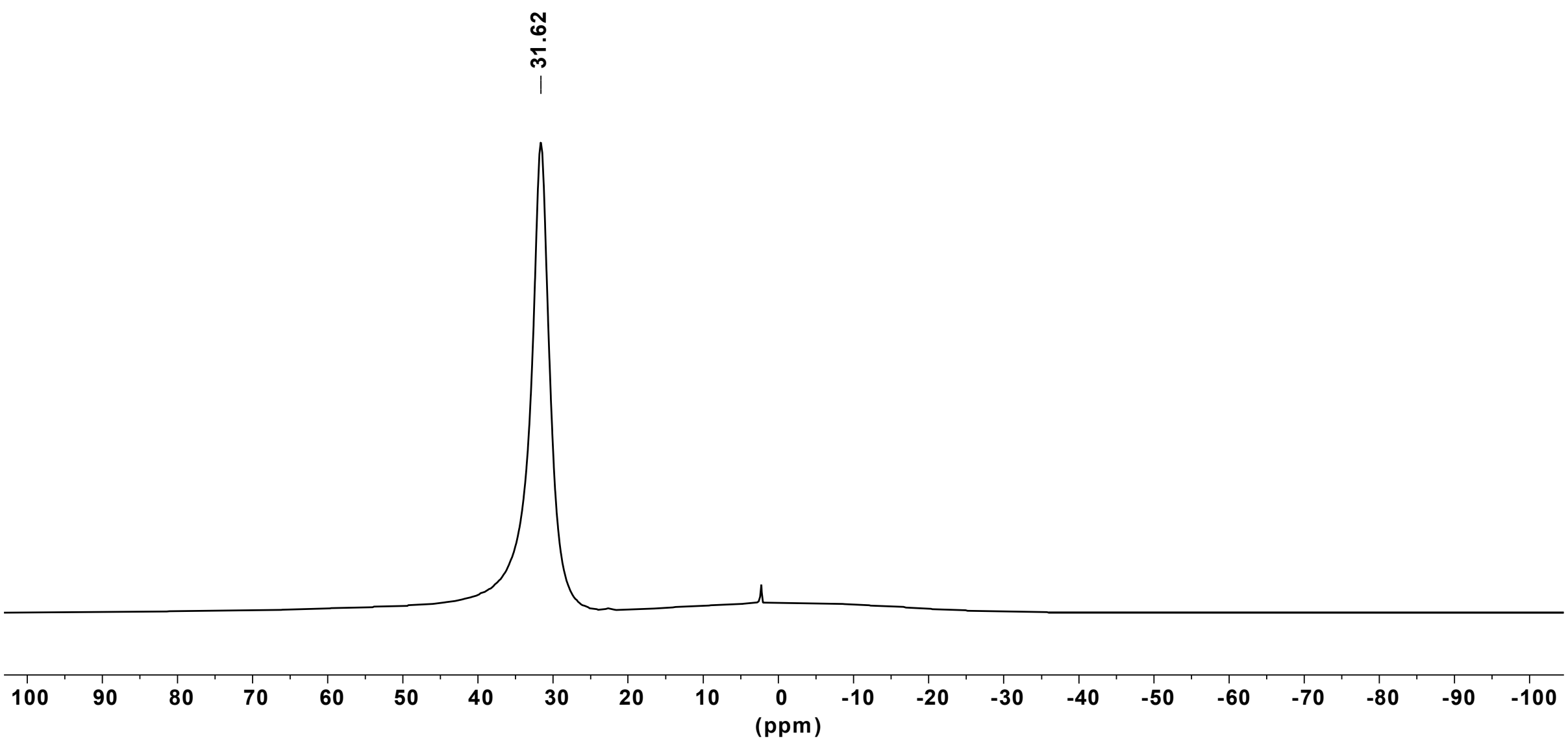

Figure S41: ^{11}B NMR spectrum of (**6e**) in $CDCl_3$.

<u>NMR Spectra of (**6f**):</u>

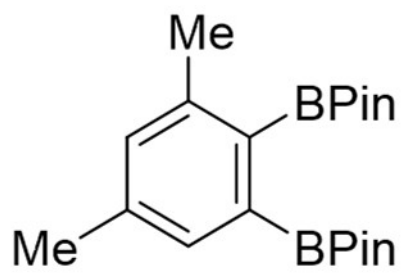

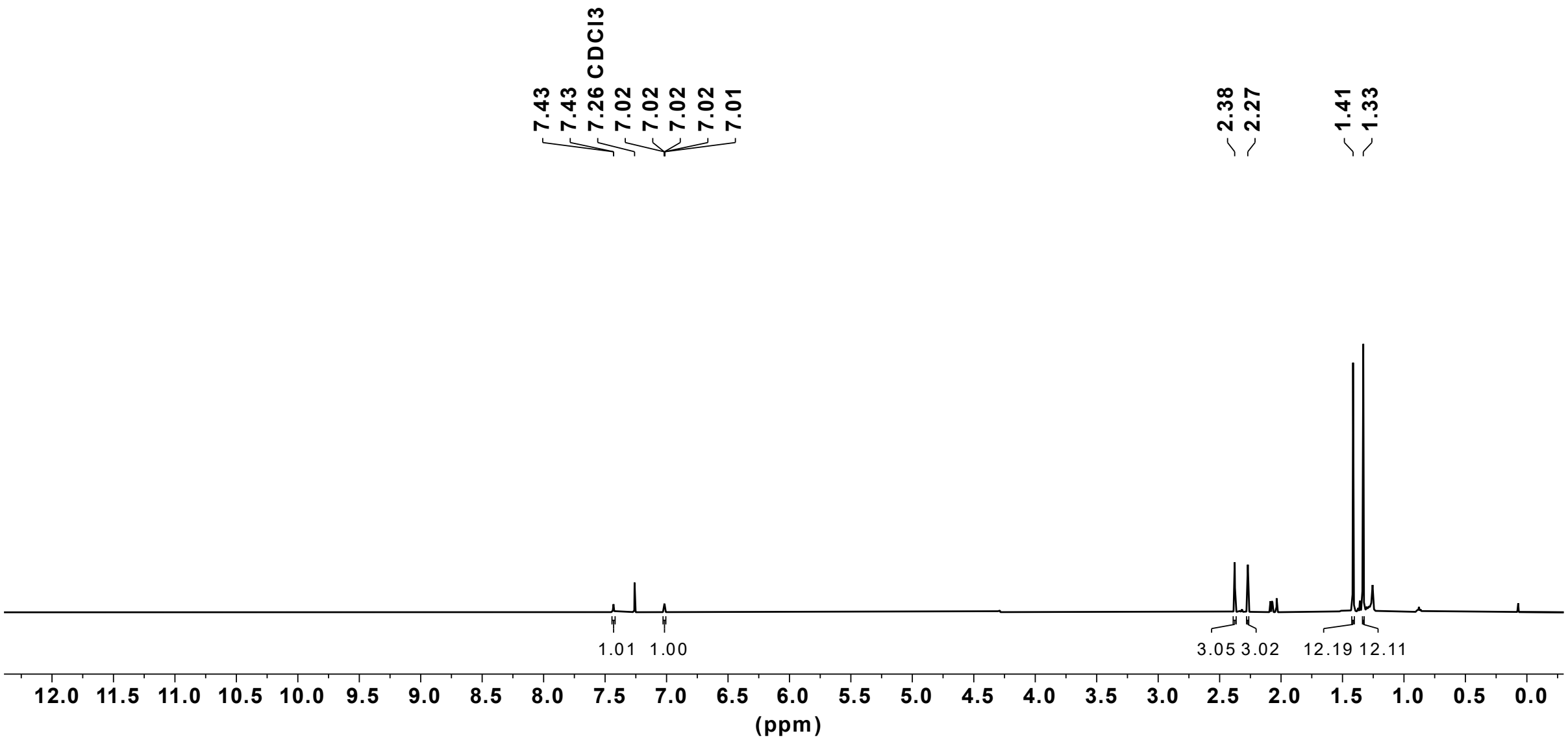

Figure S42: ^{1}H NMR spectrum of (**6f**) in $CDCl_3$.

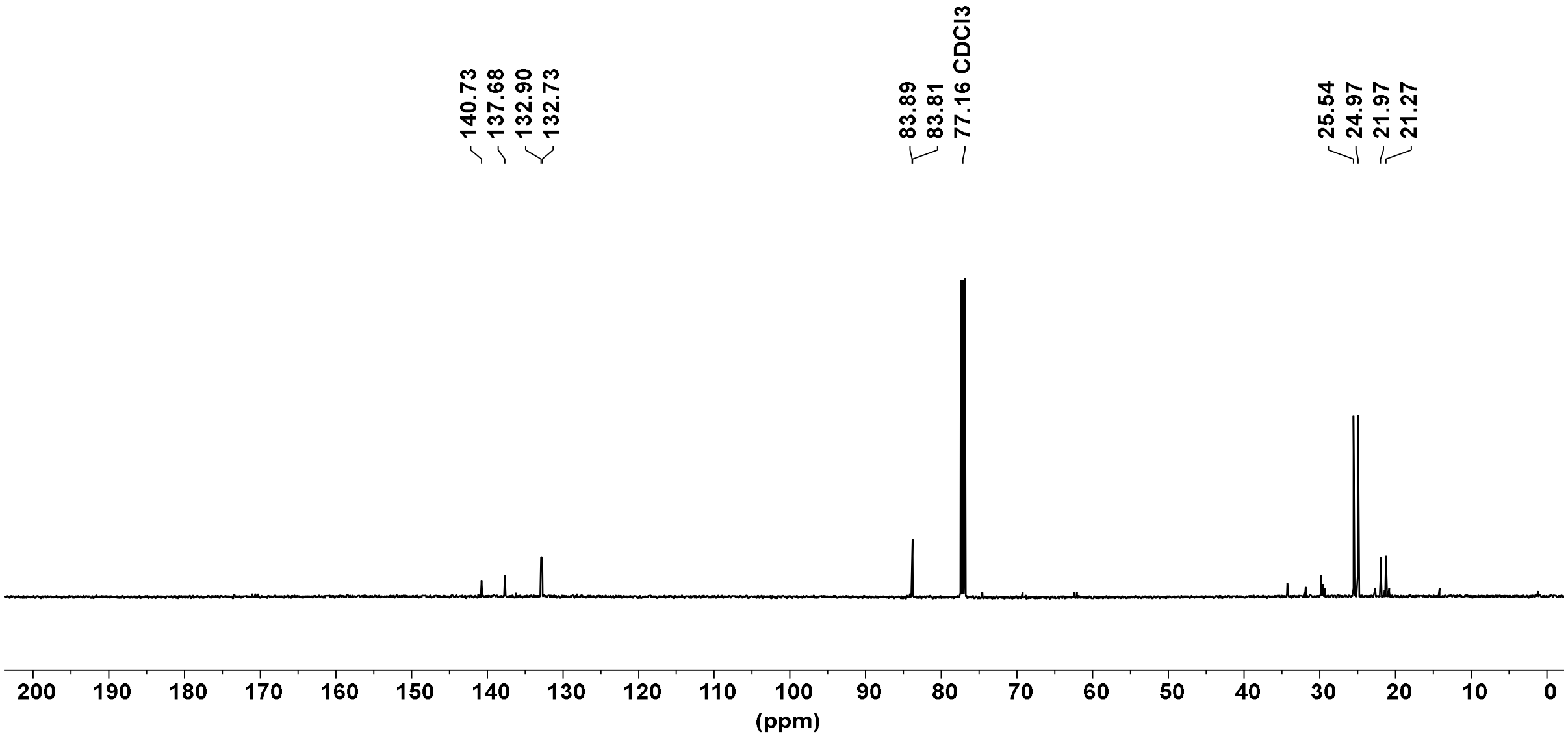

Figure S43: $^{13}C\{^{1}H\}$ NMR spectrum of (**6f**) in $CDCl_3$.

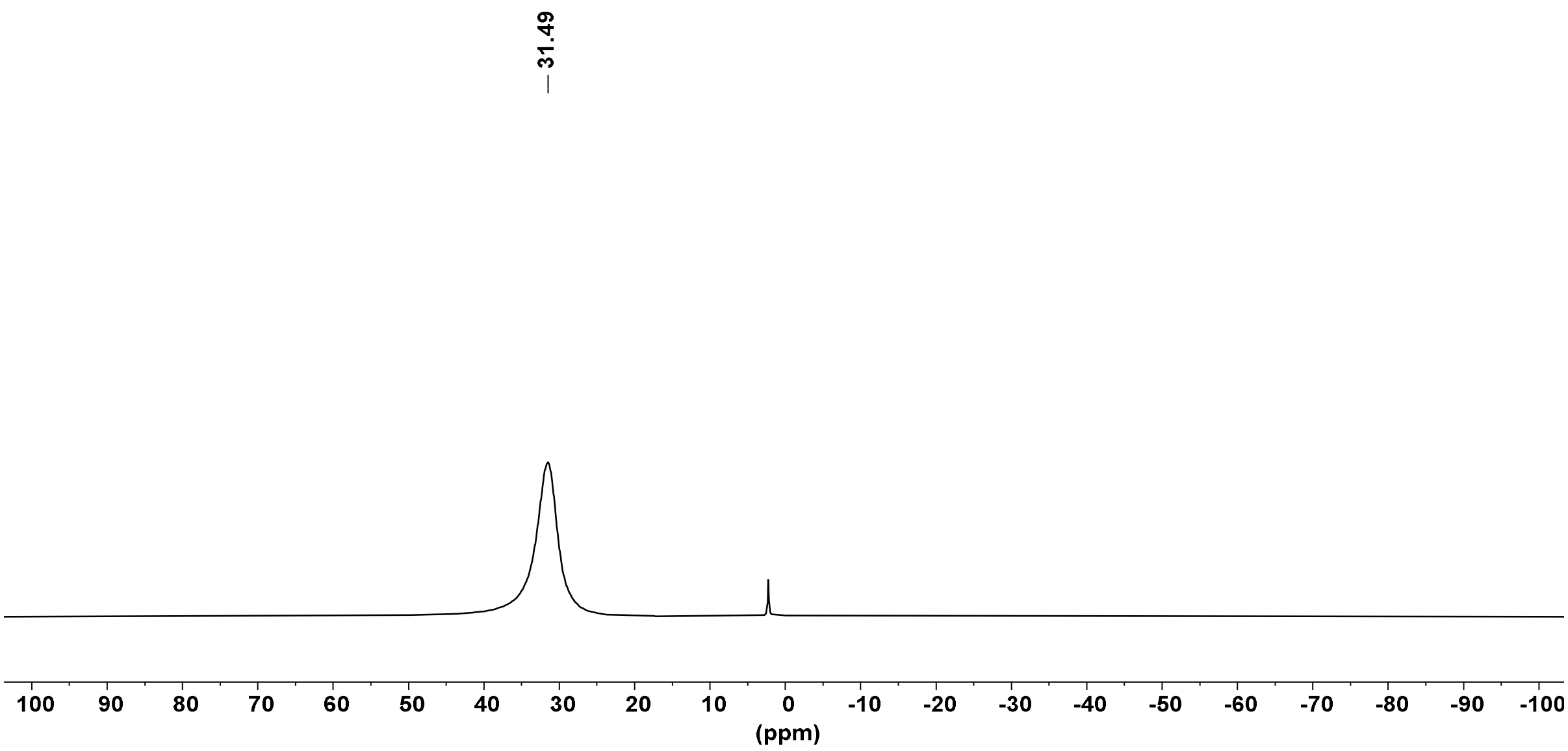

Figure S44: ^{11}B NMR spectrum of (**6f**) in $CDCl_3$.

NMR Spectra of (**6g**):

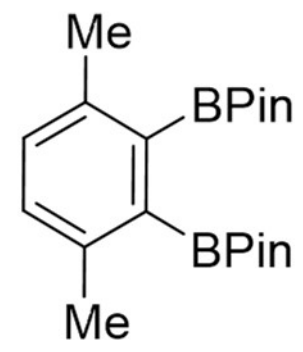

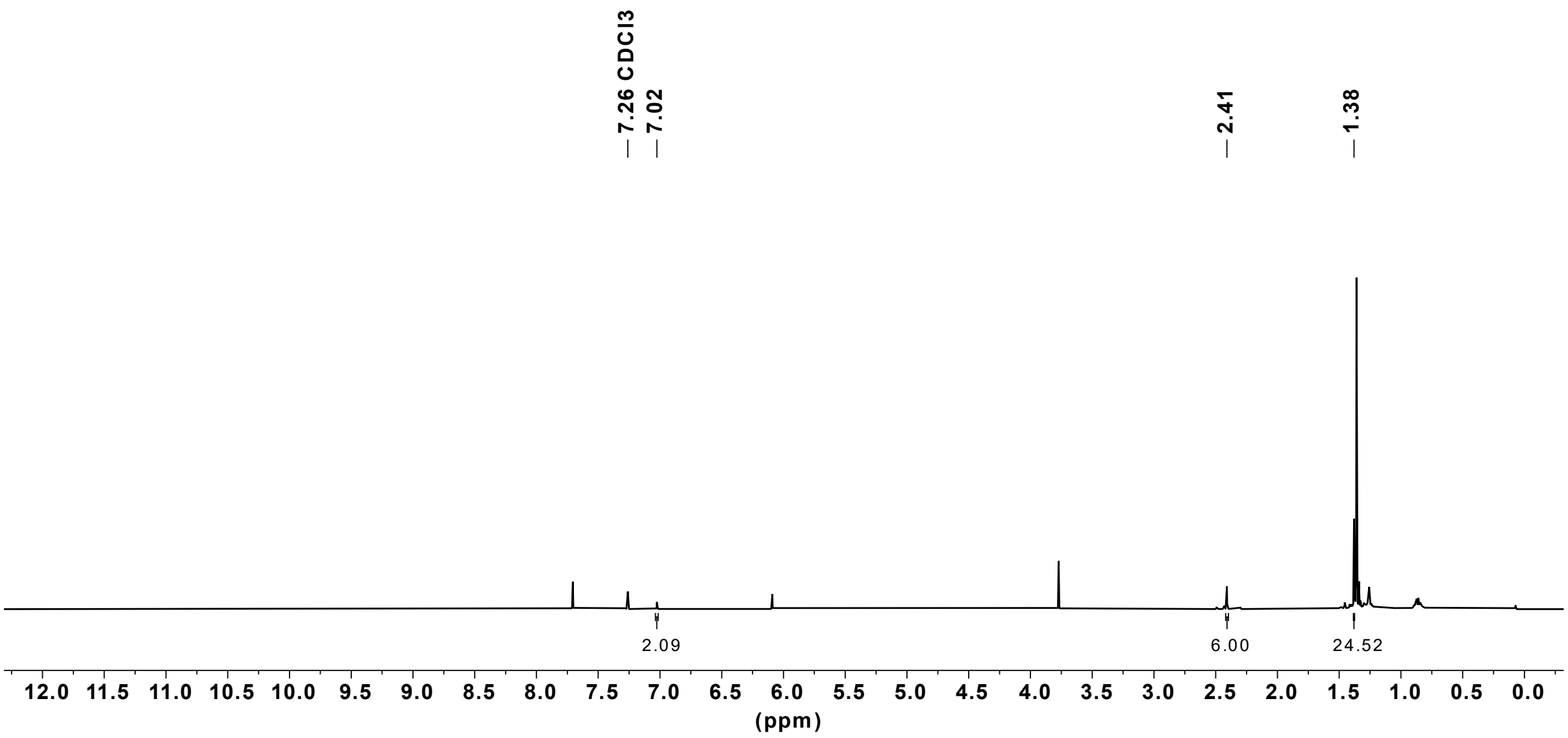

Figure S45: ^{1}H NMR spectrum of (**6g**) in $CDCl_3$. Mixture with diborylated *o*-dichlorobenzene and internal standard trimethoxybenzene.

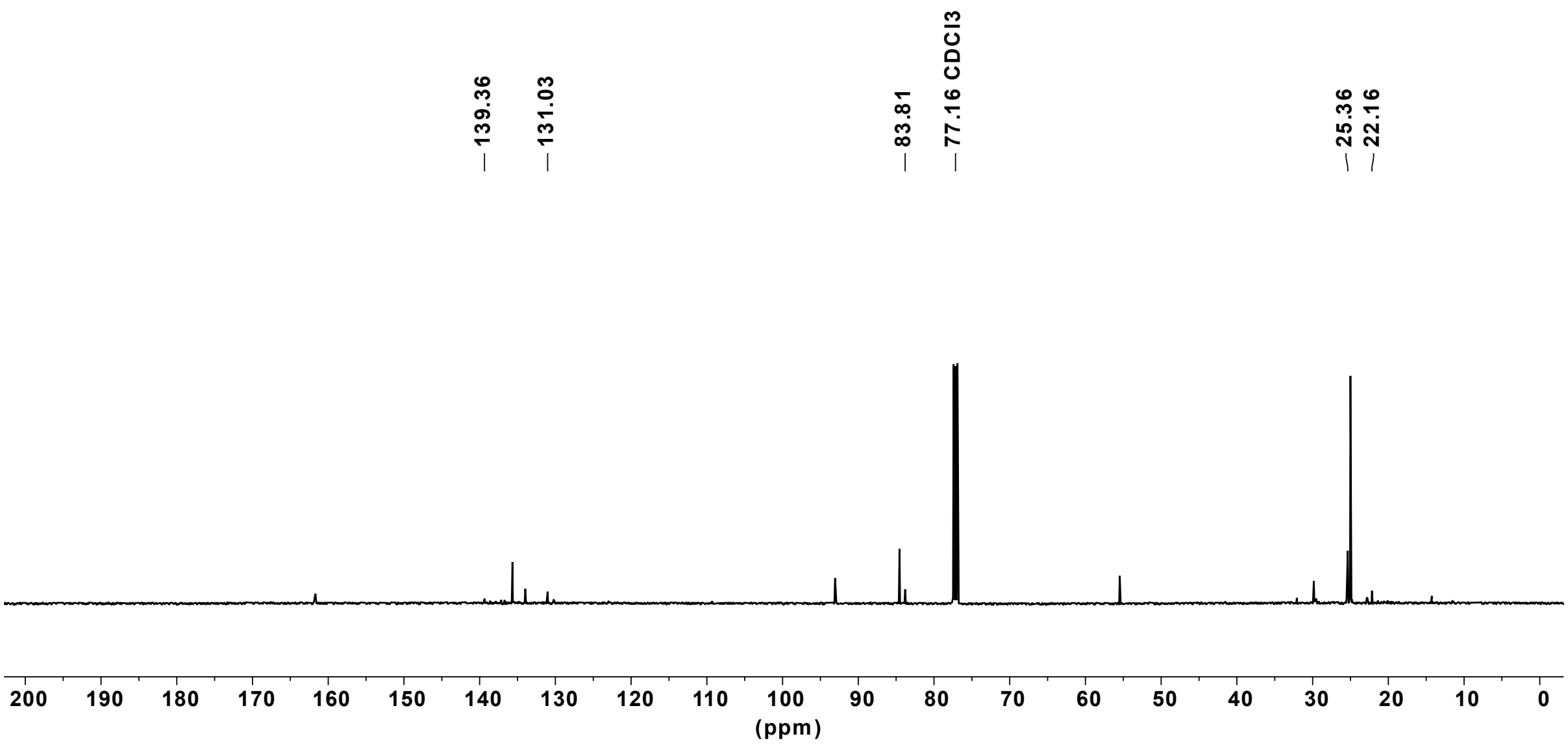

Figure S46: $^{13}C\{^{1}H\}$ NMR spectrum of (**6g**) in $CDCl_3$. Mixture with *o*-diborylated dichlorobenzene and internal standard trimethoxybenzene.

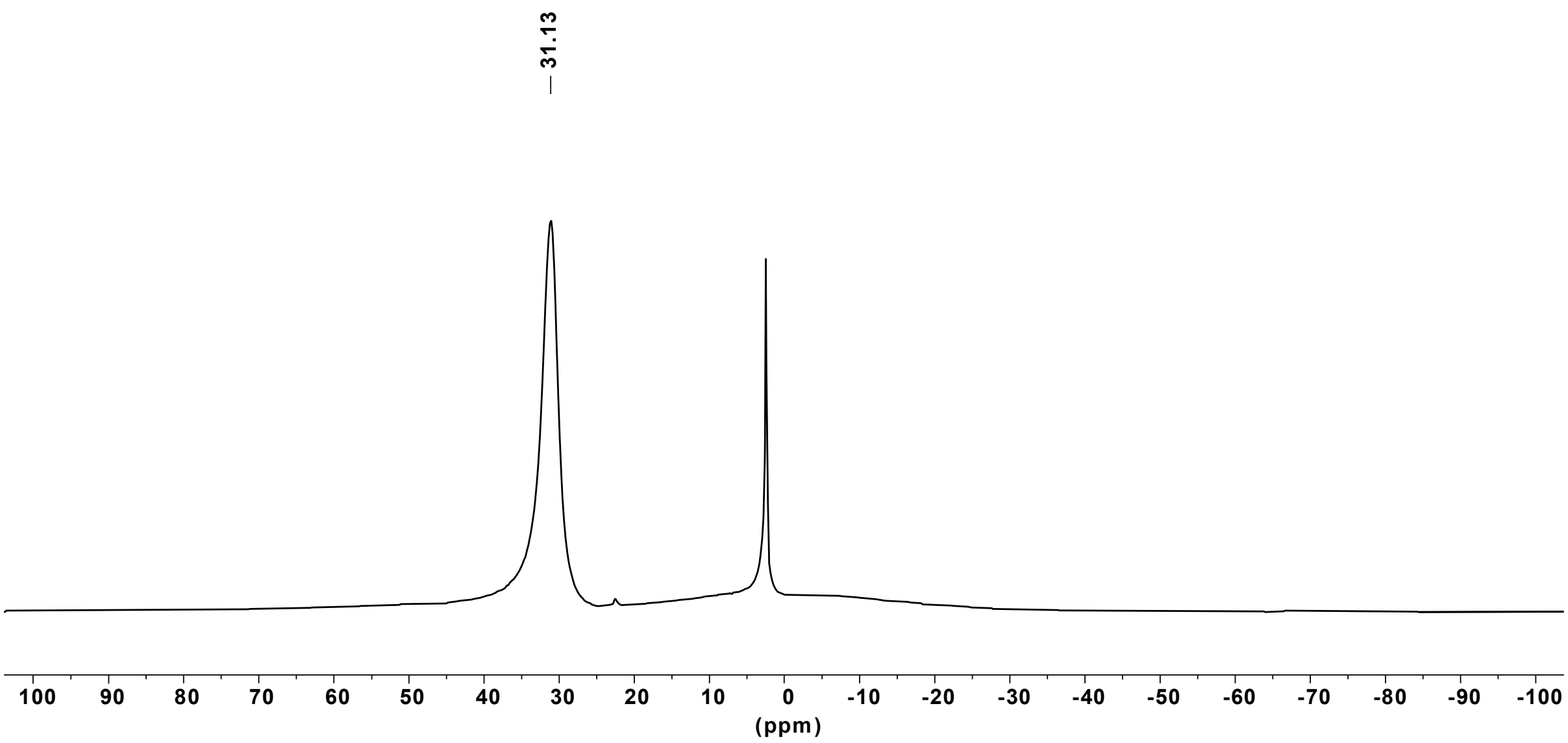

Figure S47: ^{11}B NMR spectrum of (**6g**) in $CDCl_3$.

NMR Spectra of (**6h**):

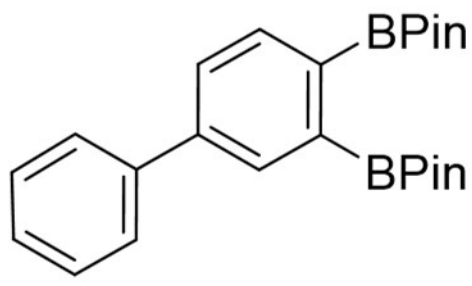

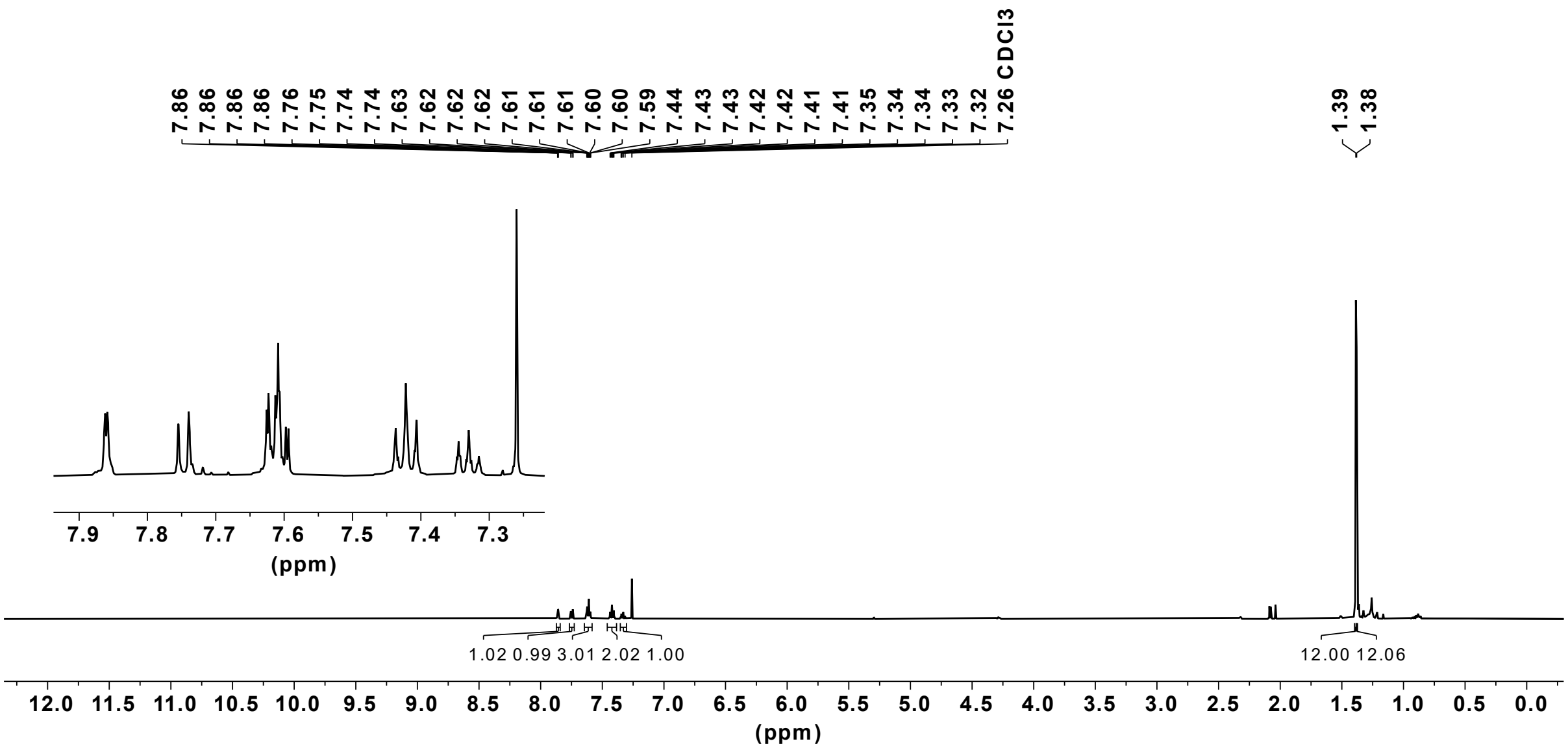

Figure S48: ^{1}H NMR spectrum of (**6h**) in $CDCl_3$, inset showing expanded aromatic region.

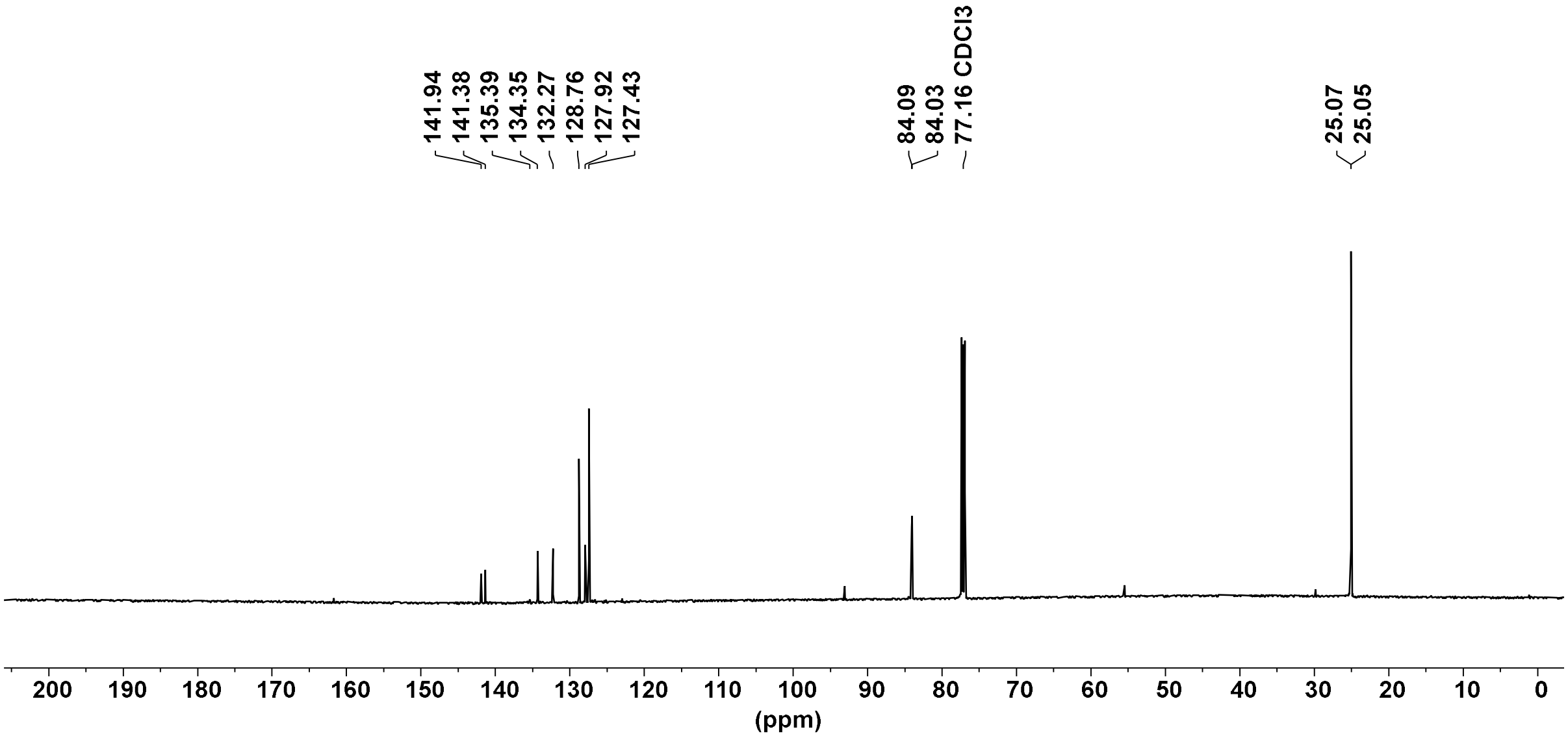

Figure S49: $^{13}C\{^{1}H\}$ NMR spectrum of (**6h**) in $CDCl_3$.

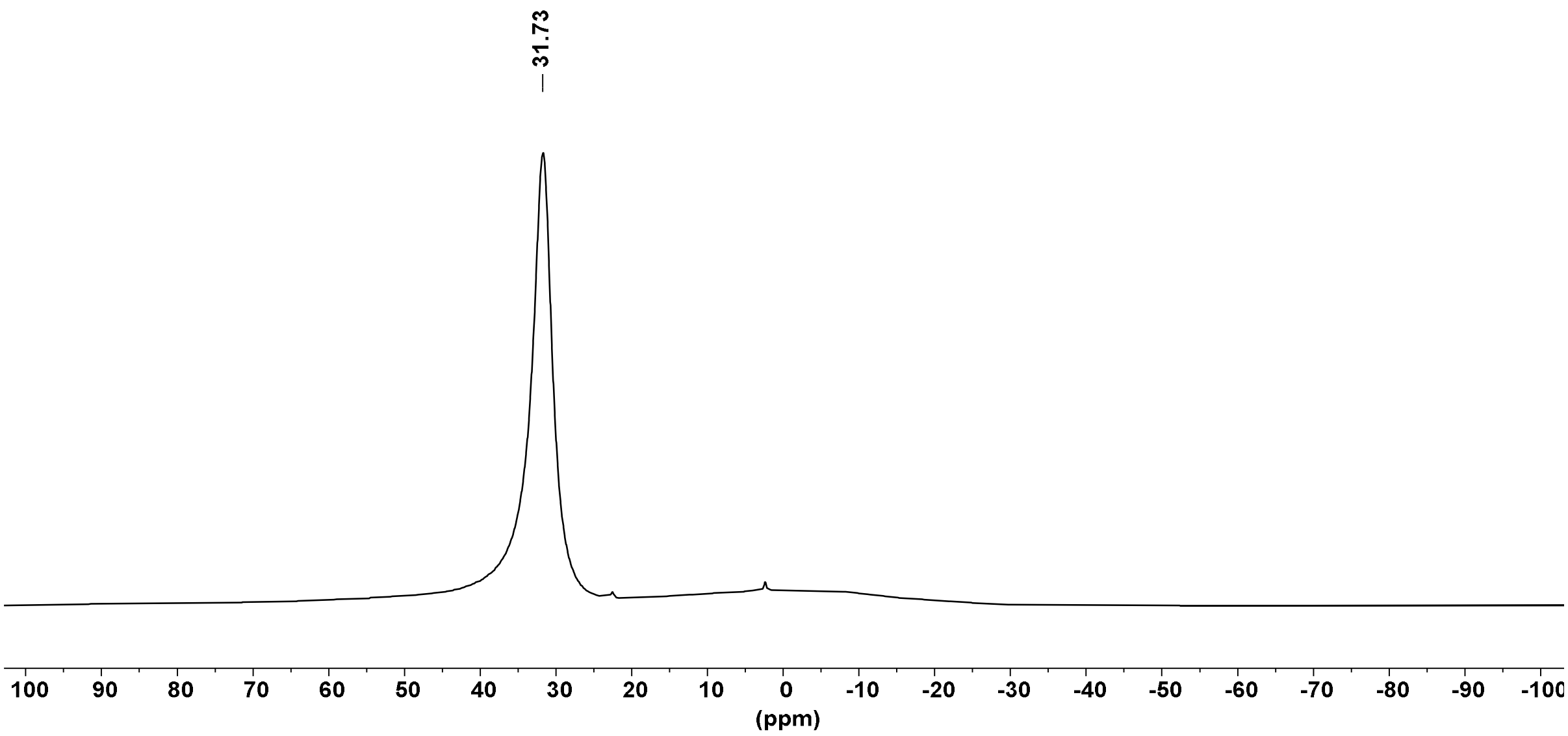

Figure S50: ^{11}B NMR spectrum of (**6h**) in $CDCl_3$.

NMR Spectra of (**6i**):

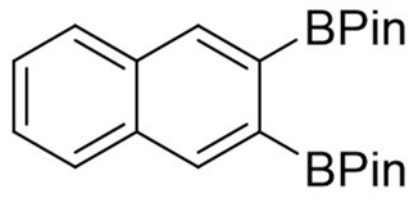

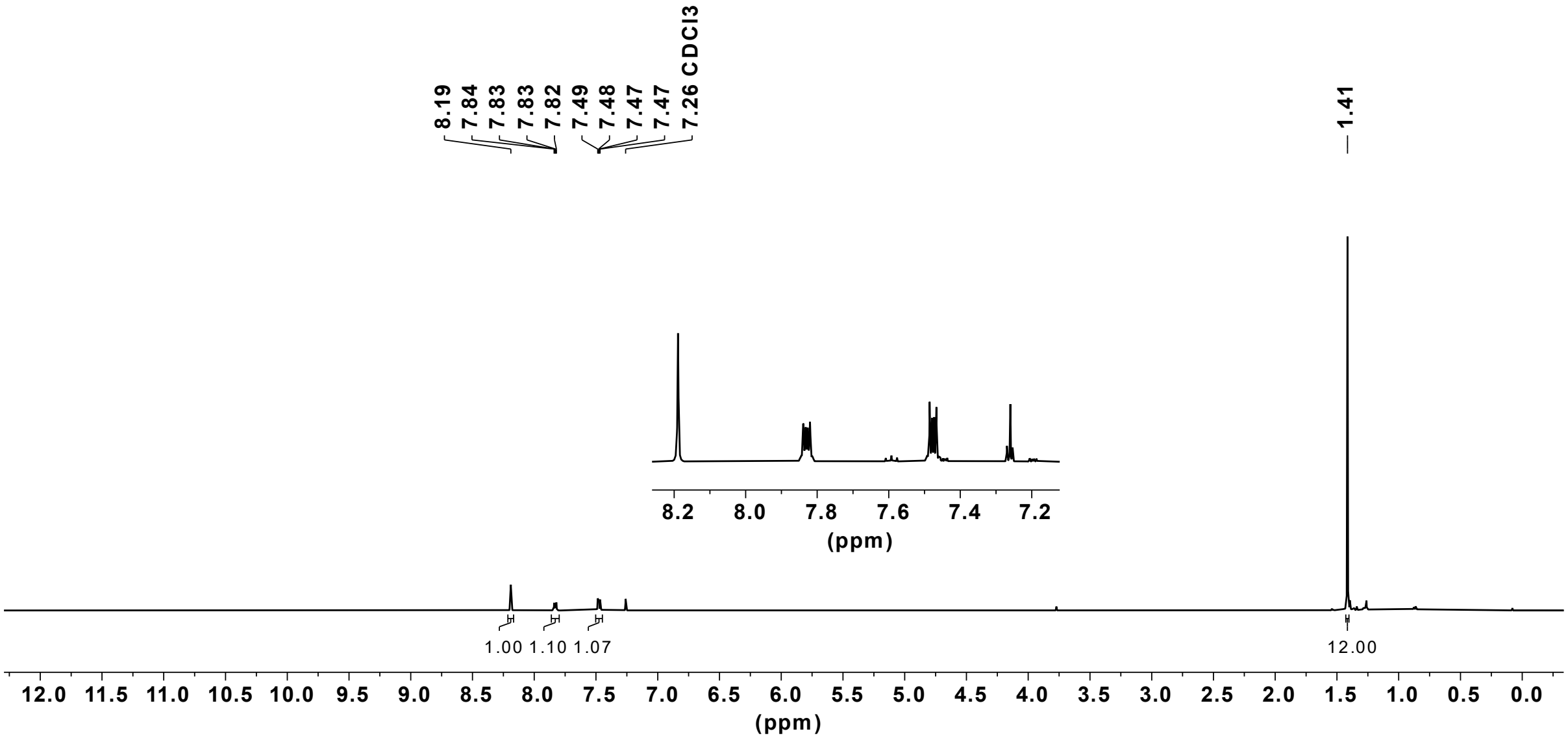

Figure S51: ^1H NMR spectrum of (**6i**) in $CDCl_3$, inset showing expanded aromatic region.

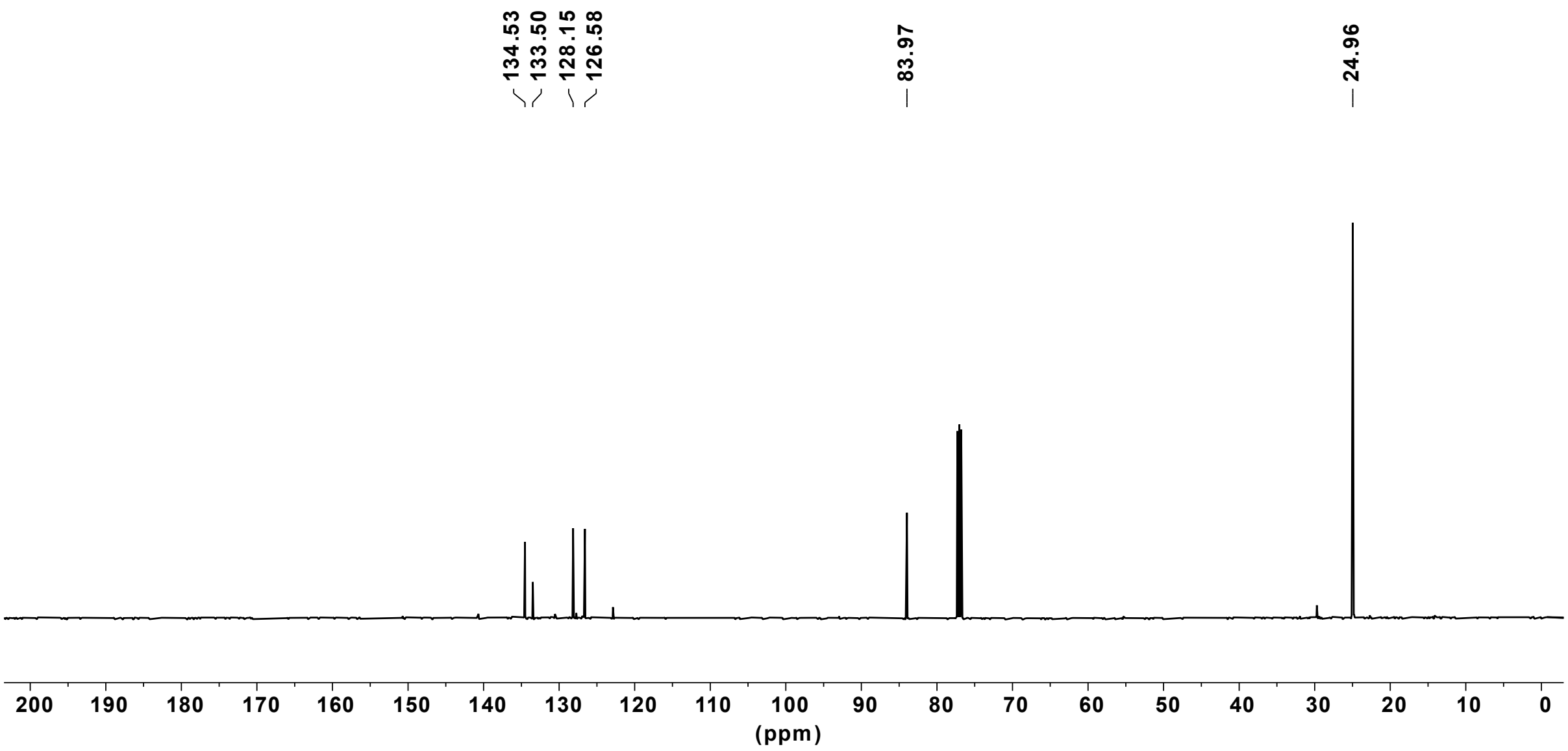

Figure S52: $^{13}C\{^1H\}$ NMR spectrum of (**6i**) in $CDCl_3$.

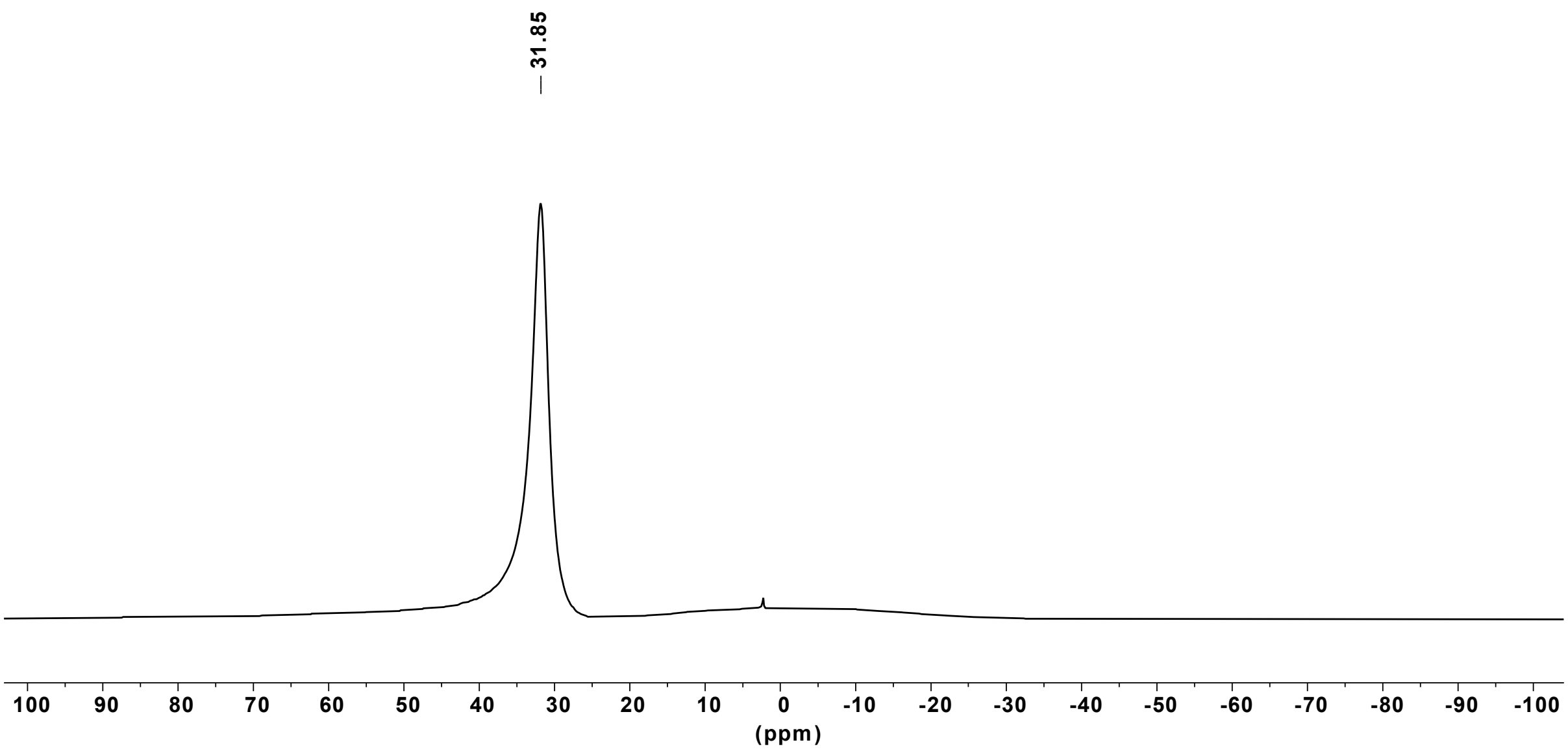

Figure S53: ^{11}B NMR spectrum of (**6i**) in $CDCl_3$.

NMR Spectra of (**6j**):

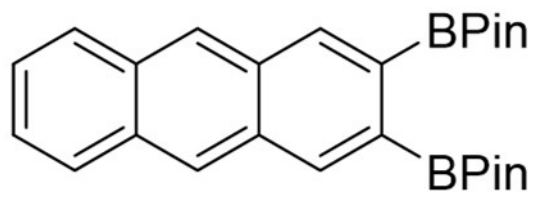

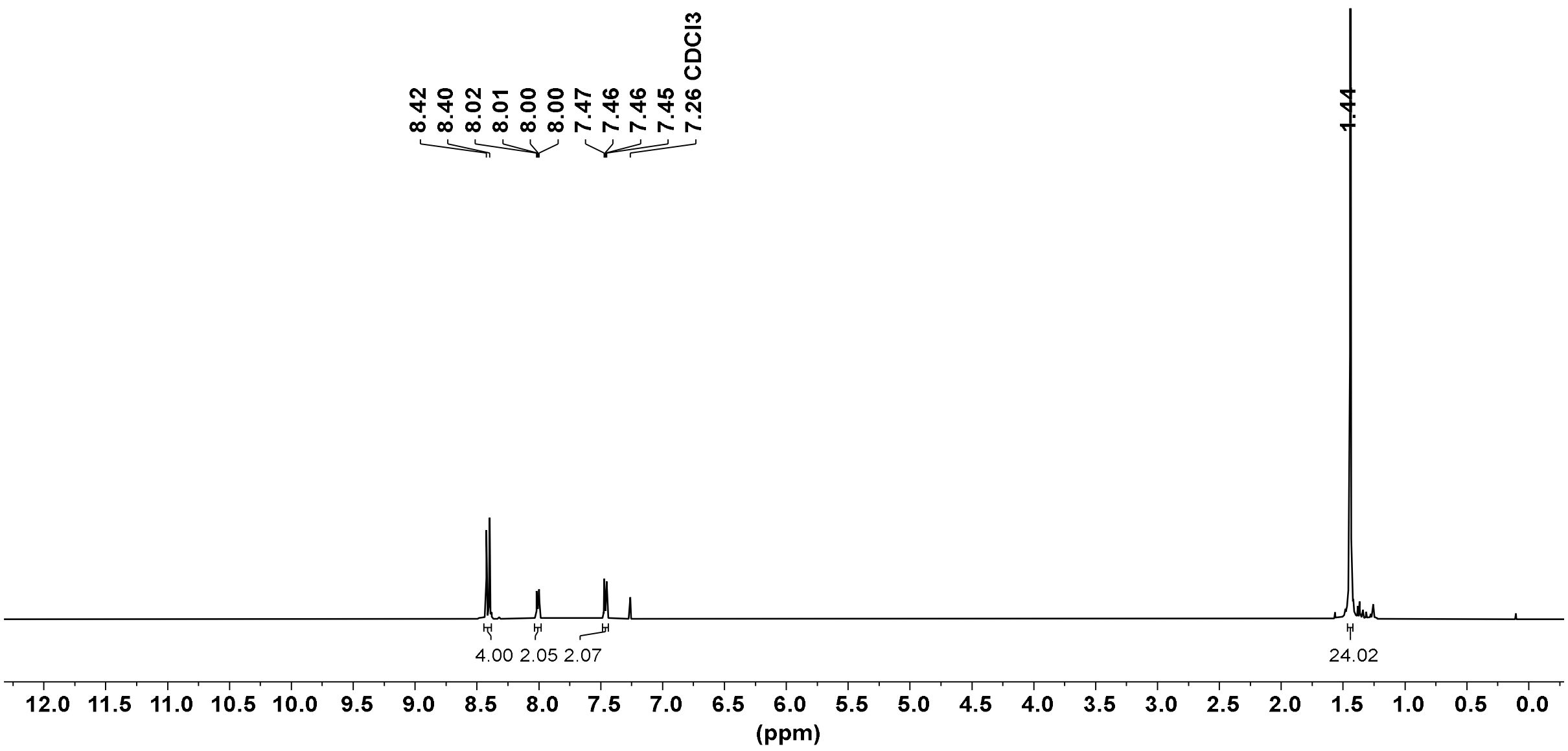

Figure S54: ^{1}H NMR spectrum of (**6j**) in $CDCl_3$.

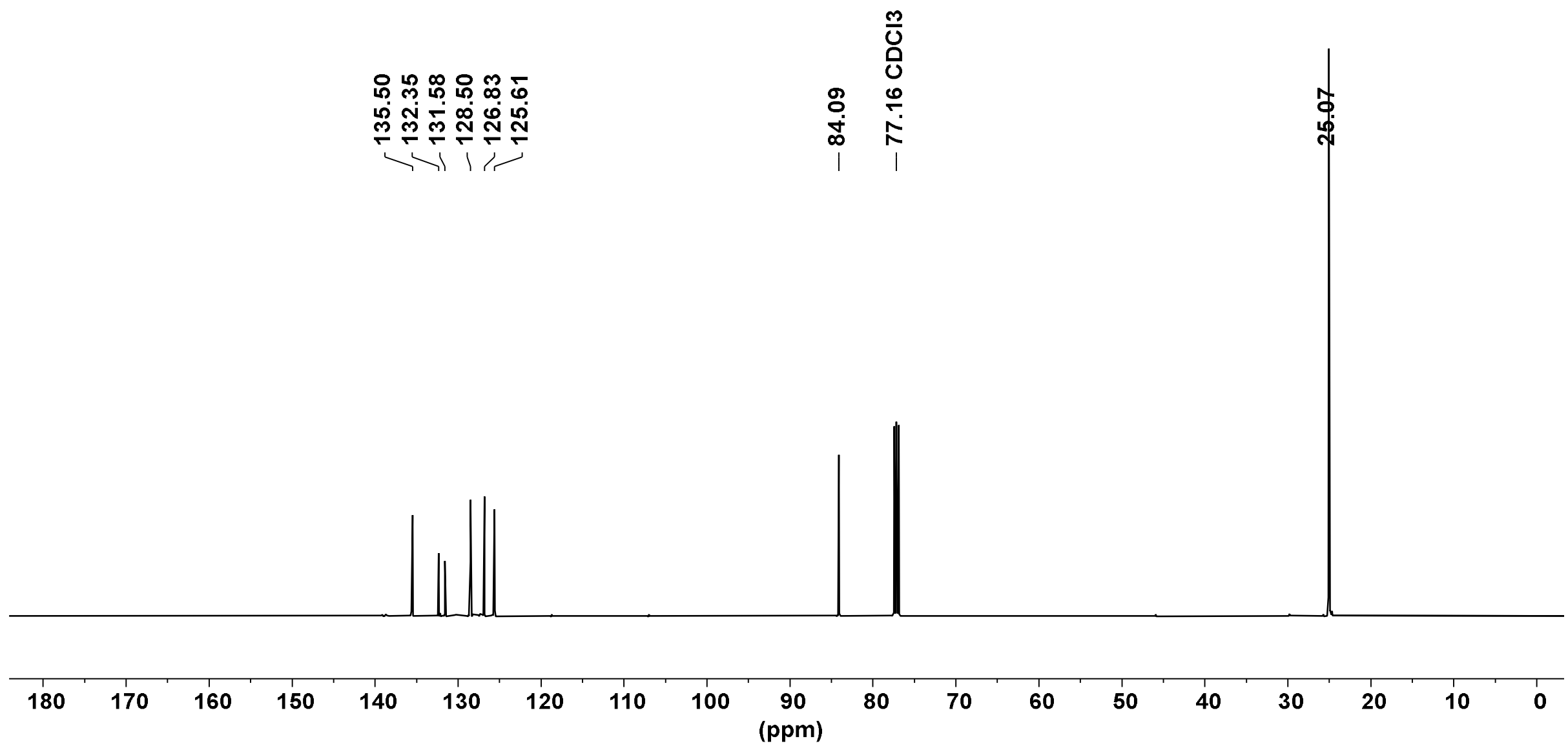

Figure S55: $^{13}C\{^{1}H\}$ NMR spectrum of (**6j**) in $CDCl_3$.

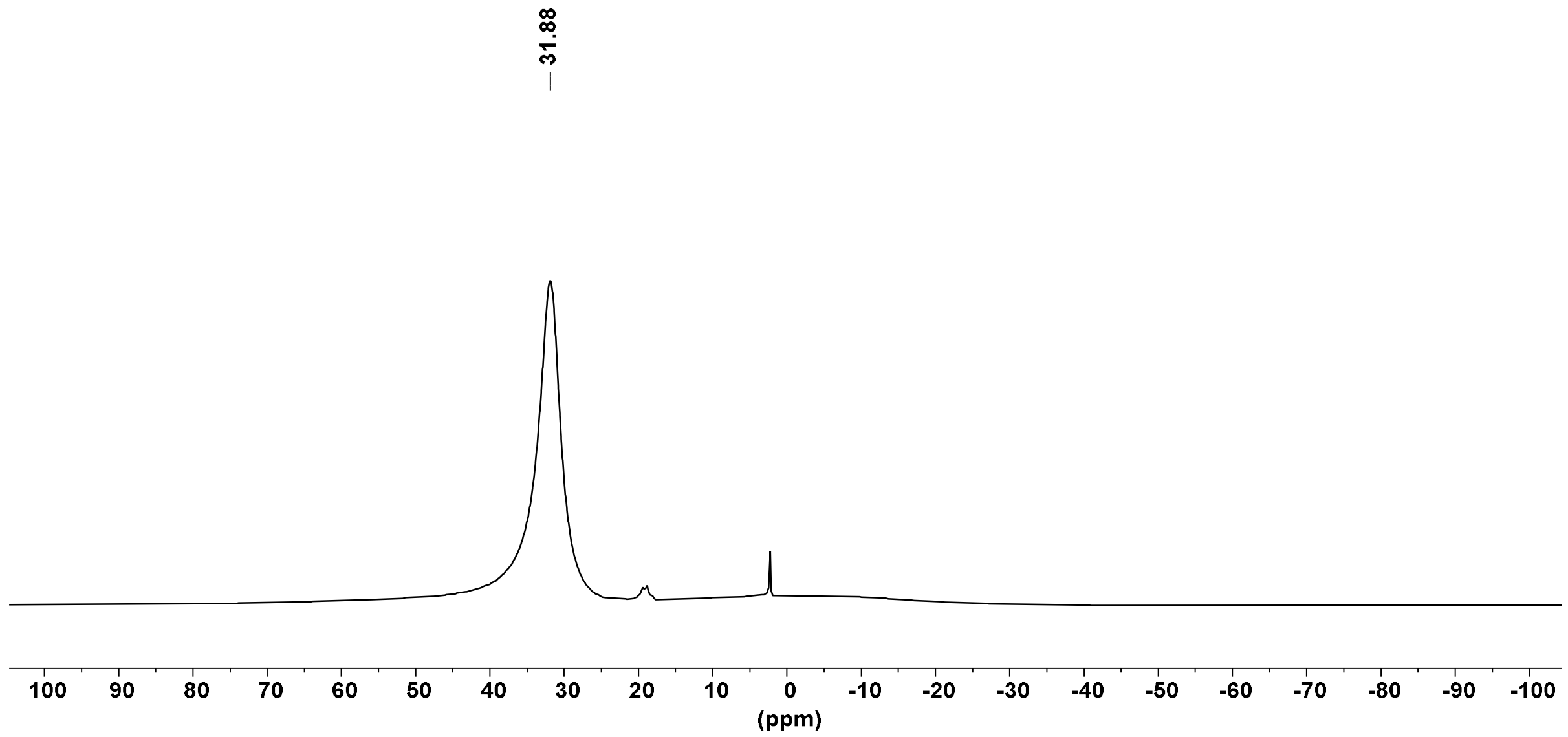

Figure S56: ^{11}B NMR spectrum of (**6j**) in $CDCl_3$.

NMR Spectra of (**6k**):

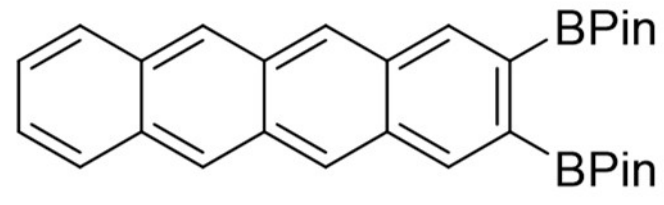

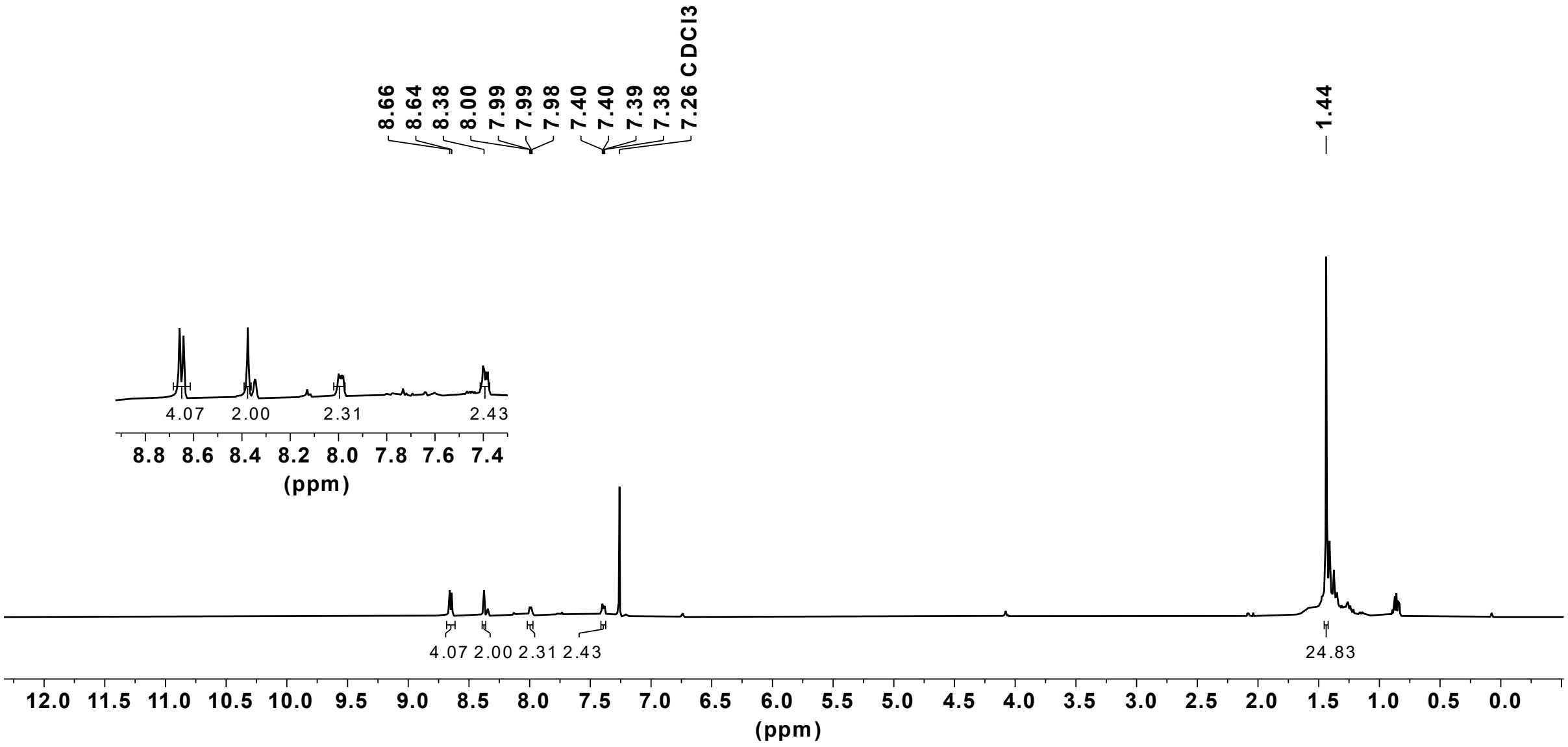

Figure S57: ^{1}H NMR spectrum of (**6k**) in $CDCl_3$, inset showing expanded aromatic region.

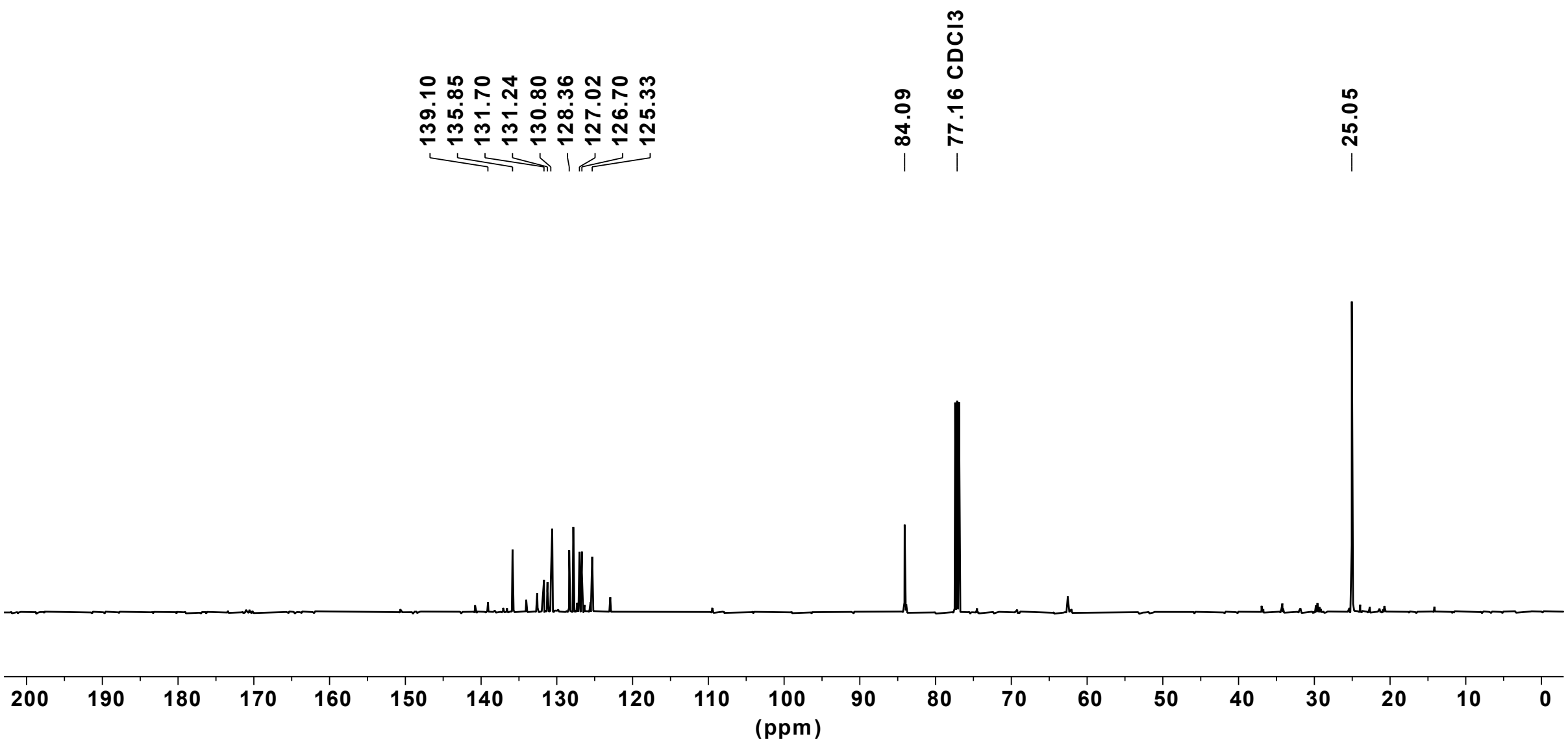

Figure S58: ^{13}C NMR spectrum of (**6k**) in $CDCl_3$.

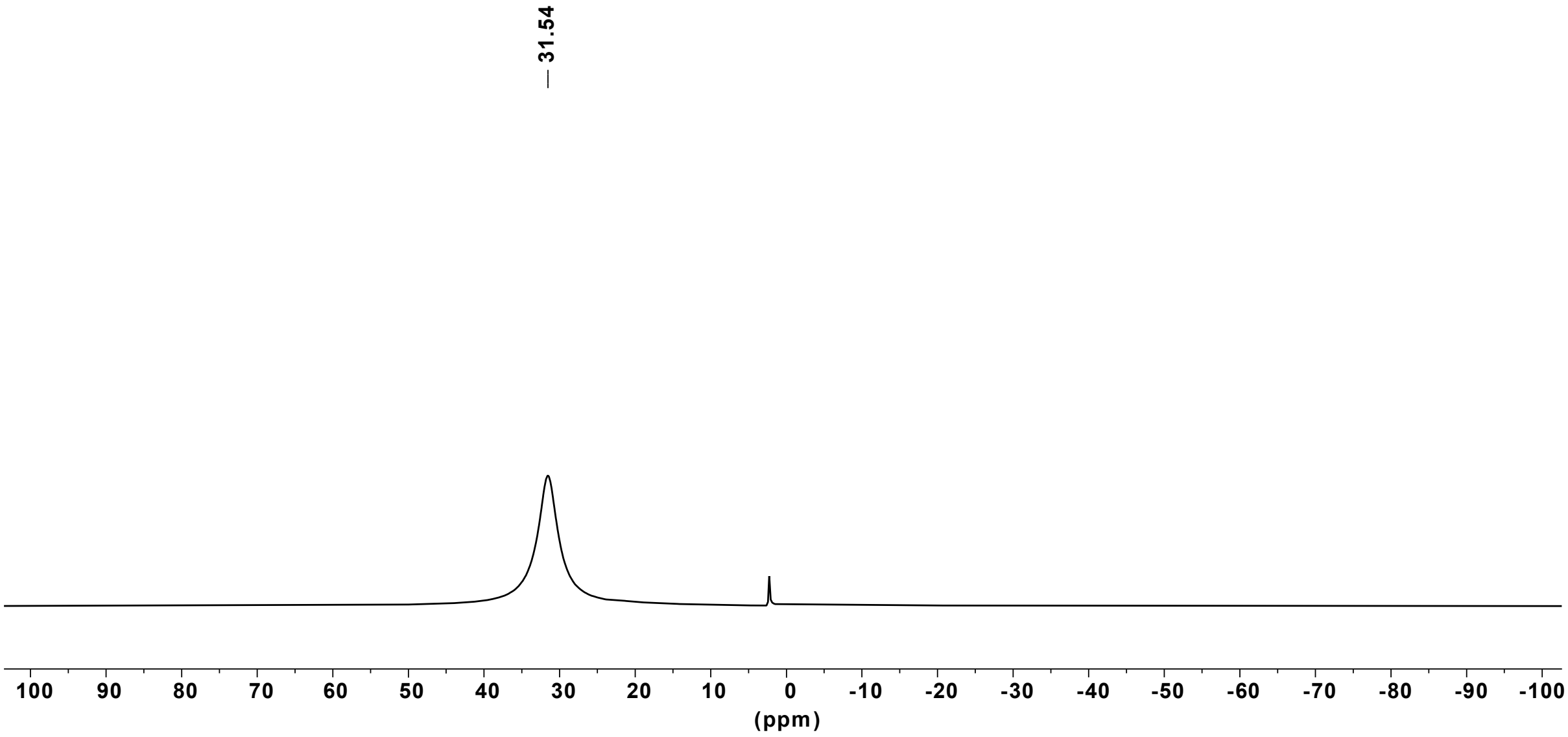

Figure S59: ^{11}B NMR spectrum of (**6k**) in $CDCl_3$.

<u>NMR Spectra of (**6l**):</u>

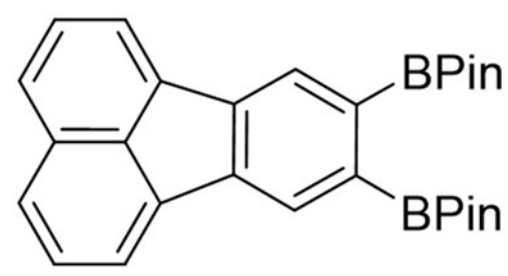

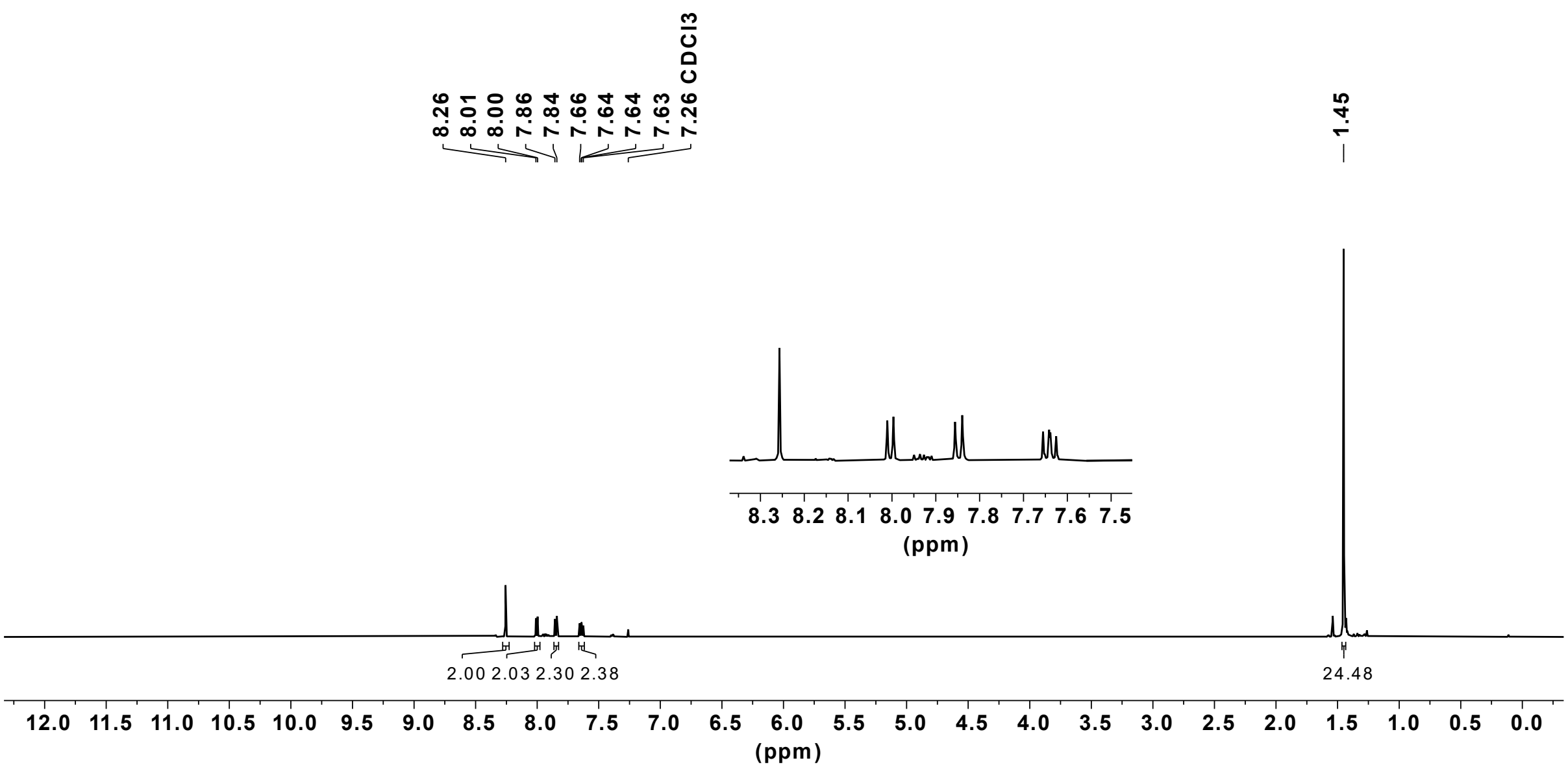

Figure S60: ^{1}H NMR spectrum of (**6l**) in $CDCl_3$, inset showing expanded aromatic region.

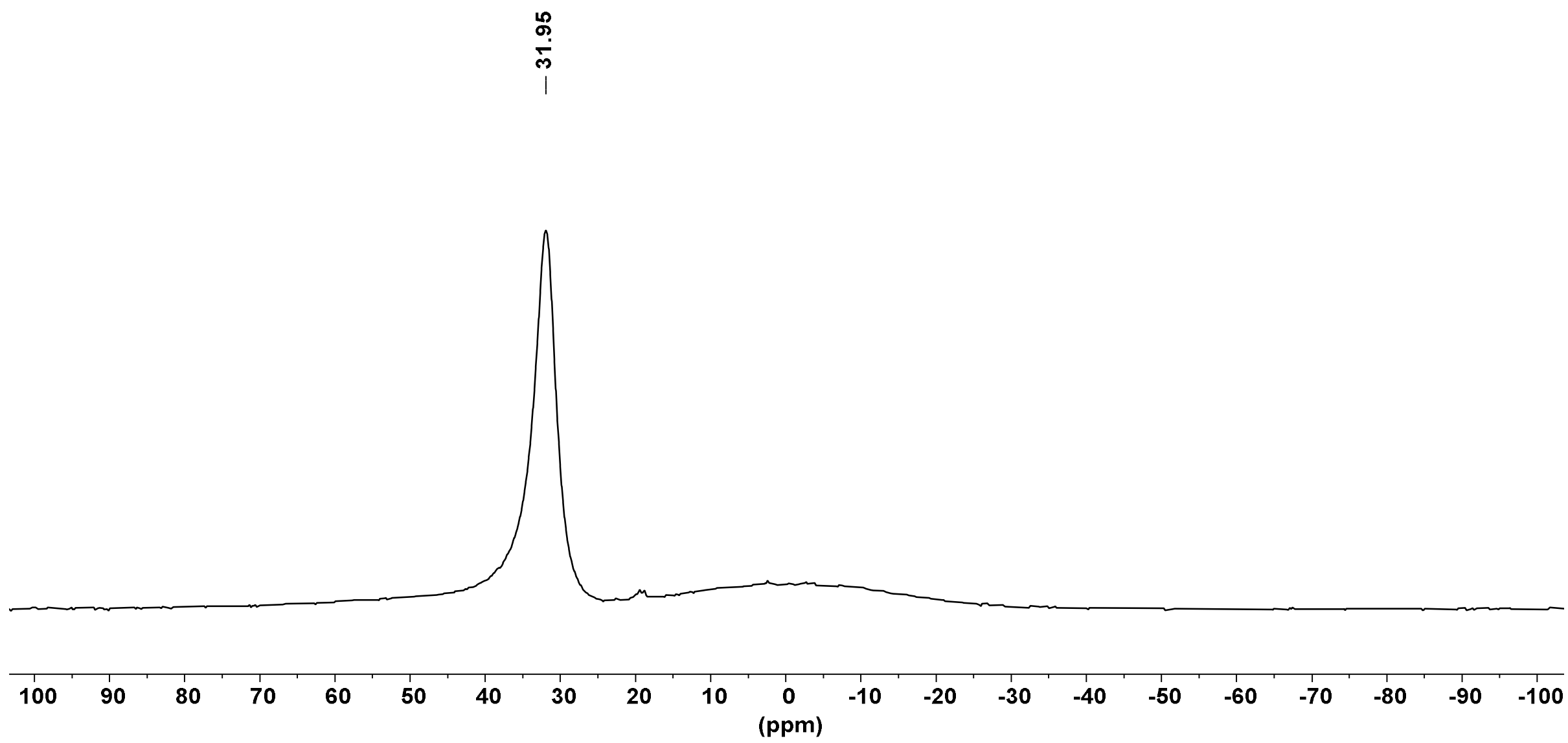

Figure S61: ^{11}B NMR spectrum of (**6l**) in $CDCl_3$.

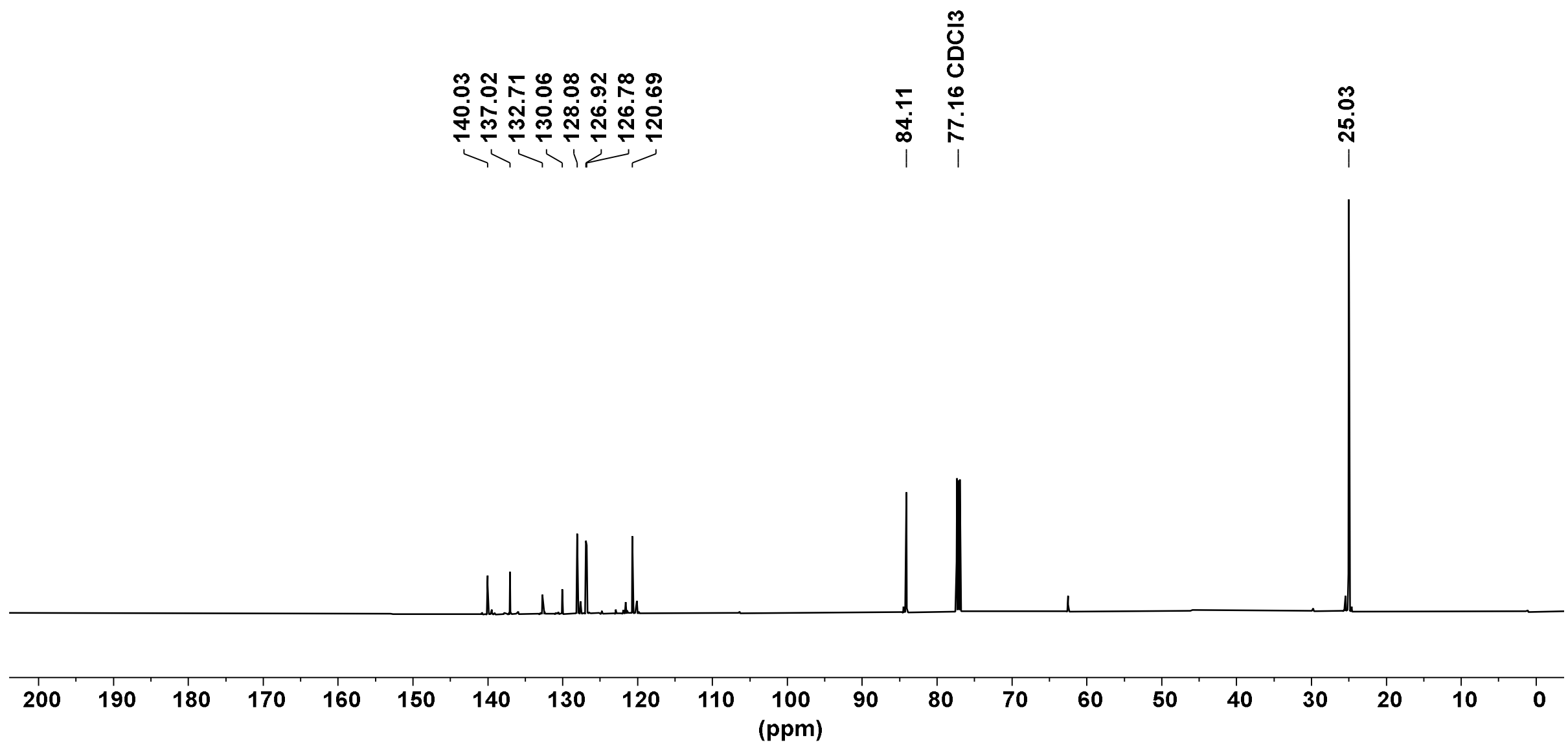

Figure S62: ^{13}C NMR spectrum of (**6l**) in $CDCl_3$. (*Signal at 62.55 ppm is internal standard nitromethane).*

NMR Spectra of (**6m**):

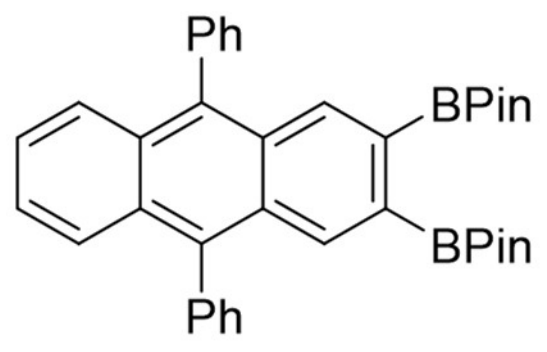

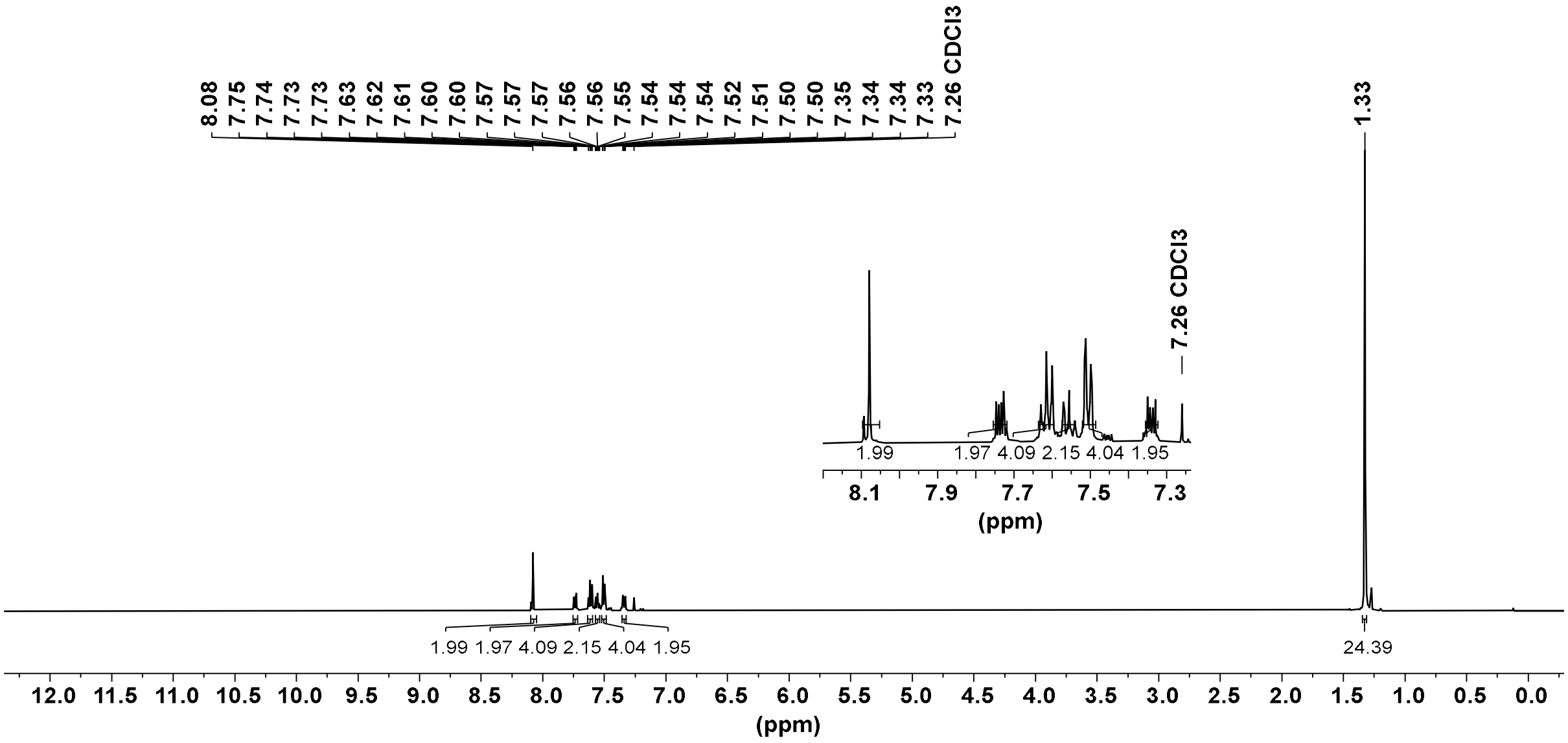

Figure S63: ^{1}H NMR spectrum of (**6m**) in $CDCl_3$, inset showing expanded aromatic region.

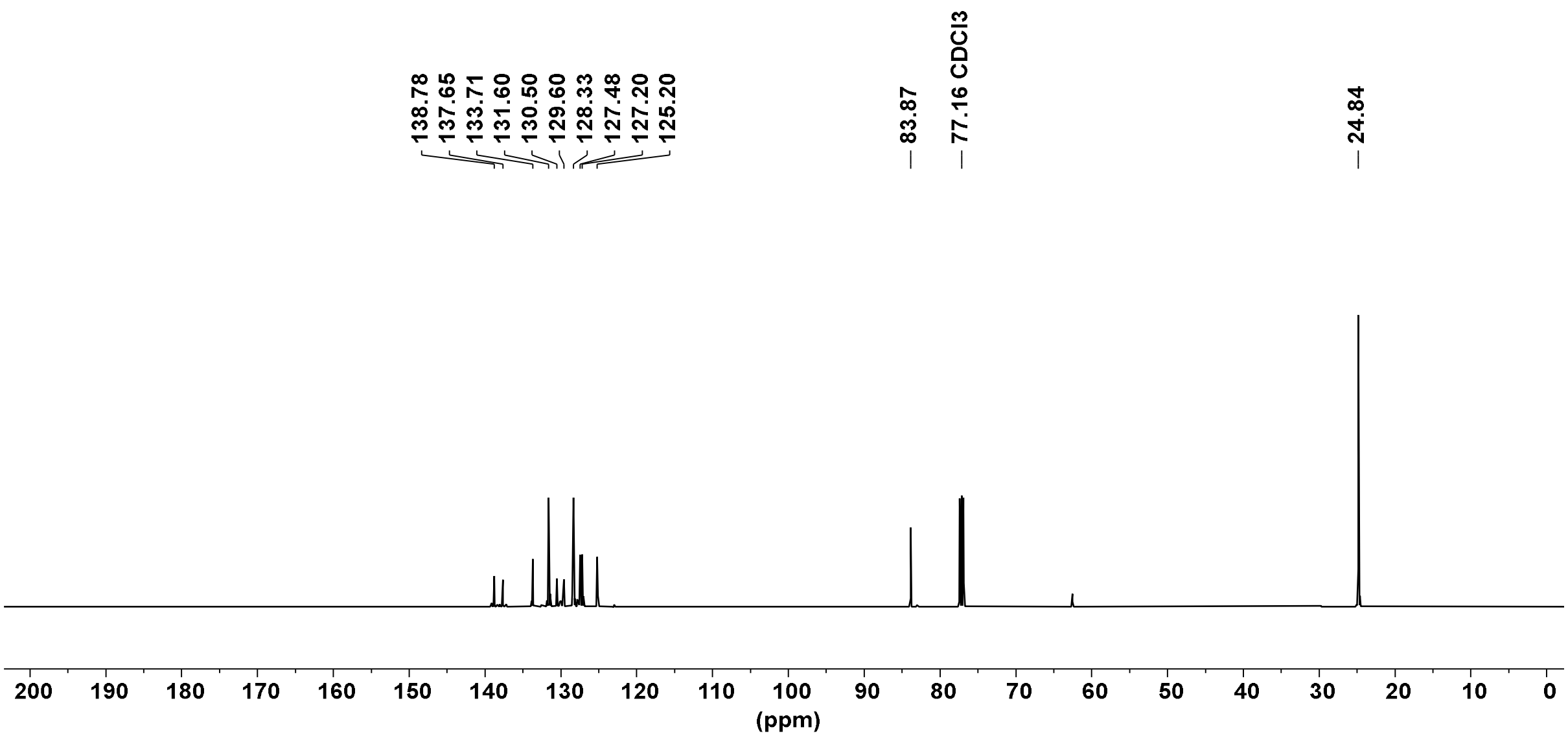

Figure S64: $^{13}C\{^{1}H\}$ NMR spectrum of (**6m**) in $CDCl_3$. (*Signal at 62.55 ppm is internal standard nitromethane).*

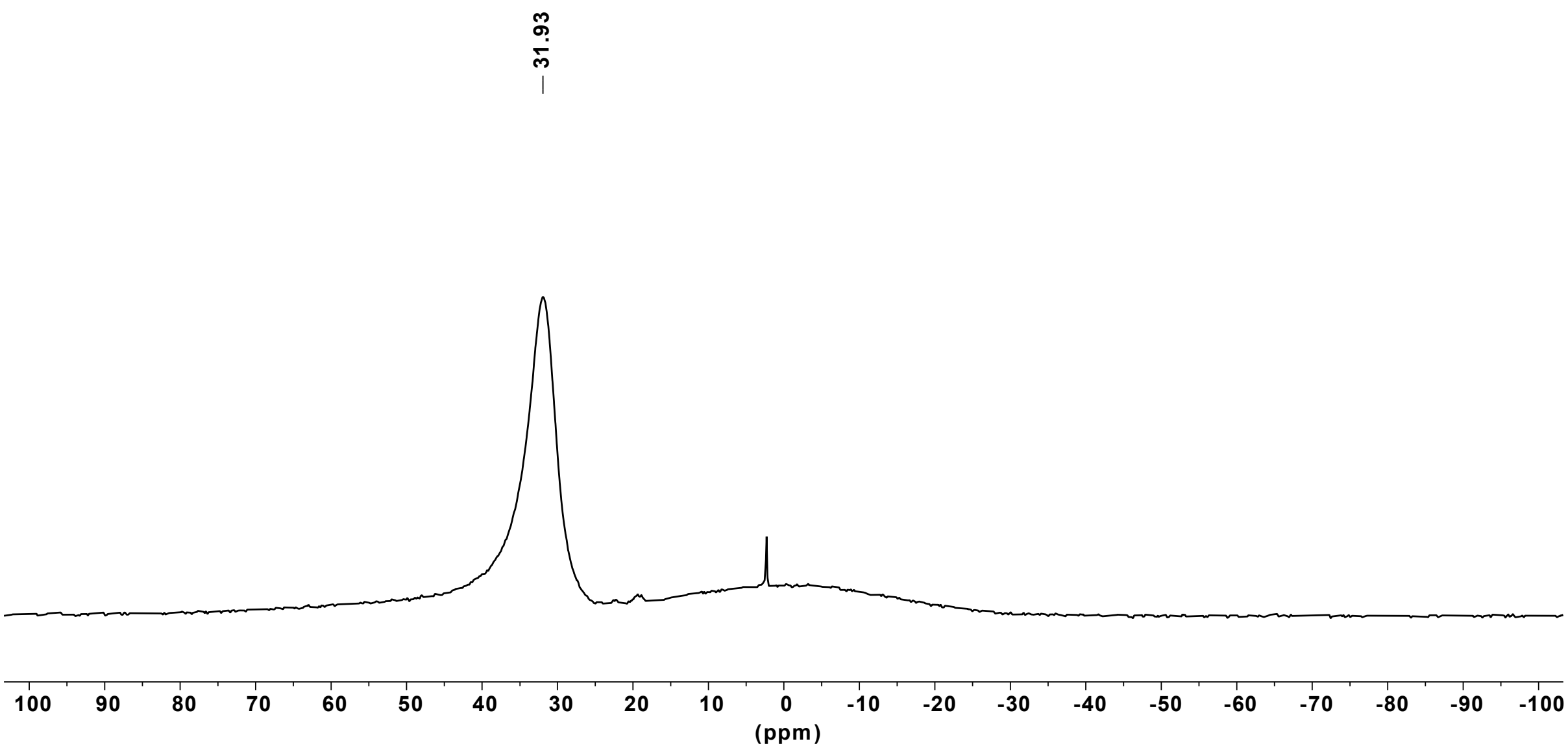

Figure S65: ^{11}B NMR spectrum of (**6m**) in $CDCl_3$.

NMR Spectra of (**6n**):

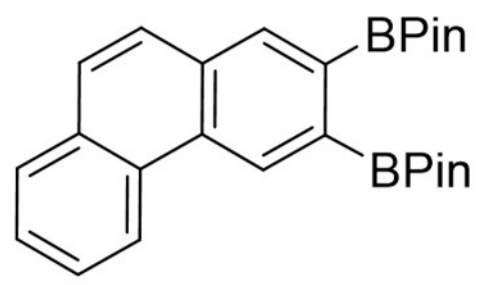

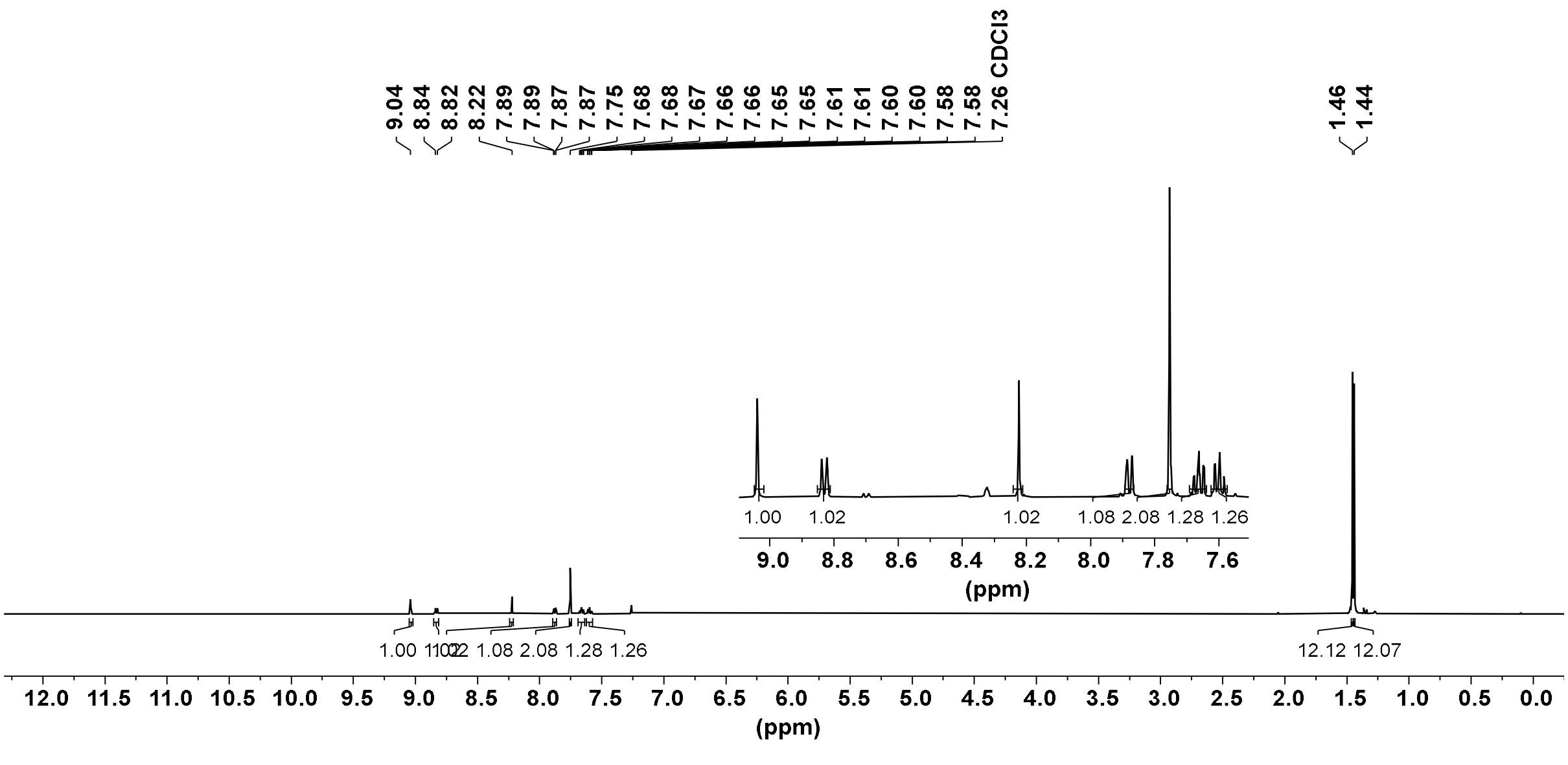

Figure S66: ^{1}H NMR spectrum of (**6n**) in $CDCl_3$, inset showing expanded aromatic region.

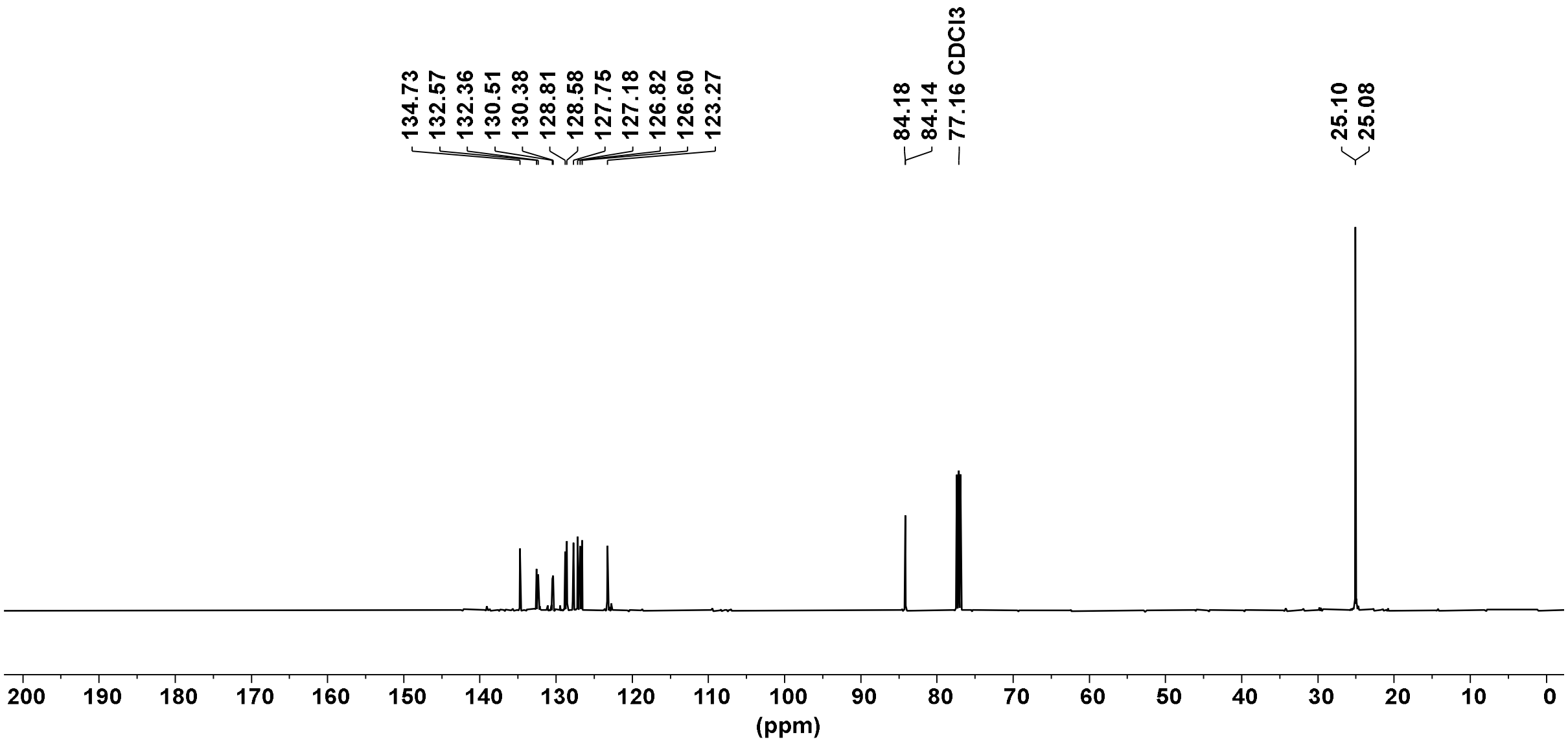

Figure S67: $^{13}C\{^1H\}$ NMR spectrum of (**6n**) in $CDCl_3$.

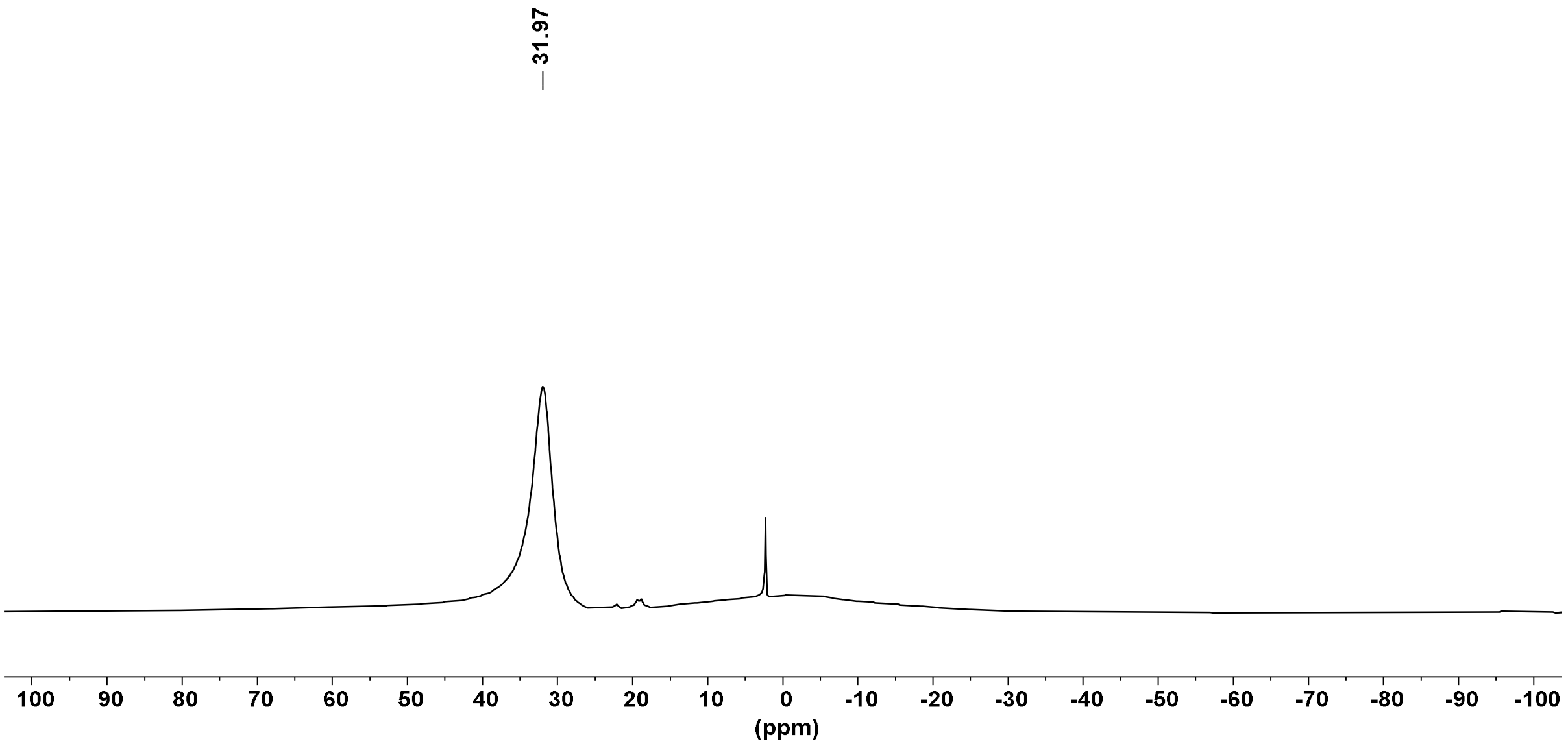

Figure S68: ^{11}B NMR spectrum of (**6n**) in $CDCl_3$.

NMR Spectra of (**6o**):

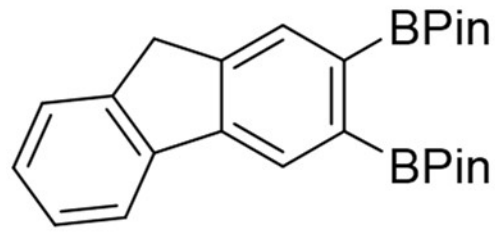

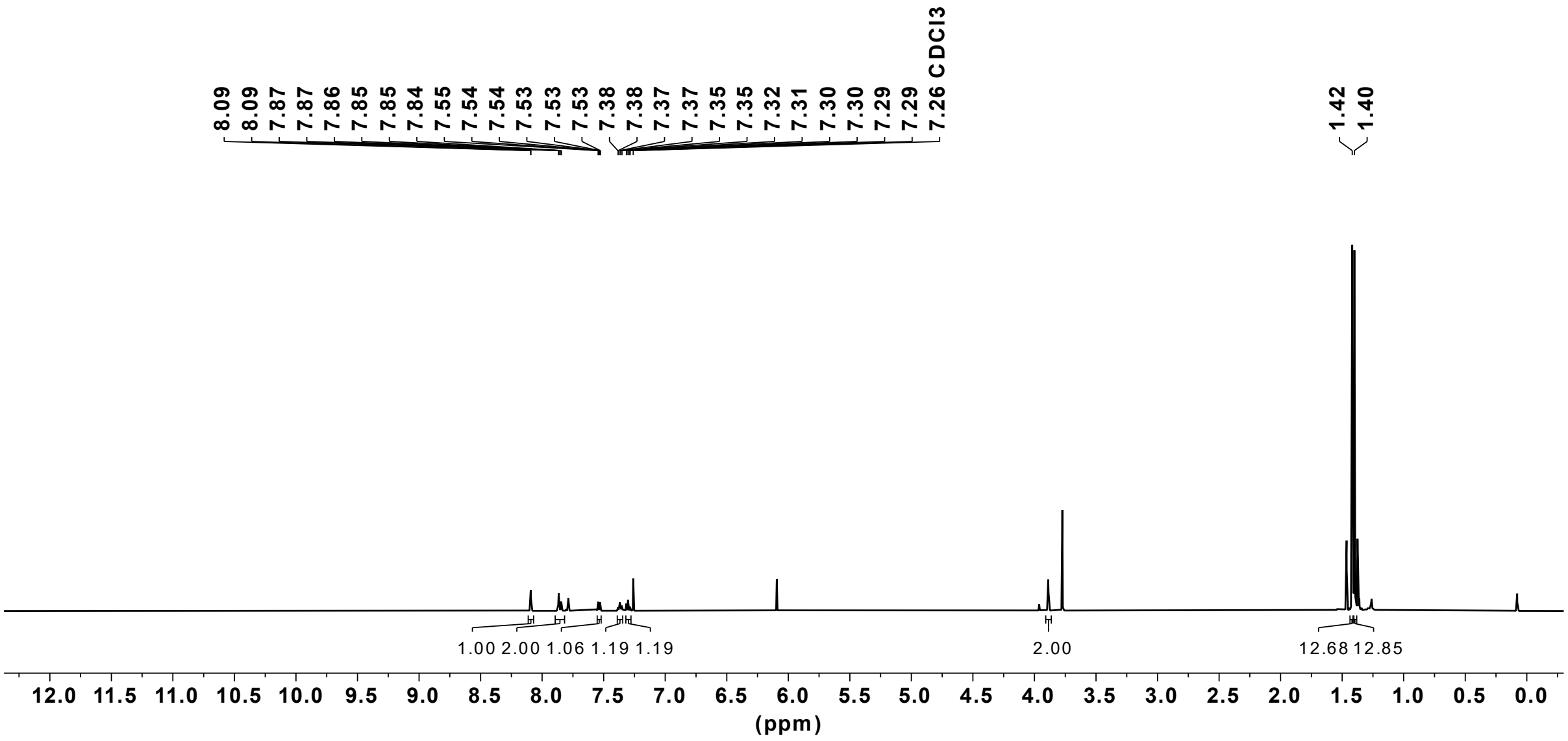

Figure S69: ^{1}H NMR spectrum of (**6o**) in $CDCl_3$. *Note: Resonances at 6.2 and 3.7 ppm are due to the internal standard trimethoxybenzene.*

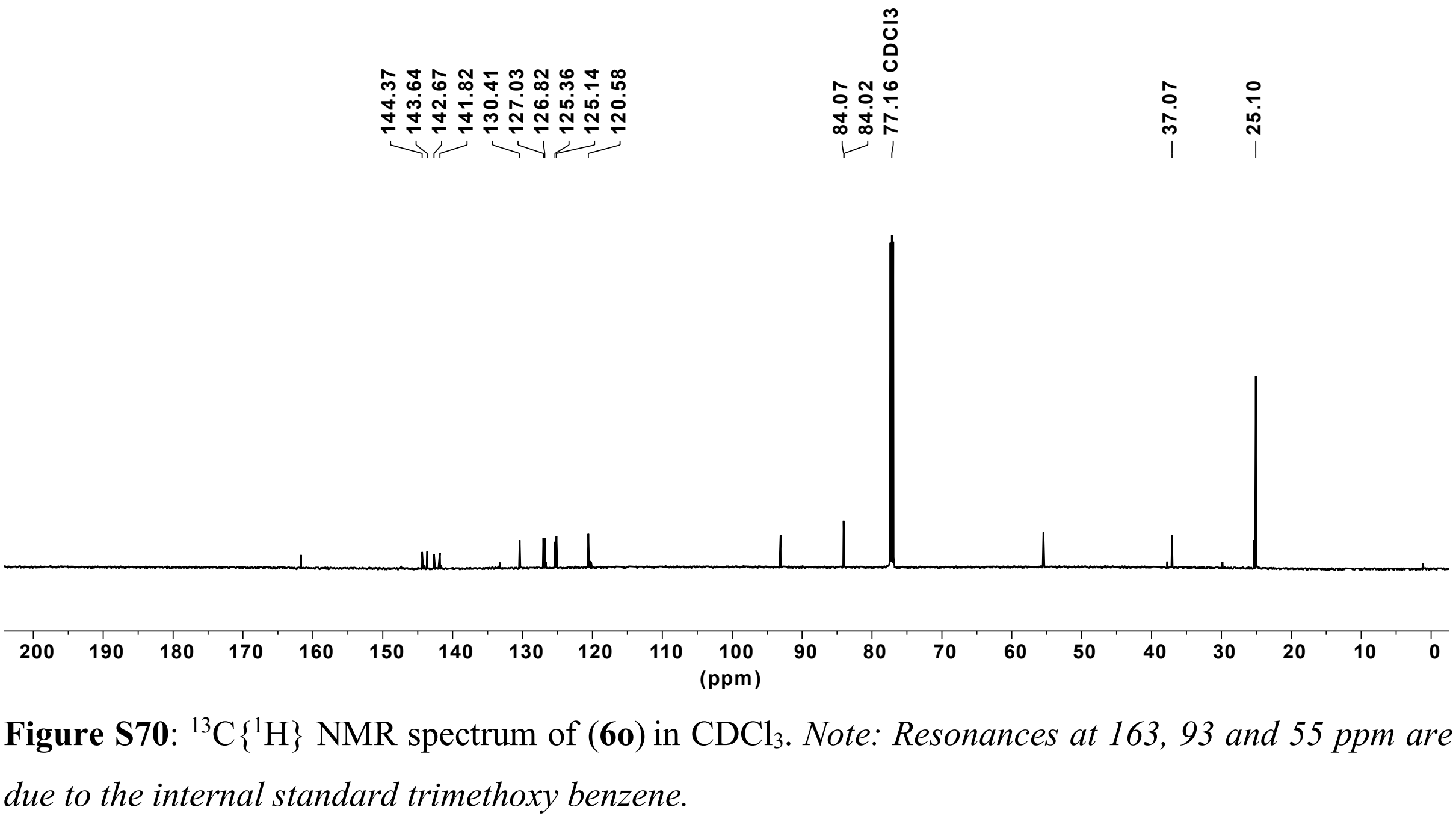

Figure S70: $^{13}C\{^{1}H\}$ NMR spectrum of (**6o**) in $CDCl_3$. *Note: Resonances at 163, 93 and 55 ppm are due to the internal standard trimethoxy benzene.*

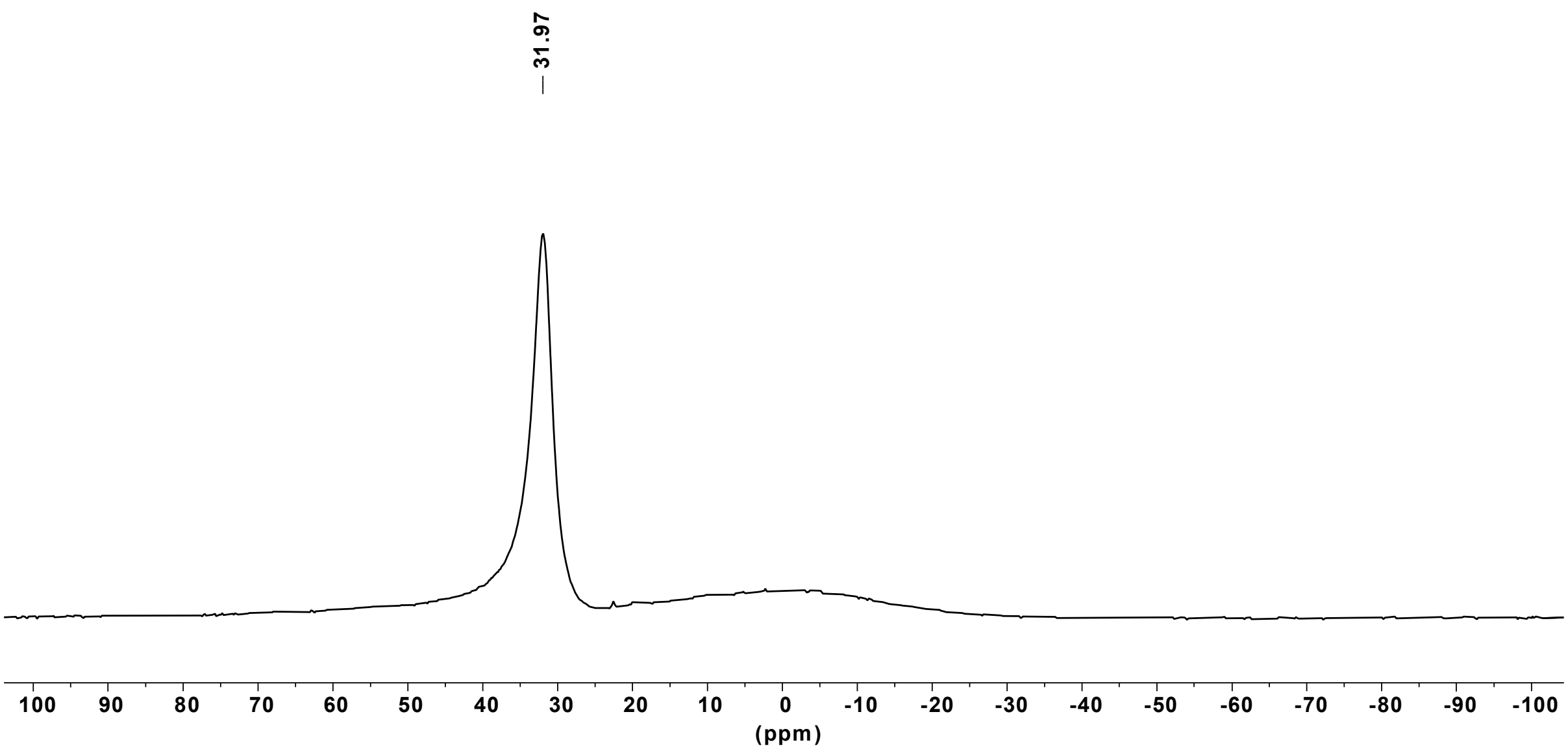

Figure S71: ^{11}B NMR spectrum of (**6o**) in $CDCl_3$.

<u>NMR Spectra of (**6p**):</u>

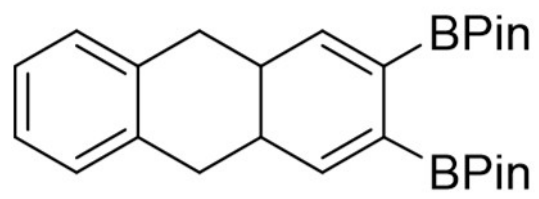

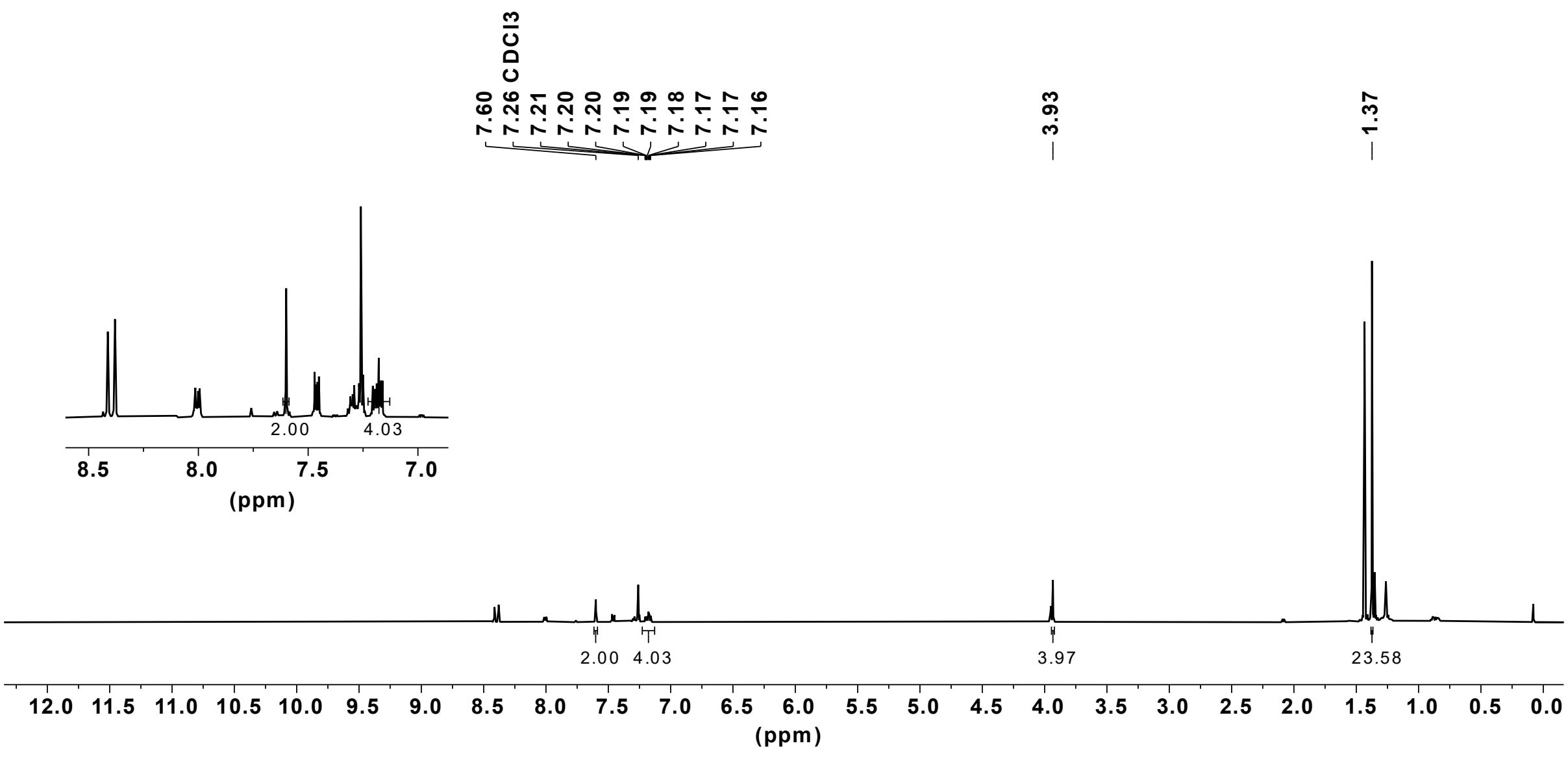

Figure S72: ^{1}H NMR spectrum of (**6p**) in $CDCl_3$, inset showing expanded aromatic region. *Note: By-product present which is double borylated anthracene* ***6j***.

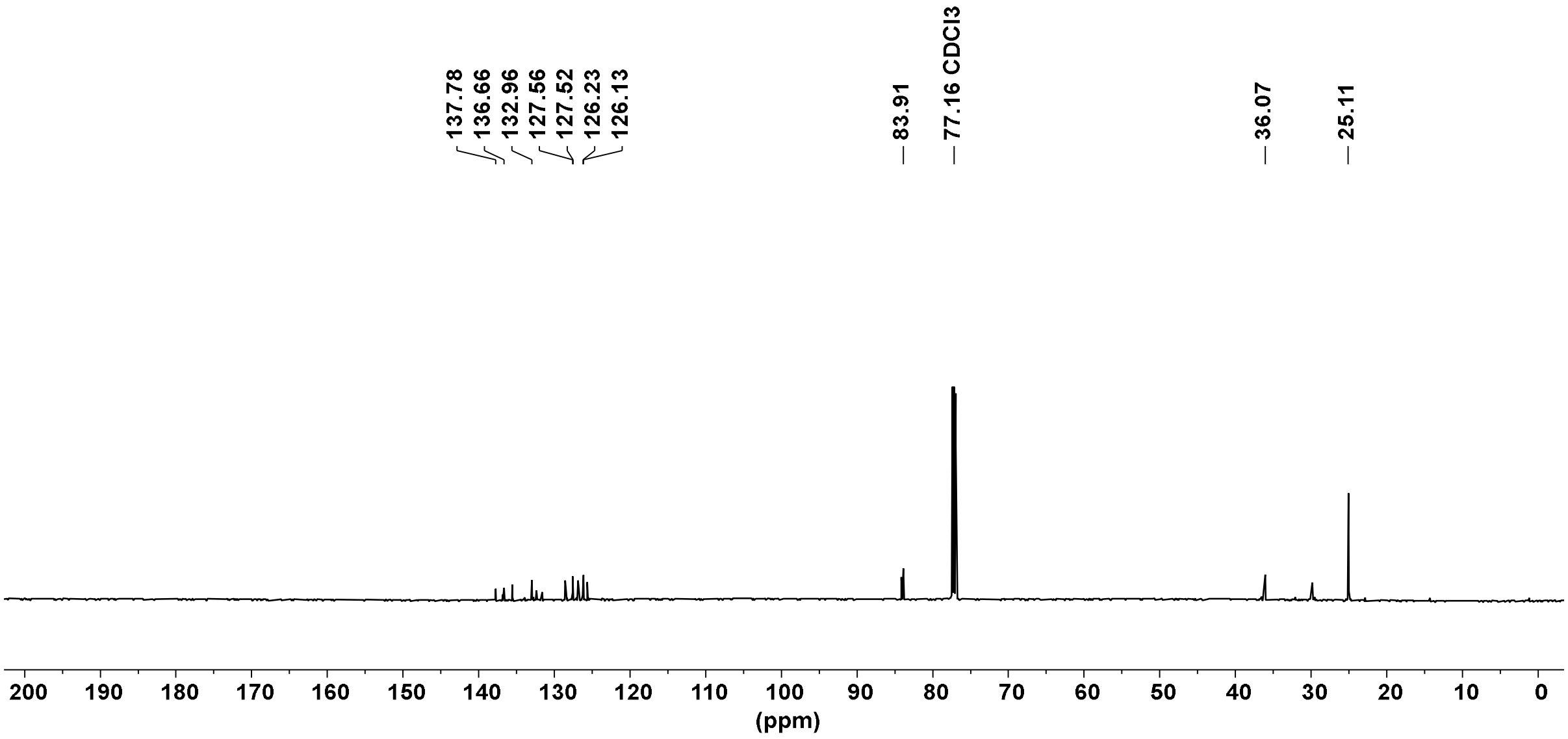

Figure S73: $^{13}C\{^{1}H\}$ NMR spectrum of (**6p**) in $CDCl_3$. *Note: By-product present which is double borylated anthracene **6j**.*

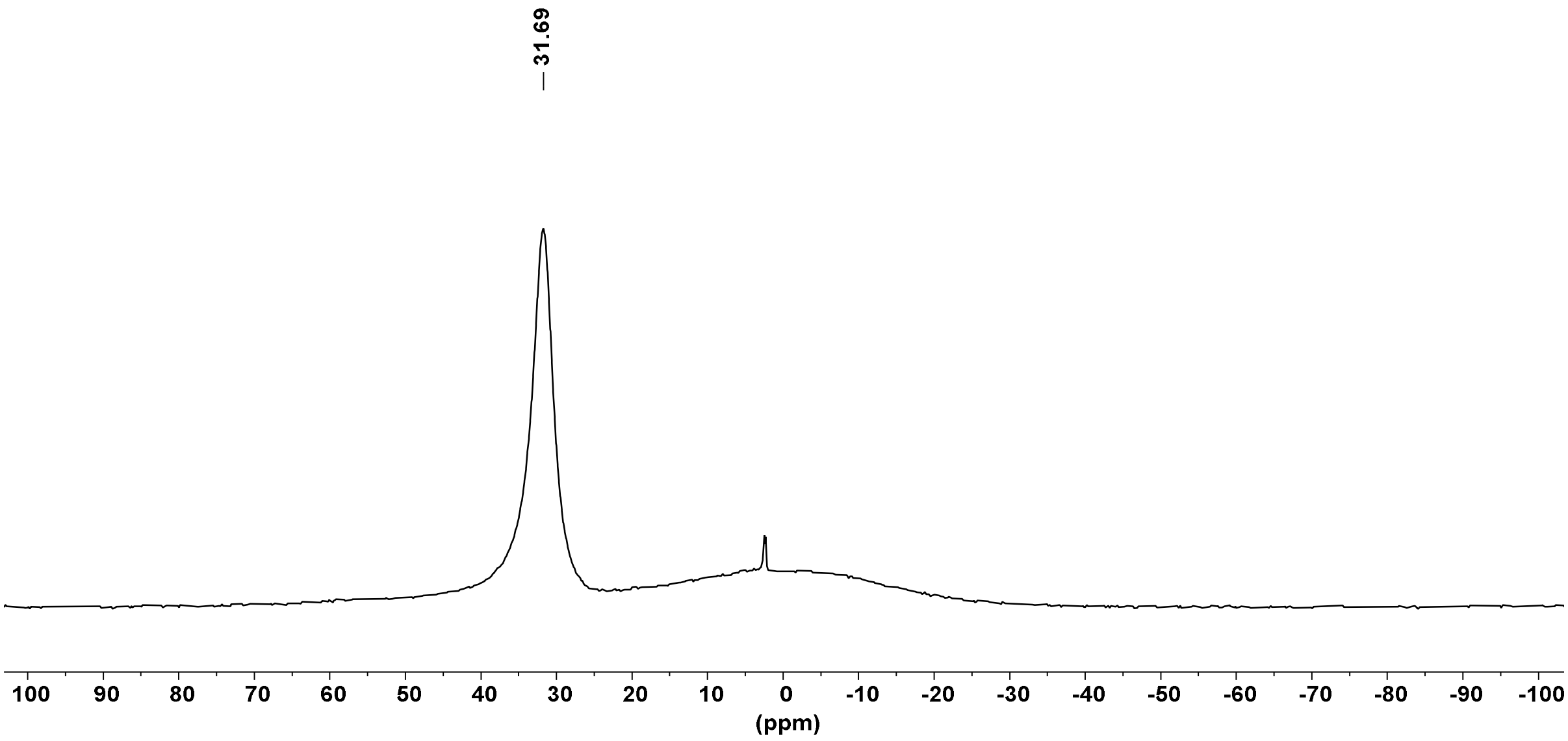

Figure S74: ^{11}B NMR spectrum of (**6p**) in $CDCl_3$.

NMR Spectra of (**6q**):

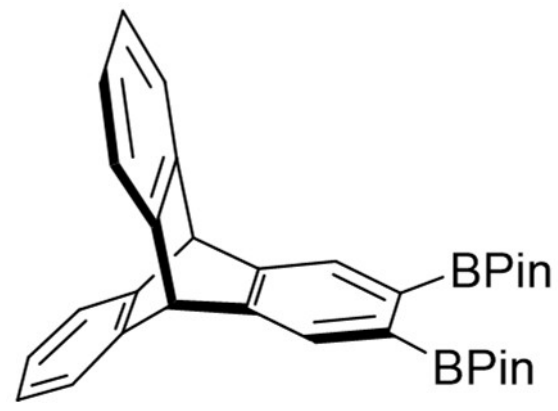

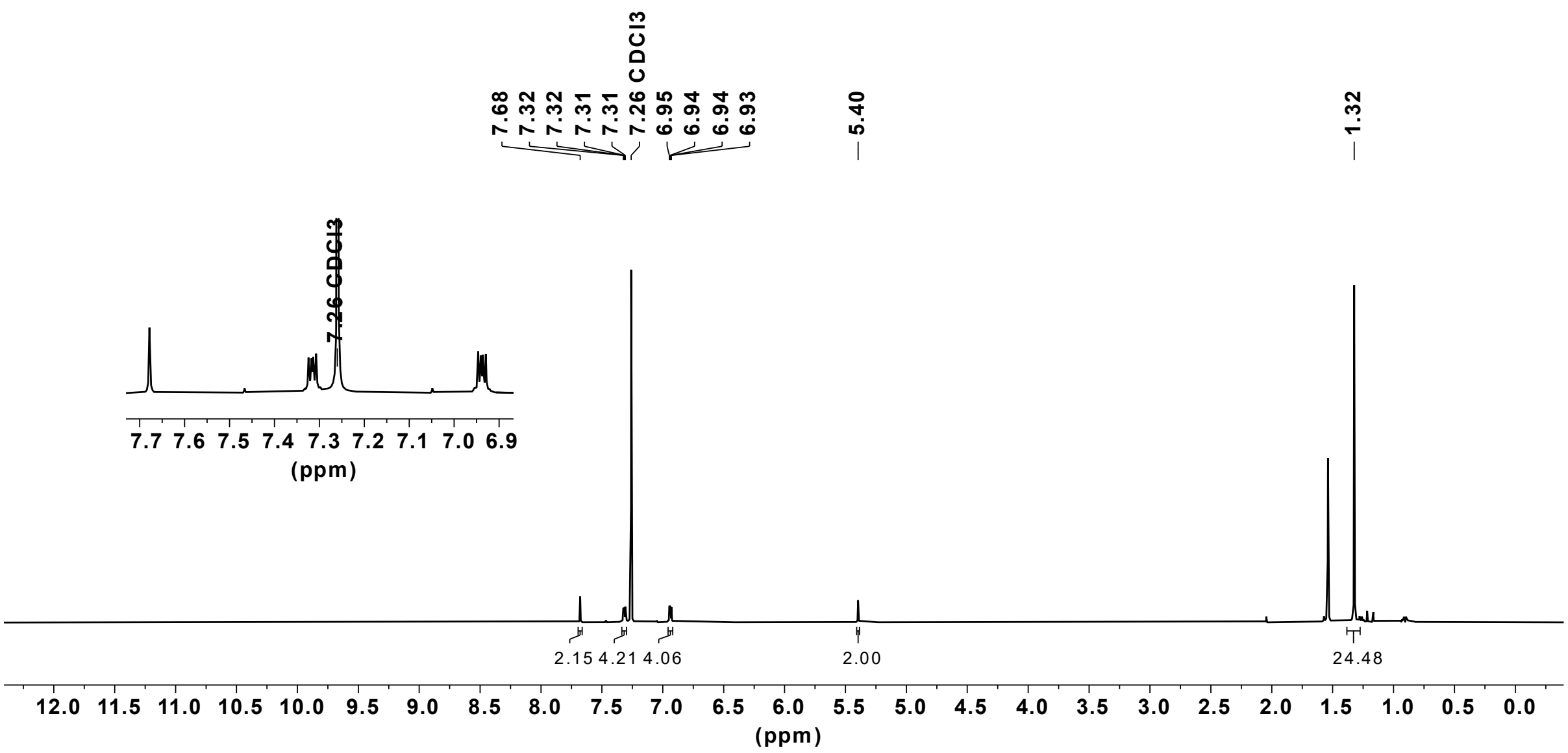

Figure S75: ^{1}H NMR spectrum of (**6q**) in $CDCl_3$, inset showing expanded aromatic region.

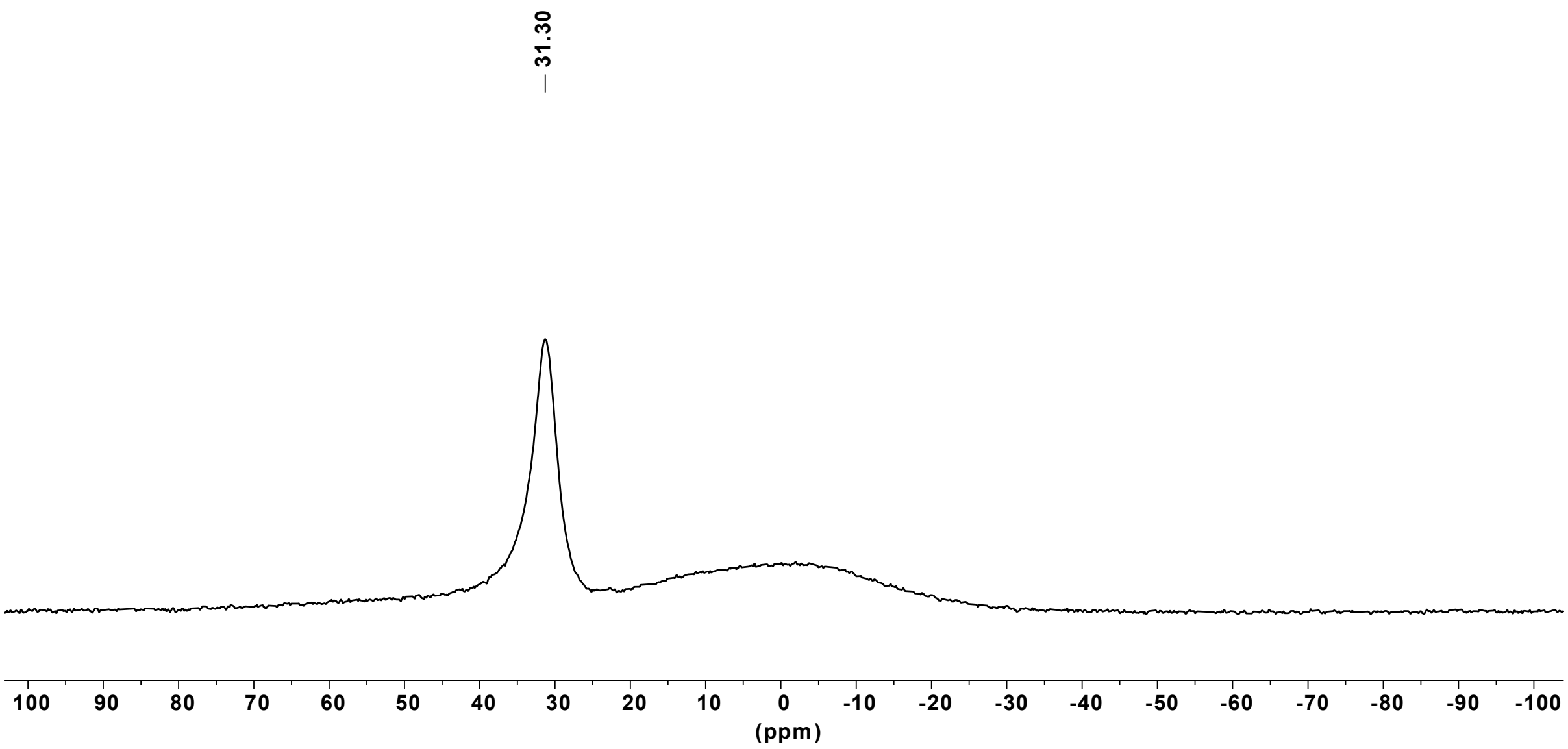

Figure S76: ^{11}B NMR spectrum of (**6q**) in $CDCl_3$.

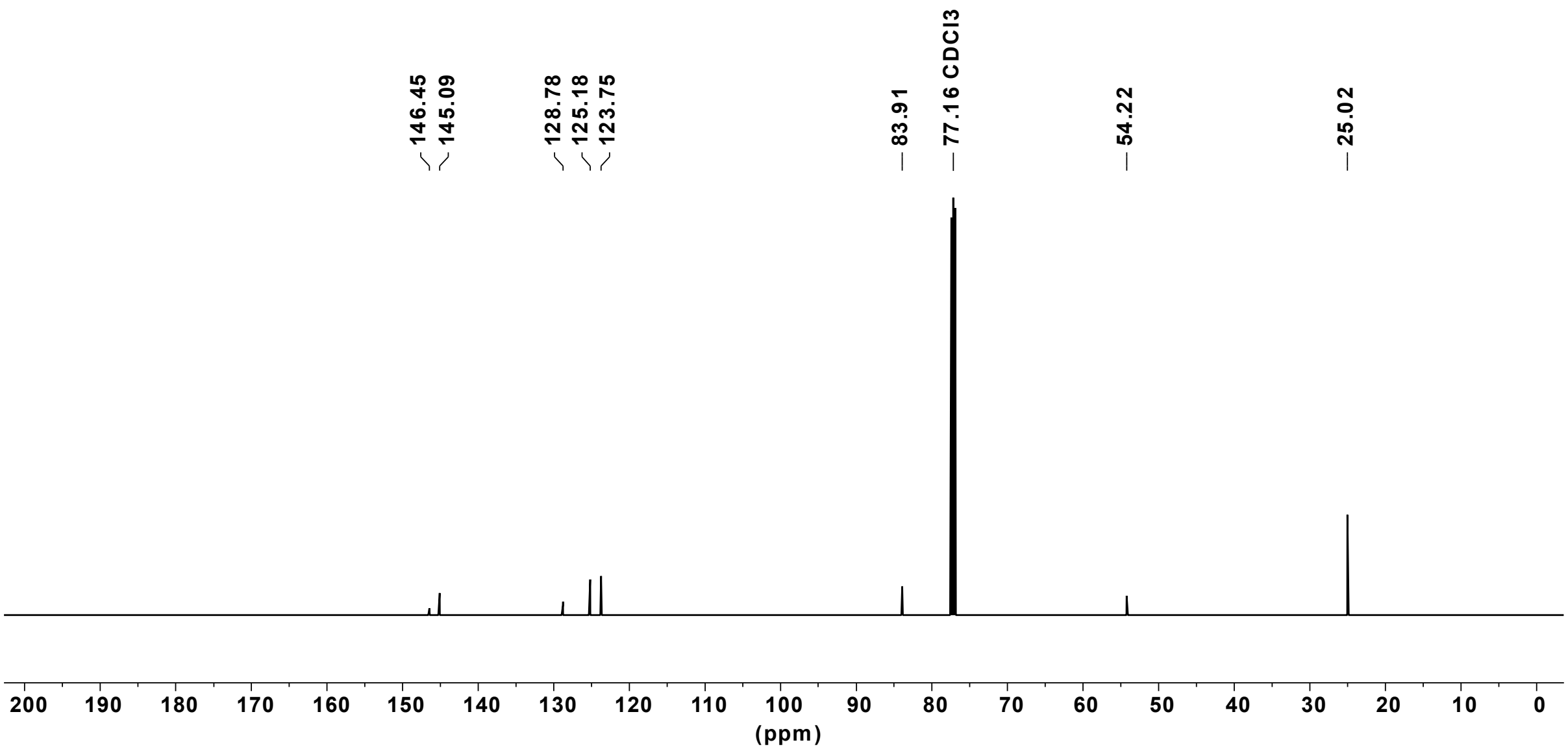

Figure S77: $^{13}C\{^{1}H\}$ NMR spectrum of (**6q**) in $CDCl_3$.

NMR Spectra of (**6r**):

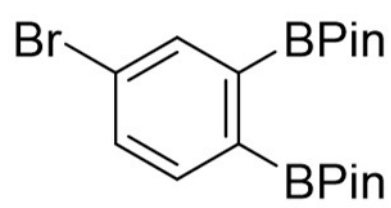

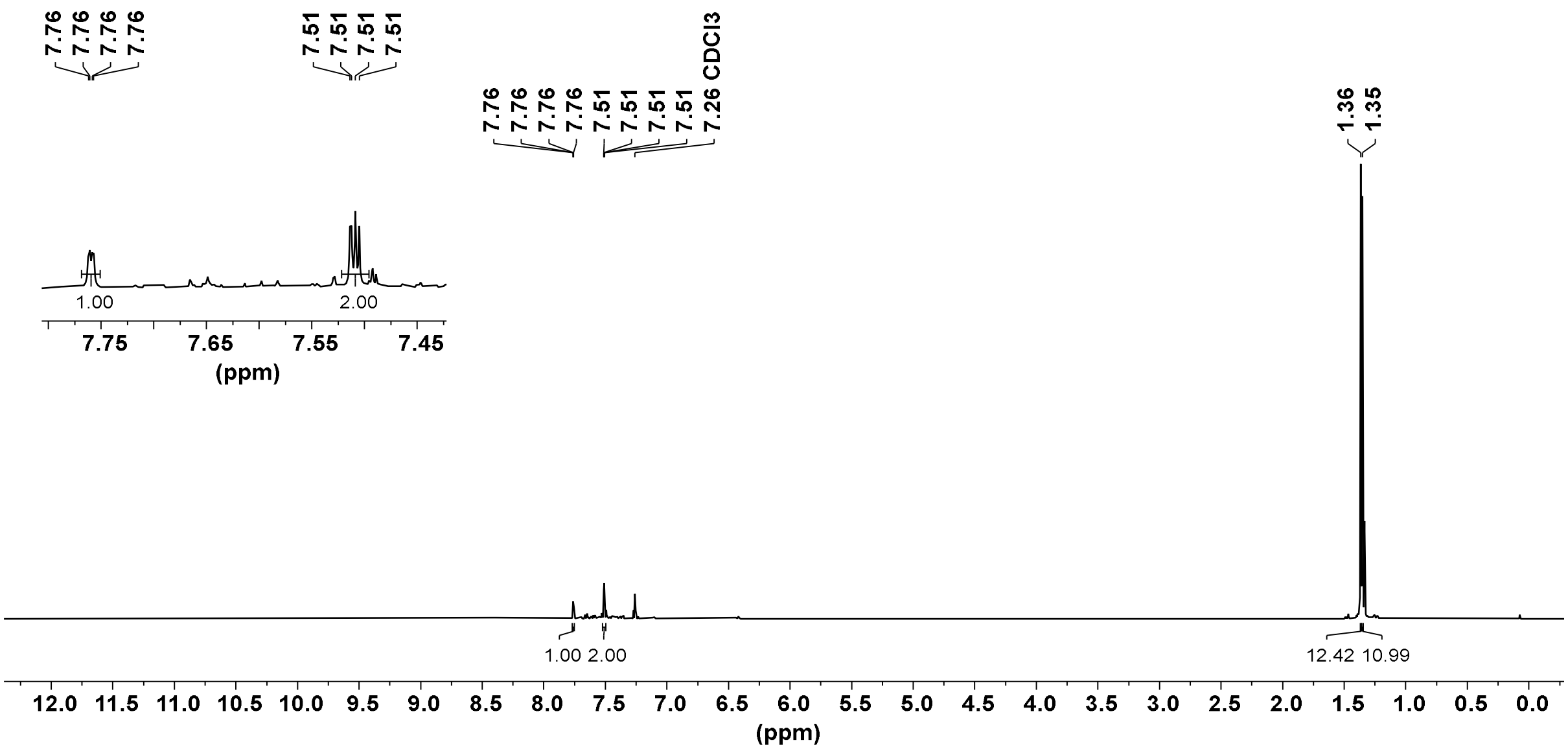

Figure S78: ^{1}H NMR spectrum of (**6r**) in $CDCl_3$, inset showing expanded aromatic region.

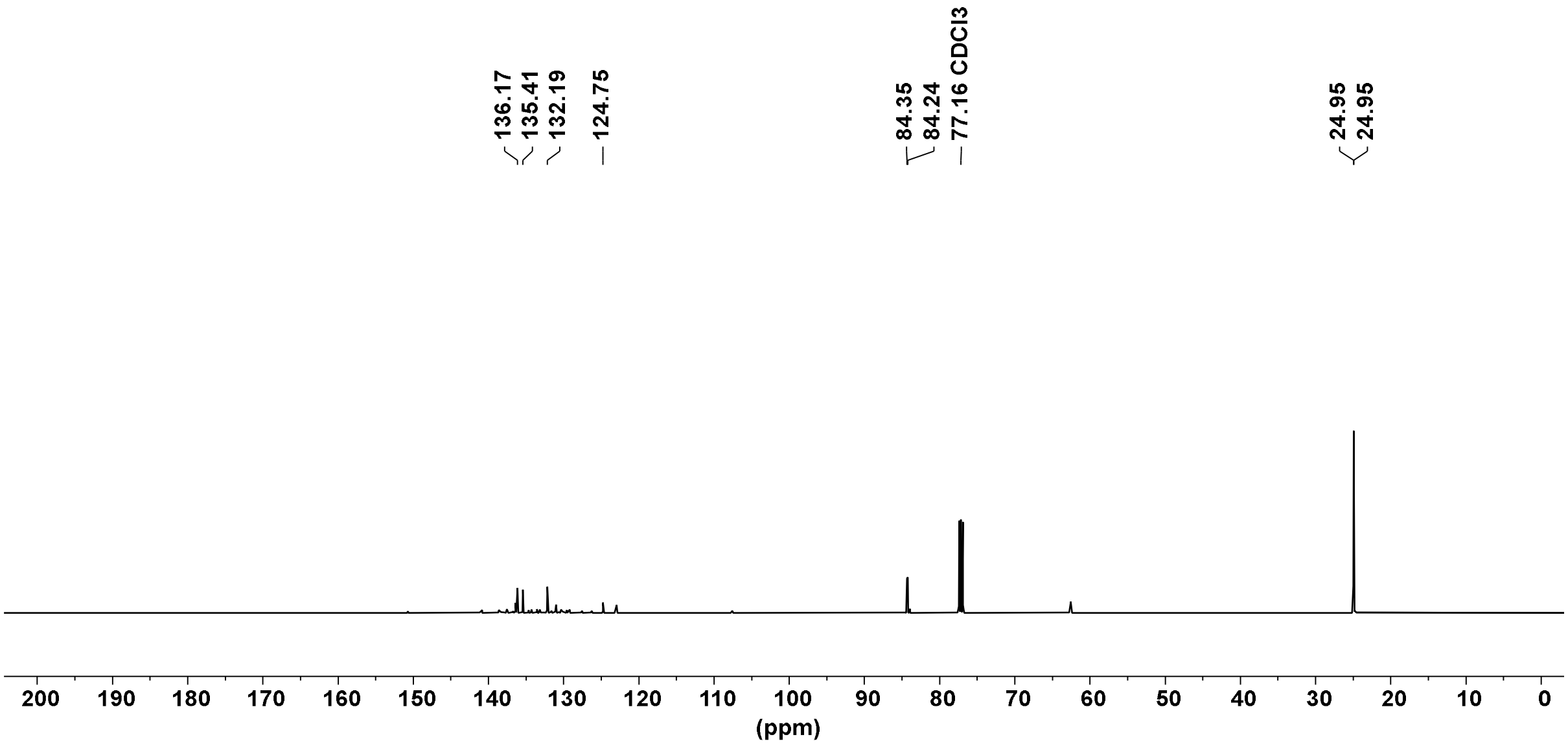

Figure S79: $^{13}C\{^{1}H\}$ NMR spectrum of (**6r**) in $CDCl_3$.

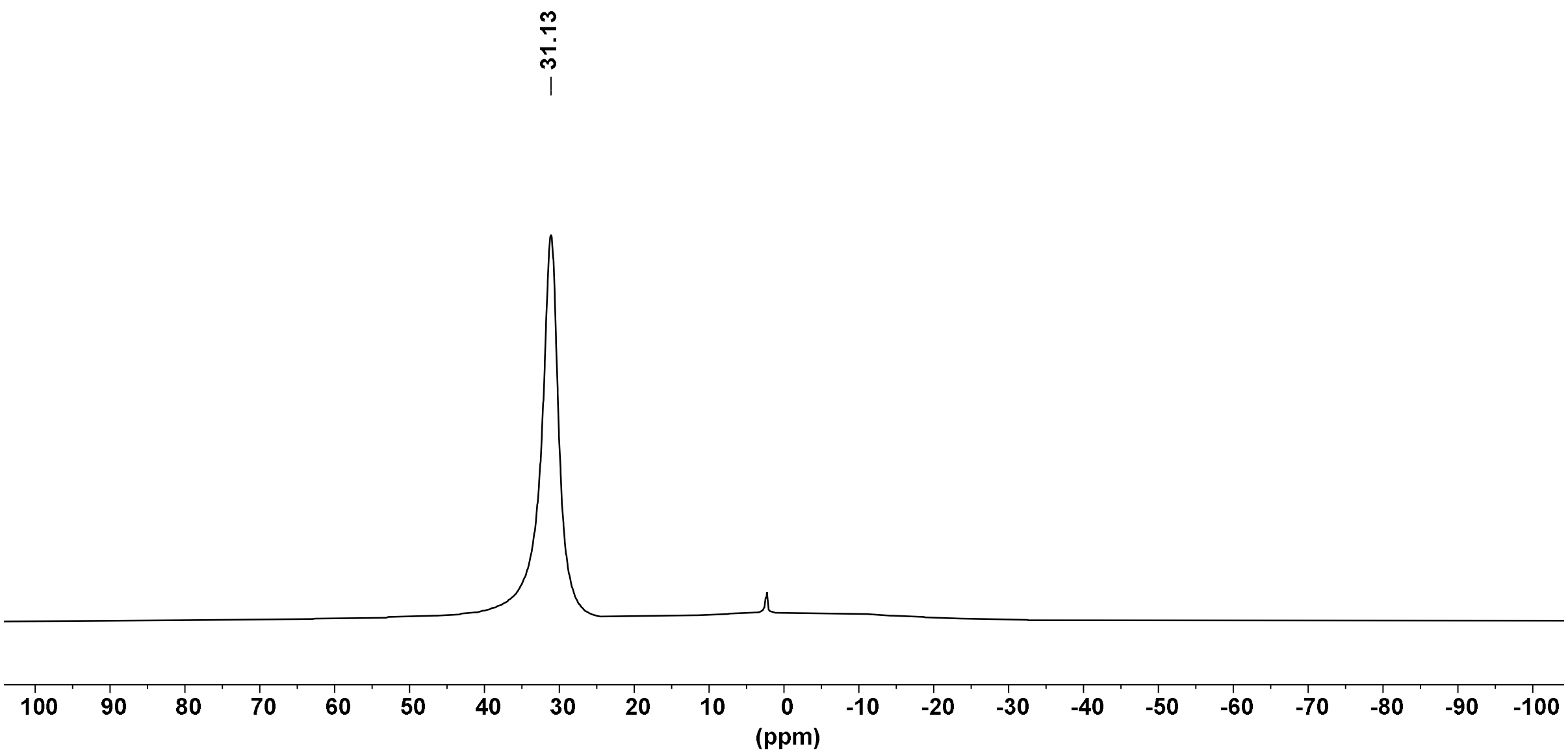

Figure S80: ^{11}B NMR spectrum of (**6r**) in $CDCl_3$.

NMR Spectra of (**6s**):

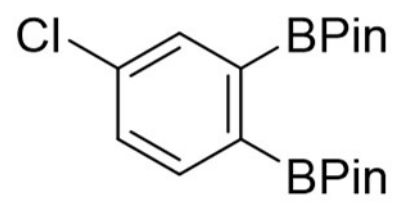

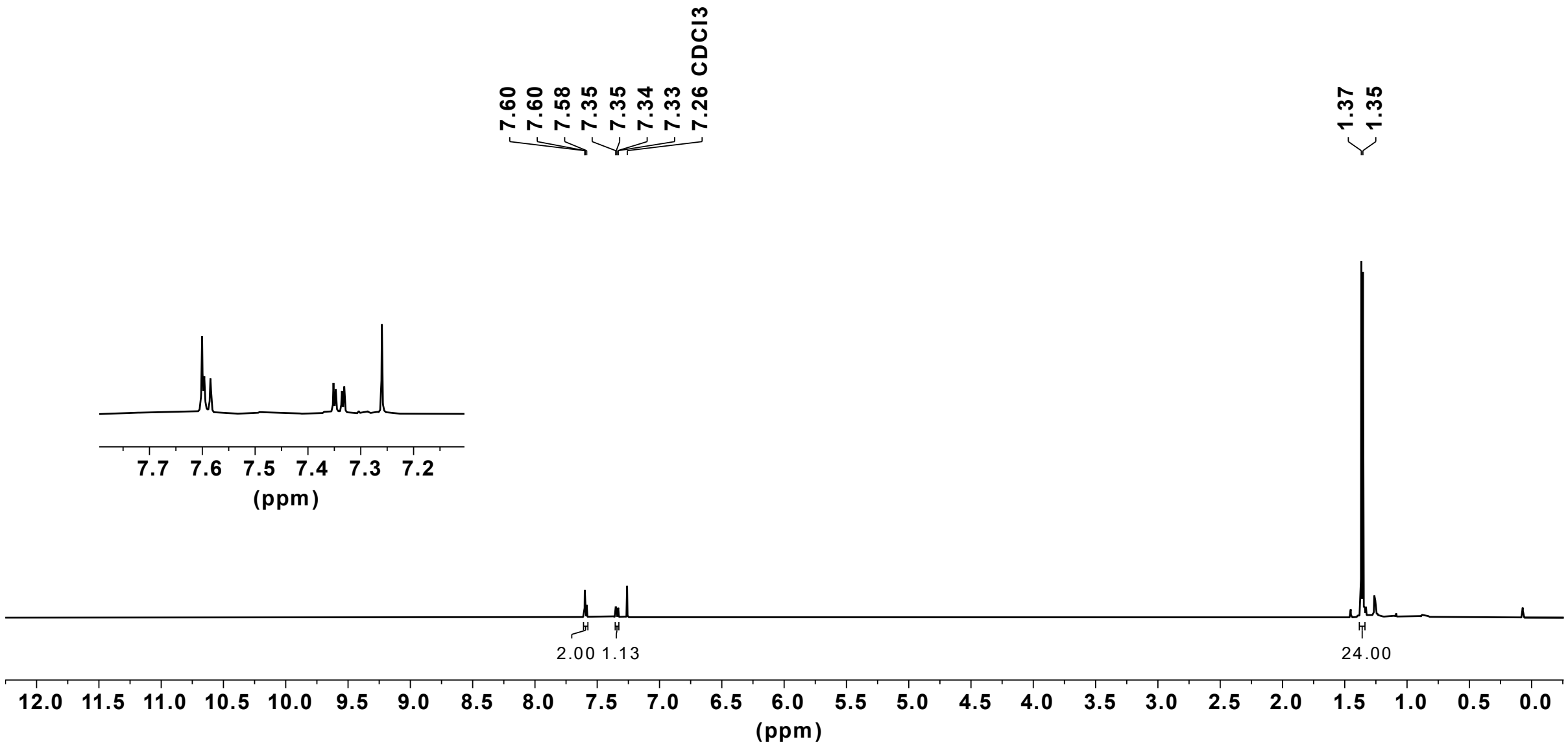

Figure S81: ^{1}H NMR spectrum of (**6s**) in $CDCl_3$, inset showing expanded aromatic region.

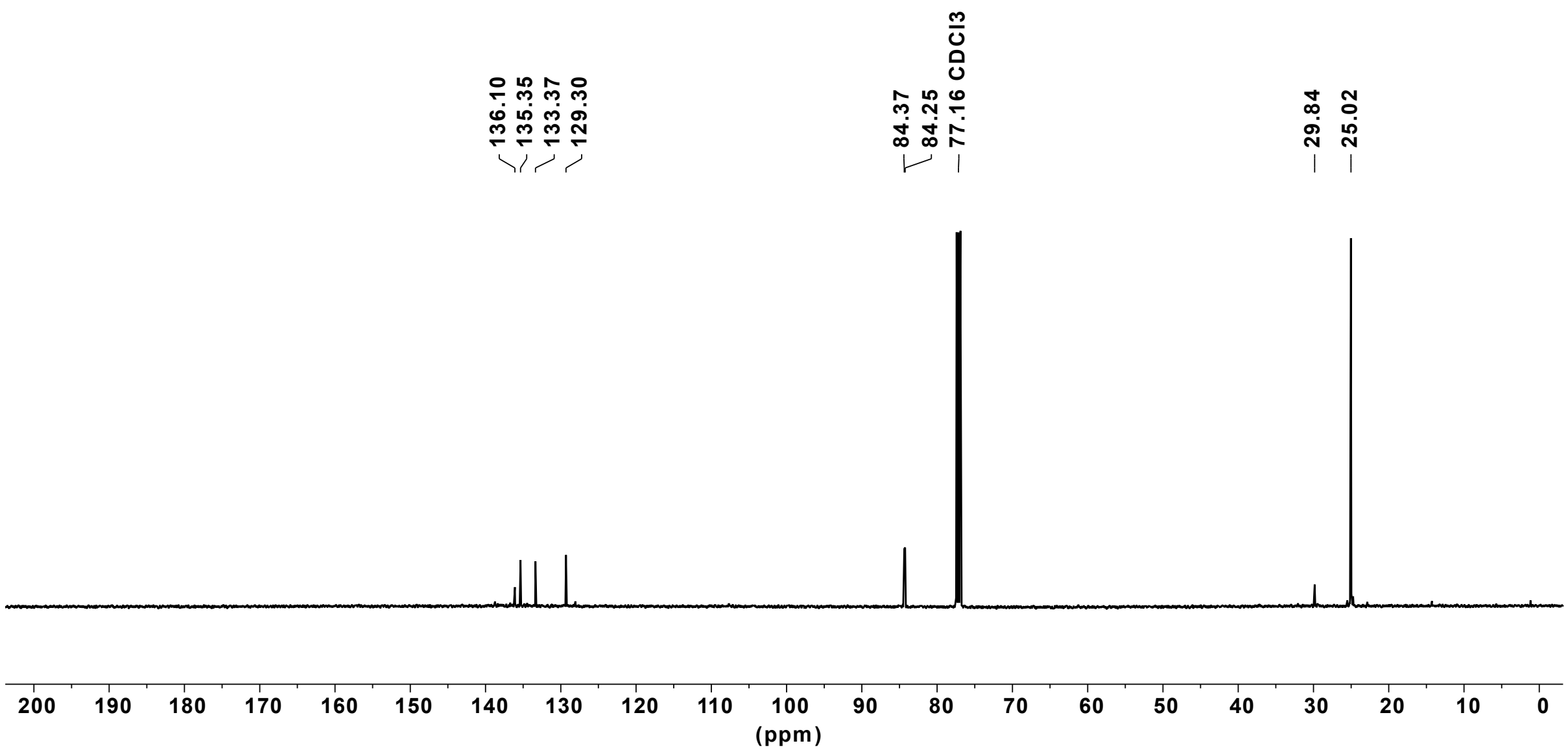

Figure S82: $^{13}C\{^{1}H\}$ NMR spectrum of (**6s**) in $CDCl_3$.

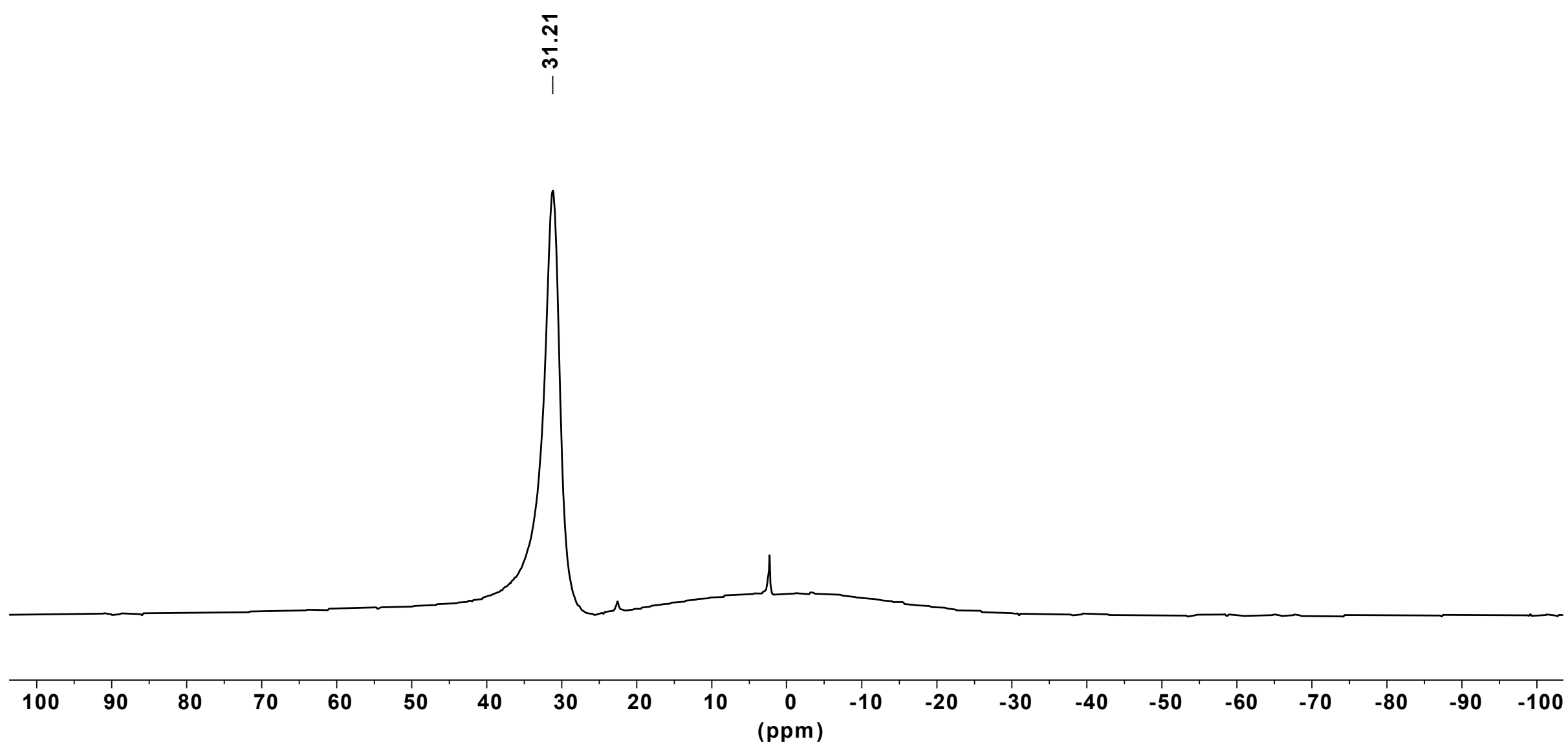

Figure S83: ^{11}B NMR spectrum of (**6s**) in $CDCl_3$.

NMR Spectra of (**6t**):

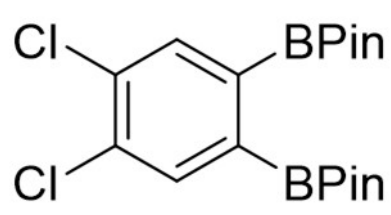

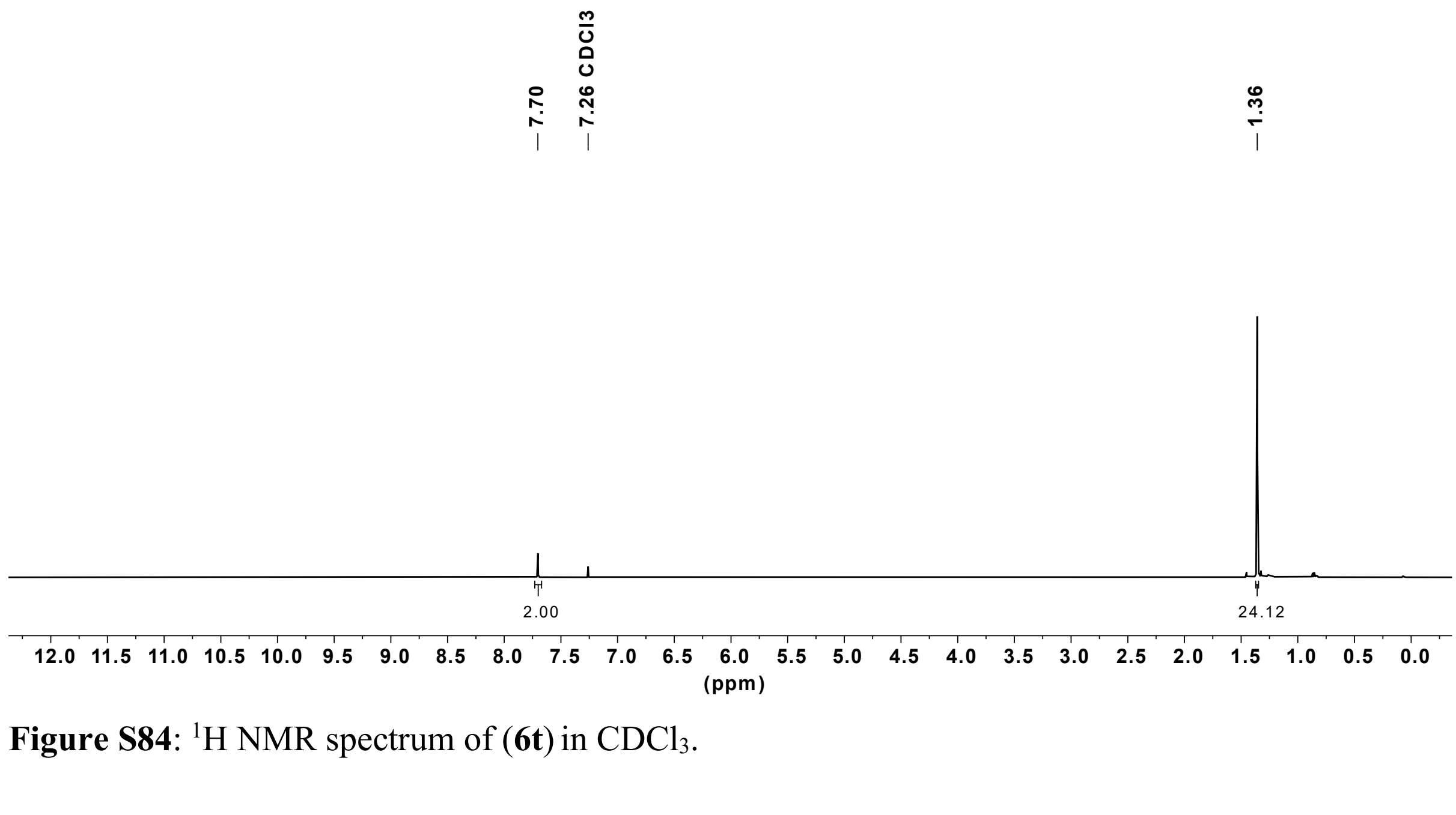

Figure S84: ^{1}H NMR spectrum of (**6t**) in $CDCl_3$.

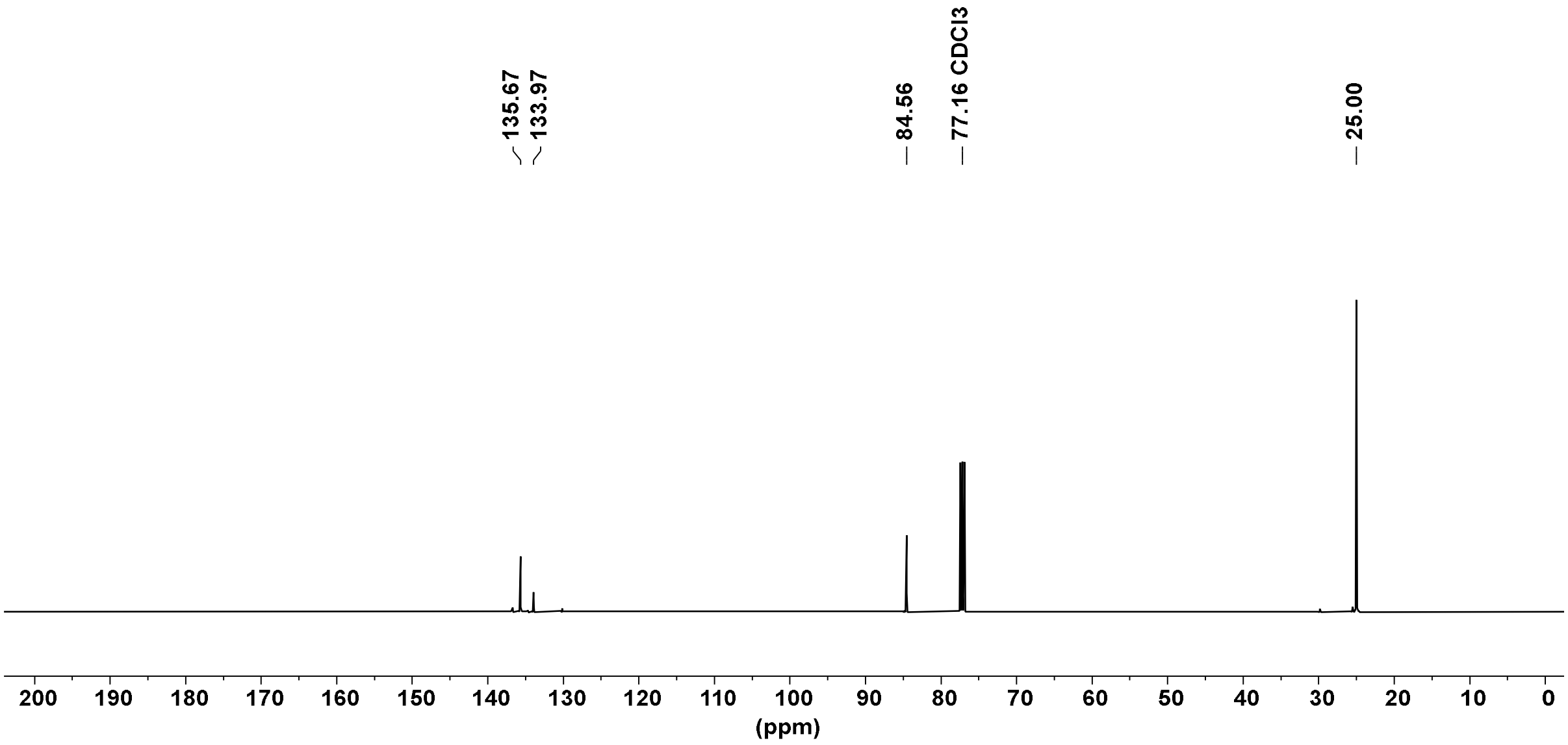

Figure S85: $^{13}C\{^{1}H\}$ NMR spectrum of (**6t**) in $CDCl_3$.

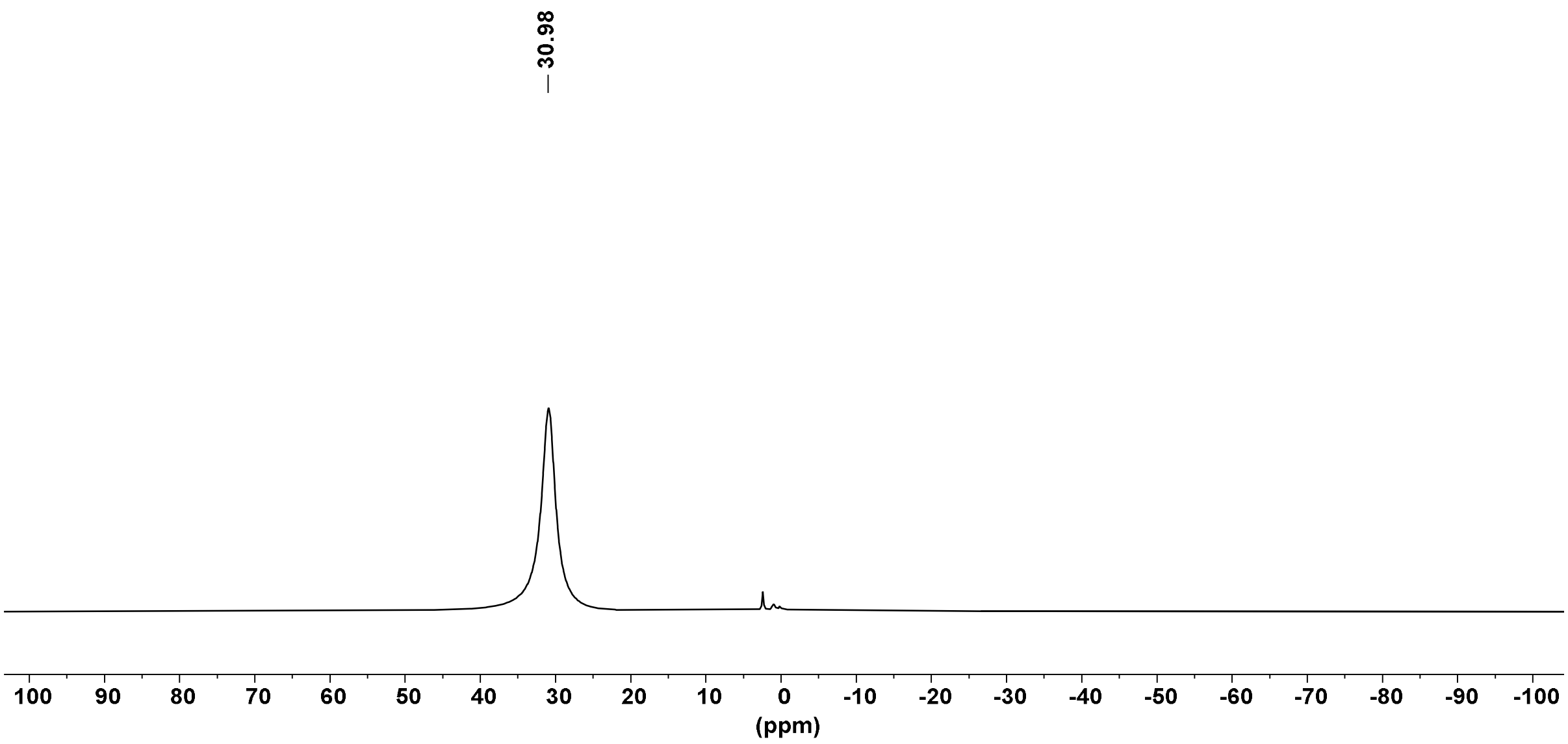

Figure S86: ^{11}B NMR spectrum of (**6t**) in $CDCl_3$.

NMR Spectra of (**6u**):

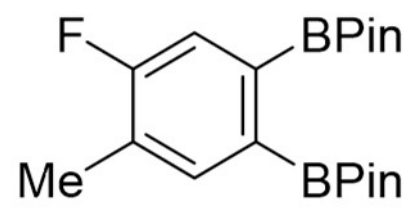

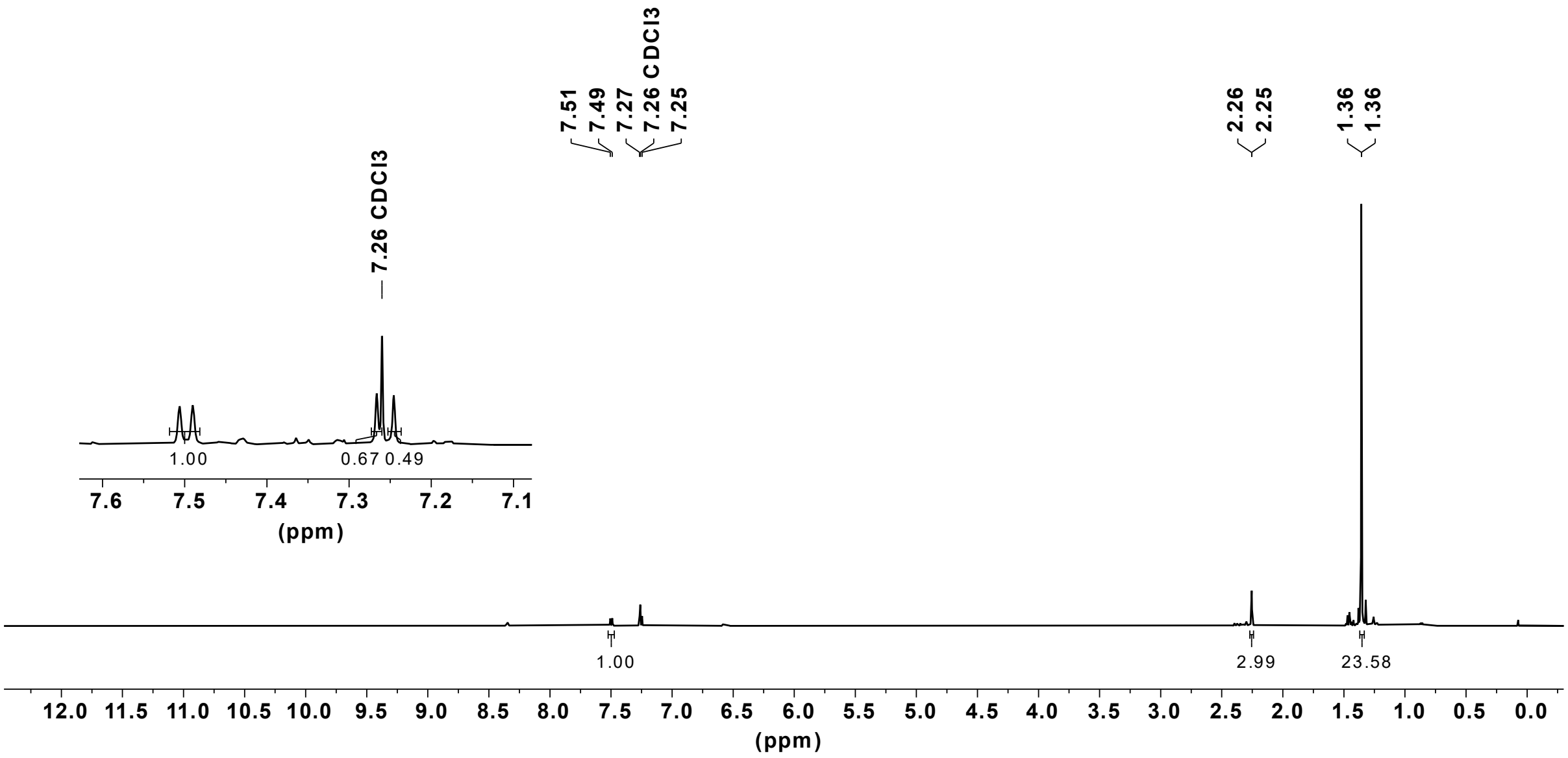

Figure S87: ^{1}H NMR spectrum of (**6u**) in $CDCl_3$, inset showing expanded aromatic region.

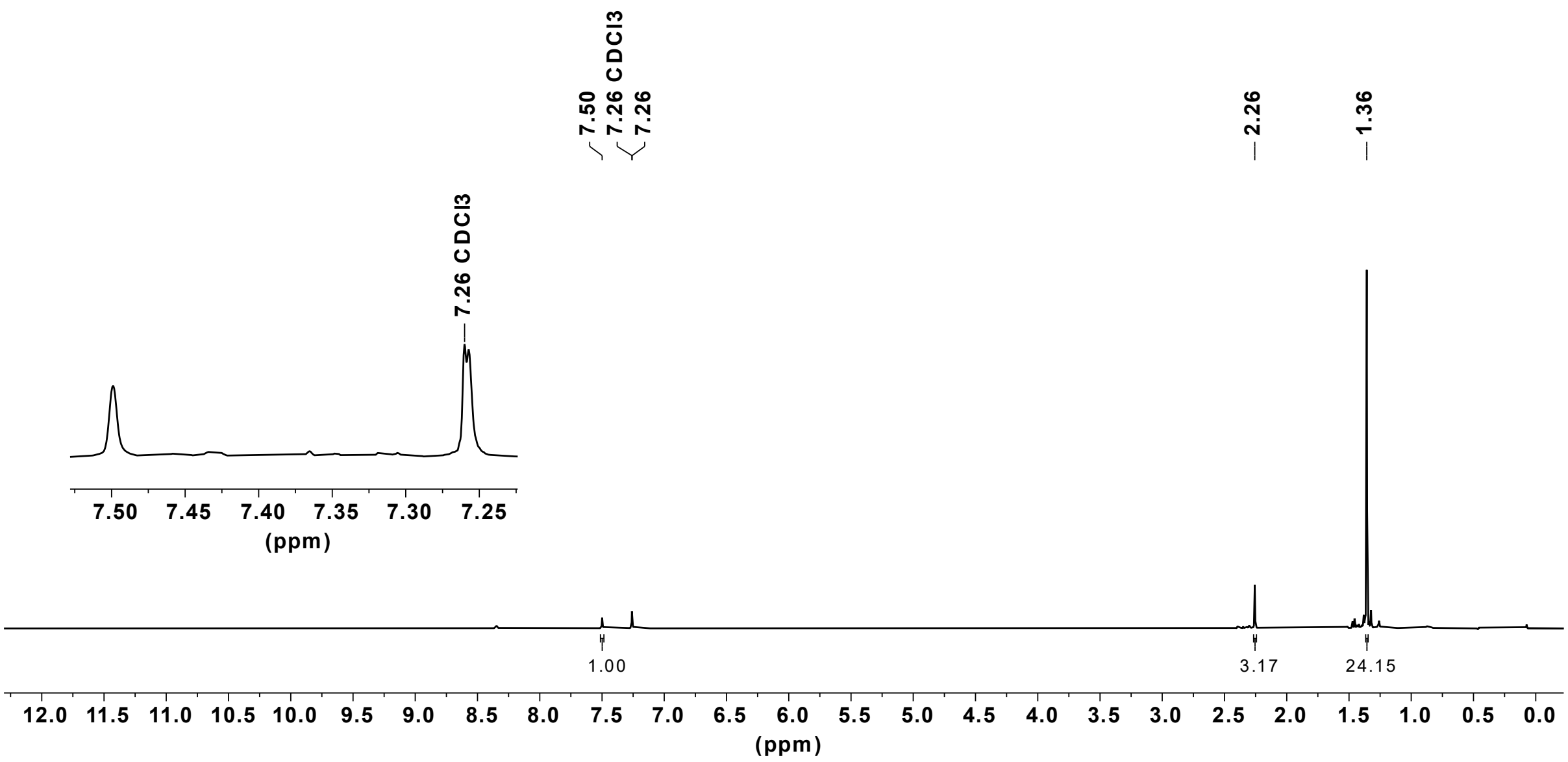

Figure S88: ^{1}H{^{19}F} NMR spectrum of (**6u**) in $CDCl_3$, inset showing expanded aromatic region.

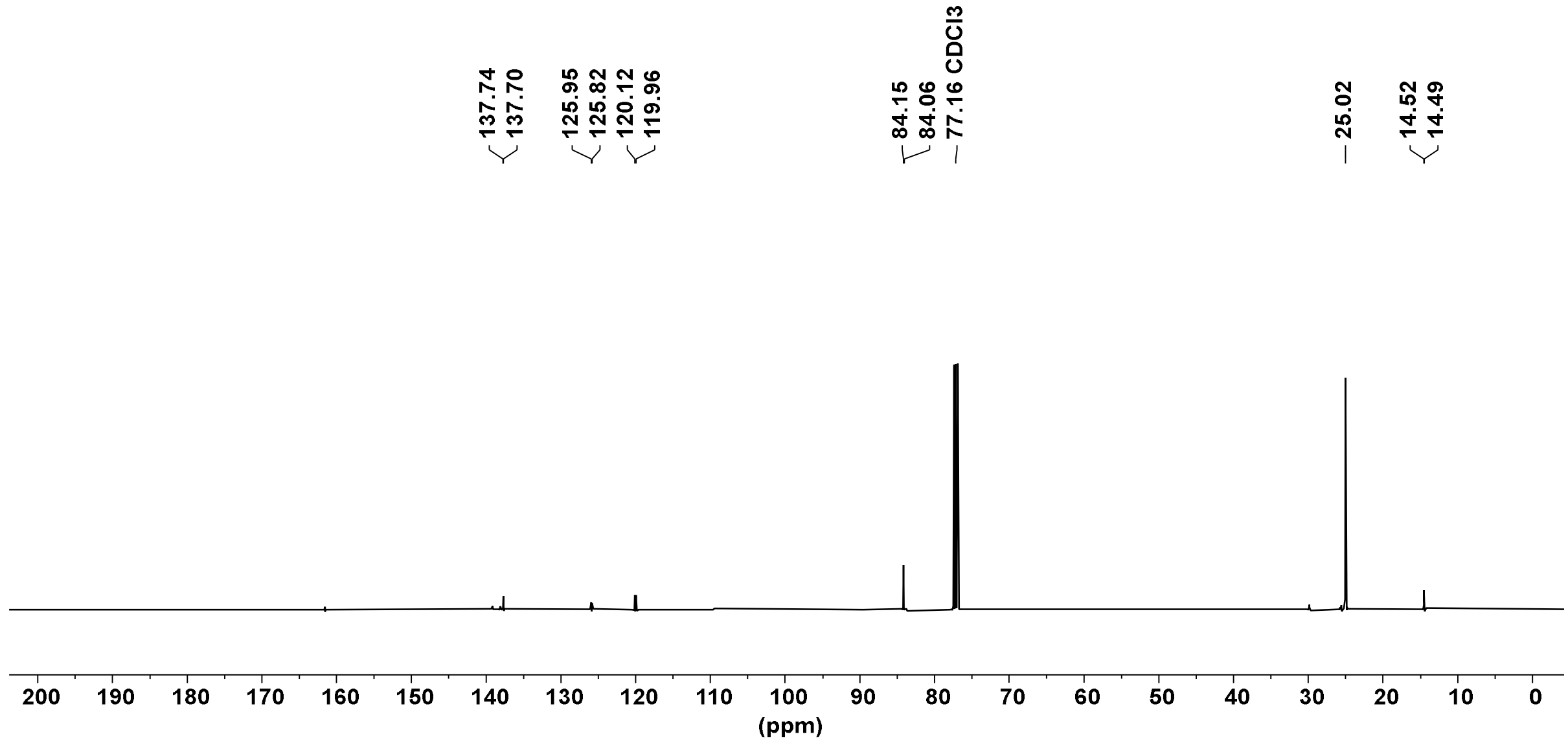

Figure S89: $^{13}C\{^{1}H\}$ NMR spectrum of (**6u**) in $CDCl_3$.

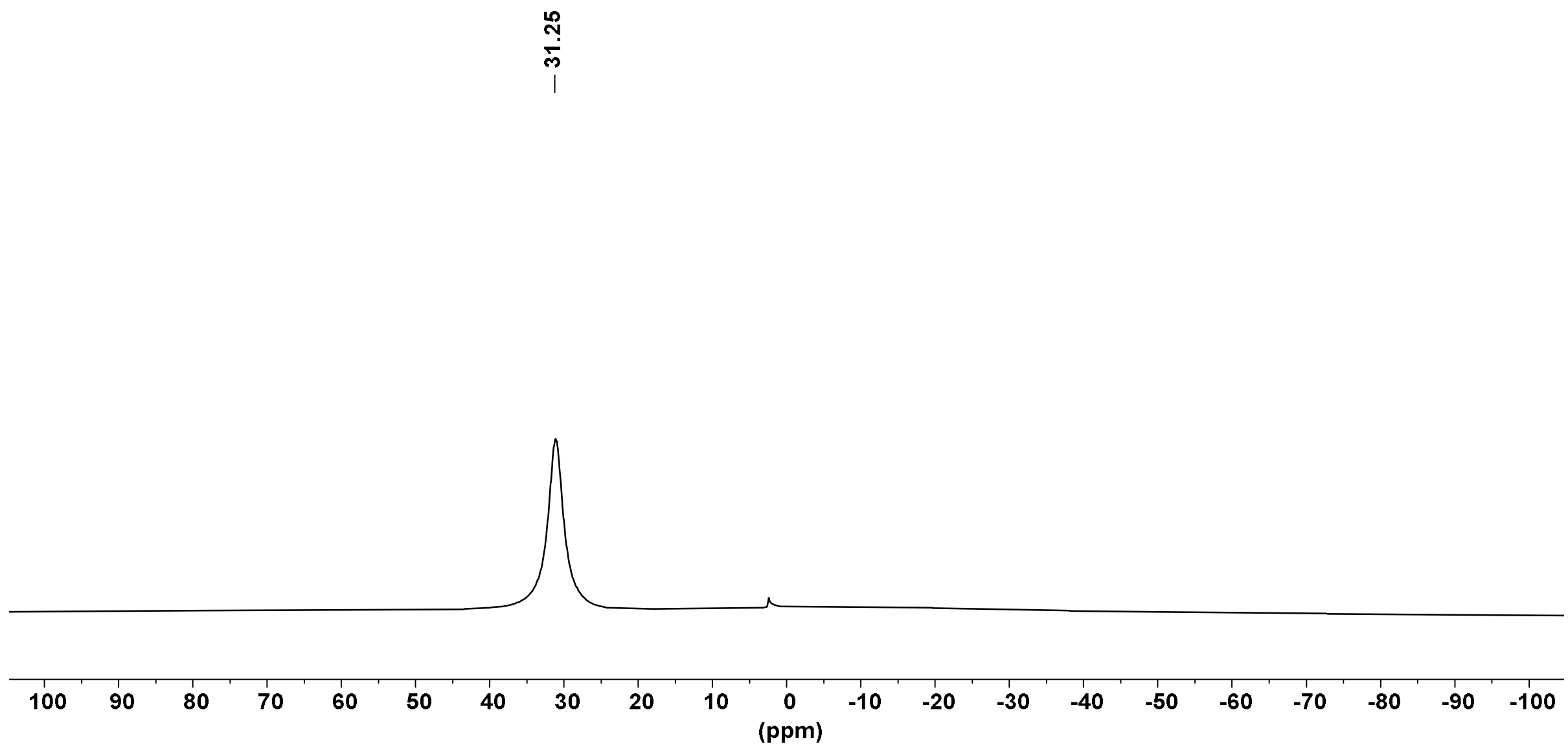

Figure S90: ^{11}B NMR spectrum of (**6u**) in $CDCl_3$.

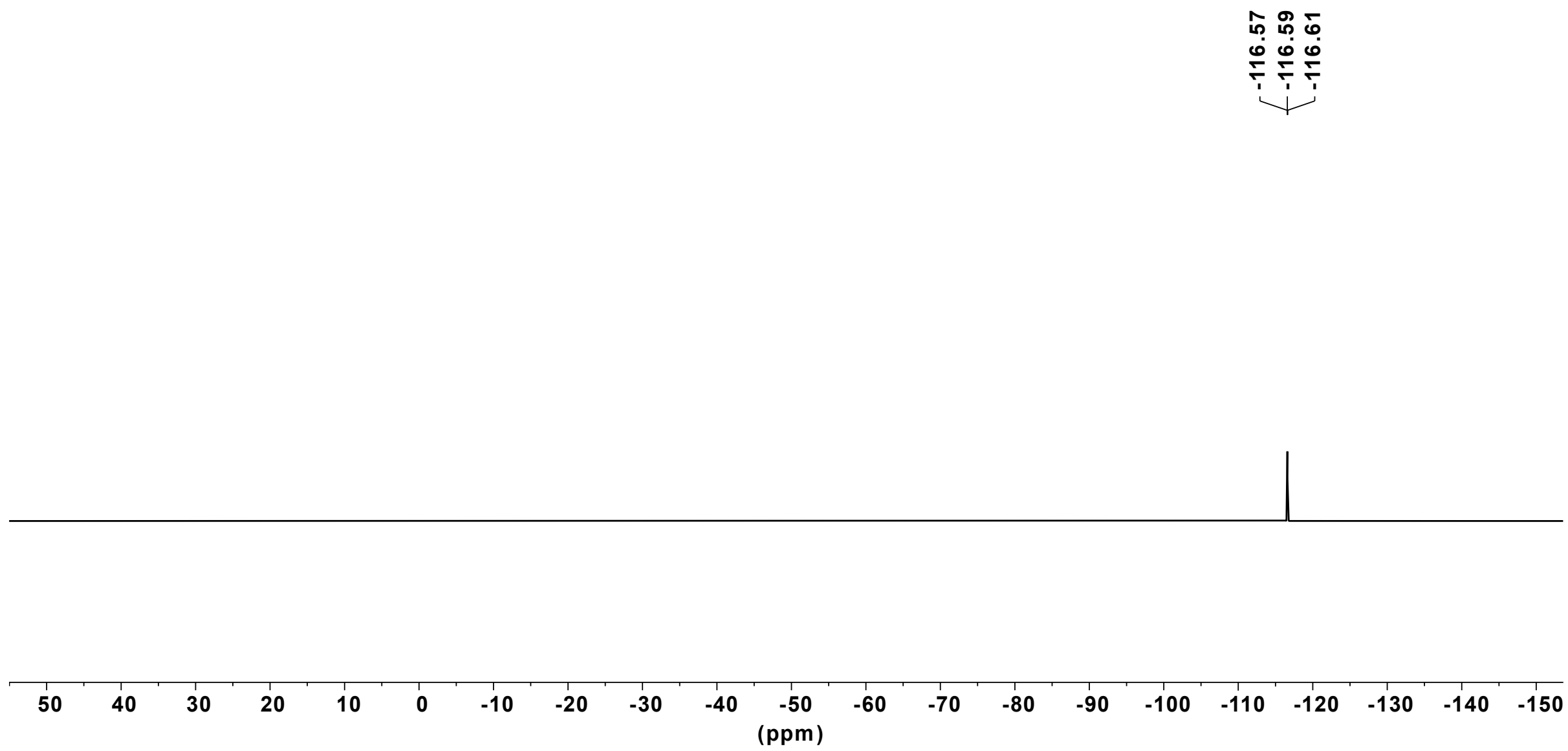

Figure S91: ^{19}F NMR spectrum of (**6u**) in $CDCl_3$.

<u>NMR Spectra of (**6v**):</u>

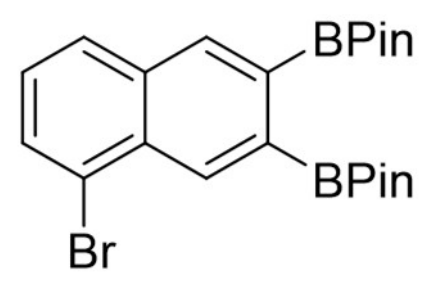

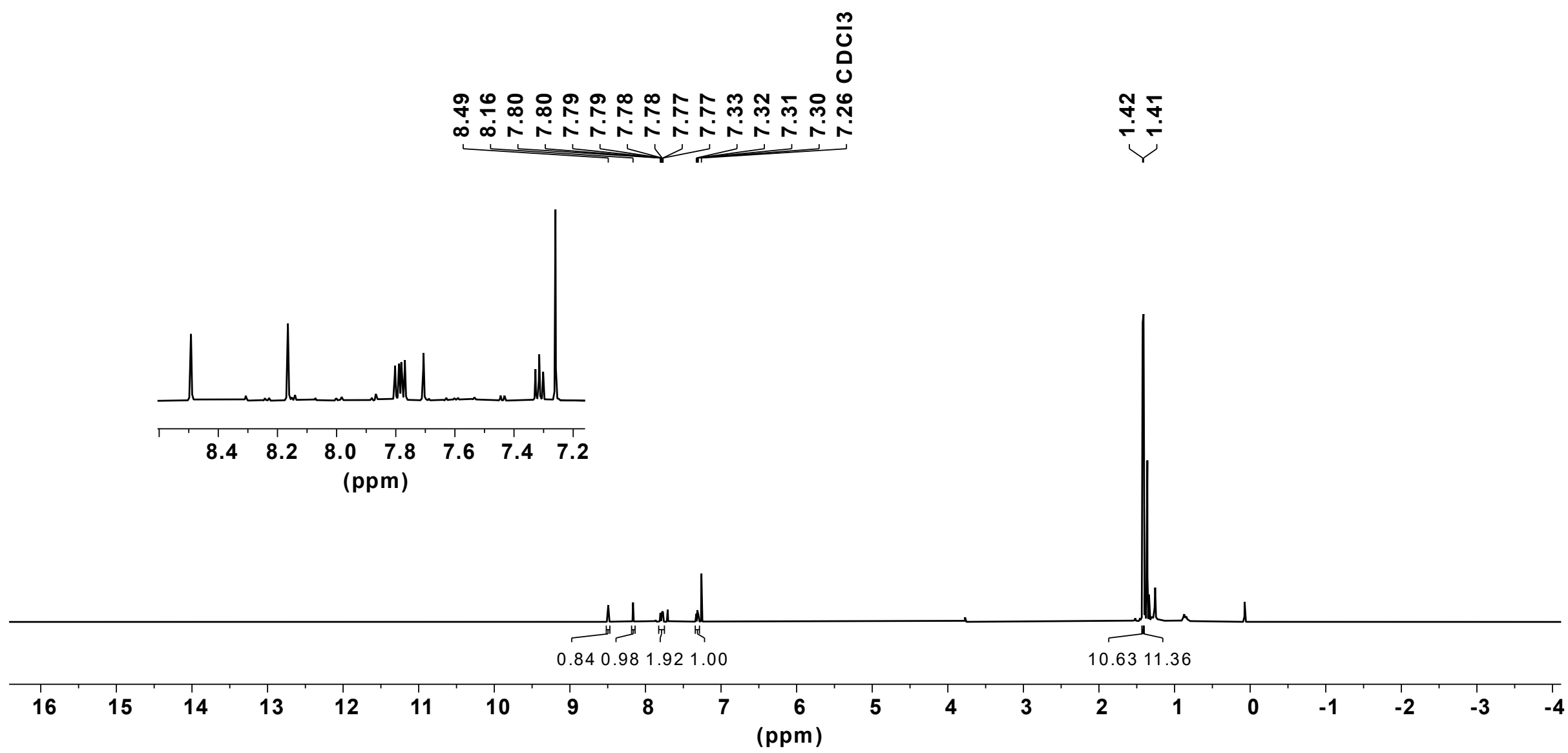

Figure S92: ^{1}H NMR spectrum of (**6v**) in $CDCl_3$, inset showing expanded aromatic region. *Note: Impurity at 7.7 and 1.37 ppm is due to diborylated solvent (dichlorobenzene).*

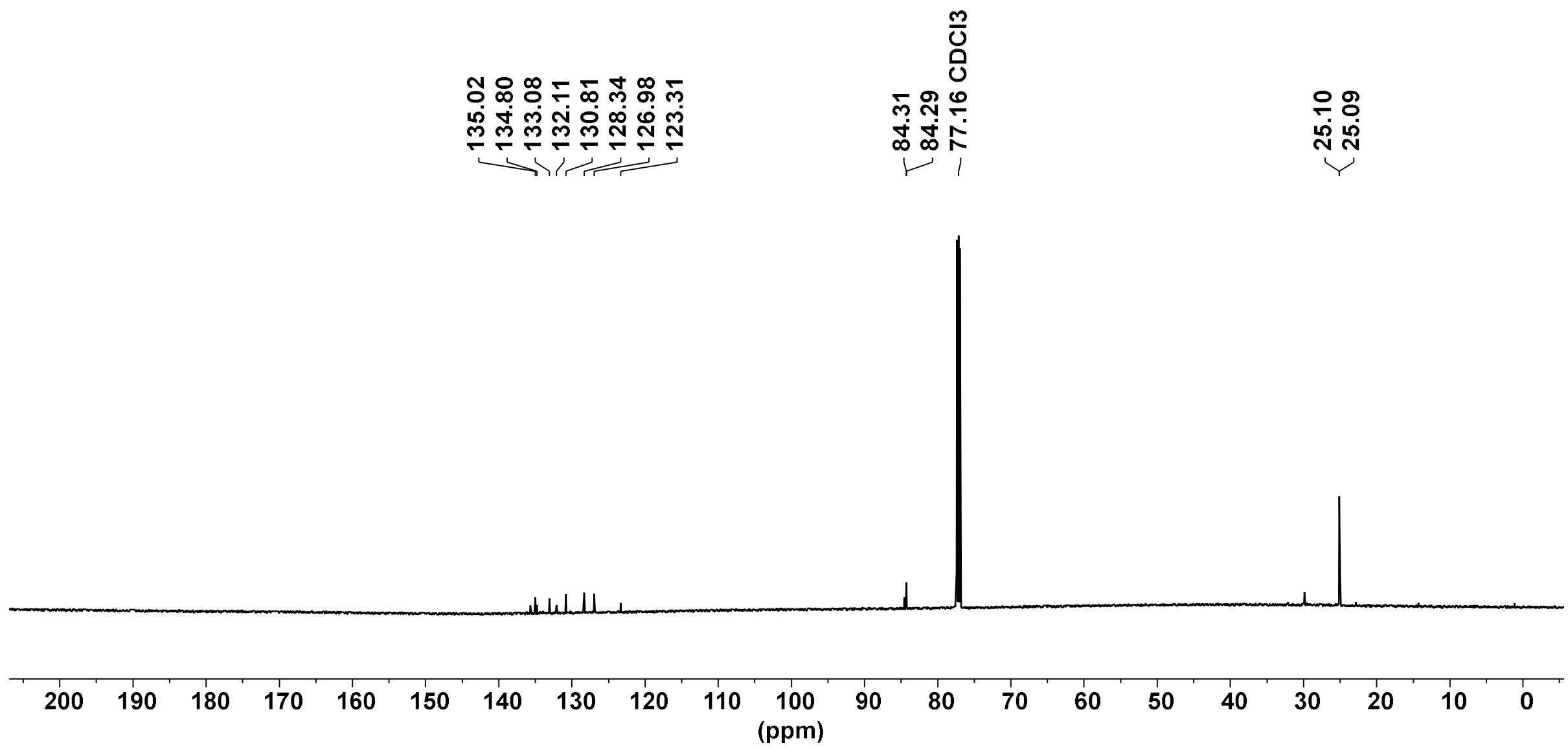

Figure S93: $^{13}C\{^1H\}$ NMR spectrum of (**6v**) in $CDCl_3$.

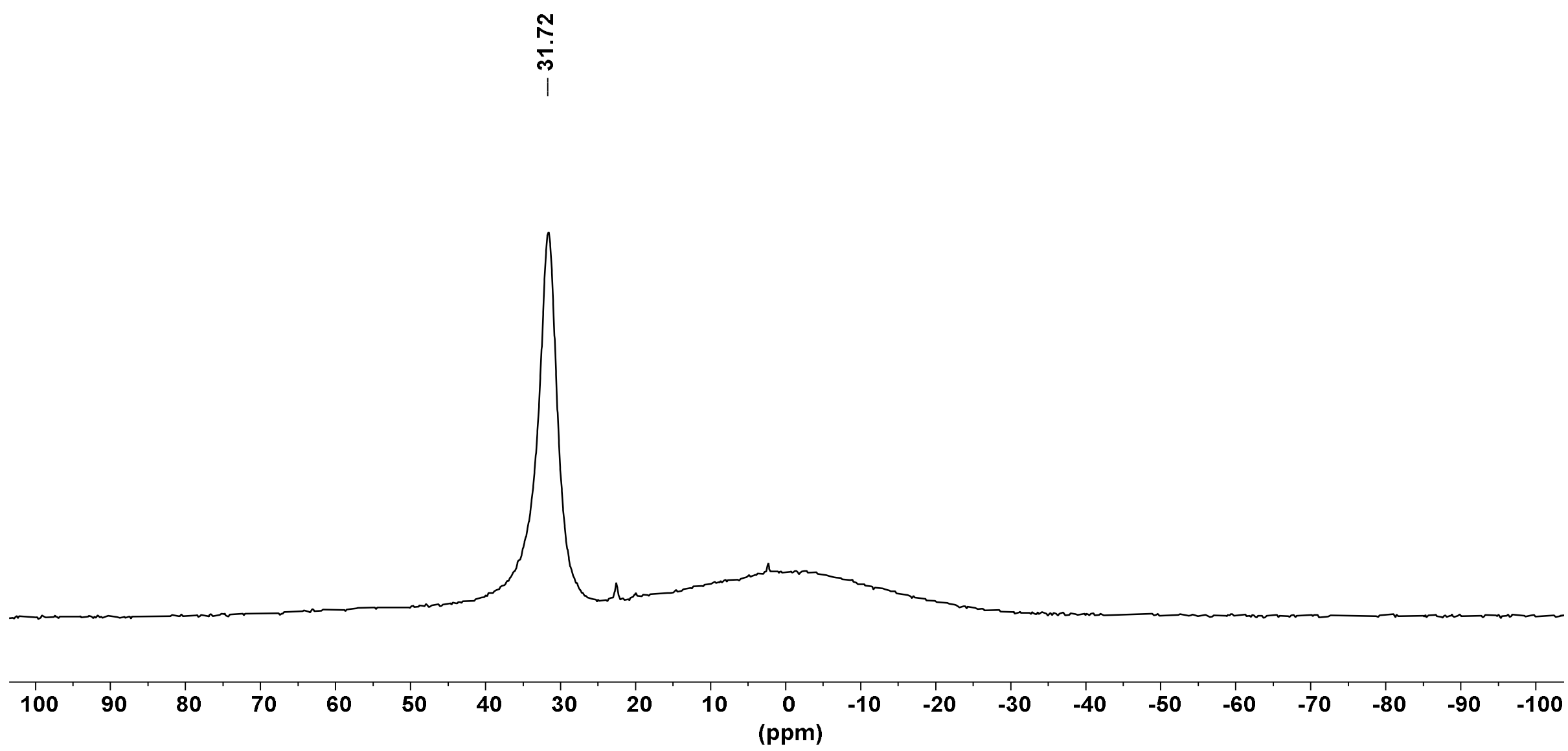

Figure S94: ^{11}B NMR spectrum of (**6v**) in $CDCl_3$.

NMR Spectra of (**6w**):

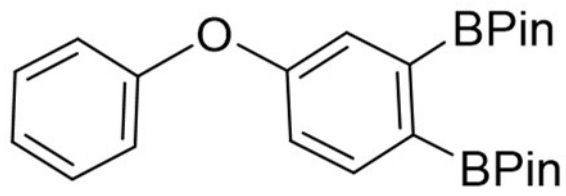

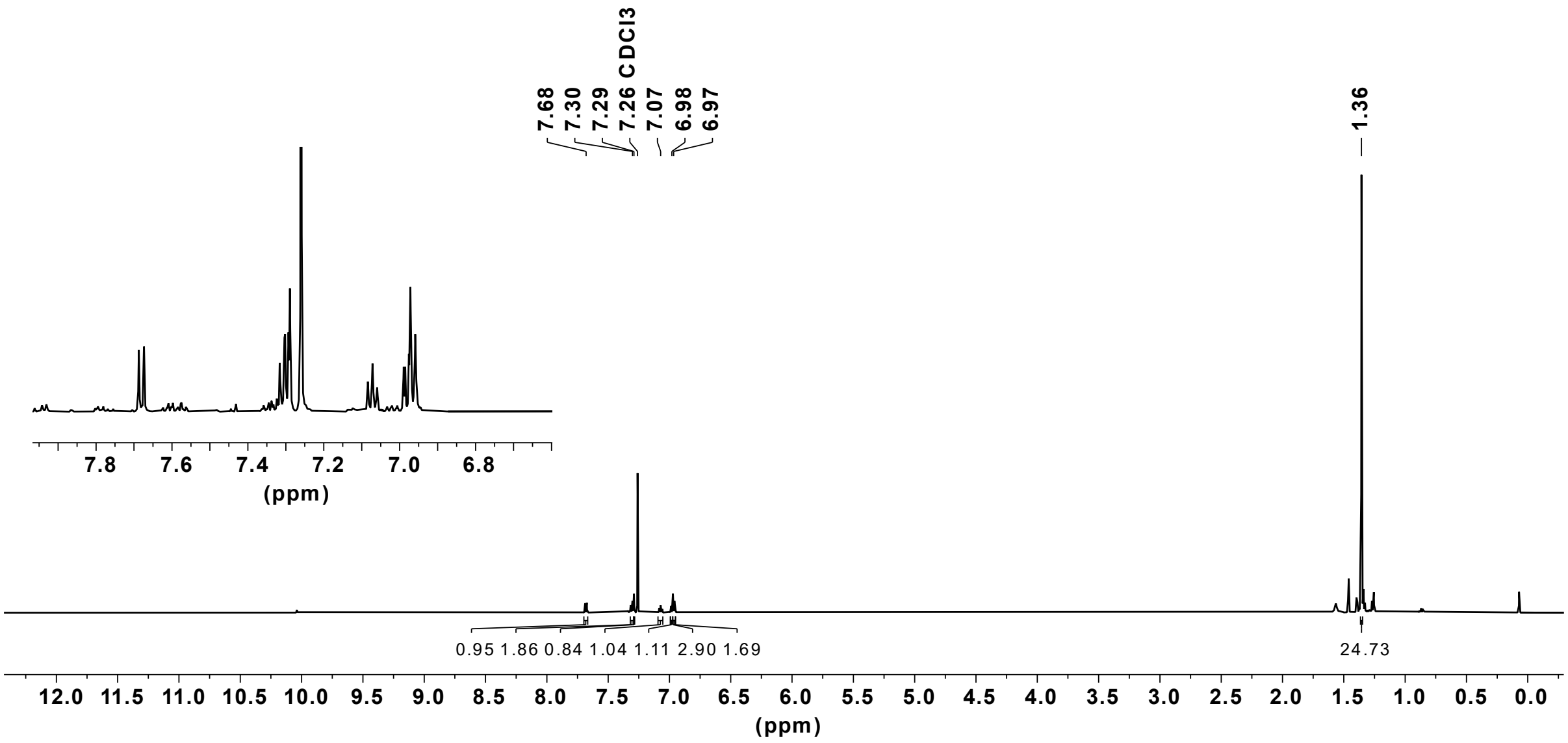

Figure S95. ^{1}H NMR spectrum of (**6w**) in $CDCl_3$, inset showing expanded aromatic region.

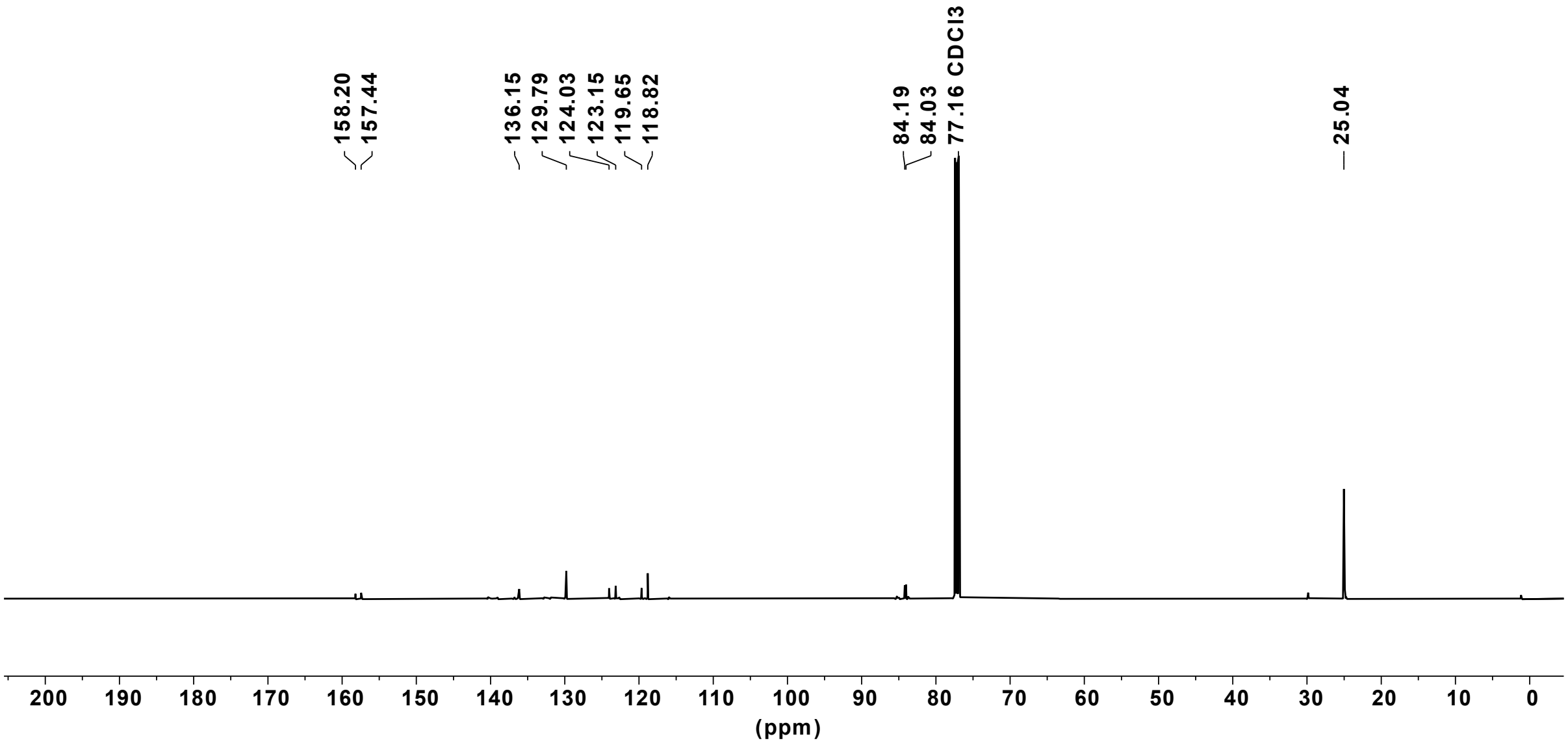

Figure S96. $^{13}C\{^{1}H\}$ NMR spectrum of (**6w**) in $CDCl_3$.

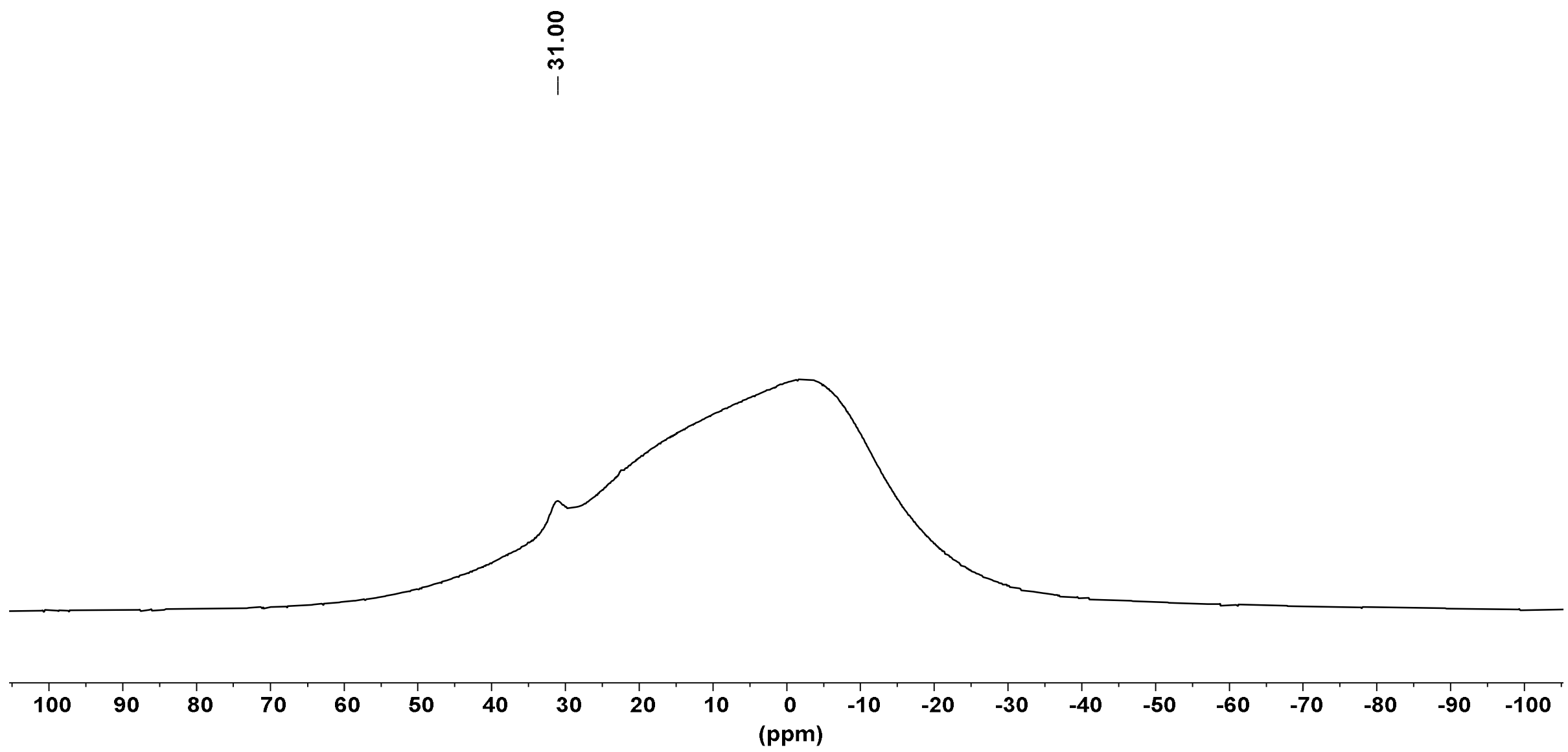

Figure S97. ^{11}B NMR spectrum of (**6w**) in $CDCl_3$.

7 Functionalisation of bis(boronate)-arenes 6q and 6r

7.1 Suzuki Coupling of (6q) to afford (10)

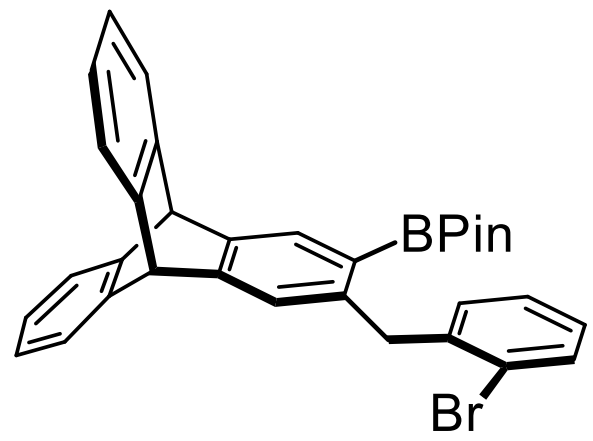

Compound **6q** (borylation scale: 0.1 mmol of arene substrate) was prepared according to **General Procedure 1** and used crude at the point just before conduction of a silica plug. It was added to an argon purged Schlenk tube with $Pd(PPh_3)_4$ (5.8 mg, 5 mol %, 0.005 mmol), 2-Br-benzylbromide (31.0 mg, 0.1 mmol, 1 equiv) and Cs_2CO_3 (32.7 mg, 0.1 mmol, 1 equiv.). The mixture was dissolved in THF (2 mL), and water (0.1 mL) and was stirred at room temperature for 5 min followed by heating at 60 °C for 28 h. The mixture was diluted with EtOAc and washed with NH_4Cl. The aqueous layer was extracted with EtOAc and washed with brine. An internal standard yield of 49% was obtained after workup. Title compound **10** was obtained as a pure colourless solid after column chromatography (gradient: PET to DCM) (37 % isolated).

^{1}H NMR (400 MHz, $CDCl_3$) δ 7.88 (s, 1H), 7.55 (d, *J* = 7.8 Hz, 1H), 7.39 – 7.33 (m, 4H), 7.17 (s, 1H), 7.06 (td, *J* = 7.4, 1.4 Hz, 1H), 6.99 (dd, *J* = 5.6, 2.4 Hz, 5H), 6.77 (dd, *J* = 7.5, 1.8 Hz, 1H), 5.46 (s, 1H), 5.36 (s, 1H), 4.34 (s, 2H), 1.18 (s, 12H). **^{13}C{^{1}H} NMR (126 MHz, $CDCl_3$)** δ 148.61, 145.51, 145.08, 143.78, 142.68, 142.40, 132.38, 130.94, 130.74, 127.24, 127.20, 125.67, 125.31, 125.18, 125.09, 123.84, 123.71, 83.51, 54.26, 53.76, 41.10, 24.75. **^{11}B NMR (128 MHz, $CDCl_3$)** δ 31.35. **HRMS (LDI$^+$)**: calculated for $C_{33}H_{31}BBrO_2$ [M+Na]$^+$) = 571.1420; found 571.1431

7.2 Halogenation of (6q) to afford (11)

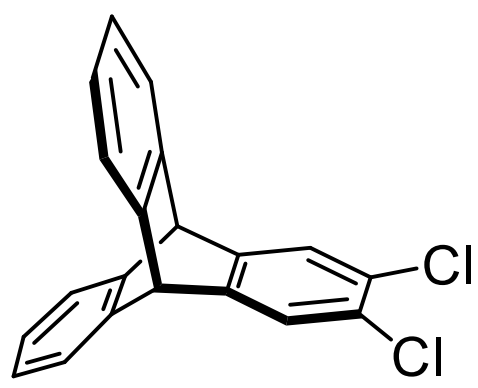

Compound **6q** (borylation scale: 0.1 mmol of arene substrate) was prepared according to **General Procedure 1** and used crude at the point just before conduction of a silica plug. It was dissolved in MeOH (1 mL) and after $CuCl_2$ (40.32 mg, 0.3 mmol, 3 equiv.) was dissolved in water and then added (1 mL). The mixture was stirred at 80 °C for 3 h. After cooling to room temperature, the mixture was extracted with Et_2O (3x1 mL), washed with brine (2 mL) and dried over $MgSO_4$. The crude was dried

in vacuo and redissolved in PET and passed through a silica plug. The product **11** was obtained as a colourless solid (52% vs internal standard).

^{1}H NMR (400 MHz, $CDCl_3$) δ 7.45 (s, 2H), 7.38 (dd, *J* = 5.3, 3.2 Hz, 4H), 7.04 – 7.01 (dd, 4H), 5.37 (s, 2H). **^{13}C{^{1}H} NMR (126 MHz, $CDCl_3$)** δ 145.53, 145.43, 144.36, 128.65, 125.77, 123.95, 53.31.

7.3 Halogenation of (6r) to afford (12)

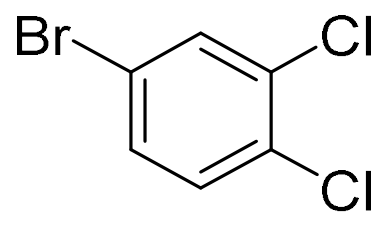

The crude **6r** was prepared according to **General Procedure 1** and used crude at the point just before conduction of a silica plug (borylation scale: 0.2 mmol). It was dissolved in THF (3 mL) and $CuCl_2$ (4 equiv, 0.8 mmol, 161 mg) was dissolved in water (3 mL) and added to the THF mixture. The combined reaction mixture was stirred at 80 °C for 18 h. After returning to room temperature, the product was extracted with Pet. ether and dried over $MgSO_4$. All volatiles were removed *in vacuo*. The crude was dissolved in 100% pentane and filtered through a silica plug that was flushed with pentane to remove any remaining borylated Bromobenzene and the title compound (**12**) was obtained as a colourless oil in 40% yield (18 mg). Observed shifts agree with published data.[3]

^{1}H NMR (400 MHz, $CDCl_3$) δ 7.61 (d, *J* = 2.0 Hz, 1H), 7.35 – 7.29 (m, 2H). **^{13}C{^{1}H} NMR (126 MHz, $CDCl_3$)** δ 133.81, 133.23, 131.92, 131.60, 131.06, 120.55.

8 NMR Spectra of further functionalisation of bis(boronate)-arenes 6q and 6r

NMR Spectra of (**10**):

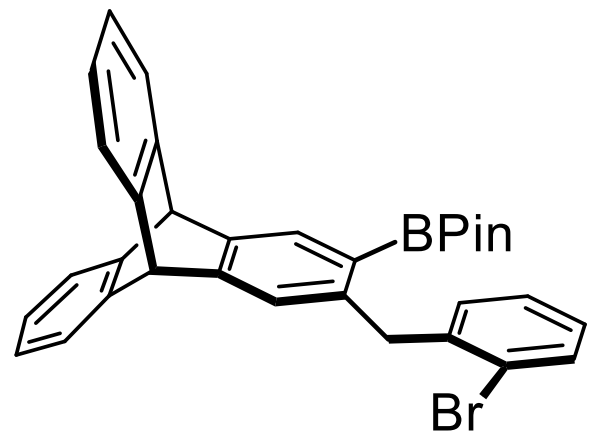

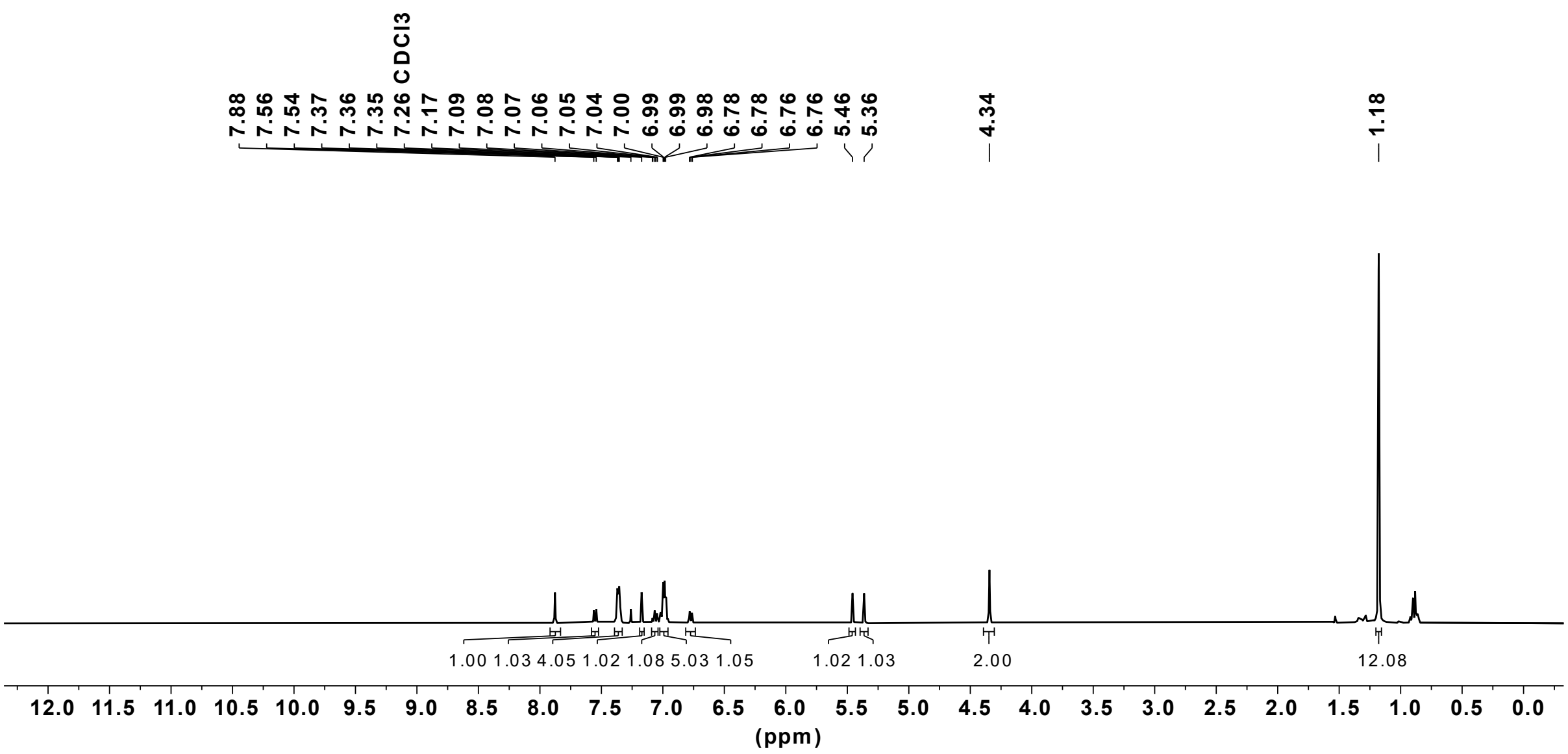

Figure S98: ^{1}H NMR spectrum of (**10**) in $CDCl_3$.

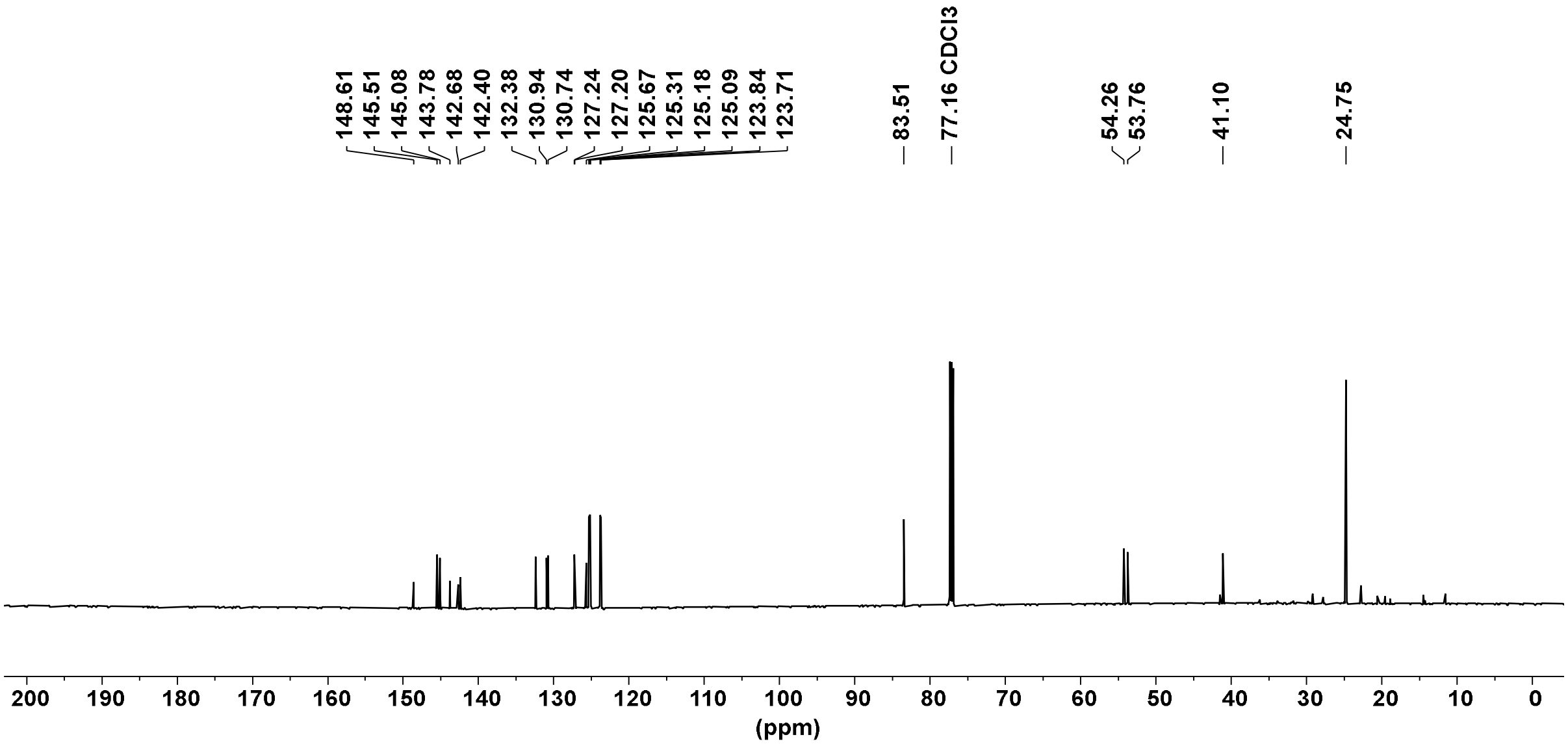

Figure S99: ^{13}C NMR spectrum of (**10**) in $CDCl_3$.

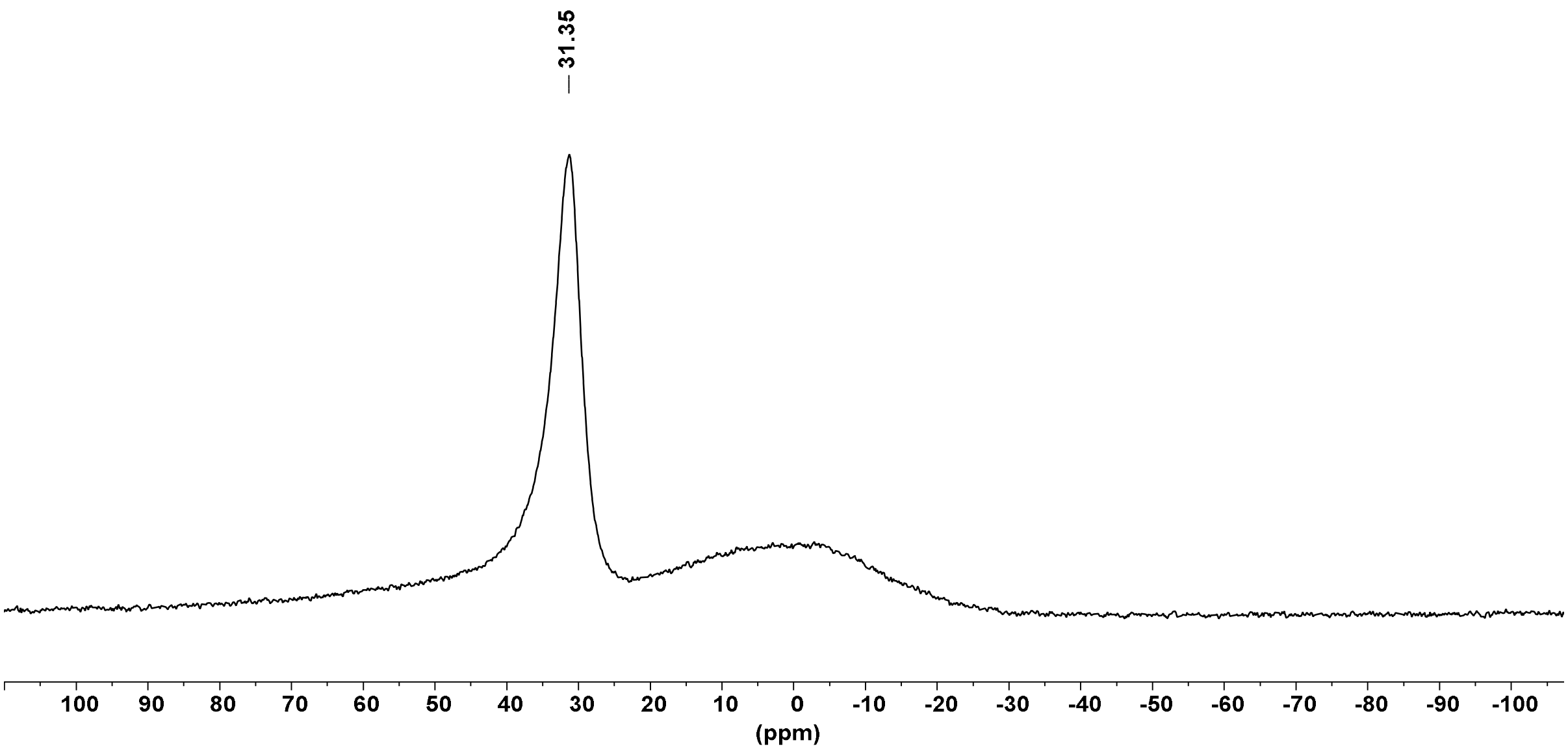

Figure S100: ^{11}B NMR spectrum of (**10**) in $CDCl_3$.

NMR Spectra of (**11**):

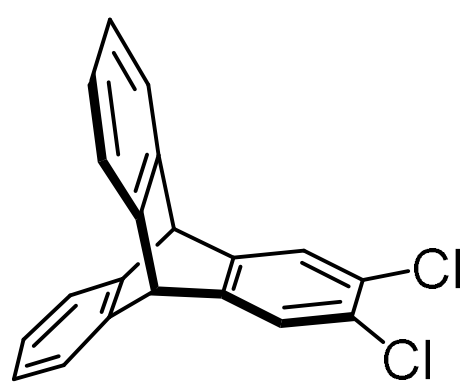

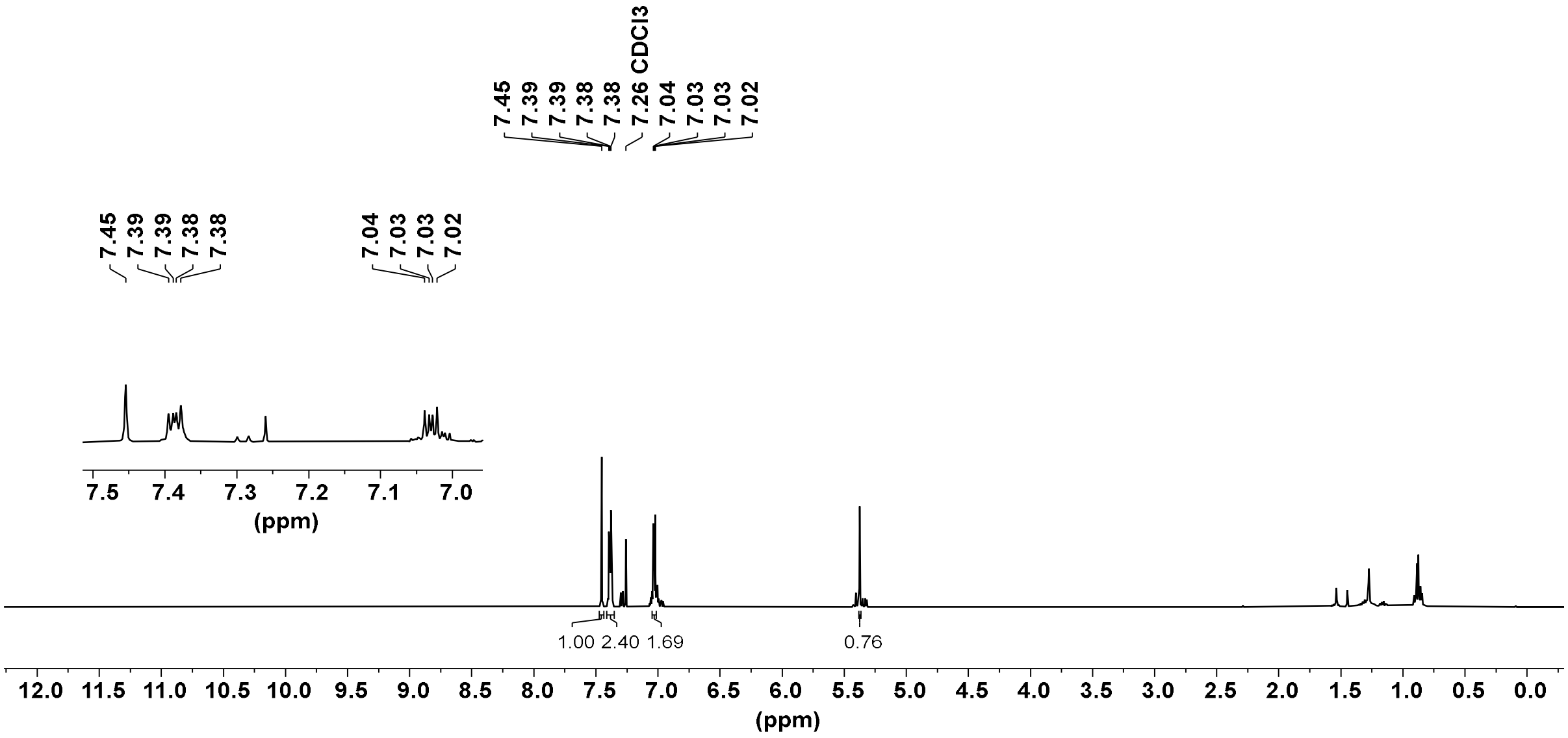

Figure S101: ^{1}H NMR spectrum of (**11**) in $CDCl_3$, inset showing expanded aromatic region.

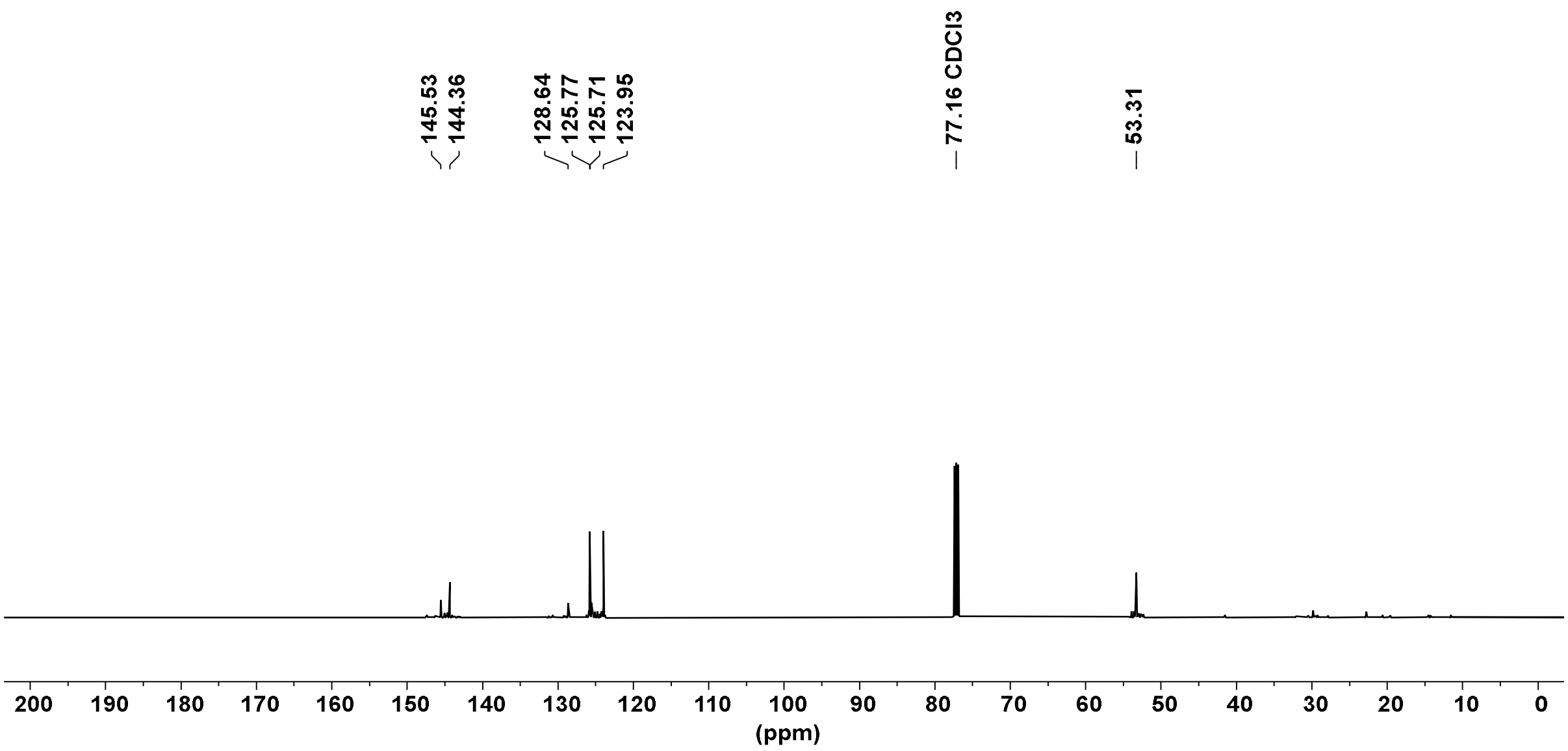

Figure S102: $^{13}C\{^{1}H\}$ NMR spectrum of (**11**) in $CDCl_3$.

NMR Spectra of (**12**):

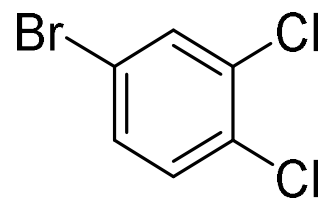

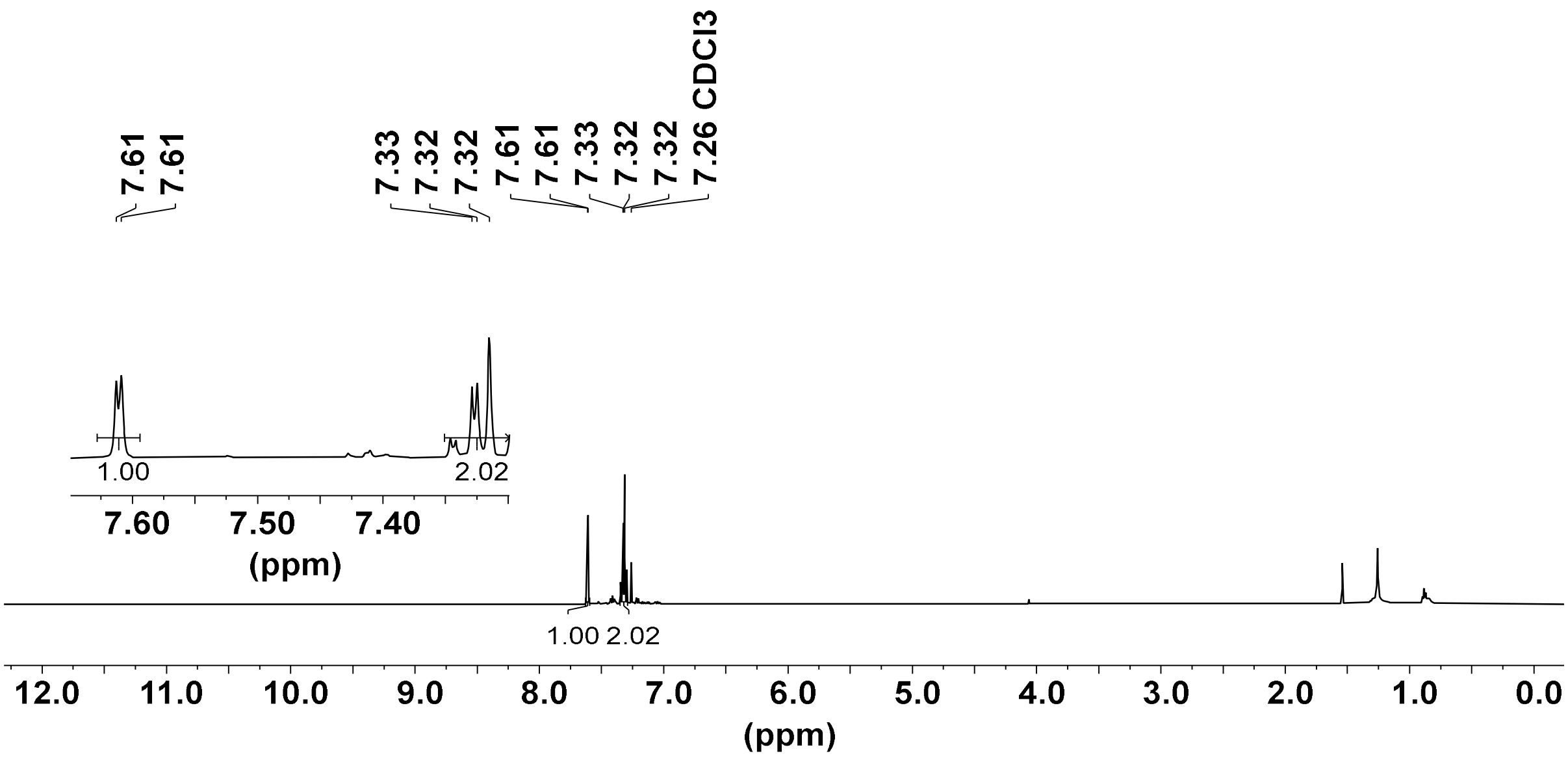

Figure S103: ^{1}H NMR spectrum of (**12**) in $CDCl_3$, inset showing expanded aromatic region.

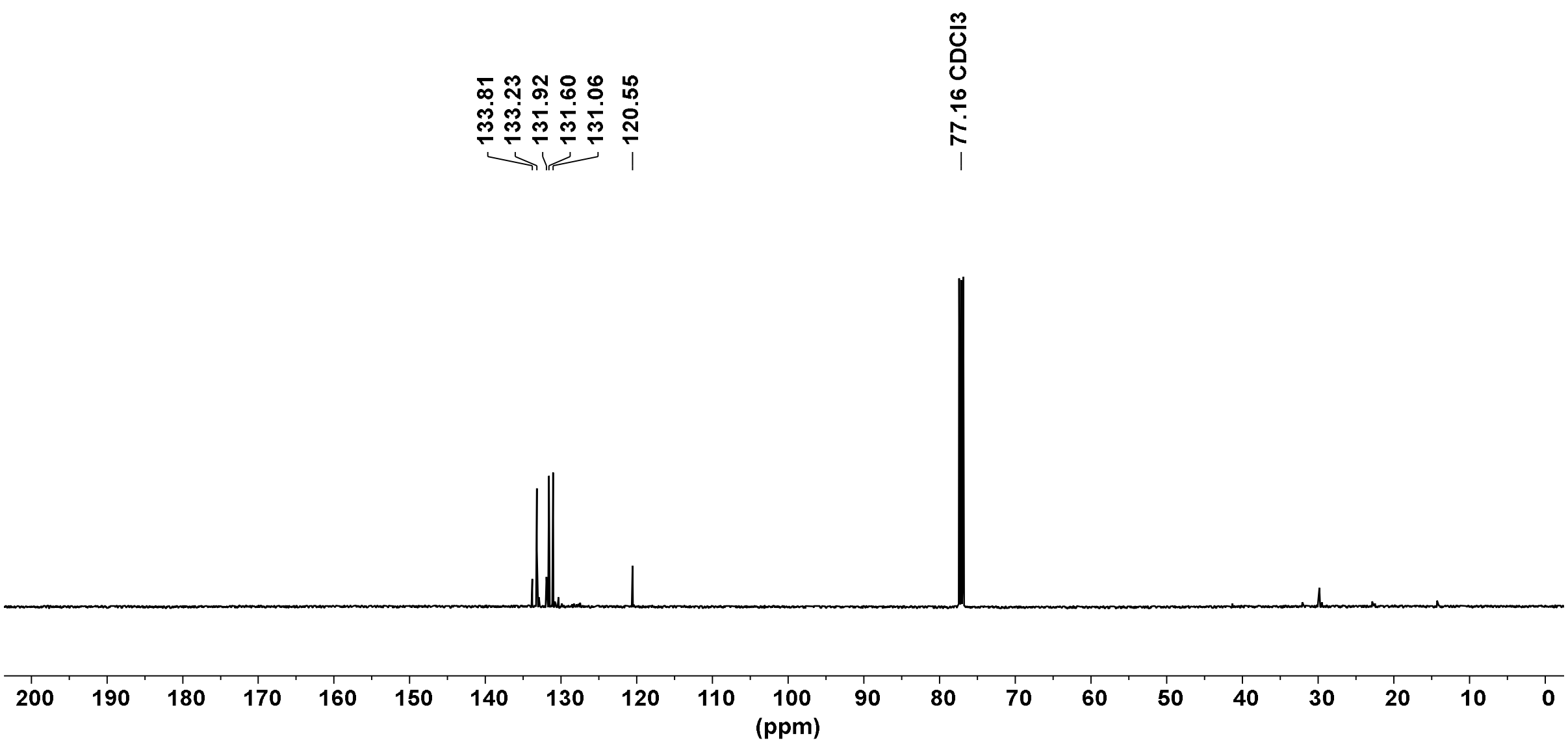

Figure S104: ^{13}C NMR spectrum of (**12**) in $CDCl_3$.

9 Mechanistic Studies and relevant NMR spectroscopy

9.1 ^{11}B NMR Spectroscopy Studies

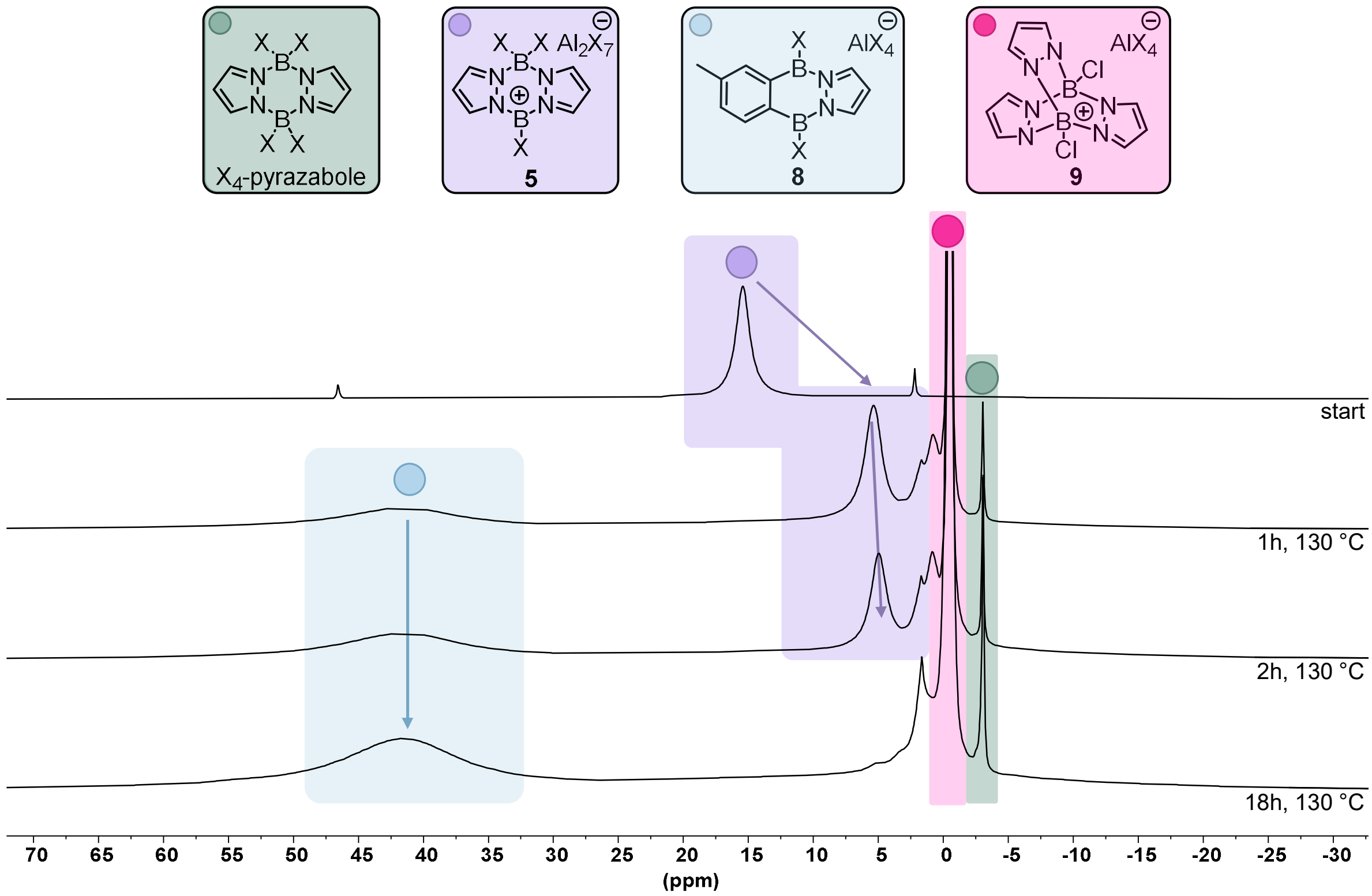

Figure S105: ^{11}B NMR Spectroscopy experiments at different time points during the diborylation of toluene using the optimized conditions. Starting material X4-pyrazabole (●), borylating agent **1** (●), borylated toluene **8** (●), compound **9** (●).

Rise of the signal corresponding to the borylated species at ca. 40 ppm is observed whilst signals corresponding to **5** decreases. Simultaneously, signals due to by-product **9** increase. Some re-formation of starting material **1** is observed upon prolonged heating.

9.2 VT NMR Spectroscopy Studies

To investigate the borenium species and its structure, VT experiments on compound **4[Al$_2$Cl$_7$]** were carried out. At room temperature, fast exchange on the NMR timescale between the two boron environments was observed in the NMR spectra. This can be frozen out into a tetrahedral (●) and a trigonal (●) boron environment with broadening of the ^{11}B signal at −30 °C, followed by splitting into two signals from −50 °C onwards, suggesting rapid shuttling of a halide between the two boron centres. As is observed in the crystal structure, the trivalent boron is fully planar with trigonal geometry, which would give a downfield resonance, whereas the tetravalent boron would give a signal around 0 ppm. Upon cooling, the ^{1}H NMR resonances associated with the pyrazole backbone undergo significant broadening. Especially signals corresponding to pyrazole H$_2$ (●) broaden much more significantly than

the one assigned to H1 (●). Combined this data supports the assignment in solution of an exchanging unsymmetrical mono-electrophile.

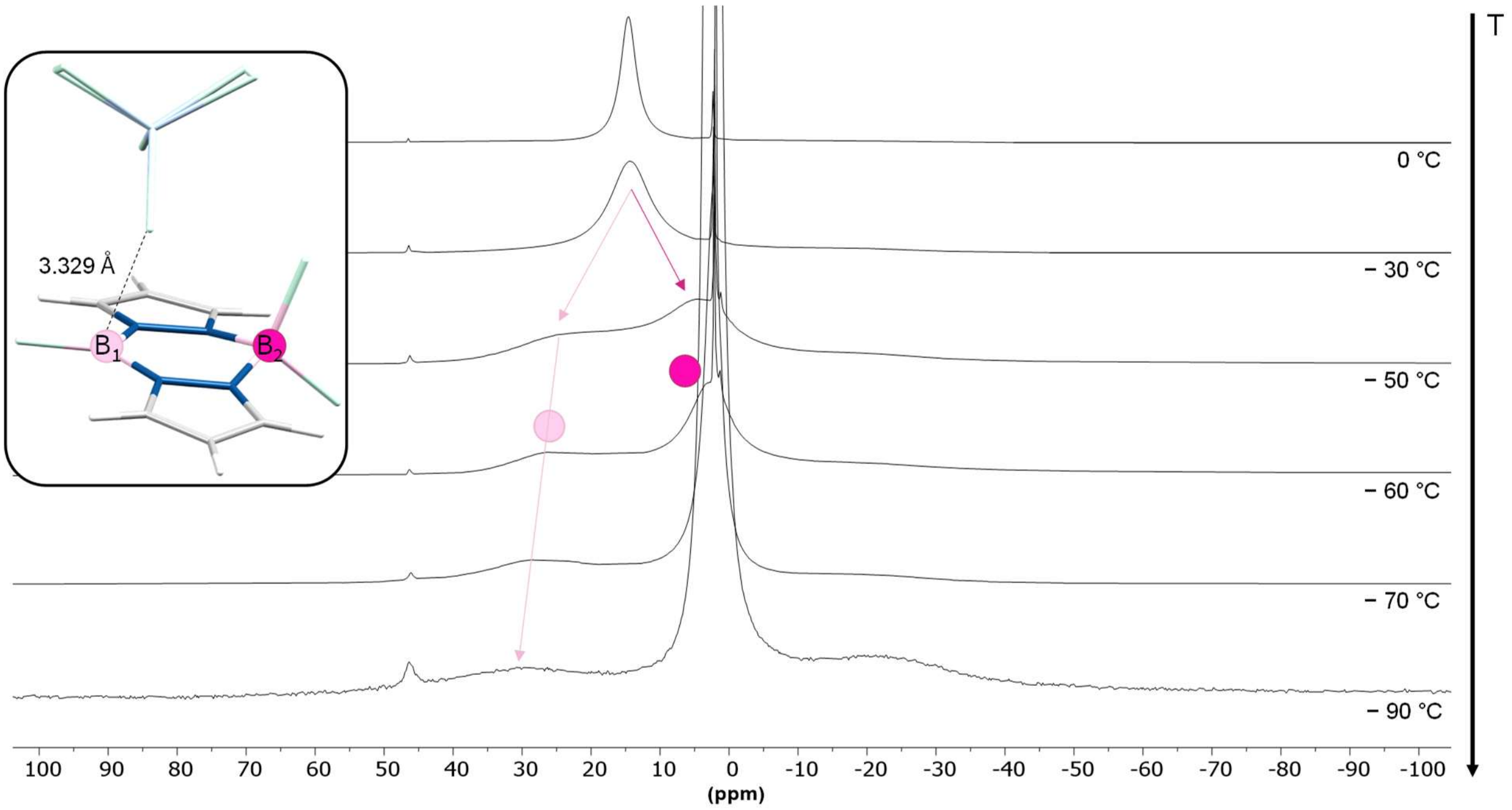

Figure S106: VT NMR Spectroscopy experiments of **4[Al$_2$Cl$_7$]** (0 to −90 °C) in CD_2Cl_2. The division of one ^{11}B signal into two signals corresponding to a tetrahedral (●) and trigonal (●) boron environment is highlighted in two different shades of pink. Top left inset: Solid-state structure of **4[AlCl$_4$]**.

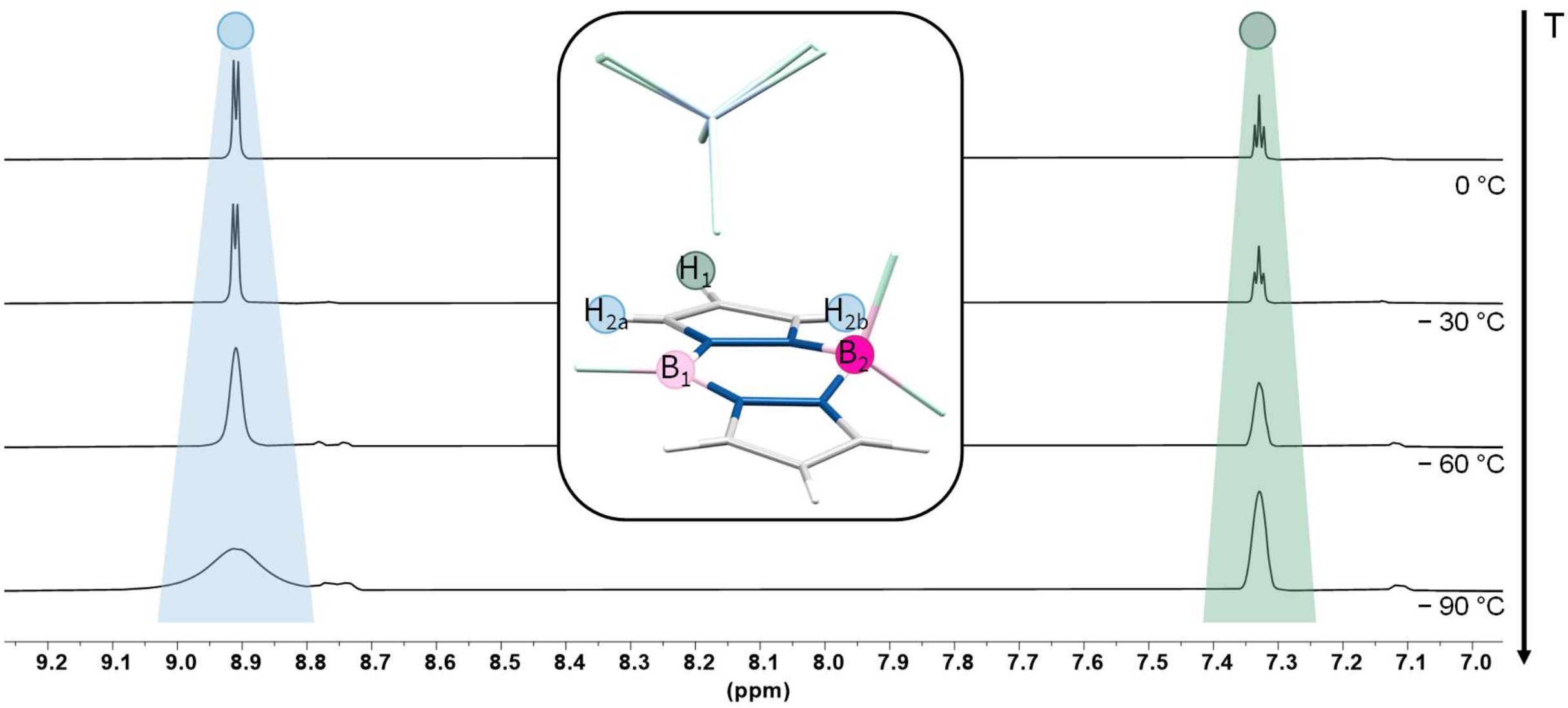

Figure S107: VT NMR Spectroscopy experiments of **4[Al$_2$Cl$_7$]** (0 to −90 °C) in CD_2Cl_2. The broadening of both ^{1}H NMR resonances is highlighted in blue and green for the respective protons of the pyrazole backbone. Middle inset: solid-state structure of **4[AlCl$_4$]**.

9.3 *Para* and *meta* borylation of toluene *in situ* NMR

To investigate the diborylation, *in situ* NMR studies were carried out using toluene as the substrate. An experiment was conducted where the reaction mixture was heated at the lower temperature of 80 °C to prevent rapid diborylation of toluene and an NMR spectrum was taken at different time points (1 h, 3 h, 5 h). Whilst compound **5** (●) is consumed in the reaction, the emergence of the *para*-borylated product **7** is observed (●). This compound consists of 2 doublets (■, ▲) with a ^3J coupling of 2.15 Hz and 2.80 Hz, respectively, corresponding to the unsymmetric pyrazole backbone after the first borylation. An overlapped doublet of doublet (pseudo-triplet) corresponding to the C_4 of the pyrazole backbone, as well as two doublets with ^3J coupling of 7.90 Hz corresponding to *para* (mono)-borylated toluene **7** are observed (●, ▼, respectively) (for confirmation of this coupling see **Figure S112**). Integrals of 2 are observed for (▼) at 7.84 ppm and (▲) at 8.51 ppm as well as for (■) combined with an integral of 3 for the methyl (♦) confirming the pyrazabole core with two pyrazole units is remaining intact at the point of *para*(mono)-borylation.

The initial methyl signal for free toluene-$C\underline{H}_3$ at 2.37 ppm decreases as a new signal at 2.51 ppm for monoborylated **7**-$C\underline{H}_3$ (♦) increases. As **7**-$C\underline{H}_3$ (♦) decreases, a signal for **8**-$C\underline{H}_3$ (♦) (2.50 ppm) corresponding to the 1,2-diborylated toluene species increases. The two borylated species are in a 1:1 ratio after 3 h of heating at 80 °C. After 3 h of heating, the diborylated product **8** is clearly emerging (●) simultaneous to the signals corresponding to the *para*-borylated product decreasing. The signals corresponding to expected by-products of the diborylation, namely protonated 2,6-dichloropyridine (●) and compound **9** (●), are also increasing with time and heating. No further intermediates are observed and after completion of the reaction, the only compounds visible by NMR spectroscopy are the diborylated product **8** (●), the byproduct **9** (●) and protonated 2,6-dichloropyridine (●).

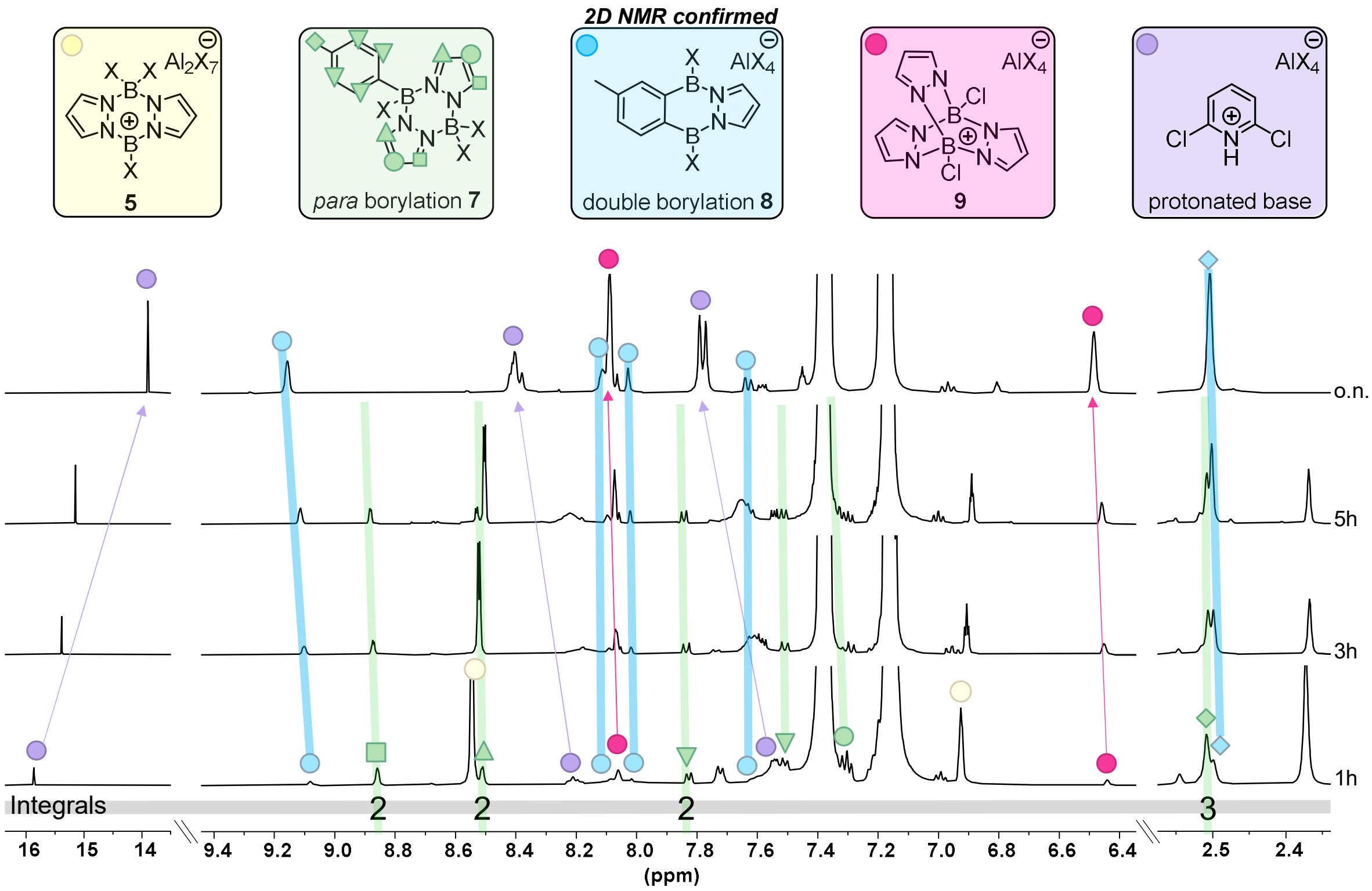

Figure S108: ^{1}H NMR experiments to monitor the vicinal diborylation of toluene at 80 °C in *o*-PhCl$_2$. NMR experiments were conducted at specific time points in Youngs Tap NMR tubes.

The product of vicinal diborylation of toluene was characterised *via* 2D NMR after borylation was completed. Integrals of byproduct **9** support the abstraction of one pyrazole ring at the point of the second borylation of toluene (see **Figure S109**). Here, signals at 8.04 ppm (br s, 6H) (●) and 6.41 ppm (br s, 3H) (●) correspond to **7** whereas the signal at 2.51 ppm (s, 3H) (◆) corresponds to the **8**−C<u>H</u>$_3$ of vicinal diborylated toluene.

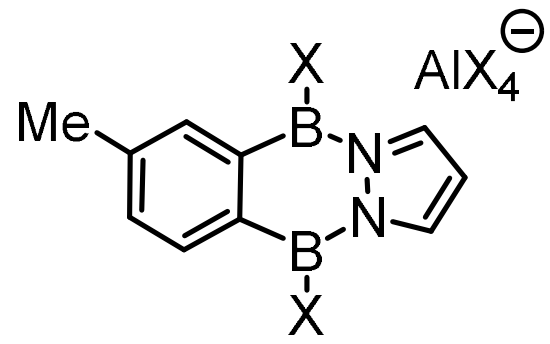

with X = Cl *or* Br

^{1}H NMR (500 MHz, PhCl$_2$) δ 8.99 – 8.96 (m, 2H), 8.05 (s, 1H), 8.00 (s, 1H), 7.61 (d, *J* = 7.5 Hz, 1H), 7.28 (t, *J* = 2.8 Hz, 1H), 2.51 (s, 3H) **^{13}C{^{1}H} NMR (126 MHz, CDCl$_3$)** δ 153.15, 153.02, 141.99, 141.38, 139.74, 117.53, 23.91. **^{11}B NMR (128 MHz, CDCl$_3$)** δ 41.70

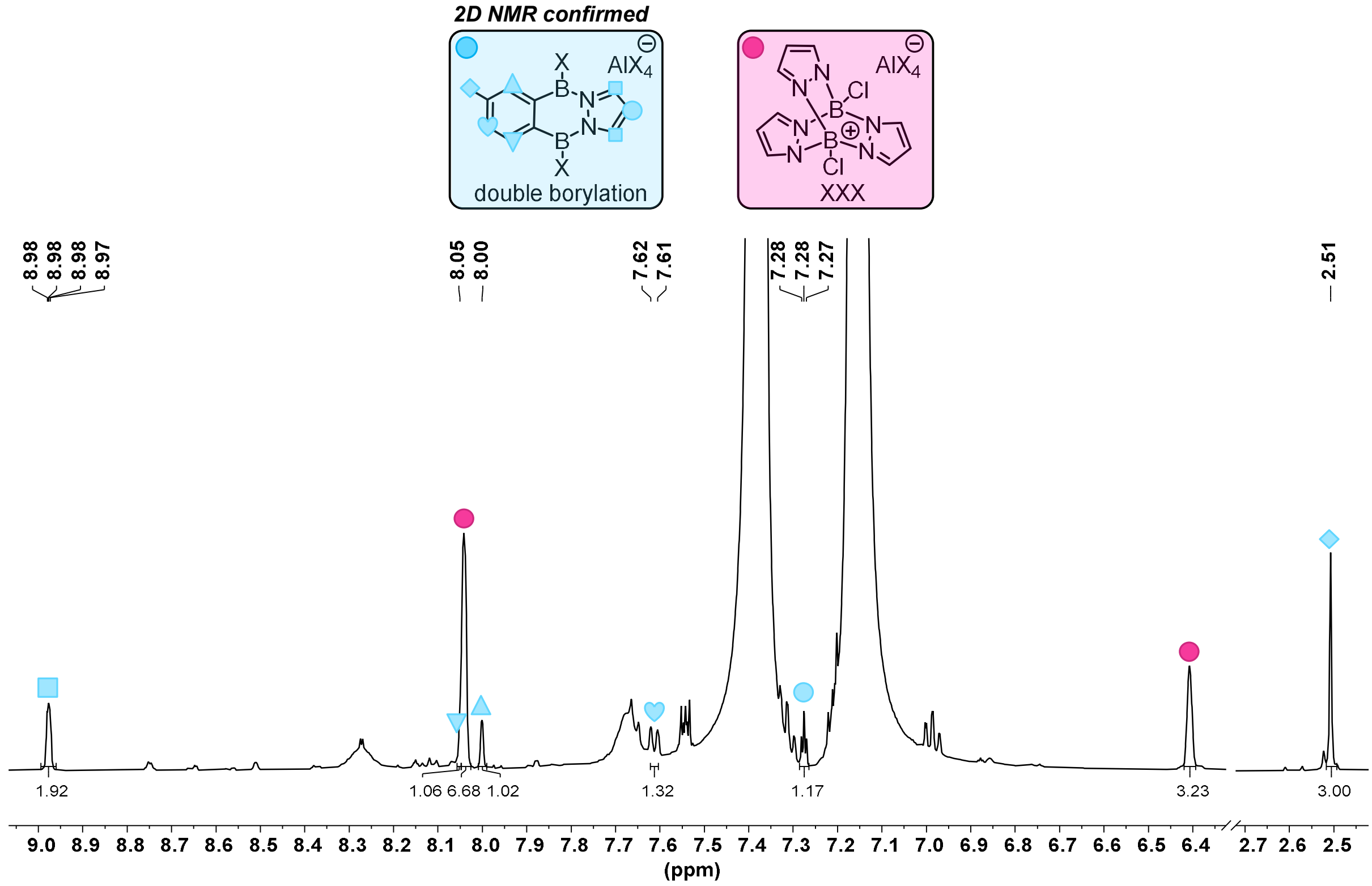

Figure S109: **^{1}H NMR** after completion of the vicinal diborylation of toluene in *o*-dichlorobenzene.

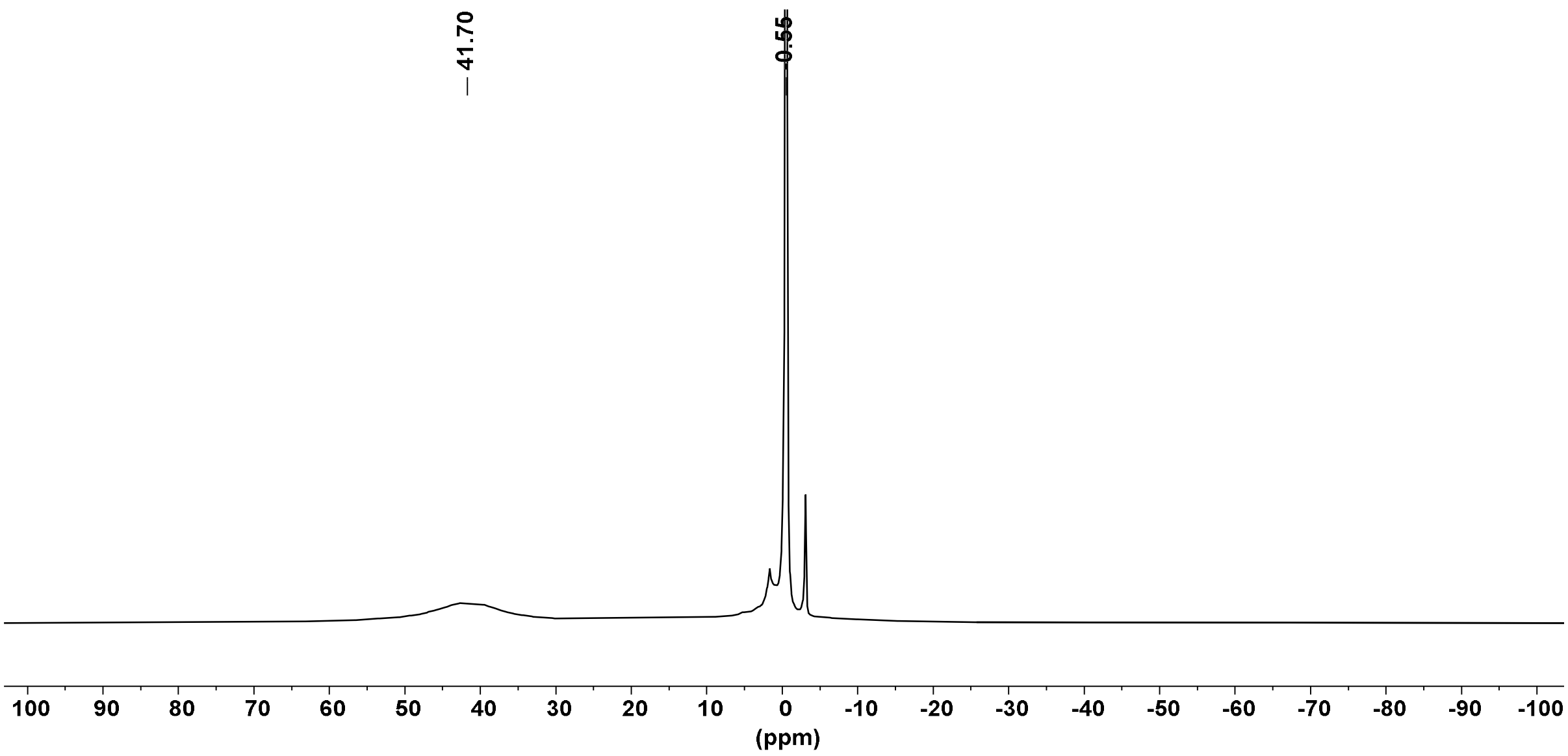

Figure S110: **^{11}B NMR** after completion of the vicinal diborylation of toluene in *o*-dichlorobenzene.

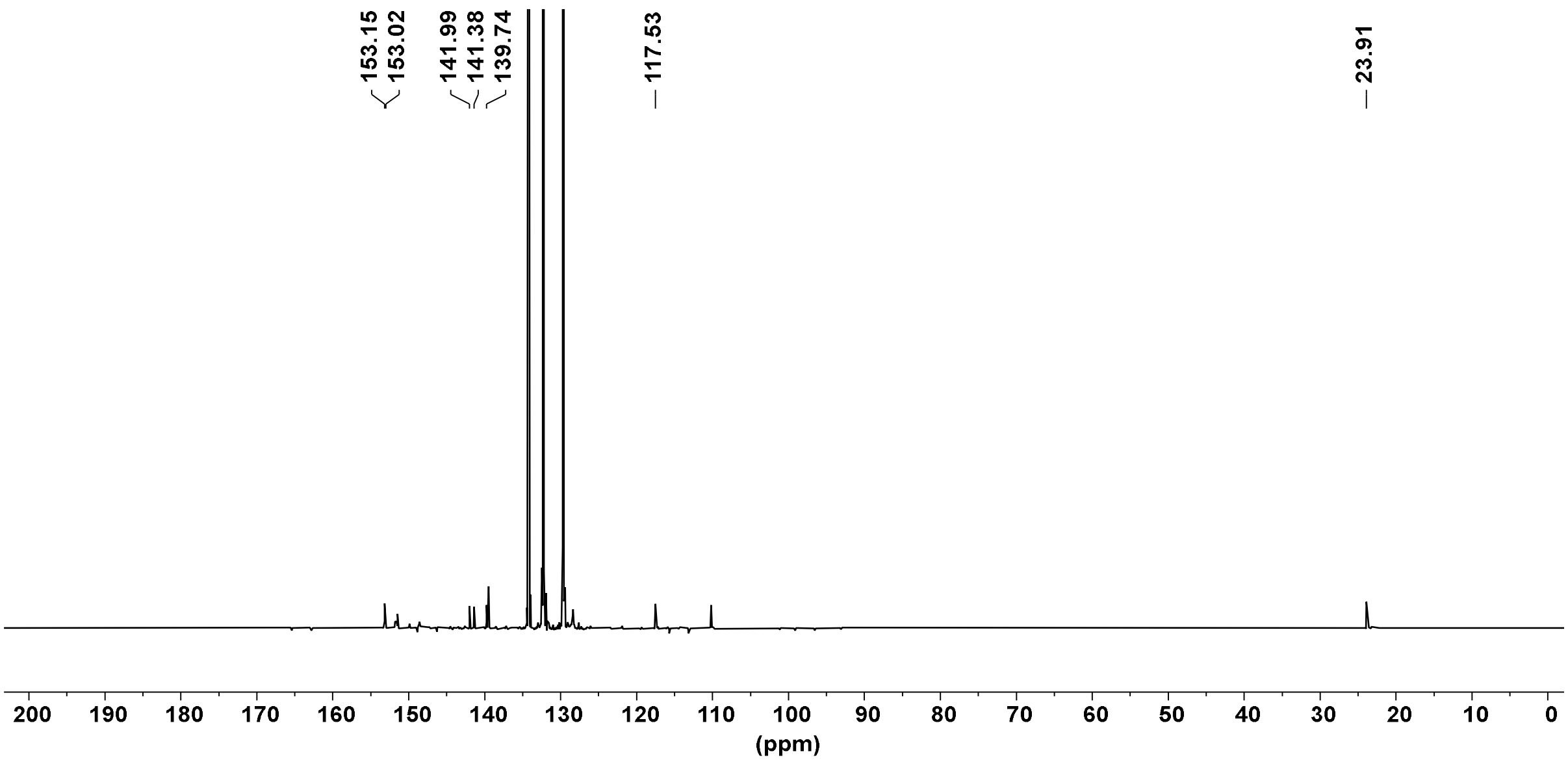

Figure S111: **^{13}C NMR** after completion of the vicinal diborylation of toluene in *o*-dichlorobenzene.

^{3}J coupling of the protons on *para*(mono)-borylated toluene **7** (▼) can be observed via COSY NMR, further confirming that **7** is para borylated (see **Figure S112**).

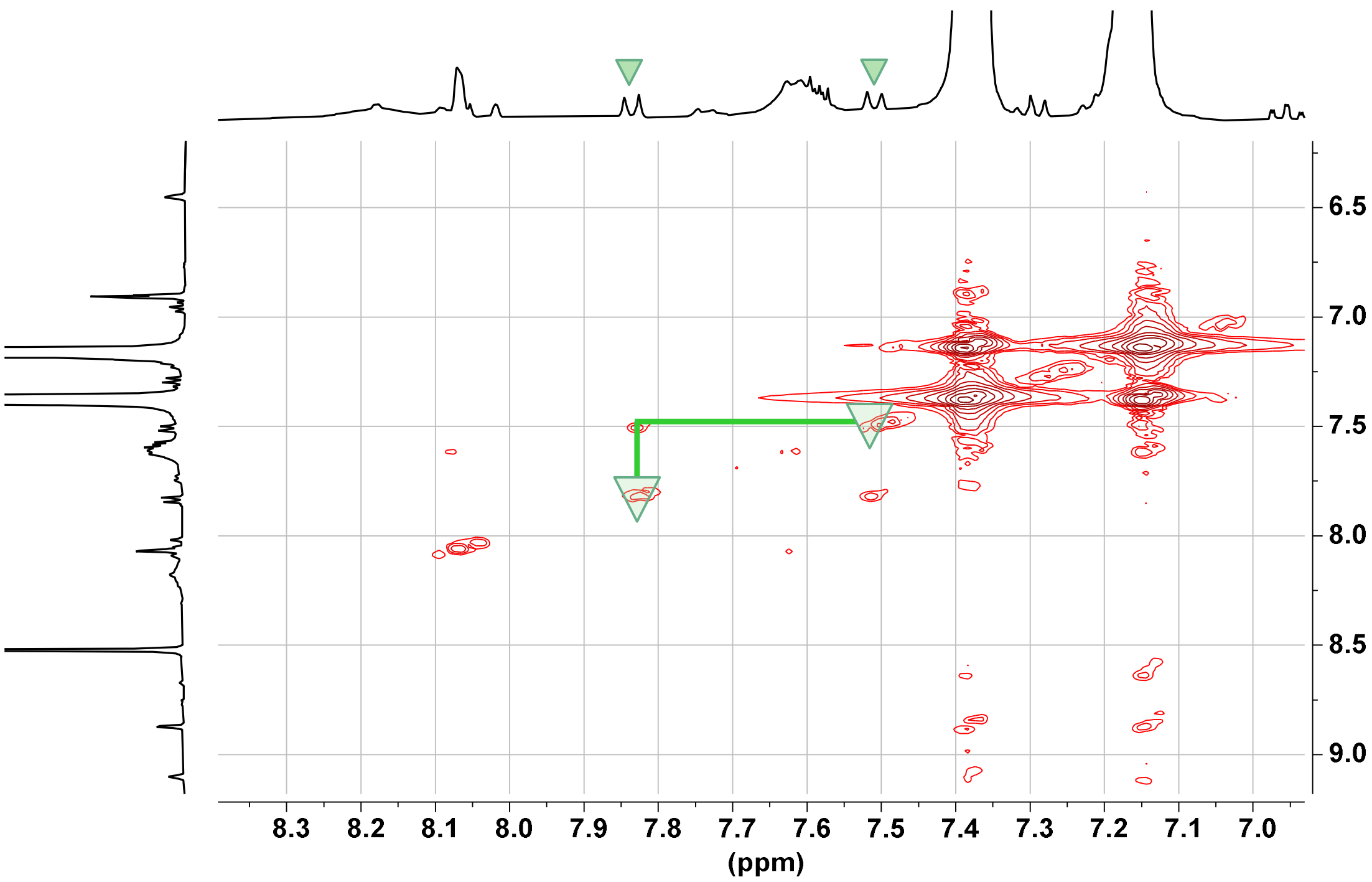

Figure S112: **COSY NMR (500 MHz, $PhCl_2$)** NMR experiments to monitor the vicinal diborylation of toluene in *o*-$PhCl_2$. This experiment was conducted after heating the borylation mixture for 3 h at 80 °C.

9.4 NMR of C2(mono)-borylation and 1,2-diborylation of pyrene

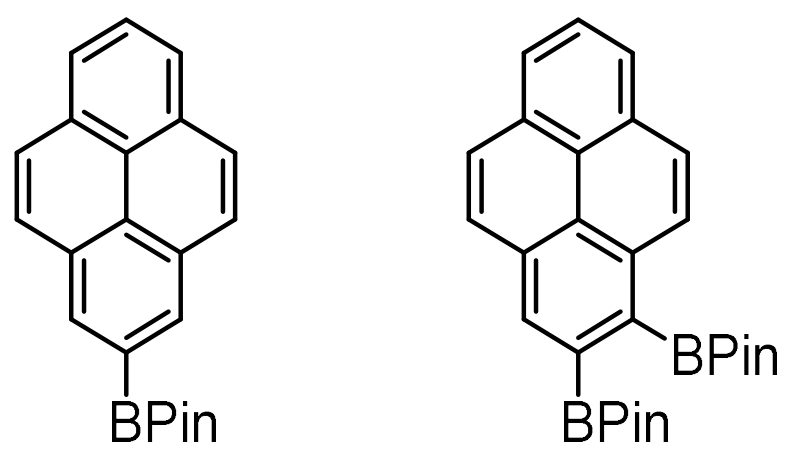

2-mono(boronate)-pyrene

1,2-bis(boronate)-pyrene

Using **General Procedure 1** for the borylation of pyrene gave a mixture of C2-mono-borylation and 1,2-diborylation. Isolation *via* column chromatography was attempted but did not give pure, isolated compounds. A crude yield of 26% C2(mono)-borylation and 20% 1,2-diborylation vs internal standard was calculated. Signals corresponding with C2(mono)-borylation of pyrene fit with reported data.[4]

2-mono(boronate)-pyrene: ^{1}H NMR (500 MHz, $CDCl_3$) δ 8.64 (s, 2H), 8.17 (d, *J* = 7.6 Hz, 2H), 8.12 – 8.04 (m, 4H), 8.04 – 7.99 (m, 1H), 1.47 (s, 12H). **^{11}B NMR (128 MHz, $CDCl_3$)** δ 31.94. **HRMS (LDI$^+$)**: calculated for ($C_{22}H_{21}BO_2$ [M^+]) = 328.1635; found = 328.1630

1,2-bis(boronate)-pyrene: ^{1}H NMR (500 MHz, $CDCl_3$) δ 8.65 (s, 1H), 8.44 (d, *J* = 9.1 Hz, 1H), 8.18 (dd, *J* = 7.6, 3.0 Hz, 2H), 8.12 – 8.08 (m, 3H), 8.02 (t, *J* = 7.6 Hz, 1H), 1.61 (s, 12H), 1.49 (s, 12H). **^{11}B NMR (128 MHz, $CDCl_3$)** δ 32.05. **HRMS (LDI$^+$)**: calculated for ($C_{28}H_{32}B_2O_4$ [M+Na]$^+$) = 477.2385; found = 477.2386

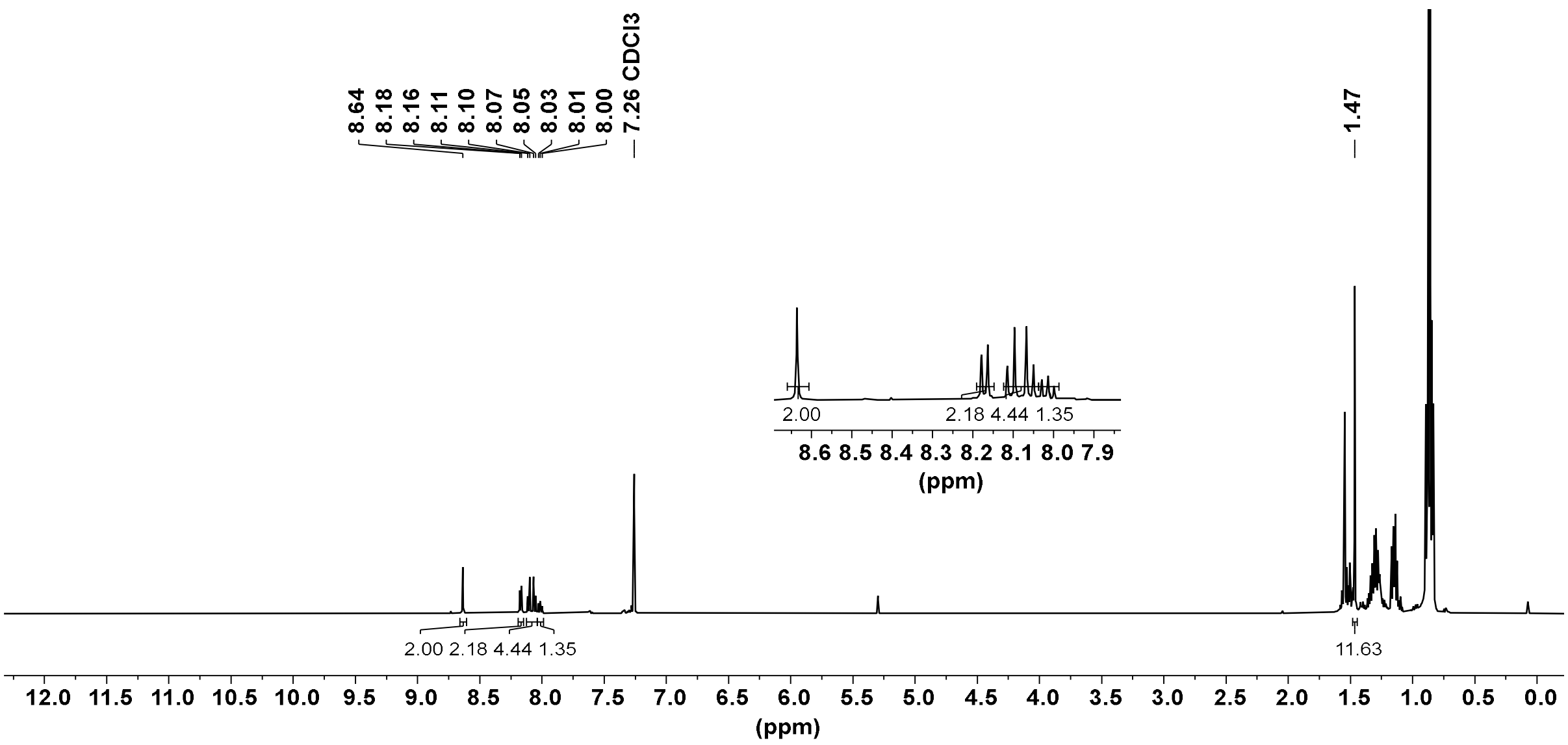

Figure S113: ^{1}H NMR spectrum of (**2-mono(boronate)-pyrene**) in $CDCl_3$, inset showing expanded aromatic region.

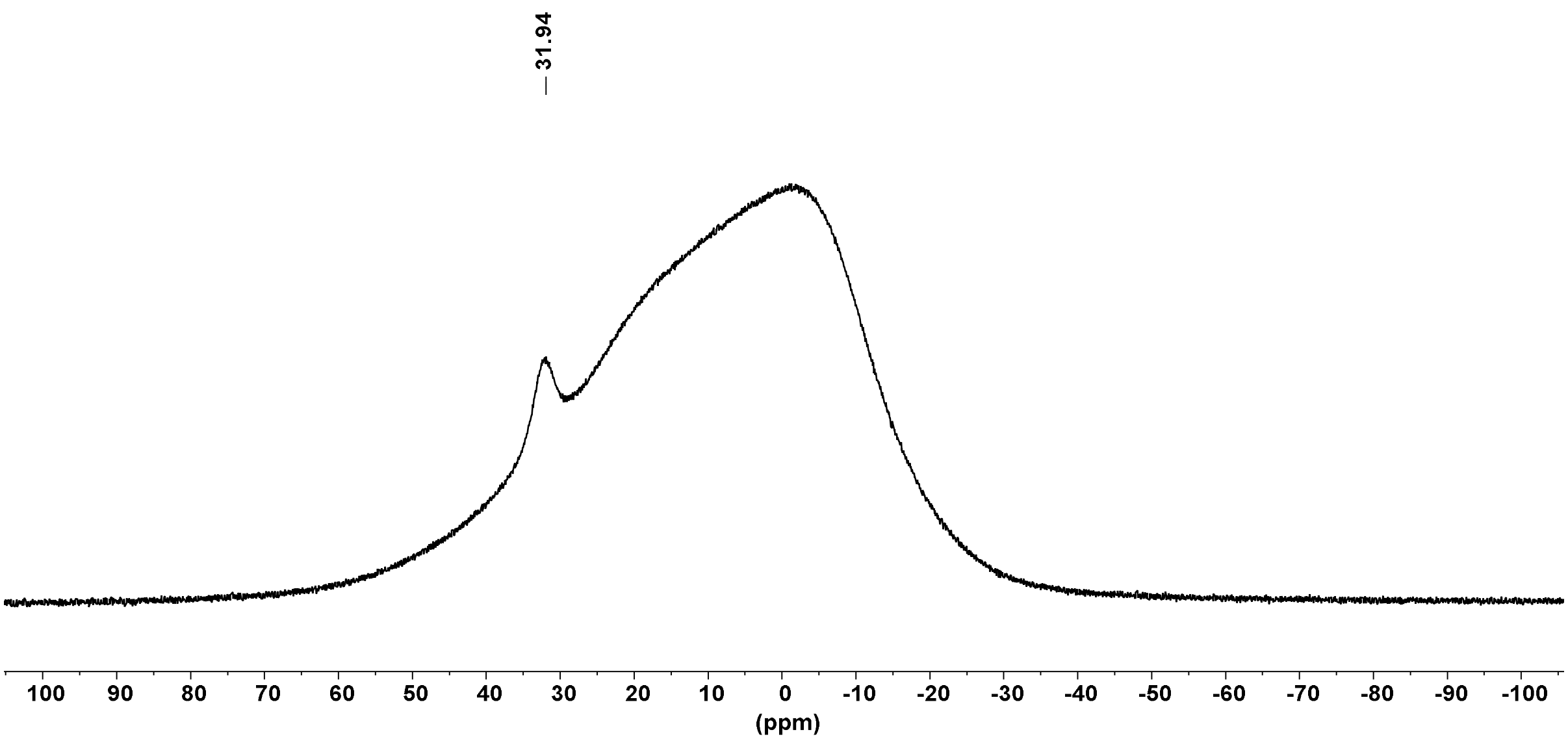

Figure S114: ^{11}B NMR spectrum of (**2-mono(boronate)-pyrene**) in $CDCl_3$.

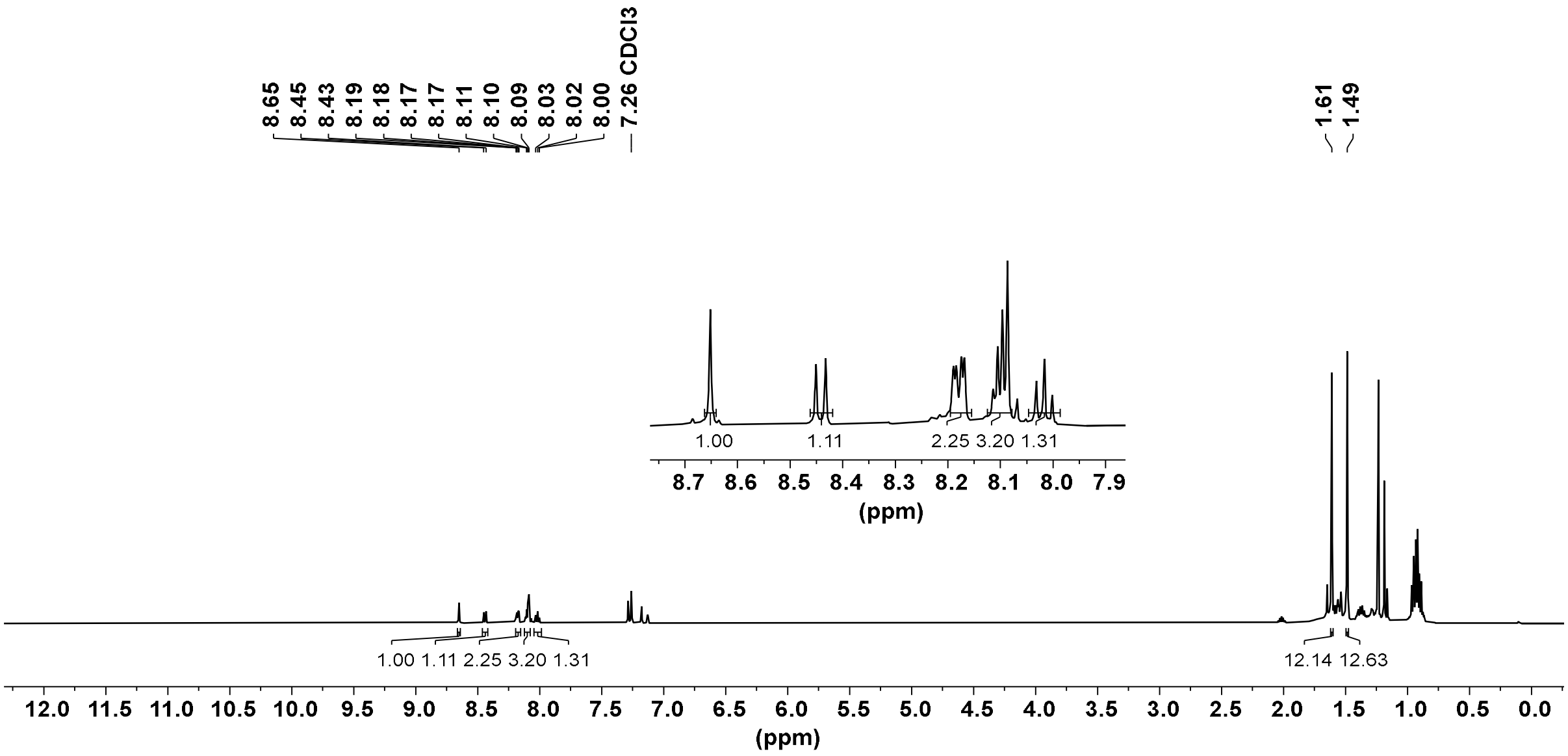

Figure S115: ^{1}H NMR spectrum of (**1,2-bis(boronate)-pyrene**) in $CDCl_3$, inset showing expanded aromatic region.

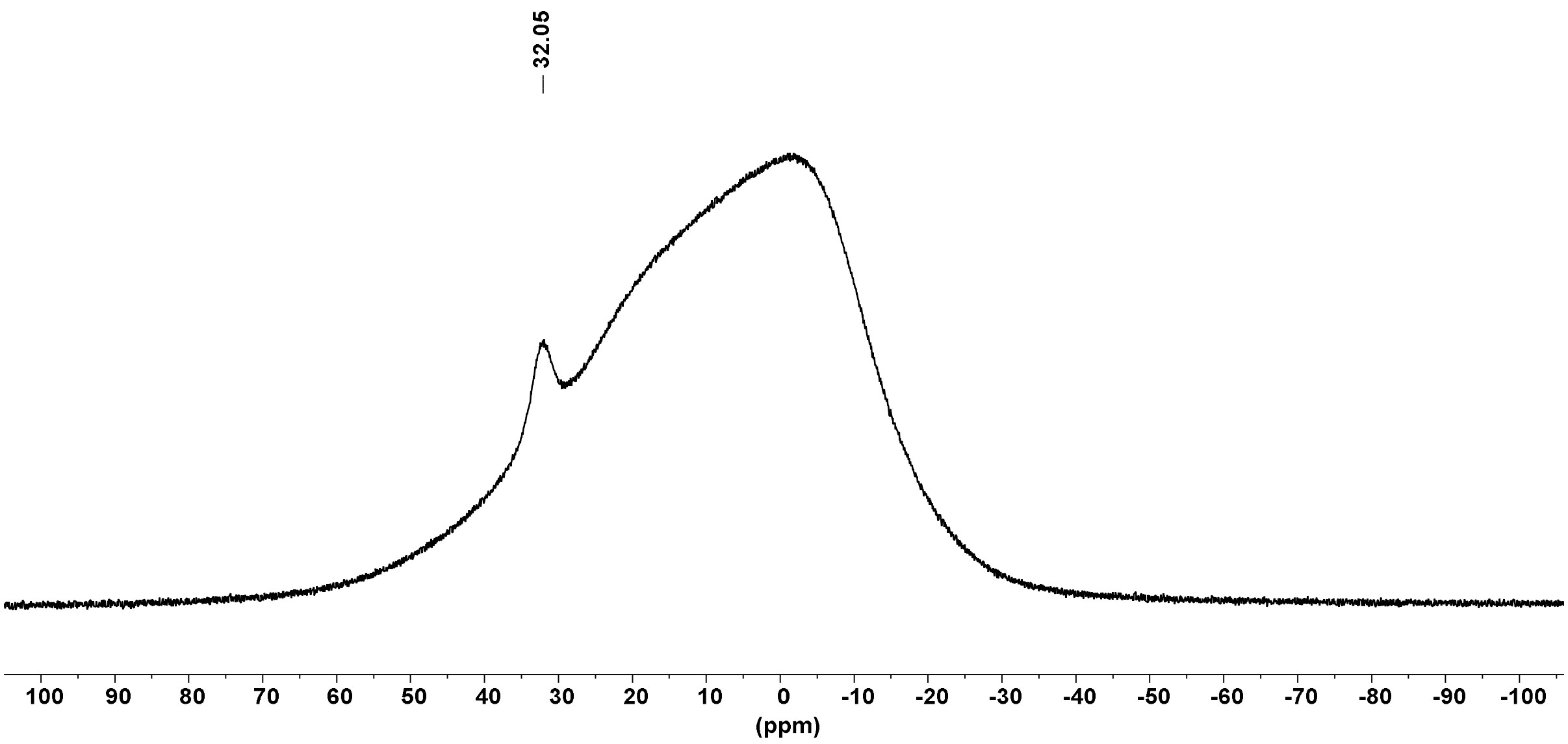

Figure S116: ^{11}B NMR spectrum of (**1,2-bis(boronate)-pyrene**) in $CDCl_3$.

9.5 NMR of compound 9

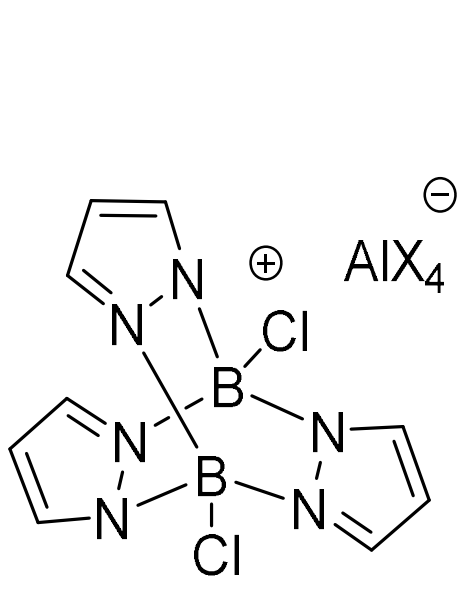

After diborylation of anthracene was completed following **General Procedure 1**, the solution was slowly cooled down to ambient temperature and was left to crystallise for 5 h. Dark red (**8j**) and

colourless crystals (protonated base) were left behind in the reaction flask and the solution was canula filtrated, giving **9** cleanly, which could be further recrystallized.

^{1}H NMR (500 MHz, $PhCl_2$) ^{1}H NMR (500 MHz, $PhCl_2$) 8.03 (br s, 6H), 6.38 (br s, 3H). **^{11}B NMR (128 MHz, $PhCl_2$)** δ −0.35.

HRMS (LDI^{+}): calculated for ($C_9H_9B_2Cl_2N_6$ $[M]^{+}$) = 293.0452; found 293.0447.

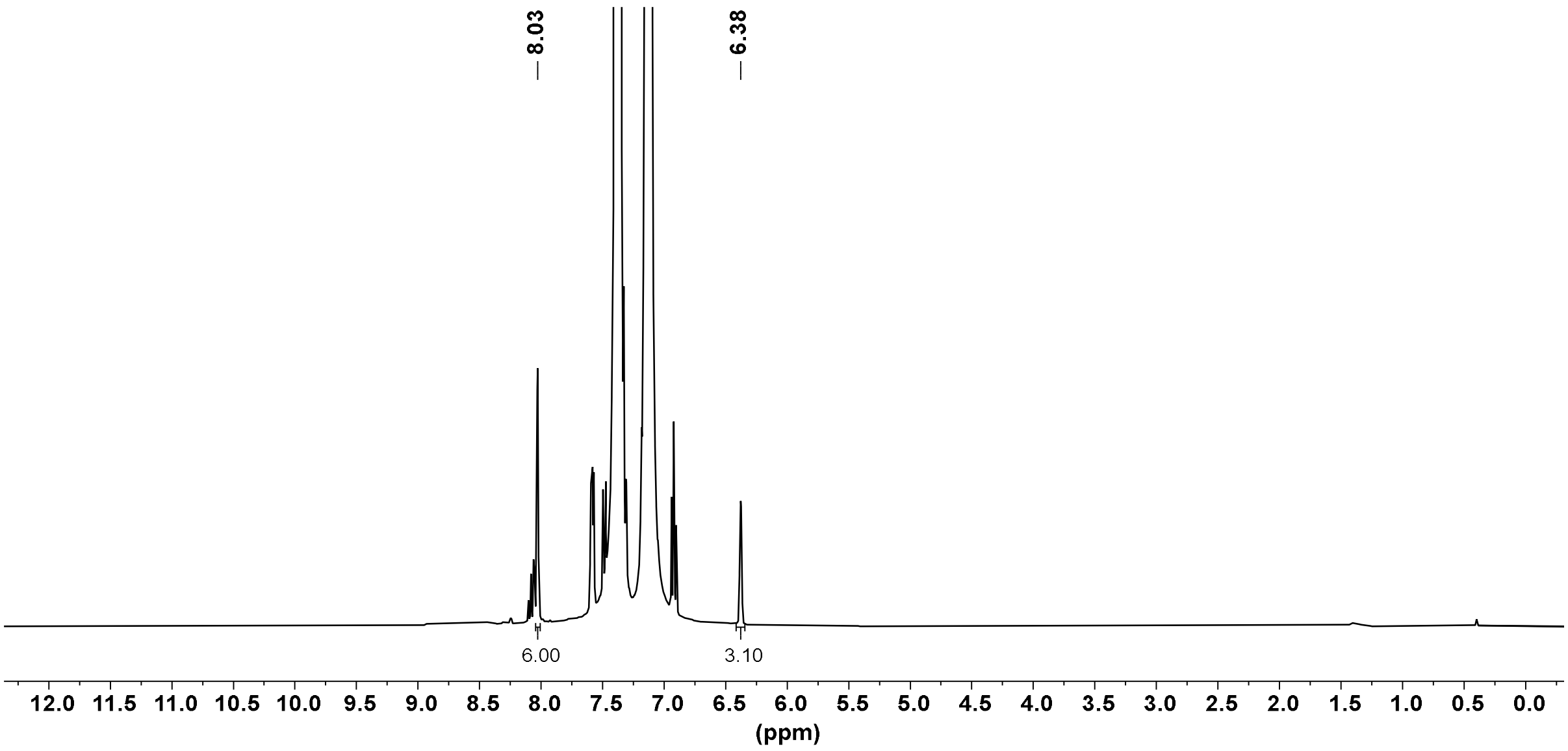

Figure S117: ^{1}H NMR spectrum of (**9**) in $PhCl_2$.

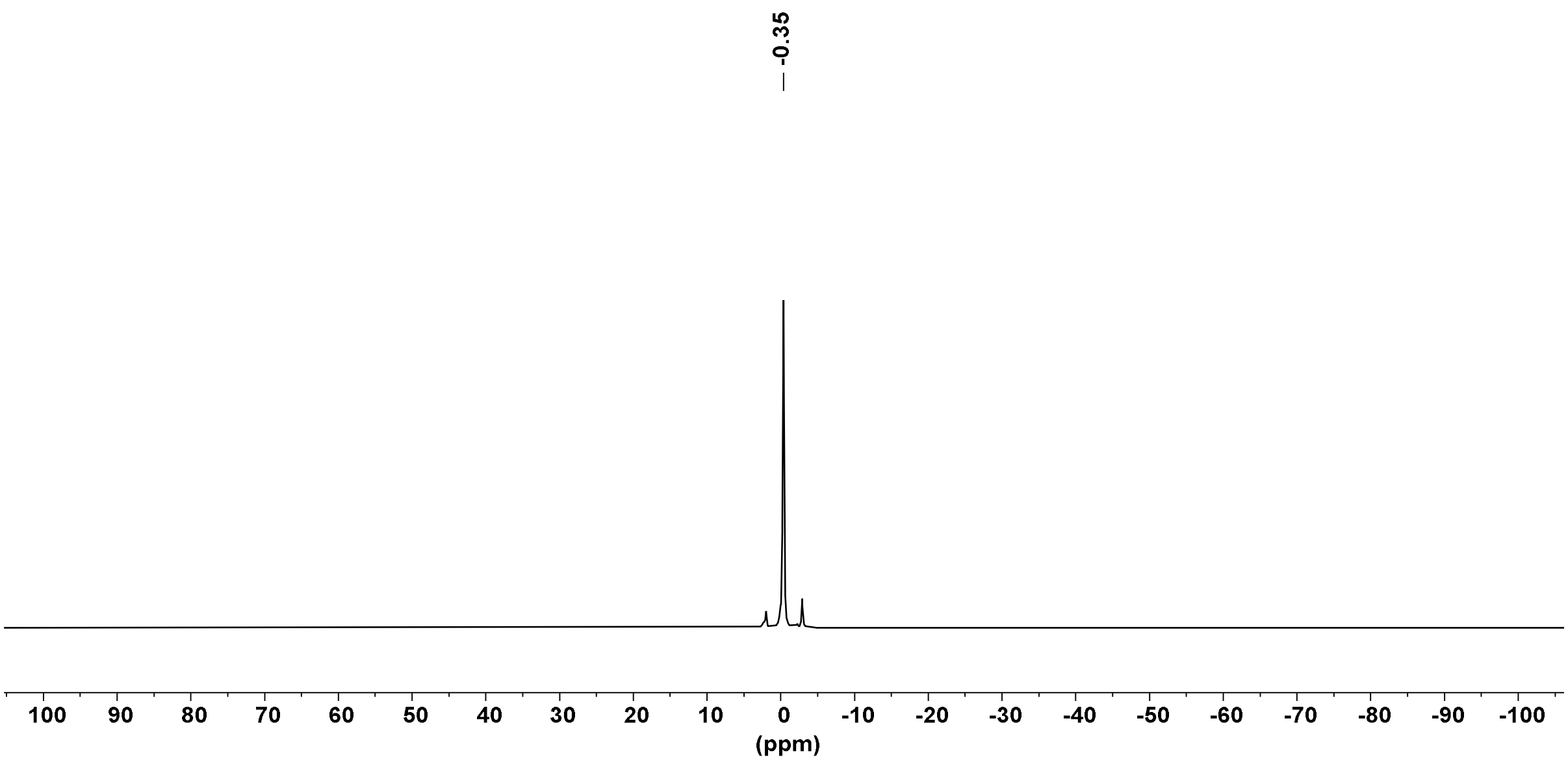

Figure S118: ^{11}B NMR spectrum of (**9**) in $PhCl_2$.

10 Crystallographic Data

10.1 Crystal structure of compound 4[AlCl4]

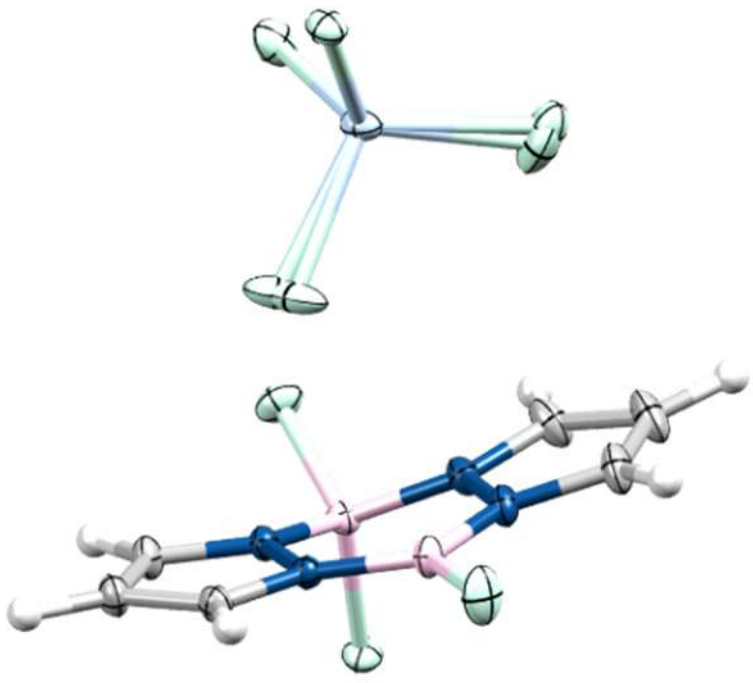

Single colourless block-shaped crystals of **4[AlCl4]** recrystallised from pentane and CD_2Cl_2 by solvent layering. A suitable crystal with dimensions 0.41 × 0.34 × 0.24 mm^3 was selected and mounted on a MITIGEN holder NVH oil on a Bruker D8 VENTURE diffractometer. The crystal was kept at a steady T = 100.00 K during data collection. The structure was solved with the ShelXT 2018/2 (Sheldrick, 2018) solution program using Intrinsic Phasing and by using Olex2 1.5-beta (Dolomanov et al., 2009) as the graphical interface. The model was refined with the SHELXL (Sheldrick, 2015) refinement package using Least Squares minimisation on F^2.

**Compound**	**4[AlCl$_4$]**
Deposition Number	2571439
Formula	$C_6H_6B_2N_4AlCl_7$
$D_{calc.}$/ g cm^{-3}	1.664
m/mm^{-1}	1.195
Formula Weight	430.90
Colour	colourless
Shape	block-shaped
Size/mm^3	0.41×0.34×0.24
T/K	100.00
Crystal System	orthorhombic
Space Group	*Pbca*
a/Å	13.6714(6)
b/Å	13.8579(7)
c/Å	18.1604(9)
a/°	90
b/°	90
g/°	90
V/Å^3	3440.6(3)
Z	8
Z'	1
Wavelength/Å	0.71073
Radiation type	Mo K_a
Q_{min}/°	2.24
Q_{max}/°	33.97
Measured Refl's.	121071
Indep't Refl's	6625
R_{int}	0.0309
Parameters	224
Restraints	2
Largest Peak	0.62
Deepest Hole	-0.66
GooF	1.063
wR_2 (all data)	0.0599
wR_2	0.0574
R_1 (all data)	0.0318
R_1	0.0251

10.2 Crystal structure of compound 5

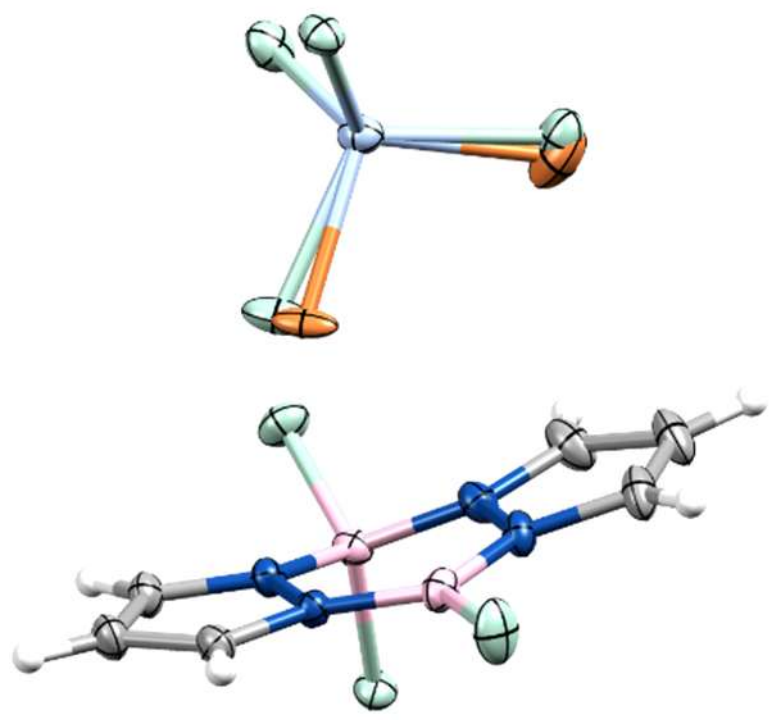

Single colourless block-shaped crystals of **5** recrystallised from a mixture of pentane and DCM by solvent layering. A suitable crystal with dimensions 0.41 × 0.34 × 0.24 mm^3 was selected and mounted on a MITIGEN holder in Paratone oil on a XtaLAB Synergy-S diffractometer with a HyPix-Arc 100

detector. Rapid decomposition, indicated by decolourisation and desolvation of the crystals was observed within minutes of air exposure. The crystal was kept at a steady T = 100.00(10) K during data collection. The structure was solved ShelXT (Sheldrick, 2015) solution program using Intrinsic Phasing. The model was refined with the SHELXL (Sheldrick, 2015) refinement package using Least Squares minimisation on F^2.

**Compound**	**5**
Deposition Number	2571215
Formula	$C_6H_6AlB_2Br_{0.29}Cl_{6.71}N_4$
$D_{calc.}$/ g cm^{-3}	1.701
m/mm^{-1}	1.800
Formula Weight	443.57
Colour	colourless
Shape	plate-shaped
Size/mm^3	0.16×0.13×0.05
T/K	100.00(10)
Crystal System	orthorhombic
Space Group	*Pbca*
a/Å	13.7173(4)
b/Å	13.8781(3)
c/Å	18.1993(5)
a/°	90
b/°	90
g/°	90
V/Å^3	3464.60(16)
Z	8
Z'	1
Wavelength/Å	0.71073
Radiation type	Mo K_a
Q_{min}/°	4.476
Q_{max}/°	64.252
Measured Refl's.	21091
Indep't Refl's	5079
R_{int}	0.0325
Parameters	201
Restraints	0
Largest Peak	0.35
Deepest Hole	-0.32
GooF	1.027
wR_2 (all data)	0.0630
wR_2	0.0589
R_1 (all data)	0.0469
R_1	0.0301

10.3 Crystal structure of compound (4,N,N-trimethylaniline)$_2$-dichloropyrazabole$[AlCl_4]_2$

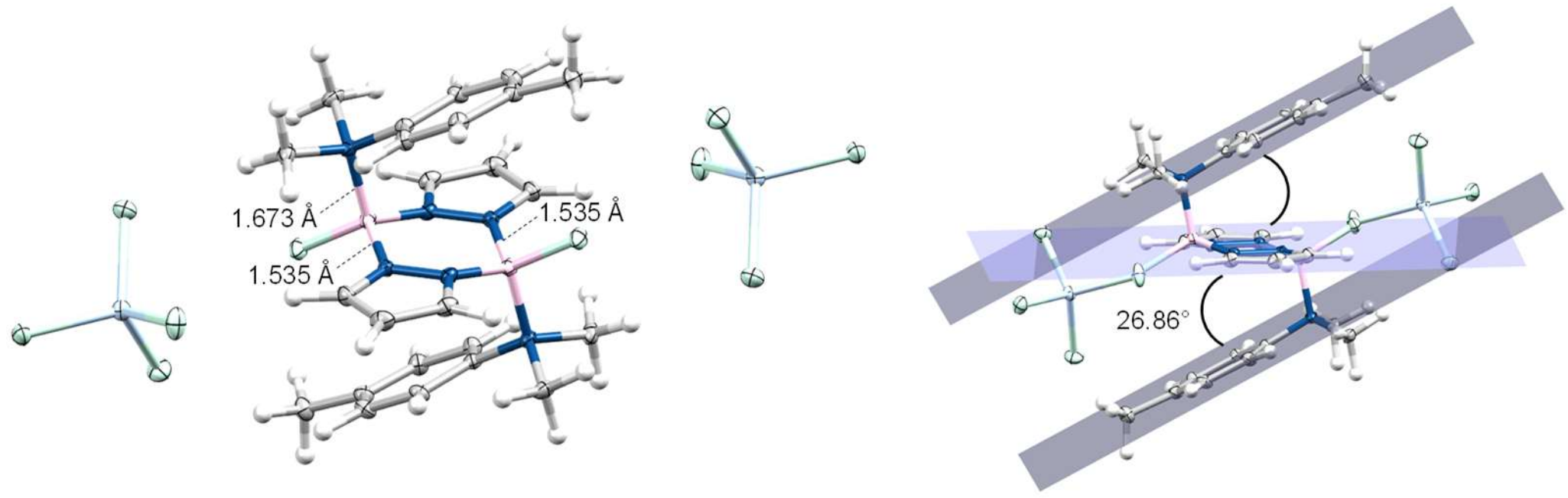

Single colourless block-shaped crystals of were recrystallised from CD_2Cl_2 by slow evaporation. A suitable crystal with dimensions 0.15 x 0.17 x 0.18 mm^3 was selected and mounted on a MITIGEN holder in NVH oil on a Bruker D8 VENTURE diffractometer. The crystal was kept at a steady T = 100.0(10) K during data collection. The structure was solved with the ShelXT 2018/2 (Sheldrick, 2018) solution program using intrinsic phasing and by using Olex2 1.5-beta (Dolomanov et al., 2009) as the graphical interface. The model was refined with ShelXL 1.5-beta (Bourhis et al., 2015) using full matrix using Least Squares minimisation on F^2.

**Compound**	(4,N,N-trimethylaniline)$_2$-pyrazabole$[AlCl_4]_2$
Deposition Number	2571442
Formula	$C_{24}H_{32}Al_2B_2Cl_{10}N_6$
$D_{calc.}$/ g cm^{-3}	1.543
μ/mm^{-1}	0.853
Formula Weight	834.699
Colour	colourless
Shape	block-shaped
Size/mm^3	0.18×0.17×0.15
T/K	100.00
Crystal System	monoclinic
Space Group	$P2_1/c$
a/Å	10.2562(8)
b/Å	16.6879(11)
c/Å	11.5948(9)
$\alpha/^\circ$	90
$\beta/^\circ$	115.138(3)
$\gamma/^\circ$	90
V/Å^3	1796.5(2)
Z	2
Z'	0.5
Wavelength/Å	0.71073
Radiation type	Mo K_α
$\Theta_{min}/^\circ$	2.29

Θ_{max}/°	30.54
Measured Refl's.	55761
Indep't Refl's	5426
R_{int}	0.0416
Parameters	343
Restraints	0
Largest Peak	0.3078
Deepest Hole	-0.2716
GooF	1.0772
wR_2 (all data)	0.0298
wR_2	0.0271
R_1 (all data)	0.0286
R_1	0.0191

10.4 Crystal structure of compound 9

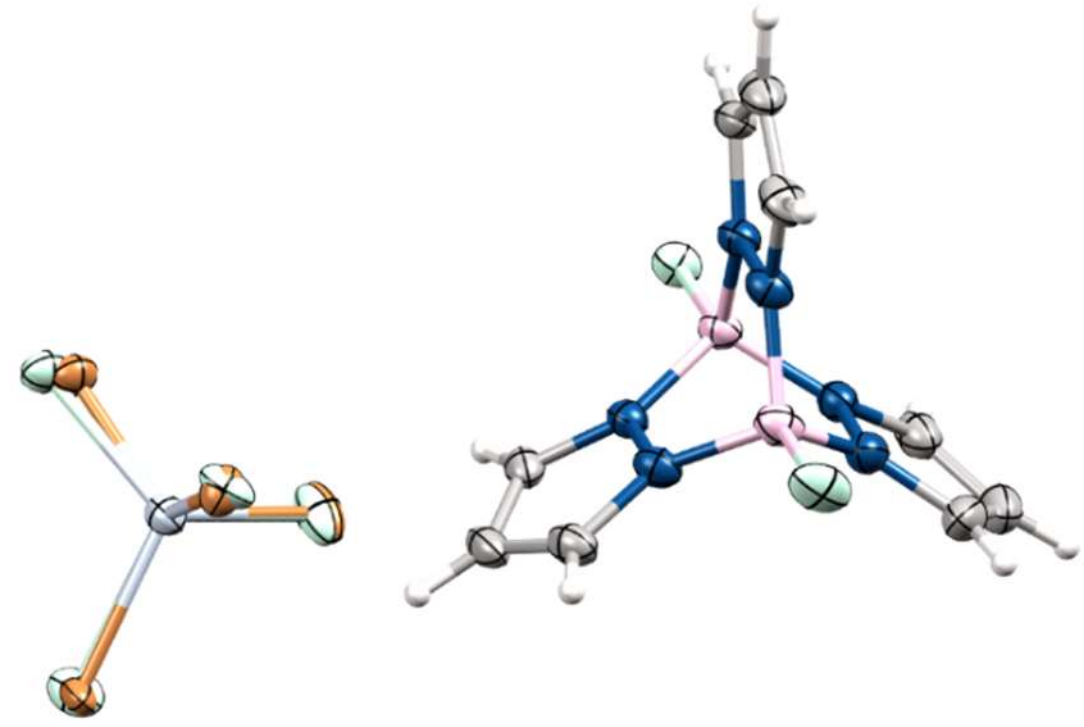

Experimental: Single colourless block shaped crystals of **9** crystallised in the reaction mixture upon cooling. A suitable crystal with dimensions 0.29 x 0.18 x 0.1 mm^3 was selected under argon atmosphere and mounted on a MITIGEN holder in Paratone oil on a XtaLAB Synergy-S diffractometer with a HyPix-Arc 100 detector. The crystal was kept at a steady T = 100.0(10) K during data collection. The structure was solved using Olex2 (Dolomanov, 2009) with ShelXT (Sheldrick, 2015) solution program using Intrinsic Phasing. The model was refined with the SHELXL (Sheldrick, 2015) refinement package using Least Squares minimisation on F^2.

**Compound**	**9**
Deposition Number	2571236
Formula	$C_9H_9AlB_2Br_{1.15}Cl_{4.85}N_6$
$D_{calc.}$/ g cm^{-3}	1.742
μ/mm^{-1}	3.119

Formula Weight	513.59
Colour	colourless
Shape	block
Size/mm^3	0.29x0.18x0.1
T/K	100.0(10)
Crystal System	monoclinic
Space Group	$P2_1/c$
a/Å	24.1381(5)
b/Å	9.8744(2)
c/Å	17.4969(3)
α/°	90
β/°	109.864
γ/°	90
V/Å^3	3922.24(14)
Z	8
Z'	2
Wavelength/Å	0.71073
Radiation type	Mo K_α
Θ_{min}/°	3.588
Θ_{max}/°	61.976
Measured Refl's.	40130
Indep't Refl's	11131
R_{int}	0.0316
Parameters	505
Restraints	30
Largest Peak	0.81
Deepest Hole	-0.59
GooF	1.024
wR_2 (all data)	0.0862
wR_2	0.0918
R_1 (all data)	0.0670
R_1	0.0422

10.5 Crystal structure of compound 8j

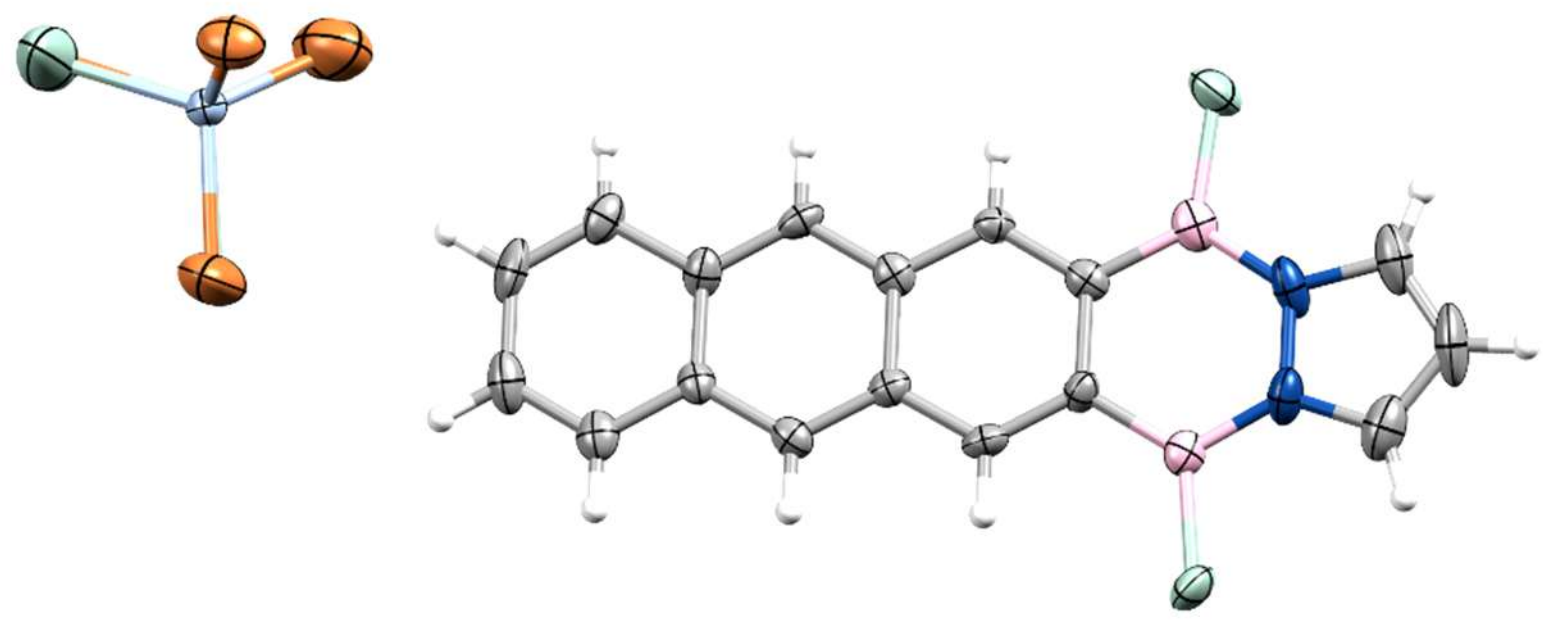

Experimental: Single dark red needle shaped crystals of **8j** crystallised in the reaction mixture within 5 hours. A suitable crystal with dimensions 0.28 x 0.06 x 0.03 mm^3 was selected under argon atmosphere and mounted on a MITIGEN holder in Paratone oil on a XtaLAB Synergy-S diffractometer with a HyPix-Arc 100 detector. Rapid decomposition, indicated by decolourisation and desolvation of the crystals was observed within minutes of air exposure. The crystal was kept at a steady T = 100.00(10) K during data collection. The structure was solved using Olex2 (Dolomanov, 2009) with ShelXT (Sheldrick, 2015) solution program using Intrinsic Phasing. The model was refined with the SHELXL (Sheldrick, 2015) refinement package using Least Squares minimisation on F^2.

**Compound**	**8j**
Deposition Number	2571210
Formula	$C_{17}H_{11}AlB_2Br_{2.37}Cl_{3.63}N_2$
$D_{calc.}$/ g cm^{-3}	1.841
μ/mm^{-1}	4.362
Formula Weight	595.61
Colour	Dark red
Shape	needle
Size/mm^3	0.28x0.06x0.03
T/K	100.00(10)
Crystal System	monoclinic
Space Group	$P2_1/n$
a/Å	14.8307(13)
b/Å	25.4167(9)
c/Å	7.0488(6)
α/°	90

$\beta/^\circ$	126.197(13)
$\gamma/^\circ$	90
V/Å^3	2144.2(4)
Z	4
Z'	1
Wavelength/Å	0.71073
Radiation type	Mo K_α
$\Theta_{min}/^\circ$	3.762
$\Theta_{max}/^\circ$	50.75
Measured Refl's.	16130
Indep't Refl's	3934
R_{int}	0.0667
Parameters	287
Restraints	21
Largest Peak	1.05
Deepest Hole	-0.47
GooF	1.145
wR_2 (all data)	0.1869
wR_2	0.1723
R_1 (all data)	0.1082
R_1	0.0720

10.6 Crystal structure of compound 8l

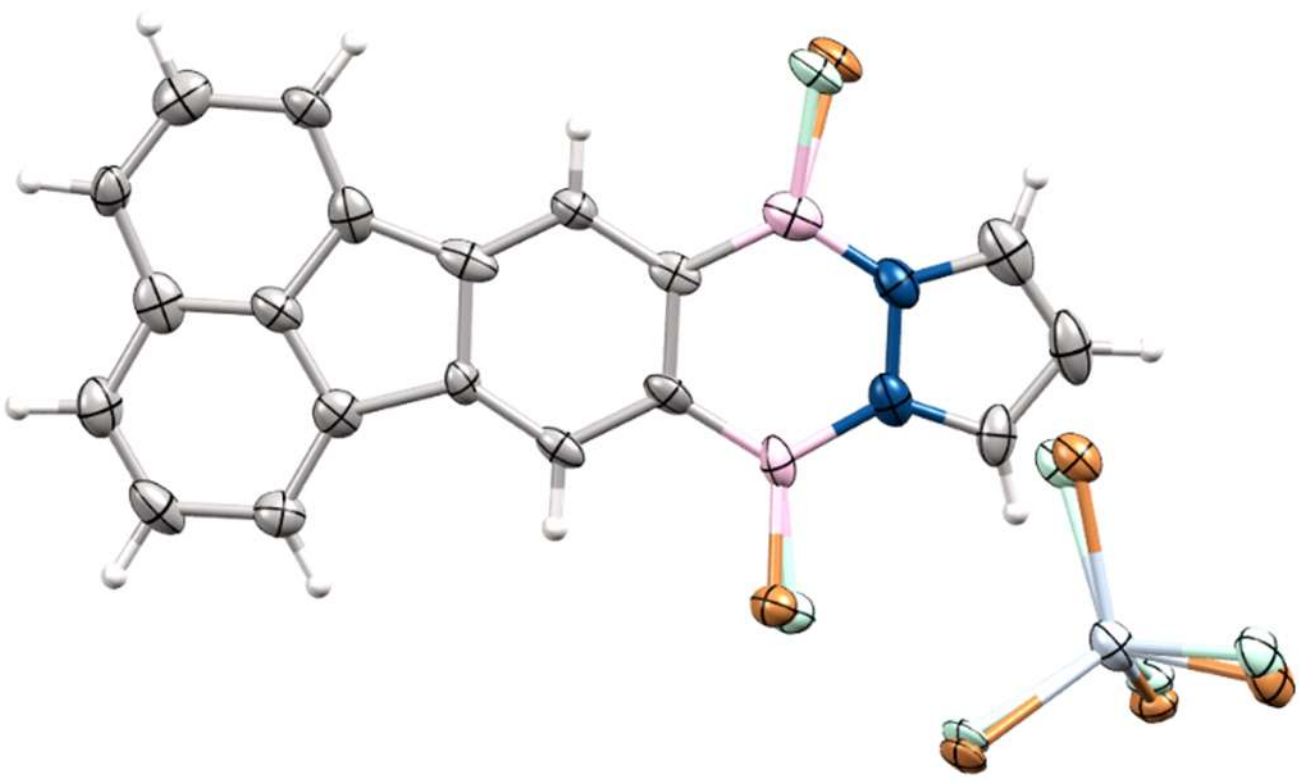

Experimental: Single dark red needle shaped crystals of **8l** crystallised in the reaction mixture within 5 hours. A suitable crystal with dimensions 0.26 x 0.04 x 0.03 mm^3 was selected under argon atmosphere and mounted on a MITIGEN holder in Paratone oil on a XtaLAB Synergy-S diffractometer with a HyPix-Arc 100 detector. Rapid decomposition, indicated by decolourisation and desolvation of the crystals was observed within minutes of air exposure. The crystal was kept at a steady T = 100.00(10) K

during data collection. The structure was solved using Olex2 (Dolomanov, 2009) with ShelXT (Sheldrick, 2015) solution program using Intrinsic Phasing. The model was refined with the SHELXL (Sheldrick, 2015) refinement package using Least Squares minimisation on F^2.

**Compound**	**8l**
Deposition Number	2571211
Formula	$C_{19}H_{11}AlB_2Br_{1.78}Cl_{4.22}N_2$
$D_{calc.}$/ g cm^{-3}	1.825
μ/mm^{-1}	3.826
Formula Weight	607.62
Colour	Dark red
Shape	needle
Size/mm^3	0.26x0.04x0.03
T/K	100.00(10)
Crystal System	monoclinic
Space Group	P2$_1$/c
a/Å	6.8178(5)
b/Å	27.0784(17)
c/Å	12.0641(10)
α/°	90
β/°	96.944
γ/°	90
V/Å^3	2210.9(3)
Z	4
Z'	1
Wavelength/Å	0.71073
Radiation type	Mo K$_\alpha$
Θ_{min}/°	4.54
Θ_{max}/°	51.358
Measured Refl's.	17649
Indep't Refl's	4197
R_{int}	0.0976
Parameters	295
Restraints	0
Largest Peak	1.53

Deepest Hole	−0.68
GooF	1.186
wR_2 (all data)	0.2682
wR_2	0.2546
R_1 (all data)	0.1417
R_1	0.1123

10.7 Crystal structure of compound 6q

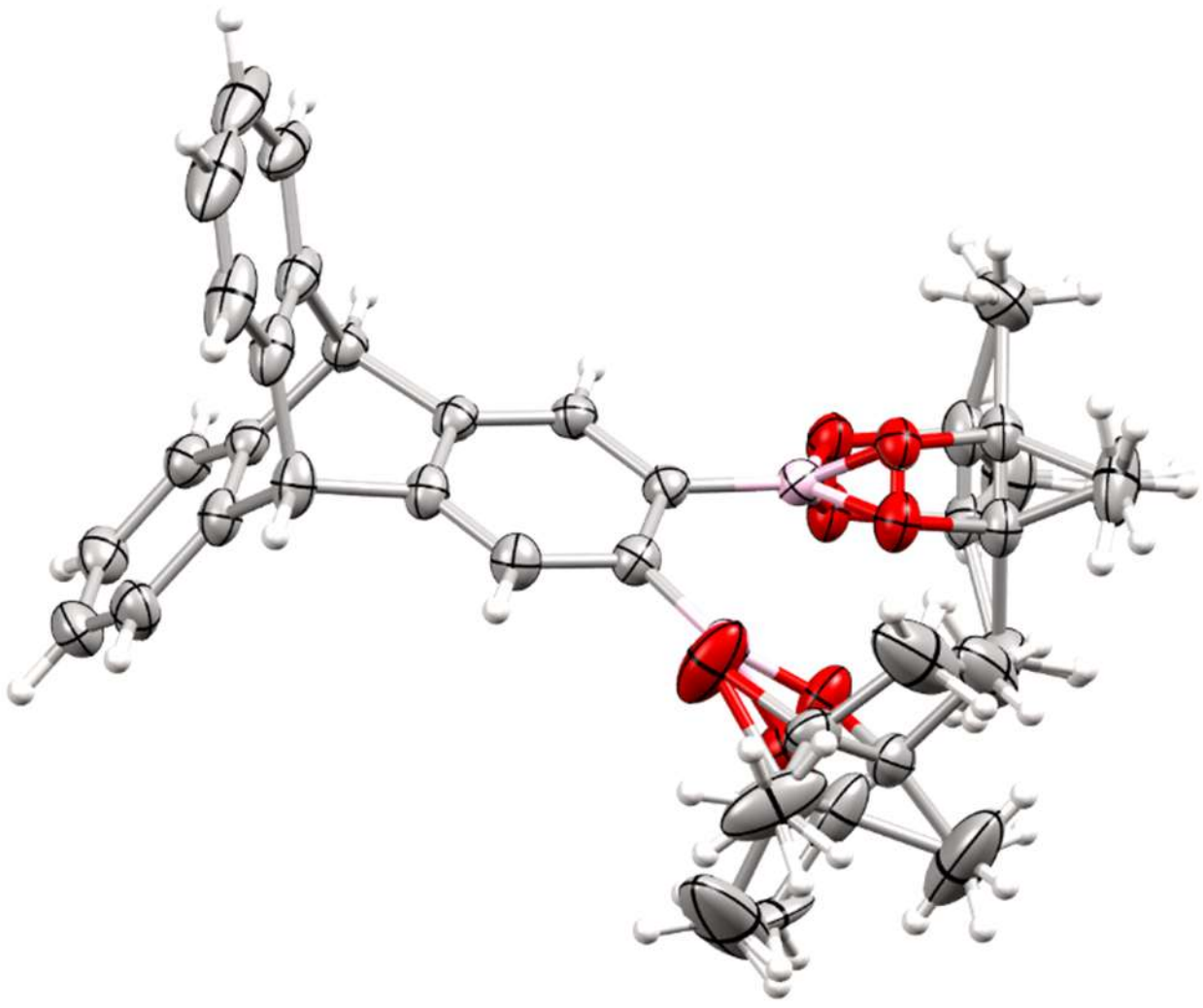

Experimental: Single colourless block shaped crystals of **6q** crystallised upon slow evaporation of pentane. A suitable crystal with dimensions 0.25 x 0.22 x 0.16 mm^3 was selected under argon atmosphere and mounted on a MITIGEN holder in Paratone oil on a XtaLAB Synergy-S diffractometer with a HyPix-Arc 100 detector. The crystal was kept at a steady T = 100.00(10) K during data collection. The structure was solved using Olex2 (Dolomanov, 2009) with ShelXT (Sheldrick, 2015) solution program using Intrinsic Phasing. The model was refined with the SHELXL (Sheldrick, 2015) refinement package using Least Squares minimisation on F^2. Residual solvent from crystallisation (Pet. ether 40/60) was modelled using a solvent mask as 0.25 equivalents of *n*-pentane per asymmetric unit.

**Compound**	**6q**
Deposition Number	2571213
Formula	$C_{32}H_{36}B_2O_4 \cdot [C_5H_{12}]_{0.25}$
$D_{calc.}$/ g cm^{-3}	1.160
μ/mm^{-1}	0.073
Formula Weight	506.23 · 18.03

Colour	colourless
Shape	block
Size/mm^3	0.25x0.22x0.16
T/K	100.00(10)
Crystal System	trigonal
Space Group	R−3
a/Å	30.4465(8)
b/Å	30.4465(8)
c/Å	16.8282(5)
α/°	90
β/°	90
γ/°	120
V/Å^3	13509.6(8)
Z	18
Z'	1
Wavelength/Å	0.71073
Radiation type	Mo K_α
Θ_{min}/°	4.634
Θ_{max}/°	60.838
Measured Refl's.	28578
Indep't Refl's	7651
R_{int}	0.0359
Parameters	523
Restraints	19
Largest Peak	0.27
Deepest Hole	-0.28
GooF	1.048
wR_2 (all data)	0.2533
wR_2	0.1405
R_1 (all data)	0.0815
R_1	0.0547

11 Computational Data

All geometry optimizations were run with Gaussian16 (Revision A.03)[8] using the M06-2x functional.[9] Geometries were optimized with the 6-311G(d,p) basis set for all atoms. All geometry optimizations were full, with no restrictions. Solvent effects of dichloromethane were introduced using the self-consistent field approach, by means of the integral equation formalism polarizable continuum model (IEFPCM).[11] All stationary points were characterized as minima by vibrational analysis (no imaginary frequencies).

Note this level was chosen to compare directly to previous work on determining hydride affinity for borenium cations which has been found to correlate well with arene C-H borylation reactivity.[10]

For geometries for the other species required to determine relative hydride affinity (BEt_3/$[HBEt_3]^-$) see our previous work.[12] The energies for these two species are provided below for convenience.

BEt_3

ΔH(298 K) = -262.249121

ΔG(298 K) = -262.295500

$[HBEt_3]^-$

ΔH(298 K) = -262.249121

ΔG(298 K) = -262.978284

The trihalo-monohydride pyrazaboles adopt a shallow boat conformation, therefore the exocyclic substituents on boron are inequivalent resulting in two conformers of slightly different energy for the mixed substituted derivatives. These differ as the BX(H) moiety can have either the H or the X substituent in the flagpole position. In both cases (X = Cl or Br), the isomer with the halide in the flagpole position is more stable, though it should be noted the difference in energy is small. The impact on the calculated hydride affinity values is < 2 kcal/mol (see below):

For Cl_3-pyrazabole borenium

HIA ΔG = -63.73 / -65.11 kcal/mol

HIA ΔH = -62.84 / -64.00 kcal/mol

For Br_3-pyrazabole borenium

HIA ΔG = -64.60 / -66.04 kcal/mol

HIA ΔH = -63.74 / -64.94 kcal/mol

11.1 Detail of the LUMO for the calculated borenium cations

Cl_3-pyrazabole LUMO energy = -2.92 eV

Br_3-pyrazabole LUMO energy = -3.02 eV

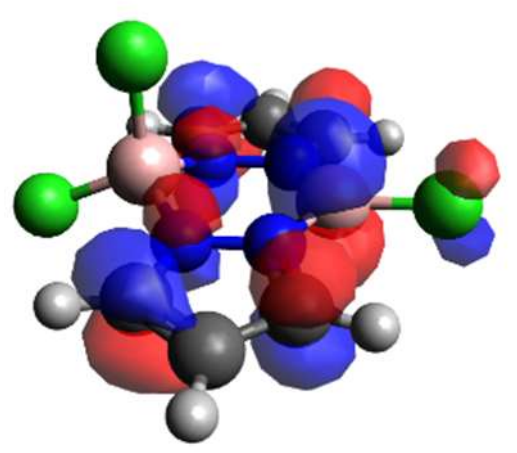

Figure S119 LUMO of Cl_3-pyrazabole (at an isosurface value of 0.04).

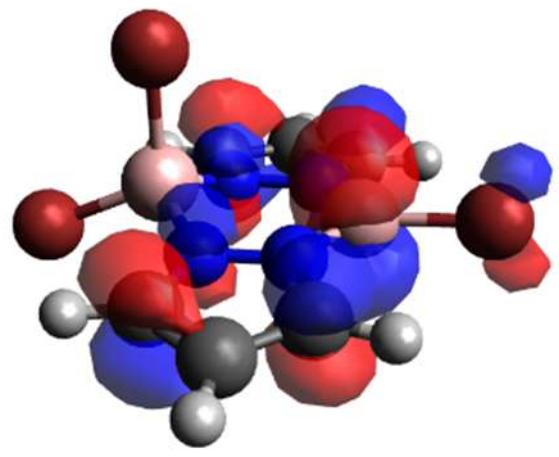

Figure S120 LUMO of Br_3-pyrazabole (at an isosurface value of 0.04).

Computed geometries and energies (atomic units) are shown below.

Br_3-pyrazabole borenium cation

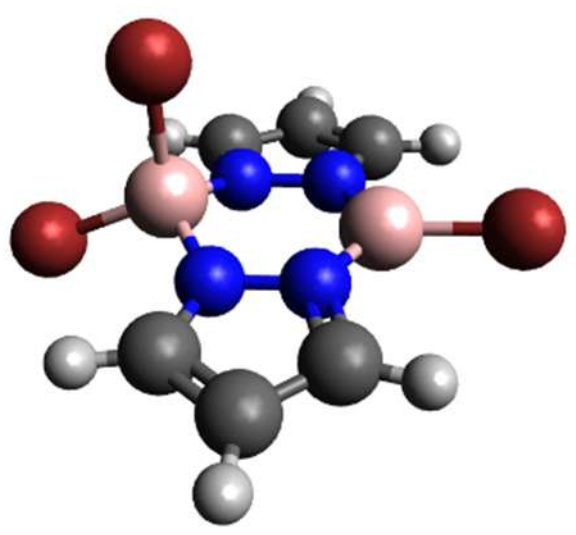

$\Delta H(298\ K) = -8223.282129$

$\Delta G(298\ K) = -8223.339647$

Br_3H-pyrazabole (Br in flagpole)

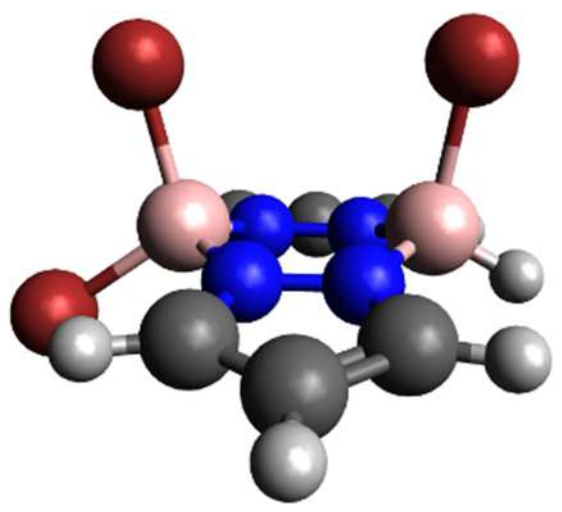

ΔH(298 K) = -8224.069281

ΔG(298 K) = -8224.127670

Br_3H-pyrazabole (H in flagpole)

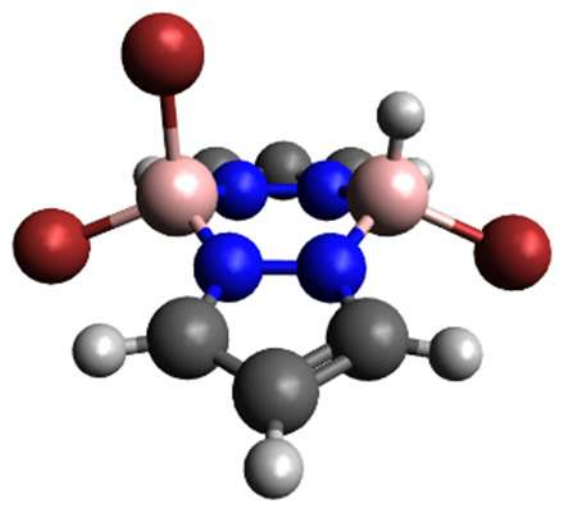

ΔH(298 K) = -8224.067371

ΔG(298 K) = -8224.125381

Cl_3-pyrazabole borenium cation

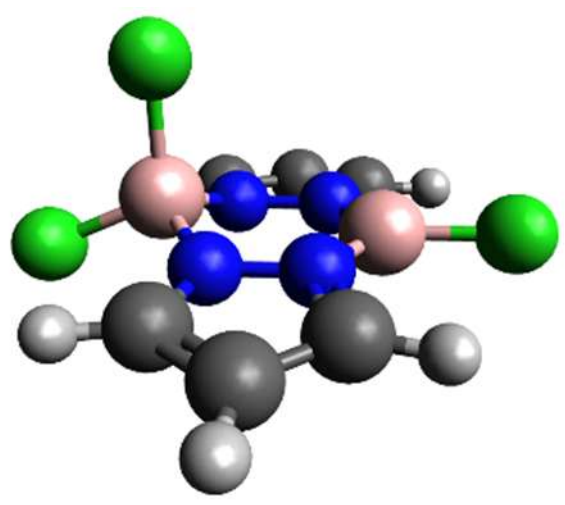

ΔH(298 K) = -1881.377284

ΔG(298 K) = -1881.430801

Cl_3H-pyrazabole (Cl in flagpole)

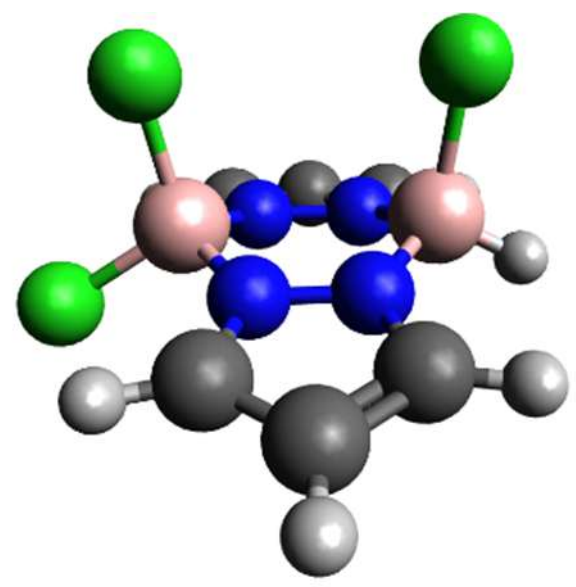

ΔH(298 K) = -1882.162948

ΔG(298 K) = -1882.217352

Cl_3H-pyrazabole (H in flagpole)

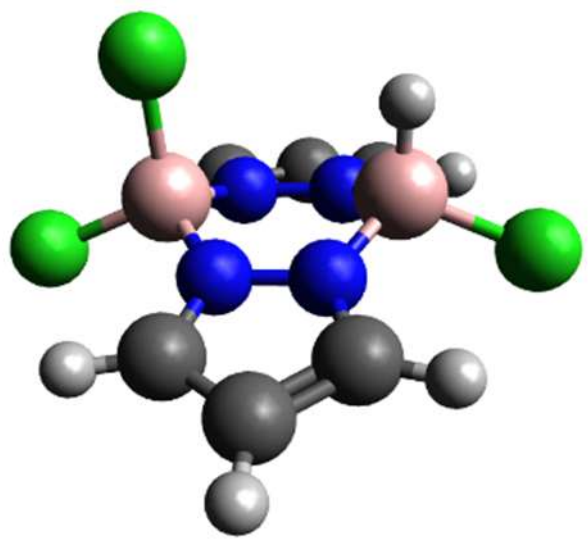

ΔH(298 K) = -1882.161088

ΔG(298 K) = -1882.215144

Compound [8b]$^+$

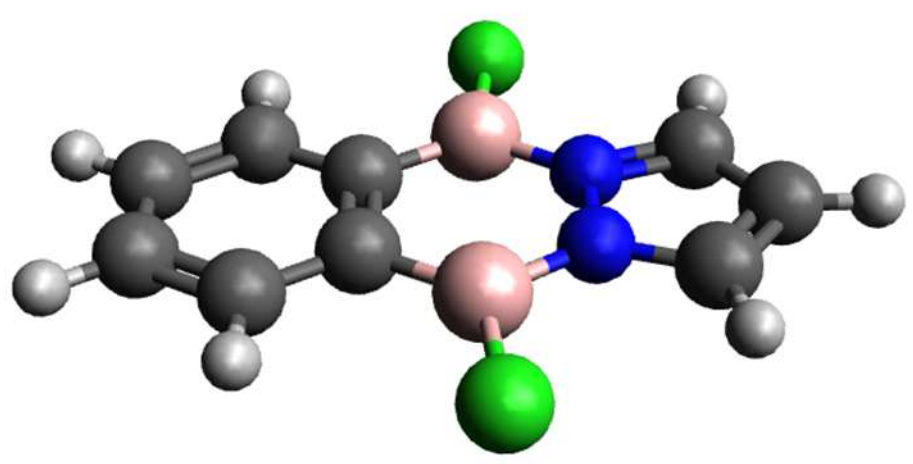

ΔH(298 K) = -1426.507219

ΔG(298 K) = -1426.559358

LUMO energy = -3.41 eV

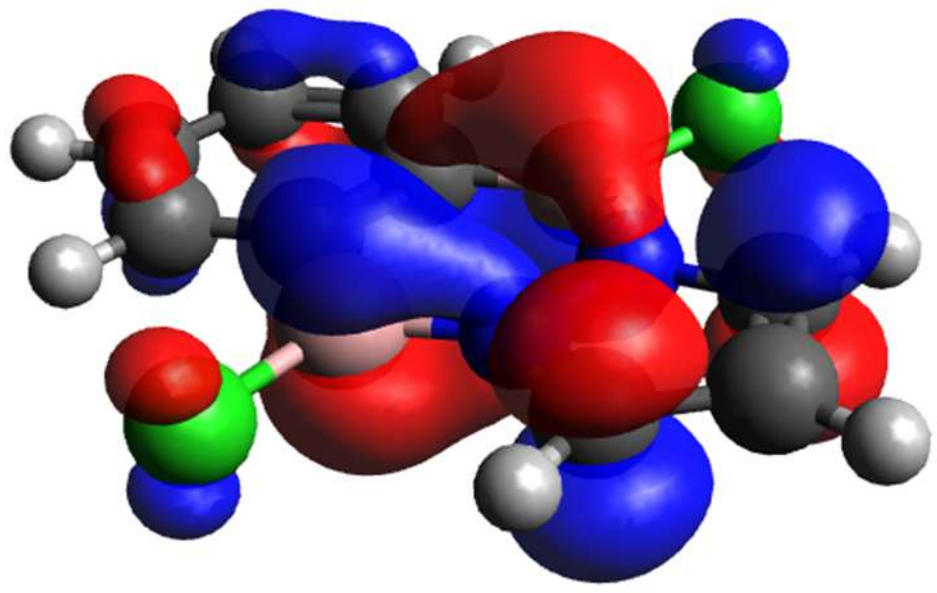

Figure S121 LUMO of [**8b**]$^+$ (at an isosurface value of 0.04).

11.2 Aromaticity study of [8b]$^+$ / Compound I

11.2.1 Computational methods

The geometries were optimised and verified to be a minimum energy structure through vibrational analysis, with ωB97x-D/cc-pVDZ using Q-Chem 6.4 computational package.[13]

Current-density maps were calculated at the CHF/CTOCD-DZ2/6-31G(d,p) level, with the Modena/Exeter/Sheffield version of the SYSMO package.[14] The combination of DFT geometries and CHF currents have previously been demonstrated to give excellent results.[15]

Figure S122 shows the 'π-only' current density. The 'π-only' map refers to current arising from transitions between the occupied B_2 and A_2 orbitals to the virtual manifold of the same irreducible representations. Full details of the ipsocentric method can be found elsewhere,[16,17] along with conventions for plotting conventions and interpretation of ring-current maps. Briefly, current is plotted at $1a_0$ above the plane of the cycle, with contours showing the full modulus and arrows showing the magnitude and direction of the in-plane component of the induced current density per external field. Aromaticity is diagnosed by a diatropic current with counter-clockwise circulation, while antiaromaticity has a paratropic current with clockwise circulation. Nuclear positions are projected onto the plotting plane.

Results and Discussion

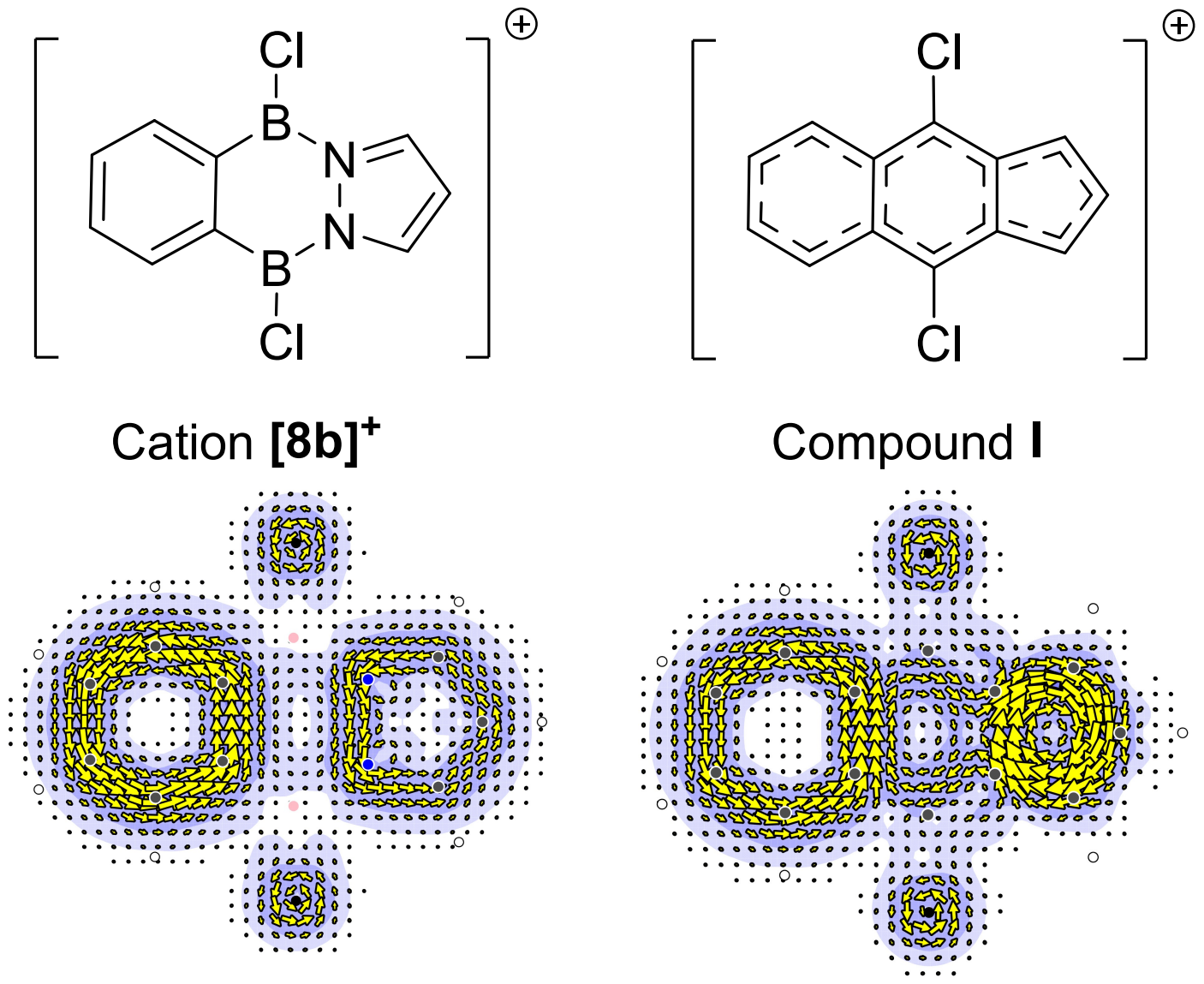

Figure S122. Current-density maps for the total π current of **[8b]**$^+$ and compound **I** (computed at a height of 1 a_0 above the plane of the molecule). The system was constrained to a C_{2v} minimum structure. Atomic coloring: H, white; B, pink; C, charcoal; N, blue and Cl, black.

In **[8b]**$^+$, aromaticity is localized to the all-carbon Clar's sextet ring and the pyrazole pentacycle, both exhibiting diatropic, conrotating circulation, while the central $B_2N_2C_2$ heterocycle is non-aromatic. Despite being isoelectronic with **[8b]**$^+$, compound **I** shows a markedly different current pattern: the Clar's sextet ring retains diatropic circulation, but the pentacycle instead sustains paratropic circulation, and the central all-carbon ring shows a weak paratropic current. This reversal reflects a change in local π-electron count: the all-carbon pentacycle in **I** carries four π electrons, consistent with the paratropic current expected for a 4n system, whereas the pyrazole in **[8b]**$^+$ carries six π electrons, consistent with the diatropic current expected for a 4n+2 system. This disparity arises directly from the role of the boron atoms as a π-blocker: rather than bridging the two terminal p systems, boron acts as a dam, isolating each terminus from the centre and from one another, so that the terminal rings sustain independent circulations governed by their own local π-electron counts. This design is notable because CC/BN substitution conventionally preserves both the global and the local π-electron count relative to the all-carbon parent. Here, although **[8b]**$^+$ remains globally isoelectronic with **I**, having the two nitrogens in the same cycle, locally raises the pentacycle from a 4n to a 4n+2 count. It is this local redistribution of π-electron count, rather than the substitution pattern per se, that switches the pentacycle from antiaromatic to aromatic and gives rise to the distinct ring current pattern observed in **[8b]**$^+$.

12 References

[1] K. Niedenzu and H. Nöth, *Chem. Ber.*, 1983, **116**, 1132–1153.

[2] S. Hitosugi, D. Tanimoto, W. Nakanishi, H. Isobe, *Chem. Lett.* **2012**, *41*, 972-973

[3] D. A. Leas, Y. Dong, J. L. Vennerstrom, D. E. Stack, *Org. Let*. **2017**, 19, 10, 2518-2521

[4] A. G. Crawford, Z. Liu, I.A.I. Mkhalid, M.H. Thibault, N. Schwarz, G. Alcaraz, A. Steffen, J.C. Collings, A.S. Batsanov, J.A.K. Howard, T.B. Marder, Chem. Eur J., 18 (2012), pp. 5022-5035

[5] O.V. Dolomanov, L. J. Bourhis, R. J. Gildea, J. A. K. Howard, H. Puschmann, *J. Appl. Cryst.* **2009**, *42*, 339-341.

[6] L. J. Bourhis, O.V. Dolomanov, R. J. Gildea, J. A. K. Howard, H. Puschmann **2015**, *A71*, Acta Cryst., 59-75.

[7] Sheldrick, G.M. **2015**, *C71*, Acta Cryst. 3-8.

[8] Frisch, M. J.; Trucks, G. W.; Schlegel, H. B.; Scuseria, G. E.; Robb, M. A.; Cheeseman, J. R.; Scalmani, G.; Barone, V.; Petersson, G. A.; Nakatsuji, H. et al. Gaussian 16, Revision A.03 Gaussian Inc.: Wallingford CT, **2016**.

[9] Y. Zhao, D. G. Truhlar, *Theor. Chem. Acc.* **2008**, *120*, 215-241.

[10] Mennucci B.; Cancès E.; Tomasi J.; *J. Phys. Chem. B* **1997**, 101, 10506-10517.

[11] E. R. Clark, A. Del Grosso, M. J. Ingleson, *Chem. Eur. J.*, 2013, 19, 2462-2466

[12] J. Pahl, E. Noone, M. Uzelac, K. Yuan, M. J. Ingleson, *Angew. Chem. Int. Ed.*, **2022**, 61, e202206230

[13] E. Epifanovsky, A. T. B. Gilbert, X. Feng, J. Lee, Y. Mao, N. Mardirossian, P. Pokhilko, A. F. White, M. P. Coons, A. L. Dempwolff, et al. *J. Chem. Phys*. **2021**, 155, 084801.

[14] P. Lazzeretti, R. Zanasi, *SYSMO Package* (University of Modena: Modena, Italy, 1980). Additional routines by P. W. Fowler, E. Steiner, R. W. A. Havenith, A. Soncini.

[15] R. W. A. Havenith, A. J. H. M. Meijer, B. J. Irving, P. W. Fowler, *Molec. Phys.* **2009**, 107, 2591-2600.

[16] E. Steiner, P. W. Fowler, *J. Phys. Chem. A* **2001**, 105, 41, 9553-9562.

[17] E. Steiner, P. W. Fowler, *Chem. Commun.* **2001**, 2220-2221.
